# Revealing Relationships Among Cognitive Functions Using Functional Connectivity and a Large-Scale Meta-Analysis Database

**DOI:** 10.3389/fnhum.2019.00457

**Published:** 2020-01-10

**Authors:** Hiroki Kurashige, Jun Kaneko, Yuichi Yamashita, Rieko Osu, Yohei Otaka, Takashi Hanakawa, Manabu Honda, Hideaki Kawabata

**Affiliations:** ^1^Institute of Innovative Science and Technology, Tokai University, Tokyo, Japan; ^2^National Institute of Neuroscience, National Center of Neurology and Psychiatry, Tokyo, Japan; ^3^Faculty of Human Sciences, Waseda University, Tokyo, Japan; ^4^Department of Rehabilitation Medicine I, School of Medicine, Fujita Health University, Aichi, Japan; ^5^Department of Rehabilitation Medicine, Tokyo Bay Rehabilitation Hospital, Chiba, Japan; ^6^Integrative Brain Imaging Center, National Center of Neurology and Psychiatry, Tokyo, Japan; ^7^Department of Psychology, Keio University, Tokyo, Japan

**Keywords:** human brain, fMRI, meta-analysis database, functional connectivity, network analysis, data mining, machine learning

## Abstract

To characterize each cognitive function *per se* and to understand the brain as an aggregate of those functions, it is vital to relate dozens of these functions to each other. Knowledge about the relationships among cognitive functions is informative not only for basic neuroscientific research but also for clinical applications and developments of brain-inspired artificial intelligence. In the present study, we propose an exhaustive data mining approach to reveal relationships among cognitive functions based on functional brain mapping and network analysis. We began our analysis with 109 pseudo-activation maps (cognitive function maps; CFM) that were reconstructed from a functional magnetic resonance imaging meta-analysis database, each of which corresponds to one of 109 cognitive functions such as ‘emotion,’ ‘attention,’ ‘episodic memory,’ etc. Based on the resting-state functional connectivity between the CFMs, we mapped the cognitive functions onto a two-dimensional space where the relevant functions were located close to each other, which provided a rough picture of the brain as an aggregate of cognitive functions. Then, we conducted so-called conceptual analysis of cognitive functions using clustering of voxels in each CFM connected to the other 108 CFMs with various strengths. As a result, a CFM for each cognitive function was subdivided into several parts, each of which is strongly associated with some CFMs for a subset of the other cognitive functions, which brought in sub-concepts (i.e., sub-functions) of the cognitive function. Moreover, we conducted network analysis for the network whose nodes were parcels derived from whole-brain parcellation based on the whole-brain voxel-to-CFM resting-state functional connectivities. Since each parcel is characterized by associations with the 109 cognitive functions, network analyses using them are expected to inform about relationships between cognitive and network characteristics. Indeed, we found that informational diversities of interaction between parcels and densities of local connectivity were dependent on the kinds of associated functions. In addition, we identified the homogeneous and inhomogeneous network communities about the associated functions. Altogether, we suggested the effectiveness of our approach in which we fused the large-scale meta-analysis of functional brain mapping with the methods of network neuroscience to investigate the relationships among cognitive functions.

## Introduction

Ones of main missions of cognitive neuroscience and psychology is to understand each cognitive function *per se* and to understand the human brain as an aggregate of cognitive functions. To this end, it is vital to relate dozens of cognitive functions, which will provide integrated views for the entire cognition in the human brain and will enable to characterize each cognitive function by relating it to others. Such understanding will provide testable hypotheses for the cognitive neuroscience and psychology communities. In addition, it will give the artificial intelligence community guidelines and ideas to develop novel brain-inspired AI algorithms (Hassabis et al., 2017).

Several efforts in psychology have been conducted to reveal hidden relationships among cognitive functions. Developing atlases and/or ontologies for psychological concepts is one of these endeavors to do so (Price and Friston, 2005; Poldrack et al., 2011; Turner and Laird, 2012; Poldrack and Yarkoni, 2016). Using such ontological data has been shown to be efficient for probing the neural bases of cognitive functions (Varoquaux et al., 2018). Therefore, we consider that building atlases and ontological databases for psychological constructs are promising approaches. However, currently existing atlases and ontological databases are highly conceptual but not sufficiently empirical, which means that most of the relationships are proposed based on the ‘common senses’ in psychology. It also may lead to missing many meaningful relationships latent in the experimental data which have become big data nowadays. Another effort is to compare cognitive concepts (or psychological constructs) with each other by trying to identify relationships in idiosyncratic features or performances in several cognitive tasks (Beaty et al., 2014; Chuderski and Jastrzêbski, 2018; Eisenberg et al., 2019; Fuhrmann et al., 2019; Rey-Mermet et al., 2019) as well as by investigating overlaps in neural substrates using neuroimaging and neuropsychological methods (Hassabis et al., 2007; Mullally and Maguire, 2014; Woolgar et al., 2018; Brandl et al., 2019; Jonikaitis and Moore, 2019). While these approaches provide insights based on empirical facts, completing such low-profile tasks exhaustively is challenging.

The magnitude of such exhaustive explorations of common or dissociated neural bases among many cognitive functions may dampen the willingness of identification of relationships among them. However, leveraging neuroscientific knowledge is still expected to be effective to our aim because the cognitive functions that overlapping brain regions are responsible for should be interrelated. Additionally, we also consider that the cognitive functions that connected brain regions are responsible for should be interrelated. Therefore, the use of large-scale meta-analysis databases with knowledge about network topology of the brain is essential to find relationships among cognitive functions to characterize each function and the entire cognition in the brain.

BrainMap (Laird et al., 2005, 2011a; Laird, 2009) and Neurosynth (Yarkoni et al., 2011; Poldrack et al., 2012) are databases specialized toward linking cognitive functions to brain regions. The former is a manually constructed database and includes activation coordinates and ontological data (e.g., behavioral domain, task paradigm, and stimulus modality) reported in fMRI studies. The latter is an automated database including activation coordinates and terms extracted from fMRI studies. We can reconstruct pseudo-activation patterns underlying the reports in each study using the stored activation coordinates. Therefore, we are able to relate cognitive functions investigated in the study to the (pseudo-)activation patterns. For instance, the BrainMap’s team proposed an approach to provide interpretations of independent components of brain activity based on the cognitive functions (Smith et al., 2009; Laird et al., 2011b; Ray et al., 2013). In another instance, brain parcellation related to cognitive functions was performed by applying decoding based on the cognitive data in BrainMap to parcels identified using connectivity data from the Human Connectome Project database (Fan et al., 2016). In addition, a Bayesian topic model that relates components of cognitive functions to well-localized brain regions was developed (Rubin et al., 2017). This enables decoding of functionality, expressed as rich text information, from any pattern of brain activity. The approaches using BrainMap or Neurosynth are effective for identifying functionalities of sub-divided brain areas, such as the temporoparietal junction (Bzdok et al., 2013), the dorsolateral prefrontal cortex (Cieslik et al., 2013), the insula (Chang et al., 2013), the striatum (Pauli et al., 2016), and the medial frontal cortex (de la Vega et al., 2016). More generally, we can construct pseudo-activation maps corresponding to various cognitive functions (Yarkoni et al., 2011). Hereafter, we term such a pseudo-activation map cognitive function map (CFM).

Here, we explore the relationships among dozens of cognitive functions on the basis of two simple assumptions: (1) cognitive functions that overlapping brain regions are responsible for should be interrelated, and (2) cognitive functions that connected brain regions are responsible for should be also interrelated. To this end, we analyze the CFMs derived from the meta-analysis database with resting-state functional connectivity (RSFC). Therefore, we consider the relationships among cognitive functions from a network neuroscience perspective, which is the subfield of neuroscience to reveal complex but well-organized interdependencies among brain regions using the methods of network analysis (Sporns and Kötter, 2004; Sporns et al., 2007; Bassett et al., 2008; Hagmann et al., 2008; van den Heuvel et al., 2008; Bullmore and Sporns, 2009, 2012; Power et al., 2011, 2013; van den Heuvel and Sporns, 2011; Zuo et al., 2012; Bertolero et al., 2015; Fornito et al., 2016; Nigam et al., 2016; Bassett and Sporns, 2017; Hwang et al., 2017). We use these methods to reveal relationships among cognitive functions.

In the present study, we analyzed 109 cognitive functions from the viewpoints of connectivity and network analysis using RSFC. First, we provide a comprehensive view of those cognitive functions by constructing relational mapping among them based on the distances quantified as the strengths of RSFCs between the CFMs. This facilitates understanding the brain as a relational network among cognitive functions. Then, we conducted so-called conceptual analysis (in philosophy) of each cognitive function by sub-dividing corresponding CFM on the basis of the connectivity between each voxel within the CFM and the other CFMs, resulting in decomposition of the concept of the function into several sub-concepts. Next, by applying clustering analysis to the whole-brain voxel-to-CFM RSFC, we constructed a whole-brain parcellation where each parcel is labeled with a vector whose components are the degrees of associations to the 109 cognitive functions. Then, by applying matrix factorization to the matrix constructed by concatenating these vectors, we identified six cognitive factors, including ‘concept processing,’ ‘action and expression,’ ‘vision and attention,’ ‘executive function,’ ‘value and judgment,’ and ‘memory.’ Each parcel had degrees of contributions with those factors. Using methods of network analysis to characterize the network consisting of the parcels, we quantified the diversity of the information sources/receivers for the six factors, identified three densely connected subnetworks which are specialized for ‘concept processing,’ ‘action and expression,’ and ‘vision and attention,’ and found (un-)uniformity of factors associated with the parcels within each network community.

The goals of our research are to exhaustively reveal relationships among cognitive functions and relationships between cognitive functions and network characteristics in the brain. Although several previous studies partially suggested such relationships by focusing on some part of the cognitive functions, to the best of our knowledge, there has been no exhaustive effort to those subjects, at least explicitly. Therefore, the main contribution of the present study is, firstly, to provide promising ways to construct comprehensive knowledge of organizations of dozens of cognitive functions as exhaustively as possible. Moreover, we also contribute to providing hopeful ways to reveal relationships between dozens of cognitive functions and network characteristics in the brain. Indeed, we found several new insights into the relationships among cognitive functions and the relationships between cognitive functions and network characteristics. These were achieved by the fusion of large-scale meta-analysis of studies of functional brain mapping and methods in the network analysis.

Taken together, we suggest the effectiveness of our approach in which we fused the large-scale meta-analysis of a task-based fMRI database with network neuroscience approaches to investigate the relationships among cognitive functions to understand each cognitive function *per se* and the human brain as a relational system consisting of cognitive functions.

## Materials and Methods

### Subjects

Fifty-two subjects (21 women) without a history of neurological or psychiatric diseases participated in this study. The mean ages of the male and female subjects were 21.5 and 22.3 years (standard deviation, 1.27 and 6.94 years), respectively. All subjects were right-handed. They had a normal or corrected-to-normal vision. We did not use any power analysis to determine the sample size but decided the size by reference to previous resting-state fMRI studies (e.g., Fox et al., 2005; Honey et al., 2009; Smith et al., 2009). To recruit participants, we mainly used announcements through Web sites (including SNS) and snowball sampling.

The study was performed in accordance with the recommendations of the institutional ethics committee of the National Center of Neurology and Psychiatry (NCNP), with written informed consent from all subjects, in accordance with the Declaration of Helsinki. The institutional ethics committee of the NCNP approved the study protocol (Approval No. A2014-072).

### MRI Acquisition

We used a 3T MRI scanner (Trio, Siemens Medical Solutions, Erlangen, Germany) with an 8-channel head coil for all measurements. Structural images were acquired using a T1-weighted 3D magnetization-prepared-rapid-gradient-echo sequence. The parameters used were: flip angle = 8°, voxel size = 1 mm isotropic, TR = 2000 ms, TI = 990 ms, TE = 4.38 ms, and number of voxels = 208 × 256 × 208. Functional images were acquired with a T2^∗^-weighted echo-planar imaging sequence. The parameters used were: flip angle = 90°, voxel size = 3 mm (isotropic, with no slice gap), TR = 3000 ms, TE = 30 ms, and number of voxels = 64 × 64 × 44. The slices were acquired in interleaved order.

### Resting-State fMRI

We acquired 148 volumes of images. As TR was 3 s, the total acquisition time was approximately 7.4 min. During imaging, a fixation point centered on a gray background was presented. We instructed the subjects to look at the fixation point and to think of nothing in particular.

### Preprocessing of MRI Data

We performed the preprocessing mainly using FSL (FMRIB Software Library Version 5.0.6^1^) (Jenkinson et al., 2012). All steps were executed by running commands in FSL from custom-made shell scripts.

First, we applied slice-time correction to functional images using the slicetimer command. Next, we conducted head motion correction using the mcflirt command (Jenkinson et al., 2002) with the ‘-stages 4 -sinc_final -meanvol -mats -plots’ option. Afterward, we applied the bet command (Smith, 2002) to structural images to extract the brain regions from whole images. For these structural images, the flirt command (Jenkinson and Smith, 2001; Jenkinson et al., 2002; Greve and Fischl, 2009) was executed with the MNI152_T1_2mm_brain template as a reference. In this step, we used six degrees of freedom, resulting in rigid-body transformation. Therefore, we executed this step only for alignment and changing the resolution to 2 mm. Then, we applied the bet command to the mean functional image and obtained registration parameters of the image to the 2-mm-resolution structural image using the flirt command. Using these parameters, we registered all functional images to the 2 mm-resolution structural image, resulting in 2-mm-resolution functional images. Next, we obtained non-linear transformation parameters by applying the fnirt command to the 2-mm-resolution structural image, with the MNI152_T1_2mm template as a reference. Then, we transformed the 2-mm-resolution functional images using the applywarp command with the non-linear transformation parameters. This yielded 2-mm-resolution functional images that were standardized into the Montreal Neurological Institute (MNI) 152 space. Additionally, we masked these functional images with the regions of the MNI152 standard brain and smoothed them with a 5-mm full-width at half-maximum. These functional images were used in the following analyses.

Additionally, the structural image was standardized into the 1-mm-resolution MNI 152 space followed by the recon-all process in Freesurfer (version 5.3.0^2^). This yielded cortical and subcortical atlases (Fischl et al., 2002; Desikan et al., 2006) standardized into the 1-mm-resolution MNI 152 space.

In the analyses for the resting-state fMRI shown in the following subsections, we excluded subjects whose translational head motions were 1 mm or more or whose rotational head motions were 1° or more, since head motion severely affects the inference of RSFC (Power et al., 2012; van Dijk et al., 2012). Our criterion is more stringent compared with the conventional criteria from previous studies (Guo et al., 2012; Jackson et al., 2016; Liu et al., 2016; Zhu et al., 2017). According to the criterion, we excluded twenty-five subjects We did not adopt any other criterion for excluding data.

### Whole-Brain Anatomical Atlas

To construct a whole-brain anatomical atlas, we used the output files of the recon-all process in Freesurfer. As described above, the input file for the process was an individual structural image standardized into the MNI152 space. Therefore, the output file provided the whole-brain atlas for each subject standardized into the MNI152 space. In this atlas, each voxel is labeled with an intensity to specify the anatomical area according to the Freesurfer convention.

We decomposed the whole-brain atlas for each subject to the anatomical regions. For each region, we aggregated the atlases for all subjects into one average atlas by the following method. First, for each voxel, we counted the number of subjects whose individual atlases for the region included the voxel and assigned it to the voxel. Then, we binarized the resulting image with a threshold of the number of subjects for the inclusion of voxels into the aggregated atlas, which made the number of voxels in the image closest to the mean of the number of voxels composing the region across the subjects. This provided an average atlas across the subjects for the anatomical regions. Finally, we merged these average atlases into one whole-brain anatomical atlas on the MNI152 standardized brain. In this whole-brain atlas, each voxel is labeled with the intensity indicating the corresponding anatomical region in a manner following the Freesurfer convention.

### Construction of Pseudo-Activation Maps

We constructed a pseudo-activation map for each cognitive function. To this end, we followed the method based on χ^2^ statistics described previously (Yarkoni et al., 2011). We will give an in-depth explanation of the procedure in the remainder of this section. In the procedure, we used version 0.4 of Neurosynth data downloaded from the Neurosynth page on GitHub^3^.

First, for the articles registered in Neurosynth data, we obtained titles, keywords, and abstracts by accessing PubMed^4^ using the Entrez Programing Utilities (E-utilities) API^5^ executed from the Biopython module (Cock et al., 2009) in Python. Then, we counted the appearances of cognitive concepts in the title, keywords, and abstract for each article. As for the cognitive terms considered in this study, we prepared 702 concepts. Of these, 692 were extracted from the list named ‘concepts’ in Cognitive Atlas (Poldrack et al., 2011). The extraction date was 8/18/2014. We added ten cognitive terms. The cognitive terms that we considered are listed in Supplementary Table S1.

We considered a cognitive term to be present in an article if the term appeared one or more times per 100 words in the text merged from the title, keywords, and abstract of the article. We included only the cognitive terms that appeared in ten or more articles in the following analyses. Additionally, we discarded the terms that are used as general words in neuroscience literature, such as ‘focus’ and ‘strength.’ Thus, we selected the 121 cognitive terms shown in Supplementary Table S2 as the targets to be considered in this study.

Then, we reconstructed the binary activation map on the 2-mm-resolution MNI 152 brain for each article registered in Neurosynth data by the following steps. First, we transformed the coordinates reported in the Talairach brain into the MNI brain using icbm2tal transform (Lancaster et al., 2007). Then, we assigned the number ‘1’ to the voxels located within 10 mm of the registered coordinates and the number ‘0’ to the other voxels. Based on these binary activation maps, we calculated the χ^2^ statistics for each cognitive term, in which we compared the appearance of the term and activation of the voxel. Additionally, we calculated the ϕ coefficients corresponding to the χ^2^ statistics. Thus, we obtained χ^2^ and ϕ maps for each cognitive term. For convenience, in the following statistical test, these maps were masked by voxels that were activated in 3% or more articles, which reduced the number of voxels to be tested.

Based on the χ^2^ test using the χ^2^ map, we constructed a pseudo-activation map for each cognitive function in the following manner. We executed multiple-test corrections using the Benjamini–Hochberg procedure for controlling the false discovery rate (Benjamini and Hochberg, 1995) with *q*^∗^ = 0.05. This yielded a significant mask for each cognitive function. Additionally, we constructed a mask for each cognitive function where the positive values of ϕ coefficients indicated a positive correlation. Applying these masks to χ^2^ or ϕ maps, we obtained pseudo-activation maps where the pattern of significant positive activation induced with the cognitive function is expressed. We call these pseudo-activation maps CFMs. In the present study, 109 CFMs had significant voxels. Therefore, we focused on these 109 cognitive functions (Supplementary Table S3).

### Two-Dimensional Embedding of Cognitive Concepts Based on the CFM-to-CFM RSFC Matrix

We constructed a two-dimensional relational map among the 109 cognitive functions based on the time-series data of blood-oxygen-level-dependent (BOLD) signals for each CFM. First, we extracted the time-series data of BOLD signals of resting-state fMRI for each voxel in the whole-brain mask. To reduce artifacts due to motion and signal drift, six head motion parameters and six differential values of head motion plus the linear trend and constant component were regressed out. Then, a 0.009–0.08 Hz band-pass filter was applied to remove the putative non-neuronal signals according to previous reports (Biswal et al., 1995; Cordes et al., 2001; Lu et al., 2007; Zuo et al., 2010). We used the 5th-order Butterworth digital filter. This filter was applied in forward and backward. We confirmed that further increase of the order led to little change the resulting waveform. In addition, the average signals of the gray matter region, white matter region, and ventricles were regressed out. Those data were transformed to *Z*-scores by each voxel to erase the intensity bias between the voxels. For all voxels for all subjects, the maximum and minimums *Z*-scores were 5.32 and −4.89, respectively. By applying the Kolmogorov–Smirnov test to each voxel of each subject, we found that 0.38% voxels were judged as non-normal distributions. Such a preprocessing flow was used also in the further analyses described below.

Then, for each subject, we obtained the mean signal for each CFM by averaging the signals across voxels in the CFM. We calculated the correlation matrices between signals of CFMs and averaged them across the subjects, resulting in the CFM-to-CFM RSFC matrix. By shifting and scaling the RSFC values, we obtained the CFM-to-CFM similarity matrix in which the minimum and maximum values were 0 and 1, respectively. Applying spectral clustering (see the next paragraph) to the similarity matrix, we identified clusters of cognitive functions. In this step, we determined the number of clusters as the value corresponding to the maximum of silhouette coefficients (Rousseeuw, 1987) up to 12 (Supplementary Figure S1).

The reason why we used the spectral clustering to identify the clusters of cognitive functions is that our problem in this analysis was based on the similarity matrix (not on the feature vectors). For convenience to the readers, we give a brief introduction to spectral clustering (von Luxburg, 2007). The procedure of the spectral clustering consists of two steps. The first step is to embed data into a representational space. In this space, coordinates (or representations) of the data are determined to minimize a loss defined with the similarity matrix and the coordinates. This minimization problem is reduced to the eigenvalue problem. Except for parameters used in the numerical calculus to solve the eigenvalue problem, the parameter that we need to set is the dimension of the representational space that is equal to the number of eigenvectors that we consider. Throughout the present study, we set this value to the same as the number of clusters. The second step is to cluster the data based on the coordinates in the representational space. In this step, we need to determine the way to cluster. Here, we used k-means clustering.

Finally, we applied multidimensional scaling (Borg and Groenen, 1997) to the CFM-to-CFM RSFC matrix using the scikit-learn module in Python and obtained the relational map that involves two-dimensional embedding of the 109 cognitive functions, in which the well-correlated pairs of cognitive functions were located as closely as possible. To check a distortion caused by the embedding, we calculated the stress that is defined as the difference between given dissimilarities and distances in the embedding space and is the value to be minimized in the multidimensional scaling.

### Subparcellation of CFMs

For each cognitive function and subject, the resting-state fMRI BOLD signals of the voxels in the corresponding CFM were extracted and preprocessed in the same manner as described in the previous sections. Now we focus on a CFM that we term target CFM. We calculated the correlation values between the processed signals of all voxels in the target CFM and the mean signals obtained from the other CFMs by averaging signals across the voxels belonging to the CFMs. These correlation values were averaged across the subjects. Thus, we obtained the target CFM voxel-to-CFM RSFC matrix. For instance, if we express the number of voxels in the ‘emotion’ CFM as *N* (emotion), the RSFC matrix has a dimension of *N*(emotion)×108, since we considered 109 cognitive functions.

Then, we executed principal component analysis for dimensionality reduction. We determined the number of principal components required to explain 95% of the total variance. Finally, we applied k-means clustering to the resulting data, in which we set the number of clusters as five.

### Whole-Brain Parcellation Based on Voxel-to-CFM Functional Connectivity

We conducted whole-brain parcellation based on RSFC. First, we extracted the time series data of BOLD signals of resting-state fMRI for each voxel in the whole-brain mask and calculated the mean signal for each CFM. These procedures were the same as those described above.

From these processed data, we obtained a matrix of voxel-to-CFM RSFC by calculating correlation coefficients between the processed BOLD signals of the 160,296 voxels in the whole-brain mask and the processed BOLD signals of the 109 CFMs for each subject. Then, we transformed them into Fisher’s *Z* values and averaged them across the subjects. We applied inverse Fisher’s *Z*-transform to this and obtained the mean voxel-to-CFM RSFC matrix in which each row was a 109-dimension feature vector for each voxel. To reduce the number of dimensions, we performed principal component analysis by solving the eigenvalue problem for the covariant matrix for the voxels. We determined the number of principal components required to explain 95% of the total variance. Based on this dimension-reduced matrix, we constructed a similarity matrix between voxels, where the similarity was defined as the exponential of the correlation coefficient between two voxels. To consider the spatial constraint, we set the similarity between the voxels that were not neighbored to 0, according to a previous study (Craddock et al., 2012), resulting in a sparse similarity matrix.

To obtain whole-brain parcellation, we applied multiclass spectral clustering (Yu and Shi, 2003) to this similarity matrix using the scikit-learn module in Python with ‘discretize’ (to use the optimal discretization approach searching a discrete partition closest to the continuous representations to identify data clusters in the representational space identified with spectral embedding) and ‘amg’ options. (The reason why we adopted the spectral clustering in this analysis was to use the spatial constraint mentioned above.) Since this algorithm requires the similarity matrix to be connected, we randomly chose 500,000 pairs of voxels and assigned them a weak positive value (0.0001). We set the number of clusters to 200 that was determined by reference to several existing atlases (Destrieux et al., 2010; Power et al., 2011; Shen et al., 2013; Baldassano et al., 2015; Fan et al., 2016). This resulted in whole-brain parcellation with 199 parcels. One cluster was discarded because it was empty (no voxel). We assigned each parcel a label vector that was the mean voxel-to-CFM RSFC obtained by averaging voxel-to-CFM RSFCs across the voxels belonging to the parcel, which represents the relatedness between the parcel and the 109 cognitive functions (parcel-to-CFM RSFC matrix).

### Dimensionality Reduction Using the Non-negative Matrix Factorization

We applied the non-negative matrix factorization (NMF) (Lee and Seung, 1999, 2001) to the parcel-to-CFM matrix to reduce the dimensionality, which was executed using the NIMFA module (Žitnik and Zupan, 2012) in Python. Before this process, we set the negative values in the matrix to 0.

The NMF is a method to decompose a non-negative data matrix (**X**) into a non-negative coefficient matrix (**Y**) and a non-negative basis matrix (**Z**). The objective of the NMF is to approximate **X** by **YZ**. Thus, we used the Frobenius norm ||**X−YZ**||_F_ as the cost function and minimized it subject to the **Y** ≥ 0 and **Z** ≥ 0. Our purpose in the dimensionality reduction was to identify well-interpretable low dimensional representations for the parcels. In the preprocessing procedure, we regressed the mean time-course of the gray matter signals out from the BOLD data. Although this is efficient to remove artifacts resulting from biological and equipment factors (Satterthwaite et al., 2013; Power et al., 2014; Li et al., 2019), it is suggested that this procedure tends to cause artifactual negative correlation (Murphy et al., 2009; Weissenbacher et al., 2009). Therefore, to lead better interpretation for the parcels, focusing only on the positive RSFCs is appropriate. Therefore, we chose the NMF as the way for dimensionality reduction.

The number of factors is a key parameter to be predefined in the NMF. A previous study suggests that the inflection point in the decrementing line of residual sum of squares (RSSs) with an increment of the values of the numbers of factors yields the adequate number (Hutchins et al., 2008). We can detect the inflection point as the crossing point between the curved lines fitted to RSSs before and after the point. Therefore, we first calculated the differentials of the RSSs and fitted them to straight lines. We repeated the linear regression and obtained the sums of the squared errors of before-point and after-point lines while changing the point. We determined the inflection point as the point realizing the minimum value of the summed squared error (Supplementary Figure S2). Using the value corresponding to the point as the number of factors, we conducted the NMF with singular value decomposition (SVD)-based initialization (Boutsidis and Gallopoulos, 2008).

Since the output vectors constituting the bases were not normalized, we scaled them to generate unit vectors and applied the inverse operation of the scaling to the coefficient matrix to keep the product invariant.

### Heat-Diffusion Analysis of Information Sources/Receivers

We extracted the time series data of BOLD signals of resting-state fMRI for each parcel in the whole-brain parcellation obtained above. These data were preprocessed in the same manner described in the previous sections. We calculated correlation coefficients between the processed BOLD signals of the parcels and obtained a parcel-to-parcel RSFC matrix averaged across subjects. We set the negative values and diagonal components in the matrix to 0 and treated it as an adjacency matrix **A**. In addition, we defined the degree matrix **D** in which the diagonal components were *D*_*ii*_ = ∑_*j*_*A*_*ij*_ and the other components were 0. From these matrices, we defined graph Laplacian matrix **L** = **D−A** (Chung, 1997), which is the homolog of the negative Laplacian −∇^2^.

For each NMF factor, we regarded the values of the NMF coefficients as the intensities of the heat sources distributed over the parcels. Based on the heat source distribution, we calculated the steady temperature distribution on the graph whose links were defined by the adjacency matrix **A** between parcels as graph nodes in the following manner, according to a procedure developed in network theory (Newman, 2010). In the usual partial differential equations, the temperature diffusion ψ with heat sources *f* is governed by the equation ∂⁡*ψ*/∂⁡t = *D*∇^2^⁡*ψ*−*βψ* + f, where *D* is a diffusion coefficient and β is a decay constant. As an analog of this equation for the graph, we obtained the equation ∂⁡ψ/∂⁡t = −*D*Lψ−*β*ψ + f, where **f** is the vector of heat source distribution defined as the NMF coefficient. As we consider the steady state ∂⁡ψ/∂⁡t = 0. Therefore, the steady temperature distribution is ψ = (*D***L** + *β***E**)^−1^f, where **E** is an identity matrix. We set *D=1* and *β* = 1 in the main analysis.

The temperature distribution was calculated for each NMF factor. The temperature of the parcels for each NMF factor represents a degree of information conveyed from the factor. Thus, each parcel has a vector of temperatures of the NMF factors. From the vector, we calculated the Gini coefficients that represent disparity of conveyed information among the NMF factors. If a parcel receives information from only one factor, the value of the Gini coefficient becomes 1. Conversely, if a parcel receives information from all factors uniformly, the value becomes 0.

We defined the Gini coefficient for each cognitive function as the mean of 10 Gini coefficients of parcels whose parcel-to-CFM RSFCs for the function were the top ten values. In other words, we averaged the ten Gini coefficients of parcels that were the most related to the cognitive function and considered the resulting mean as the Gini coefficient for the function.

In an additional analysis, we investigated the effects of the parameter values. Since the result is dependent only on the ratio of *D* and β, only *D* was varied and β was fixed (*β* = 1). Here, we compared the Gini coefficients between NMF factors. For each factor, we calculated an inner product between the vector of the Gini coefficients for cognitive functions defined above and the vector of the corresponding NMF basis that was normalized to make the summation one. We call this inner product weighted sum of the Gini coefficients. Intuitively, the weighted sum of the Gini coefficients expresses the mean of the Gini coefficients for the cognitive functions assigned to the factor.

### Local Density Identification in the Parcel-to-Parcel Network Using Clique Percolation

In this analysis, we first created the parcel-to-parcel network by defining the connectivity among parcels by thresholding the adjacency matrix **A** with 0.3. Then, we applied the clique percolation method (Palla et al., 2005) to this network to investigate the local densities of connectivity in this network using the networkX Python module. In graph theory, K-clique implies the fully connected subgraph consisting of K nodes. In the clique percolation method, first, K-cliques are identified. Then, pairs of K-cliques are connected to form a cluster if they share a (K-1)-clique. Furthermore, if the cluster shares a (K-1)-clique with another K-clique, it is assimilated to the cluster. This process is iteratively executed. When we set K to a large value, the resulting cluster becomes a densely connected subgraph.

### Community Analysis on the Parcel-to-Parcel RSFC Matrix

By shifting and scaling the values in the parcel-to-parcel RSFC matrix, we first obtained the parcel-to-parcel similarity matrix in which the minimum and maximum values were 0 and 1, respectively. To identify the community structure in the parcels based on the similarity matrix, we applied spectral clustering to the parcel-to-parcel similarity matrix using the scikit-learn module in Python. As is the case with the clustering of cognitive functions, we used the k-means method to identify data clusters in the representational space identified with spectral embedding. The number of communities was set at the value maximizing the silhouette coefficients (Rousseeuw, 1987) up to 20 (Supplementary Figure S3). The other parameters were set to the default values.

### Reliability Check of RSFC Matrices

Since the analyses described in the above subsections were basically based on the RSFC matrices defined as the correlation matrices, checking the reliabilities of the estimations is worthful to evaluate the stabilities of the results. Especially, we should be careful about the possible instabilities that might be caused by the smaller data size compared to the data stored in the recently developing large-scale databases such as the Human Connectome Project database (Smith et al., 2013; Van Essen et al., 2013). To this end, we calculated the standard errors of means (SEMs) of the RSFCs. Accordingly, we observed the small levels of the values (∼0.035) compared to the absolute RSFC values (Supplementary Figure S4), which means that the effects of the instabilities caused by the small data size were substantially limited.

We also conducted a correlation analysis between the RSFCs estimated from the present data and the Human Connectome Project data (Supplementary Figure S5). We used only 706 of about 2000 data in S500 dataset because of resource limitation. The preprocessing pipeline was the same as the one explained above. The correlation coefficients are acceptable (0.94 for CFM-to-CFM RSFCs, 0.84 for voxel-to-CFM RSFCs, and 0.58 for parcel-to-parcel RSFCs). Again, those results suggest that the small size of the present data affected the results limitedly.

## Results

### Relational Mapping for Cognitive Functions

**FIGURE 1:**
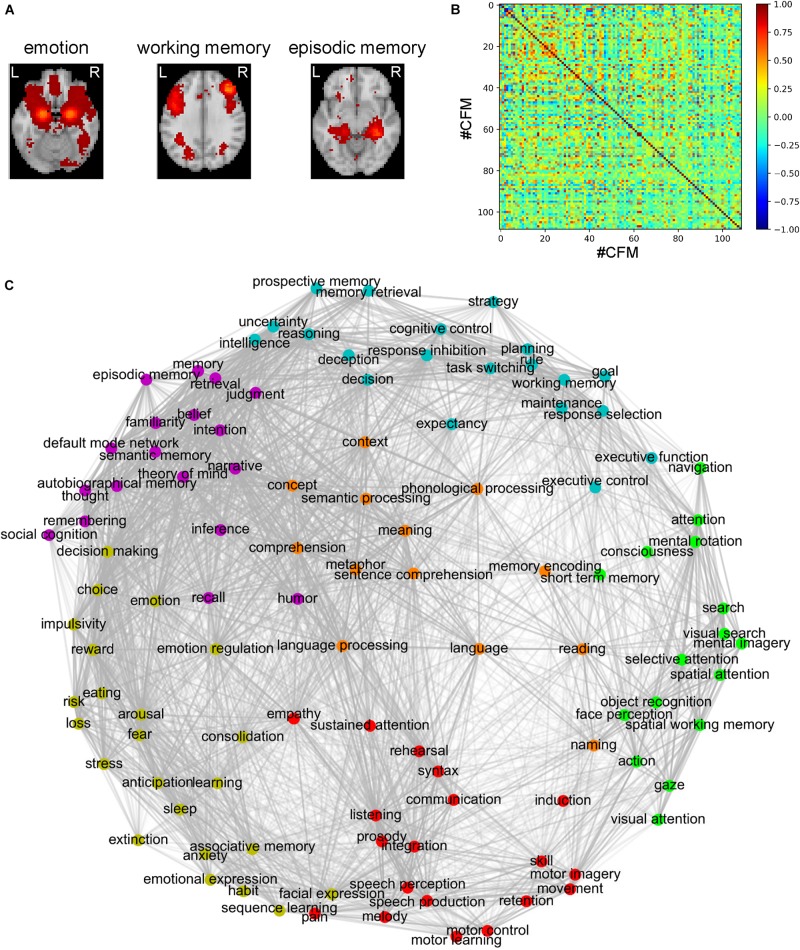
Relational mapping and clustering of cognitive functions based on the cognitive function map (CFM)-to-CFM resting-state functional connectivities (RSFCs). **(A)** Examples of the reconstructed CFMs. **(B)** The CFM-to-CFM RSFC matrix. **(C)** Relational mapping of cognitive functions. The locations of cognitive functions in the two-dimensional plane were determined by applying multidimensional scaling to the CFM-to-CFM RSFC matrix. Furthermore, spectral clustering was applied and resulted in six clusters of cognitive functions, in which, roughly, the red-purple cluster included ‘self and others’-related functions, the blue cluster included ‘executive function’-related functions, the orange cluster included ‘language’-related functions, the yellowish-green cluster included ‘value and judgment’-related functions, the red cluster included ‘action and expression’-related functions, and the green cluster included ‘vision and attention’-related functions.

In the present study, we aimed to elucidate the relationships among the cognitive functions in the human brain. To obtain a comprehensive overview of the human cognition, a visualization of the whole picture representing the relationships among cognitive functions is required. To this end, we began our analysis with the 109 CFMs which were reconstructed as pseudo-activation maps corresponding to 109 cognitive functions (Figure 1A). By applying multidimensional scaling to the CFM-to-CFM RSFC matrix (Figure 1B and Supplementary Data S1), we provided a relational mapping that involved two-dimensional embedding of the cognitive functions, in which the closely related cognitive functions were located close to each other (Figure 1C). In addition, we identified six clusters of cognitive functions (cognitive clusters) using the spectral clustering method with the silhouette coefficients (Figure 1C and Table 1). Roughly, the red-purple cluster included ‘self and others’-related functions, the blue cluster included ‘executive function’-related functions, the orange cluster included ‘language’-related functions, the yellowish-green cluster included ‘value and judgment’-related functions, the red cluster included ‘action and expression’-related functions, and the green cluster included ‘vision and attention’-related functions.

**TABLE 1:** Clustering of the cognitive functions based on the CFM-to-CFM RSFC matrix.

**Action and expression**	**Vision and attention**	**Value and judgment**	**Self and others**	**Executive function**	**Language**
Movement	Attention	Emotion	Memory	Working memory	Language
Pain	Action	Reward	Retrieval	Decision	Reading
Integration	Gaze	Learning	Judgment	Cognitive control	Context
Skill	Spatial attention	Risk	Intention	Response inhibition	Meaning
Empathy	Selective attention	Fear	Recall	Goal	Comprehension
Listening	Search	Anxiety	Episodic memory	Rule	Concept
Motor imagery	Navigation	Decision-making	Default mode network	Reasoning	Naming
Prosody	Short-term memory	Stress	Familiarity	Maintenance	Semantic processing
Speech perception	Mental rotation	Loss	Social cognition	Planning	Metaphor
Communication	Consciousness	Choice	Inference	Executive function	Memory encoding
Sustained attention	Spatial working memory	Anticipation	Belief	Uncertainty	Language processing
Motor control	Visual search	Sleep	Thought	Deception	Phonological processing
Retention	Visual attention	Facial expression	Theory of mind	Task switching	Sentence comprehension
Rehearsal	Mental imagery	Arousal	Semantic memory	Strategy	
Syntax	Face perception	Emotion regulation	Narrative	Response selection	
Induction	Object recognition	Extinction	Autobiographical memory	Executive control	
Speech production		Impulsivity	Humor	Intelligence	
Motor learning		Habit	Remembering	Memory retrieval	
Melody		Eating Consolidation Sequence learning Associative memory Emotional expression		Expectancy Prospective memory	

*CFM, cognitive function maps; RSFC, resting-state functional connectivity.*

To check a distortion caused by the embedding, for each embedding dimension up to ten, we calculated the stress that is an index quantifying the deviation of distances in the embedding space from the distances defined based on the similarity matrix. The decline of stress is shown as the scree plot in Supplementary Figure S6. According to the scree criterion, an optimal dimension seems to be four. Although the two-dimensional mapping has good readability, this means that it was somewhat distorted and could not exactly express the strengths of the RSFCs between the CFMs. Therefore, we also provide figures that are similar to Figure 1C but show the positive and negative strengths of RSFCs using red and blue colors, respectively (Supplementary Figure S7).

### RSFC-Based Conceptual Analysis of Cognitive Functions

**FIGURE 2:**
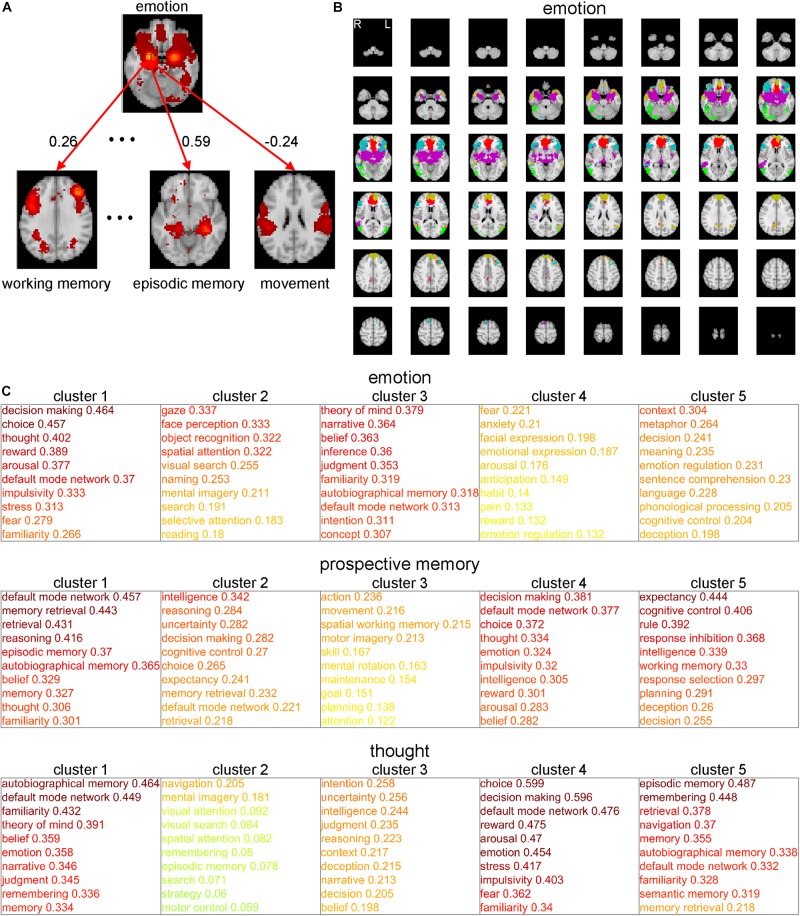
Conceptual analysis of cognitive functions based on subdivisions of the cognitive function maps (CFMs). **(A)** Schematic illustration of voxel-to-CFM resting-state functional connectivities (RSFCs) between voxels in a CFM of cognitive function under consideration (e.g., ‘emotion’) and the other CFMs. **(B)** Example of subdivision of the CFM of ‘emotion’ obtained by applying k-means clustering to the CFM. In this analysis, the number of clusters was fixed to five. Each color corresponds to each cluster resulting from the subdivision. **(C)** Examples of subdivision-based conceptual analyses for ‘emotion’ (upper), ‘prospective memory’ (middle), and ‘thought’ (lower). The cluster-to-CFM RSFCs are shown with the names of corresponding cognitive functions. For each cluster, the cluster-to-CFM RSFCs are defined as the mean values of the voxel-to-CFM RSFCs for the voxels belonging to the cluster.

One of the bottlenecks preventing us from understanding information processing during cognitive functions is that we do not have sufficient in-depth knowledge of the concepts of these cognitive functions. Therefore, we require so-called conceptual analysis of the cognitive functions (based not on philosophical deliberation but on neuroscientific evidence) to elucidate their deeper meanings. Here, we propose a method of conceptual analysis based on the voxel-to-CFM RSFCs (Figure 2). In this method, first, we selected a cognitive function (e.g., ‘emotion’) and the corresponding CFM. For all voxels within the selected CFM and the 108 remaining CFMs, a voxel-to-CFM RSFC matrix was constructed (Figure 2A). Then, we applied k-means clustering to the matrix and subdivided the CFM for the cognitive function into five clusters (Figure 2B). Finally, each cluster was related to the 108 remaining cognitive functions based on cluster-to-CFM RSFCs.

As examples, the results for ‘emotion,’ ‘prospective memory,’ and ‘thought’ are shown in Figure 2C. The subdivision of the ‘emotion’-corresponding CFM suggests that ‘emotion’ is constructed of the subfunctions related to decision-making (cluster 1), vision (cluster 2), self and others’ minds (cluster 3), fear (cluster 4), and comprehension of abstract meanings (cluster 5). The subdivision of the ‘prospective memory’-corresponding CFM suggests that ‘prospective memory’ is constructed of the subfunctions related to memory (cluster 1), intelligent decision (cluster 2), motion (cluster 3), emotional decision (cluster 4), and executive function (cluster 5). The subdivision of the ‘thought’-corresponding CFM suggests that ‘thought’ is constructed of the subfunctions related to self and others’ minds (cluster 1), imaginary navigation (cluster 2), logical intention and intelligence (cluster 3), emotional decision (cluster 4), and memory (cluster 5).

The results for all cognitive functions are provided in Supplementary Data S2. In this analysis, we set the numbers of clusters identical (i.e., five) across all CFMs by considering interpretability. On the other hand, showing results from the clustering in which the numbers of clusters were determined based on the silhouette coefficients are beneficial. Therefore, we provide these results in which the numbers of clusters were determined based on the silhouette coefficients (up to twelve clusters) in Supplementary Data S3.

The nifti-formatted images of the subdivided CFMs will be downloadable from the authors’ web page.

### Cognitive Function-Based Whole-Brain Parcellation

**FIGURE 3:**
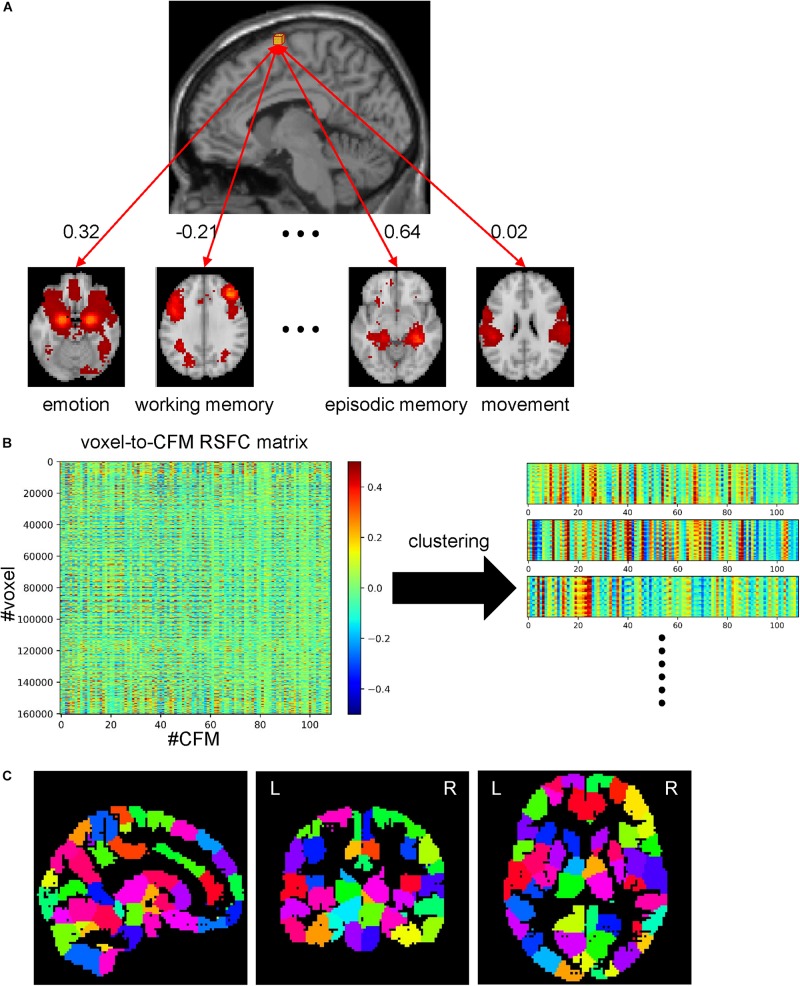
Whole-brain parcellation based on cognitive function maps (CFMs). **(A)** Schematic illustration of voxel-to-CFM resting-state functional connectivities (RSFCs). A correlation coefficient between resting-state activities of each voxel and CFM was calculated, and was defined as the RSFC between them. **(B)** Parcellation was obtained by applying spectral clustering to the whole-brain voxel-to-CFM RSFC matrix. Each panel on the right corresponds to each resulting parcel. **(C)** The resulting parcellation consisting of 199 parcels.

**FIGURE 4:**
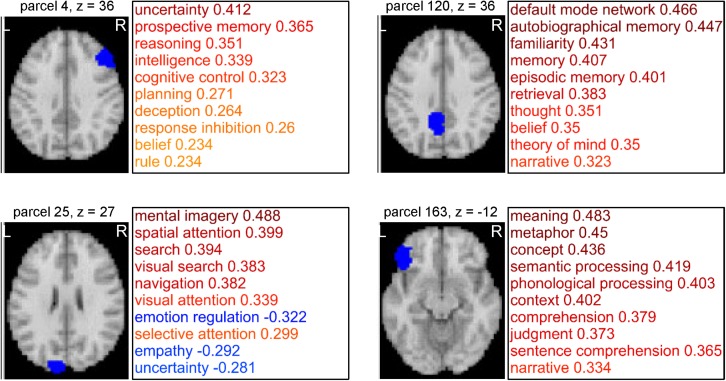
Examples of parcels resulting from cognitive function-based whole-brain parcellation. Four examples of the parcels. Maps on a standard brain (left in each parcel) and top 10 relatedness with cognitive functions (right in each panel) are shown. The relatedness was defined as mean voxel-to-cognitive function map resting-state functional connectivities over the voxels belonging to the parcel.

Network analyses using brain parcels that are associated with cognitive functions as network nodes are promising to offer insights into the characteristics of each function *per se* and the relationships among those functions. To construct such parcels, we administered a novel whole-brain parcellation method in which voxels were assembled to one of 199 clusters (or parcels) by applying spectral clustering to the voxel-to-CFM RSFC matrix (Figures 3A–C). Each resulting parcel was characterized by its relatedness with the 109 cognitive functions (i.e., parcel-to-CFM RSFCs), defined as mean voxel-to-CFM RSFCs over the voxels belonging to the parcel (Figure 4 and Supplementary Table S4). Their links to the anatomical brain regions are provided in Supplementary Table S5.

We also show the correspondence between the present parcellation and the Glasser’s atlas (Glasser et al., 2016) in Supplementary Table S6. We found that the voxels belonging to one parcel in the present parcellation are assigned to several parcels in the Glasser’s atlas. This is natural since the number of parcels in the Glasser’s atlas is larger than ours. We show the ratios of voxels assigned to the most overlapping region, the second most overlapping region, the third most overlapping region,…in Supplementary Figure S8. Thirty six percent of the voxels are included in the most overlapping regions in the Glasser’s atlas. Up to the fourth most overlapping regions, 84% of the voxels are included in them.

The nifti-formatted CFM and parcellation images will be downloadable from the authors’ web page.

### Cognitive Factor Identification Based on Dimensionality Reduction Using Non-negative Matrix Factorization

**FIGURE 5:**
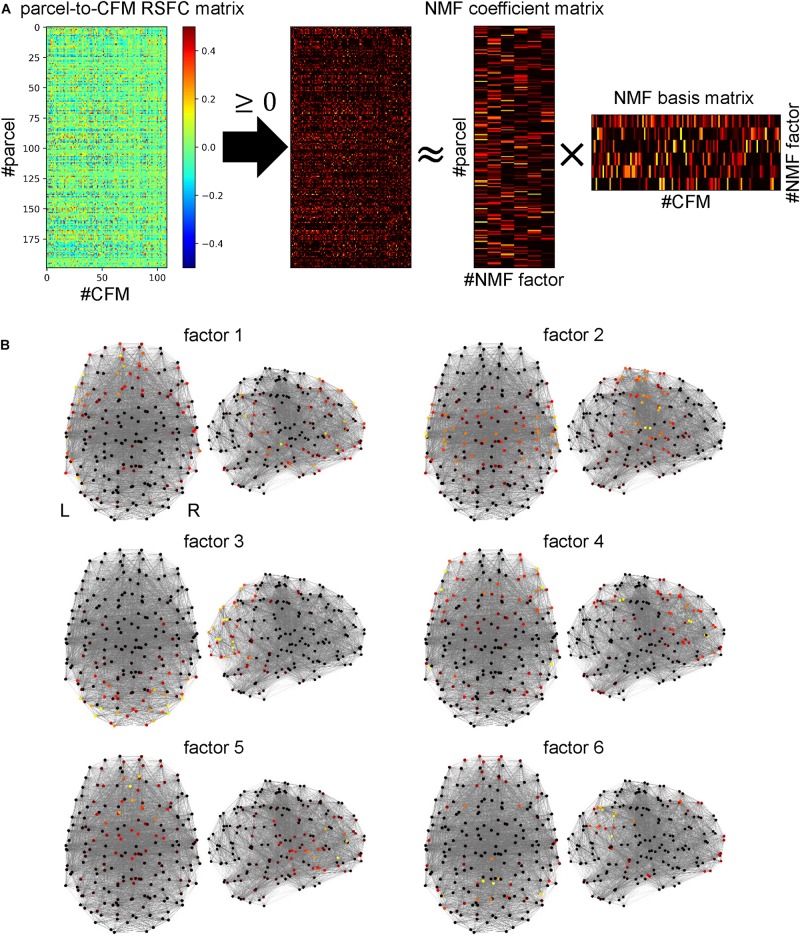
Identifying cognitive factors using non-negative matrix factorization (NMF). **(A)** Procedure for identification of cognitive factors. First, the parcel-to-cognitive function map (CFM) resting-state functional connectivity matrix was thresholded with zero. Then, NMF was applied to the thresholded matrix, and the NMF coefficient and basis matrices were identified. The rows and columns of the NMF coefficient matrix correspond to parcels and NMF factors (cognitive factors), respectively. The rows and columns of the NMF basis matrix correspond to factors and CFMs, respectively. **(B)** The NMF coefficient values for each NMF factor are mapped on the parcels (shown as dots) located according to coordinates on a standard brain using heat mapping.

The 109 cognitive functions were not independent of each other. Some functions were highly interrelated, and therefore, had common latent cognitive factors. We believe that all cognitive functions can be characterized by combinations of a few latent cognitive factors. When a group of cognitive functions is commonly dependent on such factors, the parcel-to-CFM RSFCs of members of the group should be similar. Thus, to identify the latent cognitive factors, we applied non-negative matrix factorization (NMF) to the parcel-to-CFM RSFC matrix (Figure 5A).

The number of NMF factors was determined to be six according to the evaluation of the residual sum of squares. The top ten components for each basis vector (row vector in the identified NMF basis matrix) with the corresponding cognitive functions are provided in Table 2. All components in the bases are shown in Supplementary Table S7. The NMF coefficient matrix is shown in Supplementary Table S8. We found that these cognitive factors roughly corresponded to ‘concept processing’ (factor 1), ‘action and expression’ (factor 2), ‘vision and attention’ (factor 3), ‘executive function’ (factor 4), ‘value and judgment’ (factor 5), and ‘memory’ (factor 6).

**TABLE 2:** Cognitive factors defined using non-negative matrix factorization of the parcel-to-CFM RSFC matrix.

**Factor 1 (concept processing)**		**Factor 2 (action and expression)**		**Factor 3 (vision and attention)**	
Comprehension	0.248	Movement	0.329	Mental imagery	0.342
Narrative	0.245	Motor imagery	0.314	Spatial attention	0.333
Concept	0.244	Speech production	0.302	Visual search	0.329
Judgment	0.227	Skill	0.283	Search	0.317
Metaphor	0.221	Speech perception	0.256	Object recognition	0.252
Theory of mind	0.211	Motor control	0.245	Attention	0.242
Inference	0.204	Melody	0.235	Gaze	0.241
Belief	0.204	Integration	0.226	Face perception	0.223
Intention	0.202	Prosody	0.213	Selective attention	0.218
Semantic processing	0.191	Listening	0.207	Navigation	0.204

**Factor 4 (executive function)**		**Factor 5 (value and judgment)**		**Factor 6 (memory)**	

Cognitive control	0.303	Reward	0.320	Episodic memory	0.342
Rule	0.291	Anticipation	0.270	Default mode network	0.302
Working memory	0.289	Fear	0.263	Memory	0.293
Planning	0.288	Arousal	0.261	Autobiographical memory	0.278
Maintenance	0.276	Choice	0.255	Memory retrieval	0.266
Response inhibition	0.241	Decision making	0.233	Remembering	0.264
Expectancy	0.224	Loss	0.229	Retrieval	0.264
Task switching	0.216	Risk	0.225	Thought	0.262
Decision	0.210	Stress	0.224	Familiarity	0.254
Deception	0.198	Eating	0.202	Prospective memory	0.195

*For each factor, the cognitive functions having the ten largest NMF basis values are shown with the corresponding NMF basis values. CFM, cognitive function map; RSFC, resting-state functional connectivity; NMF, non-negative matrix factorization.*

For each factor, the heat map of the NMF coefficients for the corresponding parcels are shown in Figure 5B, in which we observe factor-specific spreading patterns. The factor 1-related parcels are located on the left inferior parietal cortex, left superior and middle temporal cortex, left inferior frontal gyrus, and the left superior frontal cortex. The factor 2-related parcels are located on the bilateral sensorimotor areas and the superior temporal cortices. The factor 3-related parcels are located on the bilateral occipital cortices. The factor 4-related parcels are located on the bilateral lateral prefrontal cortices and supramarginal gyri. The factor 5-related parcels are located on the bilateral medial prefrontal cortices. The factor 6-related parcels are located on the bilateral precuneus areas and the inferior parietal cortices.

### Diversity of Information Sources/Receivers Is Dependent on Cognitive Factors

Some cognitive functions may need various kinds of information to be realized while others may require only limited kinds of information. Similarly, information derived from some cognitive functions may be required to realize various kinds of cognitive functions while other information may be needed only from a small number of cognitive functions. We considered the diversity of informational interactions to be dependent on cognitive factors. Therefore, we quantified the diversity of information sources or receivers that were collected by parcels in the parcel network. To clarify our method, we assumptively describe some parcels, functions, or factors as *sources* in the present section. However, we note that these may be *receivers* because our method did not identify the directions of informational interactions.

**FIGURE 6:**
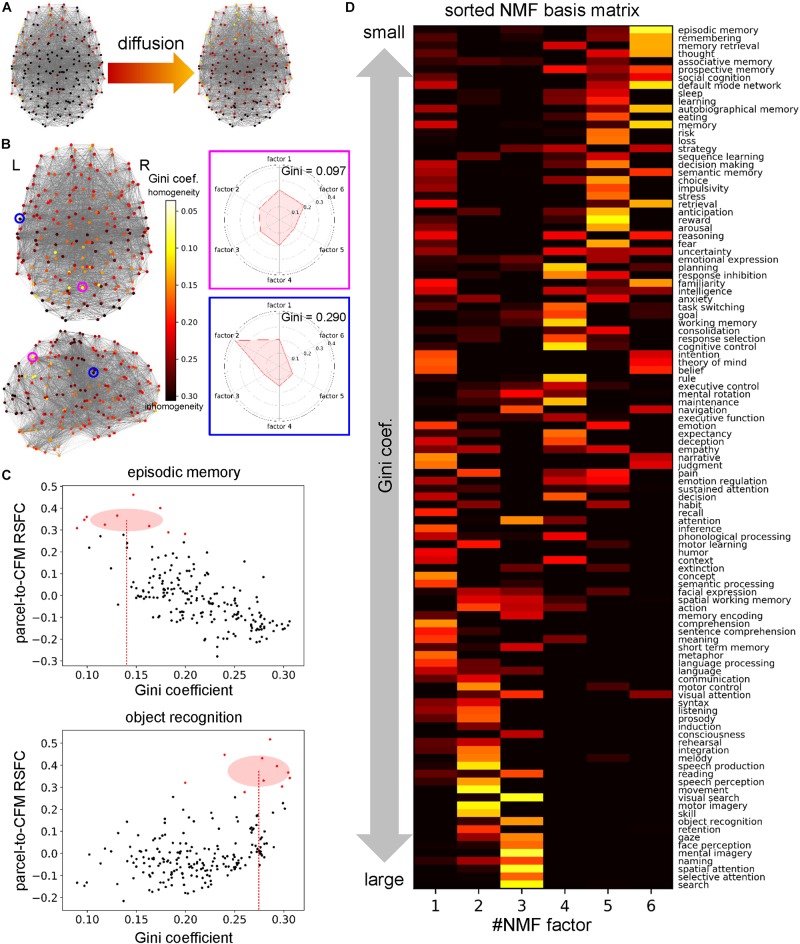
Cognitive factor-dependent diversity of informational interactions. **(A)** Heat source (left) and temperature (right) distributions on the parcels for the cognitive factor 1. The heat source values were defined as the non-negative matrix factorization (NMF) coefficient values of the corresponding column. Temperatures were calculated at steady states of the diffusion process governed by the graph Laplacian. **(B)** The Gini coefficient distribution. For each parcel, the Gini coefficient represents inhomogeneity of temperatures across the factors. The Gini coefficients and polar graph of temperature for the parcel circled with magenta and blue are shown on the right. **(C)** Example plots between the Gini coefficient values and parcel-to-cognitive function map (CFM) resting-state functional connectivities (RSFCs). Upper and lower plots correspond to the CFMs of ‘episodic memory’ and ‘object recognition,’ respectively. Each dot expresses each parcel. The parcels that have the 10 largest RSFCs are red-colored. The means and standard deviations of the Gini coefficients for these red-colored parcels are shown as the centers and radiuses of the red circles, respectively. The means are also indicated by the red dotted line. **(D)** The transposed NMF basis matrix, sorted by the Gini coefficients. The cognitive function corresponding to each CFM is shown on the right.

First, for each parcel, we defined information sent from a cognitive factor as steady pseudo-temperature calculated from the heat diffusion equation in the network with heat sources whose intensities were defined by the NMF coefficient vector (column vector of the NMF coefficient matrix) (Figure 6A). This resulted in a temperature distribution over the parcel network for each cognitive factor. We observed that the temperatures of some parcels were roughly uniform across all cognitive functions, which implies equal collection of information. On the other hand, in another parcel, only one factor provided a high temperature and the other factors provided low temperatures, which implies a polarized collection of information. To quantify the degree of polarization, we used the Gini coefficients of the distributions of temperatures across cognitive factors (Figure 6B). Smaller Gini coefficients express more uniformity over cognitive factors, suggesting more diverse information sources/receivers.

Moreover, we investigated the 109 cognitive functions in terms of the diversity of information sources/receivers. For each cognitive function (or CFM), we averaged the Gini coefficients of the parcels whose parcel-to-CFM RSFCs were among the top ten (Figure 6C). The resulting value was regarded as the Gini coefficient for the corresponding cognitive function. Upon sorting the NMF bases by the Gini coefficients of the cognitive functions, we observed cognitive factor-dependent differences in the diversity of information sources/receivers (Figure 6D and Supplementary Table S9). The factor 6-related cognitive functions tended to collect information from the most diverse sources/receivers. The factor 5- and 4-related functions had the second- and third-most diverse information sources/receivers, respectively. The diversity of information sources/receivers for the factor 1-related functions was moderate. The factor 2- and 3-related functions collected information from the most polarized sources/receivers.

**FIGURE 7:**
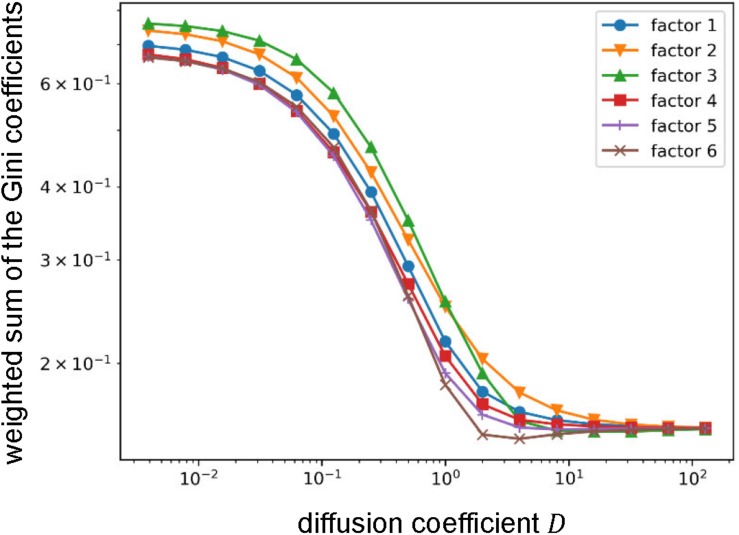
Effect of the value of the diffusion coefficient on the weighted sum of the Gini coefficient. Log-log plots of the weighted sums of Gini coefficients with varying the diffusion coefficient. Throughout the parameter region, relative orders are qualitatively similar except for the factor 3. The weighted sum of Gini coefficients for factor 3 moves from largest (i.e., lowest diversity of informational interaction) to smallest (i.e., highest diversity of informational interaction).

The method used in this section has two parameters: diffusion coefficient *D* and decay constant β. Therefore, as an additional analysis, we investigated the effects of those parameter values. Since the result is only dependent on the ratio of those parameters, we only varied the diffusion coefficient*D*. As we observed, the Gini coefficients for the cognitive functions highly loaded by some factors were small and others were large. Thus, we compared the weighted sums of the Gini coefficients that express the means of the Gini coefficients for the cognitive functions assigned to the factors (see section Materials and Methods) between factors (Figure 7). Throughout the parameter region, we found qualitatively similar results to the one shown above except for the factor 3 that relates to vision and attention. The value of the weighted sum of the Gini coefficients for the factor 3 was largest when the diffusion coefficient was small, which means that the diversity of information sources/receivers was lowest. However, the diversity (indexed with the weighted sum of the Gini coefficients) relative to the others increased with an increase in the diffusion coefficient, and, finally became highest. Since the diffusion coefficient decides the range of information transmission, this result suggests the factor 3 (relating vision and attention) changes the relative diversity of informational interactions depending on the state of information transmission.

### Cognitive Factor-Dependent Difference in Densities of Local Connectivity

**FIGURE 8:**
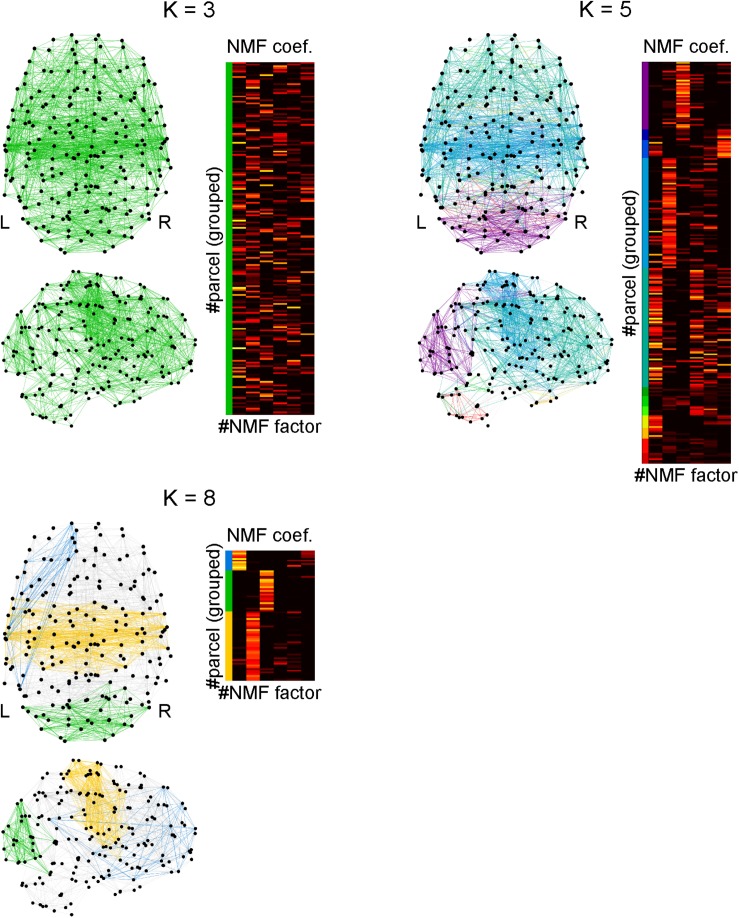
Identifying densely connected subnetworks using clique percolation method. Subnetworks are color-coded and the non-negative matrix factorization coefficients of the parcels belonging to the subnetworks are shown in corresponding colors. The clique threshold K is a criterion for densities of connectivity in subnetworks to be identified. The connectivity becomes dense with an increase in K. When *K* = 3, all parcels were interconnected, which implies only one network was identified. When *K* = 5, 11 subnetworks were identified. When *K* = 8, three subnetworks were identified, in each of which the parcels were densely interconnected.

The connection density of network which processes a cognitive function is an important factor to specify computational characteristics of the function. Using the clique percolation method, we identified local subnetworks within the parcels that were densely connected (Figure 8). By increasing the clique threshold K, subnetworks whose connectivity were denser came to the surface. When K was set to 8, we identified three densely connected subnetworks. By extracting the NMF coefficients for the parcels belonging to densely connected subnetworks, we found that these subnetworks were highly related with the factors 1 (blue), 2 (yellow), and 3 (green). The parcels composing each subnetwork are shown with the anatomical information in Supplementary Table S10.

The blue densely connected subnetwork includes the following regions: the left temporal cortex, left inferior parietal cortex, left supramarginal gyrus, left orbitofrontal cortex, left inferior frontal cortex (pars triangularis and pars orbitalis), left superior frontal cortex, left rostral middle frontal cortex, left anterior cingulate cortex, left frontal pole, and a small part of the left temporal pole.

The yellow densely connected subnetwork includes the following regions: the bilateral putamen, bilateral pallidum, bilateral caudal anterior cingulate cortices, bilateral posterior cingulate cortices, left middle temporal gyrus, bilateral superior temporal gyri, bilateral transverse temporal gyri, bilateral superior parietal cortices, bilateral supramarginal gyri, bilateral precuneus, bilateral precentral gyri, bilateral postcentral gyri, bilateral paracentral lobules, bilateral insula, bilateral pars opercularis (mainly left), and the bilateral superior frontal gyri (slightly lateralized to the left hemisphere).

The green densely connected subnetwork includes the following regions: the bilateral cerebellum, bilateral lateral occipital cortices, bilateral cuneus, bilateral pericalcarine cortices, bilateral lingual gyri, bilateral fusiform gyri, bilateral inferior parietal cortices, and the bilateral superior parietal cortices. Additionally, a small part of the inferior temporal cortex is included.

### Network Communities That Are Uniformly or Diversely Associated With Cognitive Factors

**FIGURE 9:**
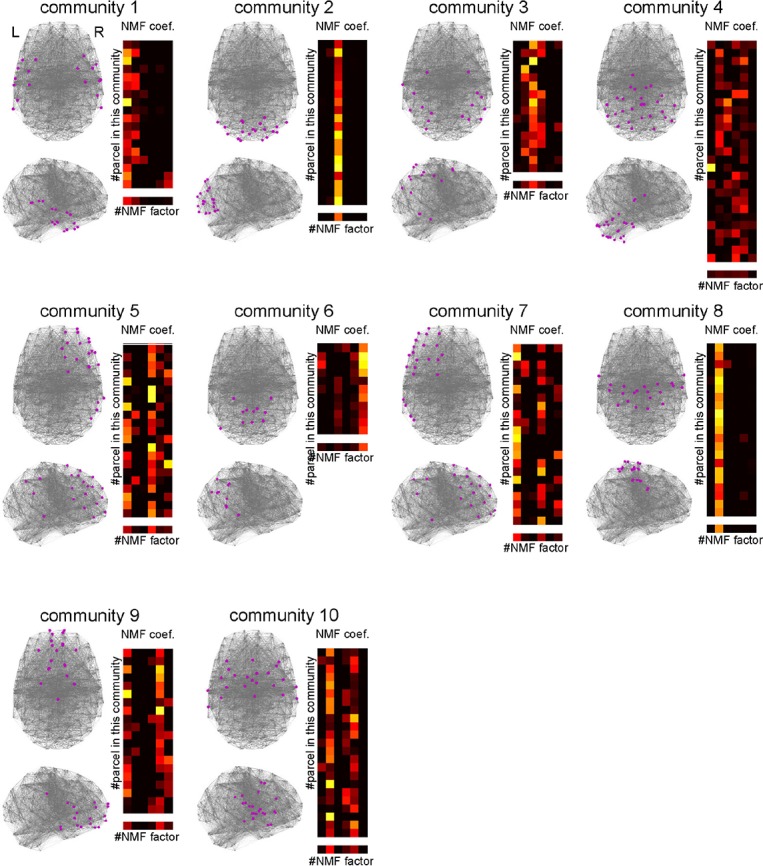
Characterizing communities in the whole-brain network with the cognitive factors. Ten network communities were identified using spectral clustering. The parcels belonging to each community are shown by magenta dots. For each community, the subpart of the non-negative matrix factorization coefficient matrix corresponding to the parcels in the community is shown on the right. The lowest row shows the row mean of the matrix.

Previous studies suggest that the RSFC network has a modular or community structure (He et al., 2009; Power et al., 2011; Bertolero et al., 2015, 2018). Such a community is considered as a module of information processing. To elucidate the information processing executed in each community, it is important to reveal whether the community is related to uniform or diverse kinds of cognitive functions. To this end, we identified the community structure by applying spectral clustering to the parcel-to-parcel RSFC matrix and investigated the functional uniformity or diversity of each community (Figure 9). The number of communities was set to 10, which maximized the silhouette coefficients. The NMF coefficients for the parcels belonging to the identified communities showed uniformity and diversity in their association with the cognitive factors in a community-dependent manner. The communities 2 and 8 specifically associated with cognitive factors 3 and 2, respectively. Conversely, the community 4, which was mainly located in the cerebellum, associated with diverse cognitive factors.

## Discussion

In the present study, we endeavored to show a whole picture of the human cognition and to reveal characteristics of each cognitive function that constitutes it. To this end, we investigated the relationships among 109 cognitive functions based on two ideas: (1) the cognitive functions that overlapping brain regions are responsible for should be interrelated, and (2) the cognitive functions that connected brain regions are responsible for should be also interrelated. Especially, we characterized 109 cognitive functions based on the CFM and RSFC-determined relationships among them. First, we presented a relational mapping that involved two-dimensional embedding of the cognitive functions using the RSFCs among CFMs. Then, we performed conceptual analysis in which a cognitive function was analyzed to identify the subfunctions constituting it, based on the RSFCs between voxels in the targeted CFM and the remaining CFMs. Moreover, we obtained a novel whole-brain parcellation in which each parcel had the vector of relatedness with these cognitive functions. Based on the network analyses using the parcels, we identified six cognitive factors, quantified the diversity of information sources/receivers for each cognitive function and factor, found the densely connected subnetworks associated with specific cognitive factors, and identified the communities that were associated with uniform or diverse cognitive factors. Altogether, we suggest the effectiveness of our approach in which we combined a large-scale meta-analysis of functional brain mapping with the methods of network neuroscience to investigate the relationships among cognitive functions to understand each cognitive function *per se* and the human as a relational system consisting of cognitive functions.

### Implications of the Results and Comparisons With Previous Studies

Categorization of cognitive functions is an essential first step not only for the scientific understanding of the brain but also for the clinical application of neuroscientific knowledge for diagnosis of psychiatric diseases. In this study, we provided such categorizations using two methods. One was based on the clustering on the CFM-to-CFM network and also yielded six cognitive clusters, including ‘language,’ ‘action and expression,’ ‘vision and attention,’ ‘executive function,’ ‘value and judgment,’ and ‘self and others.’ The other was based on the NMF, and yielded six cognitive factors: ‘concept processing,’ ‘action and expression,’ ‘vision and attention,’ ‘executive function,’ ‘value and judgment,’ and ‘memory.’

**FIGURE 10:**
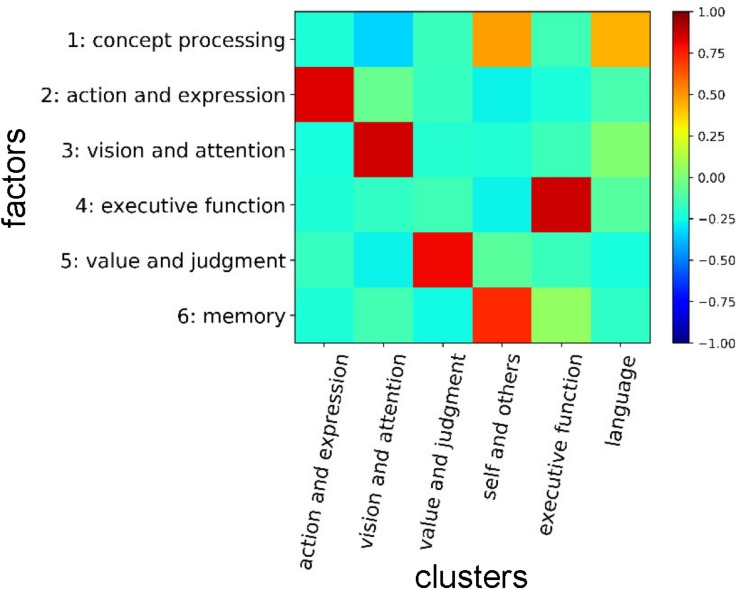
Correspondences between cognitive clusters and factors. Correlation coefficients between NMF basis vectors for the factors and presence/absence vectors of cognitive functions for the clusters are shown. In the presence/absence vectors, the presences and absences in the cluster were assigned to ‘1’ and ‘0’, respectively.

We show the entire correspondences between the cognitive clusters and the factors in Figure 10. The cognitive factors ‘action and expression,’ ‘vision and attention,’ ‘executive function,’ and ‘value and judgment’ roughly correspond to the cognitive clusters that are labeled with the same names. The ‘memory’ factor mainly relates to the ‘self and others’ cluster. Additionally, we like to stress that several functions that are strongly associated with the ‘memory’ factor (e.g., ‘memory retrieval’ and ‘prospective memory’) belong to the ‘executive function’ cluster. The ‘concept processing’ factor seems to relate to the cognitive clusters in a complex manner. Considering the NMF basis vector, it is suggested to be related to both ‘language’ and ‘self and others’ clusters. Therefore, the concepts of ‘memory,’ ‘concept processing,’ ‘executive function,’ ‘language,’ and ‘self and others’ are entangled, and the information processing relating these concepts may be executed through close interactions among them.

In the Diagnostic and Statistical Manual of Mental Disorders, Fifth Edition (DSM-5), which describes the current standardized criteria to diagnose psychiatric diseases, the neurocognitive domain is categorized into six subdomains consisting of ‘complex attention,’ ‘executive function,’ ‘learning and memory,’ ‘language,’ ‘perceptual-motor,’ and ‘social cognition’ (American Psychiatric Association, 2013). We found rough correspondences between the categorizations in DSM-5 and our results. The ‘complex attention’ subdomain in DSM-5 is considered to be included in the ‘vision and attention’ cognitive factor and cluster in the present study. The ‘executive function’ subdomain in DSM-5 probably corresponds to the cognitive factor and cluster labeled with the same name in this study. The ‘learning and memory’ subdomain in DSM-5 mainly relates to the ‘memory’ factor in this study. Since the immediate memory is included in the ‘learning and memory’ subdomain in DSM-5, this may relate to the ‘executive function’ cognitive factor and cluster in this study that involves ‘maintenance’ and ‘working memory.’ The ‘language’ subdomain in DSM-5 roughly corresponds to the ‘language’ cluster in our analysis. Furthermore, it also relates to the ‘action and expression’ cluster in this study because it includes ‘syntax,’ ‘listening,’ ‘communication,’ and so on. Additionally, the ‘language’ subdomain in DSM-5 probably has a close relationship with the ‘concept processing’ and ‘action and expression’ factors in this study. The ‘perceptual-motor’ subdomain in DSM-5 mainly relates to the ‘action and expression’ and ‘vision and attention’ factors and clusters in this study. The ‘social cognition’ subdomain in DSM-5 mainly relates to the ‘value and judgment’ and ‘self and others’ clusters in this study. It may also relate to the ‘concept processing’ and ‘value and judgment’ factors.

The relational mapping among cognitive functions that we obtained provides several insights into the mechanisms of cognition. We found that the default-mode network was located in a position close to ‘self and others’-related cognitive functions (e.g., ‘theory of mind’ and ‘autobiographical memory’) and social cognitive functions (e.g., ‘social cognition’ and ‘decision-making’). In fact, many studies suggest that these cognitive functions share underlying neural substrates (Spreng et al., 2009; Andrews-Hanna et al., 2010, 2014; Spreng and Grady, 2010; Mars et al., 2012; Reniers et al., 2012; Li et al., 2014; Meyer et al., 2019). We also found that ‘phonological processing’ was located close to the ‘executive function’ cluster. This seems to be consistent with Baddeley’s working memory system (Baddeley, 2000), in which phonological loop interacts with central execution. From the same point of view, we can link ‘episodic memory’ with episodic buffer in Baddeley’s system, since it was also located close to the ‘executive function’ cluster. More globally, we observed that the ‘executive function’ cluster neighbored the ‘self and others’ cluster, centering on the ‘default-mode network.’ Several studies reported cooperative activity between the brain areas related to these cognitive functions when subjects experienced spontaneous thoughts (Christoff et al., 2009) and engaged in creative tasks (Beaty et al., 2015) and mental simulations (Gerlach et al., 2011). Thus, our relational mapping of cognitive functions provides a whole picture of cognition which is feasible because it includes many known neurocognitive relationships. A study to survey relationships among cognitive functions whose aim was similar to ours was conducted using text analysis of neuroscience literature (Beam et al., 2014). In this study, the authors identified networks among 100 cognitive concepts, among 100 anatomical regions, and among combinations of both on the basis of the co-occurrences of the terms in the texts. More recently, a study reported the relations among 120 cognitive functions using hierarchical clustering based on correlations between pseudo-activation patterns, not RSFCs (Alexander-Bloch et al., 2018). Owing to methodological variations between the present and those studies, the present study can endow another picture complementing these studies.

In the present study, we proposed a novel method for conceptual analysis of cognitive concepts based on the CFMs and RSFCs in the brain. This yielded functional subdivisions of the cognitive concepts. Each sub-concept was characterized by its relatedness with the other cognitive concepts. We found several unexpectedly characterized sub-concepts. A sub-concept of ‘emotion’ that is characterized by functionality involving comprehension of abstract meanings is one such unexpected sub-concept. This may imply that we need emotional processing to receive an implicit message from linguistic expressions. Conversely, emotional processing may require analysis of abstract meanings. Further, we found that ‘thought’ had a sub-concept related to imaginary navigation. Navigation is considered to be handled by the grid and place cell systems. Several studies have shown that these systems play roles not only in physical spaces but also in abstract spaces such as social relationships, features of objects and events, and relational knowledge (Tavares et al., 2015; Constantinescu et al., 2016; Epstein et al., 2017; Garvert et al., 2017; Aronov et al., 2017; Schafer and Schiller, 2018). Therefore, imaginary navigation in an abstract space may be generally used in thoughts.

In the analysis for diversity of informational interactions, we observed that the nodes associated with cognitive functions that were closely related to the ‘memory’ factor interacted with the most diverse information. Since our analyses did not indicate the directions of the interactions, it was not clear whether these nodes were information sources or receivers. If the nodes play the role of information source, our result suggests that information processed with ‘memory’-related functions is necessary to realize a wide range of cognitive functions. Conversely, if the nodes are receivers of information, it suggests that execution of ‘memory’-related cognitive functions need information from a wide range of cognitive functions. Since the ‘value and judgment’- and ‘executive function’-related cognitive functions also have relatively diverse informational interactions, these results suggest similar implications. To support these results, an analysis to clarify the interaction directions will be required. In the additional analysis, the behavior of the factor relating to ‘vision and attention’ is insightful since it suggests that the diversity of informational interaction highly depends on the range of information transmission. Since the efficacy of information transmission changes depending on the brain state such as wakefulness and sleep (Massimini et al., 2005), our observation may suggest that the role of visual and attentional processing on the entire cognitive information processing changes when the brain state shifts.

We identified a densely connected subnetwork that was highly related to the ‘concept processing’ factor as well as the subnetworks related to the ‘action and expression’ and ‘vision and attention’ factors. The ‘concept processing’ subnetwork included a direct pathway between the Broca’s and Wernicke’s areas and an indirect pathway passing through the left inferior parietal cortex, which has been previously identified as constituents of the perisylvian language networks (Catani et al., 2005). Moreover, we detected participation of a wide range of structures in the left prefrontal cortex, including the lateral, medial, and orbital regions as well as the frontal pole in this subnetwork. Since these areas involve various aspects of higher-order cognition (Passingham and Wise, 2012; Fuster, 2015), this subnetwork suggests the existence of an integrated cognitive function that is highly dependent on language processing but is contributed also from functions beyond language processing.

Previous studies have shown that functional communities exist in the brain (He et al., 2009; Eickhoff et al., 2011; Power et al., 2011; Crossley et al., 2013; Bertolero et al., 2015, 2018). The studies have emphasized functional specificities of the communities. On the other hand, we found differences in the degrees of functional specificities of the communities, in which some communities were specifically associated with one cognitive factor while other communities were associated with diverse cognitive factors. One of the communities associated with the most diverse cognitive factors was located mainly in the cerebellum. Although the cerebellum was previously considered to be related to motor functions, it is now recognized that the cerebellum involves a remarkably wide range of cognitive functions (Stoodley and Schmahmann, 2009; Strick et al., 2009; Stoodley, 2012), which is consistent with our results. Viewing the internal models in the cerebellum (Wolpert et al., 1998) as a general controller working on various mental activities may give rise to a theoretical foundation for the diversity of cerebellar functionality (Ito, 2008). Additionally, a theoretical study (Yamazaki and Tanaka, 2007) suggests that the cerebellum is considered a kind of universal machine, the so-called liquid state machine (Maass et al., 2002), which may also support our finding.

### Limitations and Future Directions

There are several limitations to the present study which should be addressed in future studies. While constructing the CFMs, we used abstract texts to count the occurrences of cognitive terms. We did not utilize contextual information. Therefore, we did not discriminate as to whether the occurrences meant activation or deactivation. Additionally, to ensure that a term was the main topic in a study, we only used the frequency of the occurrences in its title, abstract, and keywords. Utilization of contextual information is a promising way to improve our analyses. The methods being developed in the field of natural language processing will probably provide such ways. Additionally, the use of natural language processing technics can provide us useful data revealing the constraints of inferring relationships among cognitive functions.

Compared to the datasets stored in the recently developing large-scale databases such as the Human Connectome Project database (Smith et al., 2013; Van Essen et al., 2013), the dataset used in the present study was small with respect to both the number of subjects and the number of scan volumes. Although we checked the reliabilities of the RSFC matrices and we consider that the outlines of the results are validated, especially in details of the results, some instabilities caused by the small data size were probably not removed. Therefore, we should continuously revise and establish knowledge suggested from our observations.

The number of parcels in the cognitive function-based whole-brain parcellation was determined not based on data but by reference to several existing atlases (Destrieux et al., 2010; Power et al., 2011; Shen et al., 2013; Baldassano et al., 2015; Fan et al., 2016). The selection of the number is a trade-off problem. The larger number of parcels results in a set of smaller parcels. This is suitable to reflect spatial heterogeneity in the brain. On the other hand, since the BOLD signal of the parcel is calculated by averaging the signals over the voxels within it, the signal of a smaller parcel tends to be more fluctuated. Several studies suggest that estimations of network characteristics in the brain depend on a resolution of parcellation (de Reus and van den Heuvel, 2013; Proix et al., 2016). Therefore, we need to address the issue of the number of parcels in the future study.

The connectivity measures used in this study were undirected and did not provide any information regarding dynamic causality and logical orders. On the other hand, we expect that the identification of directions in connectivity will provide useful insights into the issues dealt with in the present study. One representative instance is the analysis of the diversity of informational interactions, in which we found that cognitive functions highly related to the ‘memory’ factor interact with the most diverse kinds of information. If those cognitive functions are information sources (or receivers), this result suggests certain roles (or mechanisms) of memory-related information processing in the entire cognitive information processing. Similarly, directional information is required in the RSFC-based conceptual analysis, in which we observed that ‘emotion’ should implicate a sub-concept related to comprehension of abstract meanings. To determine whether emotional processing contributes to comprehension or vice versa, we need to identify the direction of connectivity between the CFM corresponding to ‘emotion’ and the CFMs of the cognitive functions related to comprehension of abstract meanings. Another instance in which directional information is required is the relational mapping of cognitive functions. This is expected to reveal the hierarchical dependencies among the cognitive functions, which will provide a more sophisticated perspective for the mechanism of the entire human cognition. To these ends, we may use methods of time series analyses, including the dynamic causal modeling (Friston et al., 2003, 2014), Granger causality (Roebroeck et al., 2005; Seth, 2010), and transfer entropy (Schreiber, 2000; Vicente et al., 2011).

In our relational mapping, we used multidimensional scaling to embed cognitive functions. Although this method provided an easily interpretable overview of the relationship among cognitive functions, the distances between them were more or less distorted. Therefore, we need more sophisticated embedding methods. The t-SNE may be such a method (van der Maaten and Hinton, 2008; van der Maaten, 2014). Recently, embedding methods into non-Euclidean spaces, such as Poincaré embedding, has been proposed (Nickel and Kiela, 2017). Such a non-Euclidean embedding method is considered to reveal other types of information regarding the relationships among cognitive functions. In addition, on the basis of CFMs, RSFCs, and other useful neuroscientific tools, exploring ontological relations [e.g., *is-a* and *part-of* relationships (Lenartowicz et al., 2010; Hastings et al., 2014; Poldrack and Yarkoni, 2016)] is an important future direction.

The methods and results provided in the present study let us clarify the meaning of each cognitive concept and obtain an analytic and synthetic understanding of the relationships among cognitive concepts. This possibly provides an empirical sketch of the research domains of cognitive neuroscience, which has been the aim of neuroimaging studies involving meta-analytical methods (Alhazmi et al., 2018). Moreover, this will stimulate the research fields of biological brain- and/or cognition-inspired artificial intelligences (Anderson and Lebiereeds, 1998; Anderson et al., 2004; Anderson, 2005; Eliasmith et al., 2012; Hassabis et al., 2017) by providing guidelines for understanding human cognition as a whole.

## Data Availability Statement

Raw data cannot be shared publicly because the sharing of raw data was not ethically approved. Data without any personally identifiable information will be available from author HK’s web page.

## Ethics Statement

The studies involving human participants were reviewed and approved by the institutional ethics committee of the National Center of Neurology and Psychiatry (NCNP). The participants provided their written informed consent to participate in this study.

## Author Contributions

HK performed the experiments and data analysis. All authors contributed to the design of the study and writing of the manuscript.

## Conflict of Interest

The authors declare that the research was conducted in the absence of any commercial or financial relationships that could be construed as a potential conflict of interest.

## Footnotes

1 http://www.fmrib.ox.ac.uk/fsl/

2 https://surfer.nmr.mgh.harvard.edu/

3 https://github.com/neurosynth

4 https://www.ncbi.nlm.nih.gov/pubmed/

5 https://www.ncbi.nlm.nih.gov/books/NBK25501/
