# Supplementary material for: Revealing Relationships Among Cognitive Functions Using Functional Connectivity and a Large-Scale Meta-Analysis Database
Source: Front Hum Neurosci. 2020 Jan 10;13:457. doi: 10.3389/fnhum.2019.00457 (PMC6965330; doi:10.3389/fnhum.2019.00457)
Supplement: Supplementary file 14 [file Data_Sheet_1.PDF]

Supplementary Material Legends

Supplementary Figure 1: Silhouette coefficients to determine the number of clusters in clustering cognitive functions.

Supplementary Figure 2: Plot of the numbers of nonnegative matrix factorization (NMF) factors and the differences of residual sum of squares (RSSs). To detect the inflection point, linear regression for two line segments (green and red) was repeated with the inflection point separating them while calculating the sums of the squared errors. This figure shows the result when the sums of the squared errors are minimum.

Supplementary Figure 3: Silhouette coefficients to determine the number of communities of parcels.

Supplementary Figure 4: Standard errors of means (SEMs) of the RSFCs. The matrices (left) and the histograms (right) of the SEM values of the CFM-to-CFM RSFCs (A), the voxel-to-CFM RSFCs (B), and the parcel-to-parcel RSFCs (C).

Supplementary Figure 5: Correlation analysis between the present data and the Human Connectome Project (HCP) data. (A) The CFM-to-CFM RSFCs. (B) The voxel-to-CFM RSFCs. (C) The parcel-to-parcel RSFCs. Note that the p-values were estimated to 0 because of very large sample sizes.

Supplementary Figure 6: Scree plot of the stresses to the number of dimensions in the multidimensional scaling. The stress is defined as the difference between given dissimilarities and distances in the embedding space and declines with an increase in the number of dimensions. According to the scree criterion, an optimal dimension seems to be four.

Supplementary Figure 7: Relational mapping of cognitive functions, focusing on each cognitive function. In the panel for each cognitive function, only the connections linked to the cognitive function are shown. The positive and negative strengths are color-coded in red and blue, respectively.

Supplementary Figure 8: Ratios of the number of voxels assigned to the Glasser's parcels ordered by the amounts of overlap. First, the number of voxels in each intersection between the parcels of the present and Glasser's parcellation was counted as shown in Table S6. According to the numbers of voxels in the intersections, for each parcel in the present parcellation, the Glasser's parcels in the table were sorted. Thus, in the sorted table, the first, second, third, … rows corresponded to the most overlapping parcel, the second most overlapping parcel, the third most overlapping parcel, …, respectively. Then, we calculated row sum of the table followed by dividing the values by the total sum, resulting in the ratios of the number of voxels assigned to the Glasser's parcels ordered by the amounts of overlap (solid line). The cumulative ratios are shown using a dashed line.

Supplementary Table 1: All cognitive terms.

Supplementary Table 2: The 121 cognitive terms selected on the basis of specificity and appearances in articles.

Supplementary Table 3: The 109 cognitive terms selected on the basis of the χ^2 test. These terms were finally considered in the present study and used in the main analyses.

Supplementary Table 4: Parcel-to-cognitive function map (CFM) resting-state functional connectivities (RSFCs). The cognitive functions corresponding to the CFMs were sorted by the abstract values of the RSFCs shown on the right. Note that the RSFCs of parcel 30 are not available because this parcel is empty.

Supplementary Table 5: Anatomical information of the parcels. The number in each cell expresses the number of voxels belonging to the corresponding anatomical area and parcel. The empty parcel 30 is gray-colored.

Supplementary Table 6: Comparing between the presented parcellation and the Glasser's atlas. The number in each cell expresses the number of voxels belonging to the corresponding Glasser's and our parcels.

Supplementary Table 7: Cognitive factors. For each factor, the cognitive functions are sorted by and shown with the corresponding nonnegative matrix factorization (NMF) basis values.

Supplementary Table 8: Nonnegative matrix factorization (NMF) coefficient matrix.

Supplementary Table 9: Gini coefficients and the nonnegative matrix factorization (NMF) basis values for the cognitive functions. The table is sorted by the Gini coefficients.

Supplementary Table 10: Lists of parcels belonging to subnetworks resulting from clique percolation with anatomical information.

Supplementary Data 1: Cognitive function map (CFM)-to-CFM resting-state functional connectivity (RSFC) matrix.

Supplementary Data 2: Results of conceptual analyses for all cognitive functions based on subdivisions of the cognitive function maps (CFMs). The cluster-to-CFM resting-state functional connectivities (RSFCs) are shown with the names of the corresponding cognitive functions. The results corresponding to the cognitive functions are included in the following Excel worksheets (see the tabs below).

Supplementary Data 3: Results of conceptual analyses for all cognitive functions based on subdivisions of the cognitive function maps (CFMs), in which the numbers of clusters were determined from their silhouette coefficients. This is the same as Data S2 but the numbers of subdivisions are based on the silhouette coefficients. The cluster-to-CFM resting-state functional connectivities (RSFCs) are shown with the names of the corresponding cognitive functions. The results corresponding to the cognitive functions are included in the following Excel worksheets (see the tabs below).
