# Supplementary material for: Revealing Relationships Among Cognitive Functions Using Functional Connectivity and a Large-Scale Meta-Analysis Database
Source: Front Hum Neurosci. 2020 Jan 10;13:457. doi: 10.3389/fnhum.2019.00457 (PMC6965330; doi:10.3389/fnhum.2019.00457)
Supplement: Supplementary file 15 [file Image_1.PDF]

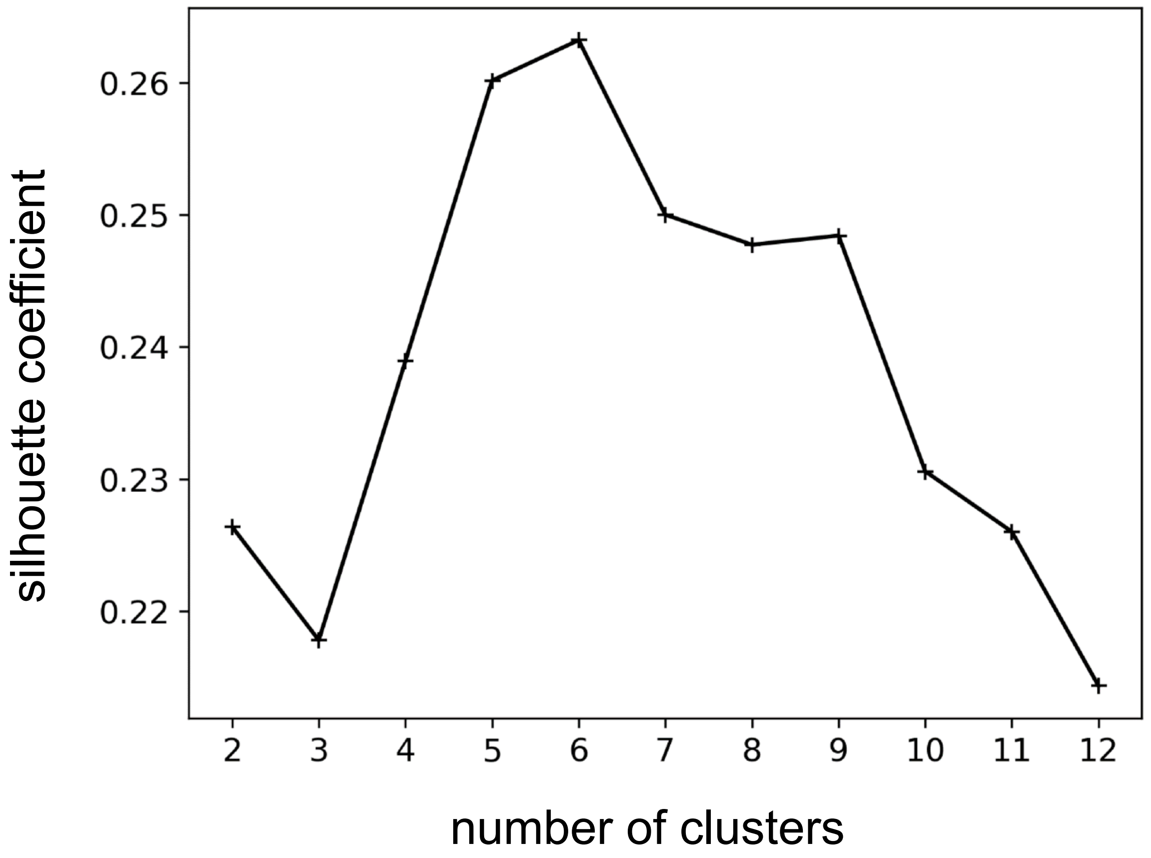

Supplementary Figure 1: Silhouette coefficients to determine the number of clusters in clustering cognitive functions.
