# Supplementary material for: Revealing Relationships Among Cognitive Functions Using Functional Connectivity and a Large-Scale Meta-Analysis Database
Source: Front Hum Neurosci. 2020 Jan 10;13:457. doi: 10.3389/fnhum.2019.00457 (PMC6965330; doi:10.3389/fnhum.2019.00457)
Supplement: Supplementary file 16 [file Image_2.PDF]

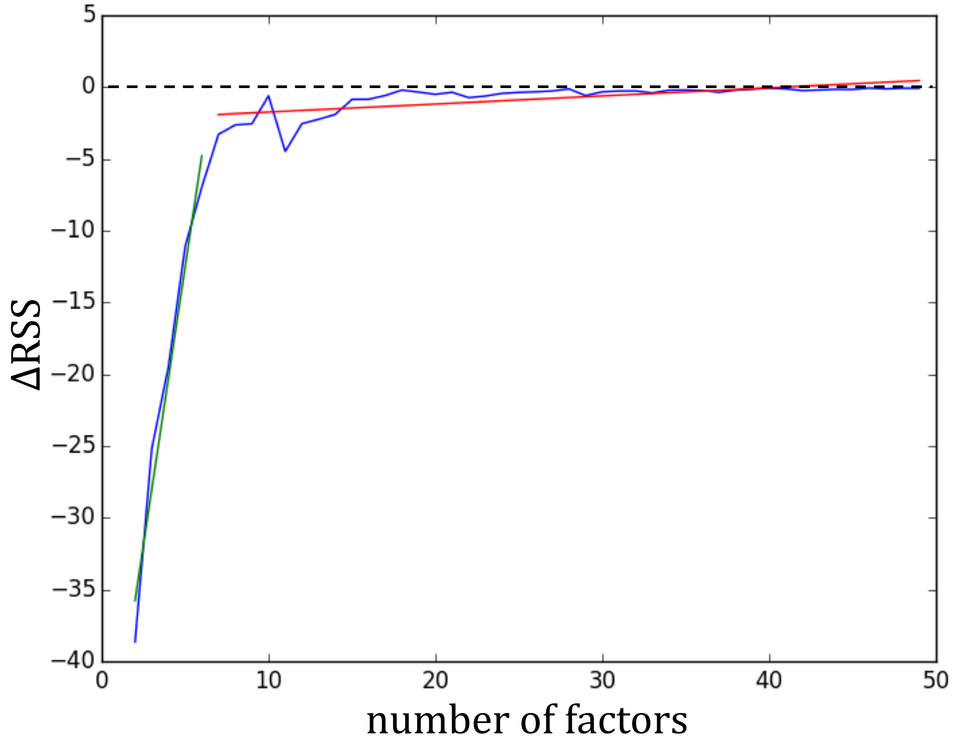

Supplementary Figure 2: Plot of the numbers of nonnegative matrix factorization (NMF) factors and the differences of residual sum of squares (RSSs). To detect the inflection point, lin-ear regression for two line segments (green and red) was repeated with the inflection point separat-ing them while calculating the sums of the squared errors. This figure shows the result when the sums of the squared errors are minimum.
