# Supplementary material for: Revealing Relationships Among Cognitive Functions Using Functional Connectivity and a Large-Scale Meta-Analysis Database
Source: Front Hum Neurosci. 2020 Jan 10;13:457. doi: 10.3389/fnhum.2019.00457 (PMC6965330; doi:10.3389/fnhum.2019.00457)
Supplement: Supplementary file 18 [file Image_4.PDF]

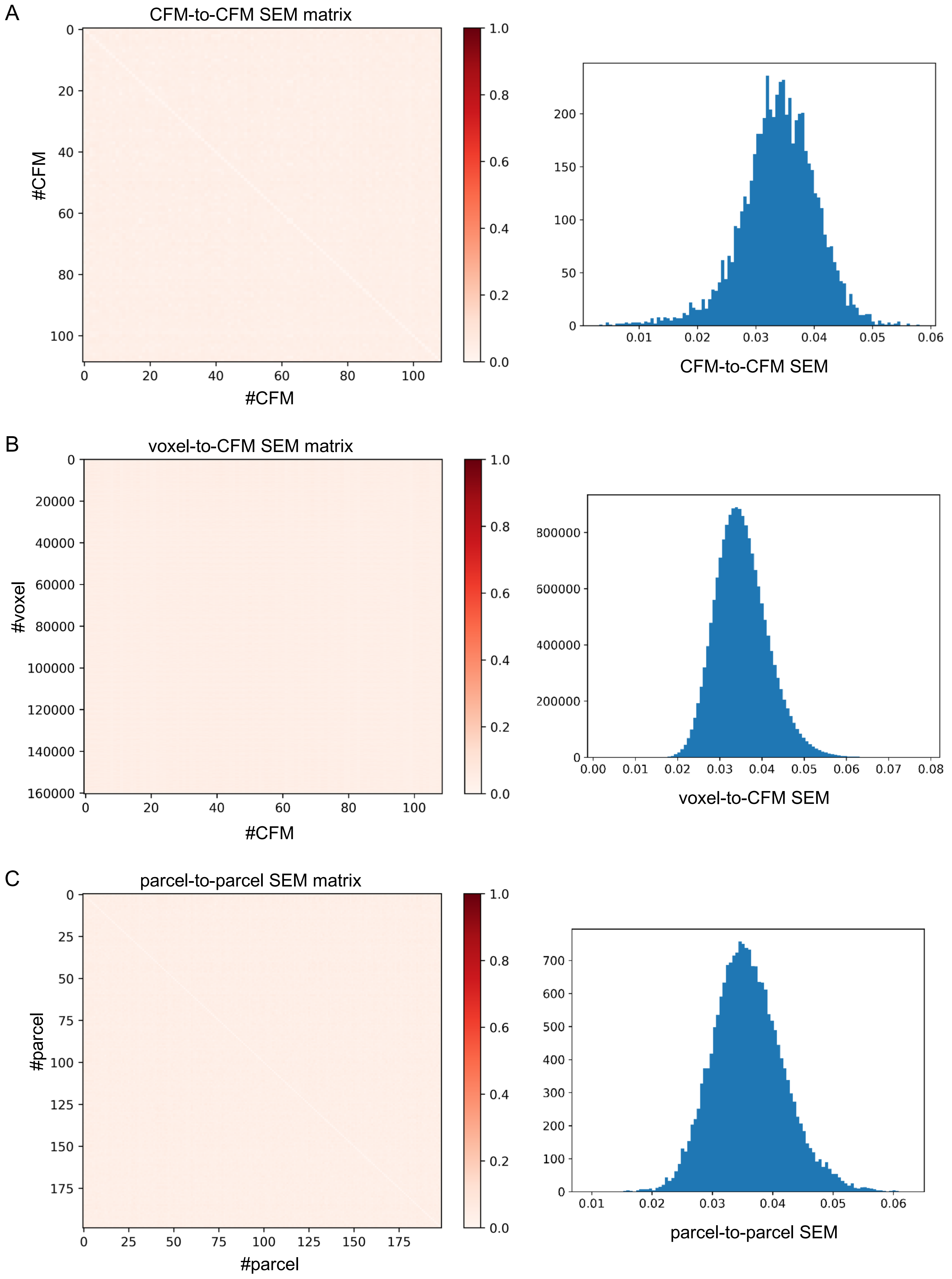

Supplementary Figure 4: Standard errors of means (SEMs) of the RSFCs. The matrices (left) and the histograms (right) of the SEM values of the CFM-to-CFM RSFCs (A), the voxel-to-CFM RSFCs (B), and the parcel-to-parcel RSFCs (C).
