# Supplementary material for: Revealing Relationships Among Cognitive Functions Using Functional Connectivity and a Large-Scale Meta-Analysis Database
Source: Front Hum Neurosci. 2020 Jan 10;13:457. doi: 10.3389/fnhum.2019.00457 (PMC6965330; doi:10.3389/fnhum.2019.00457)
Supplement: Supplementary file 19 [file Image_5.PDF]

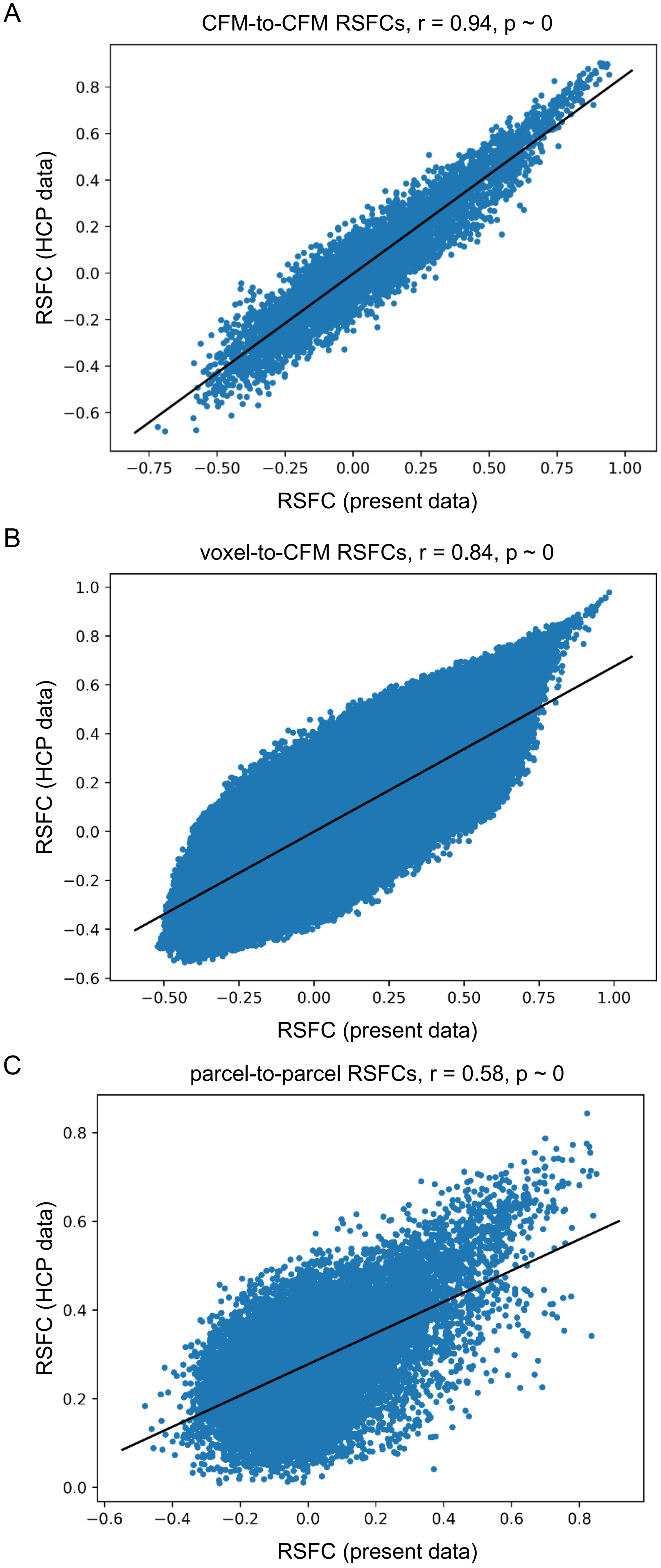

Supplementary Figure 5: Correlation analysis between the present data and the Human Connectome Project (HCP) data. (A) The CFM-to-CFM RSFCs. (B) The voxel-to-CFM RSFCs. (C) The parcel-to-parcel RSFCs. Note that the p-values were estimated to 0 because of very large sample sizes.
