# Supplementary material for: Revealing Relationships Among Cognitive Functions Using Functional Connectivity and a Large-Scale Meta-Analysis Database
Source: Front Hum Neurosci. 2020 Jan 10;13:457. doi: 10.3389/fnhum.2019.00457 (PMC6965330; doi:10.3389/fnhum.2019.00457)
Supplement: Supplementary file 20 [file Image_6.PDF]

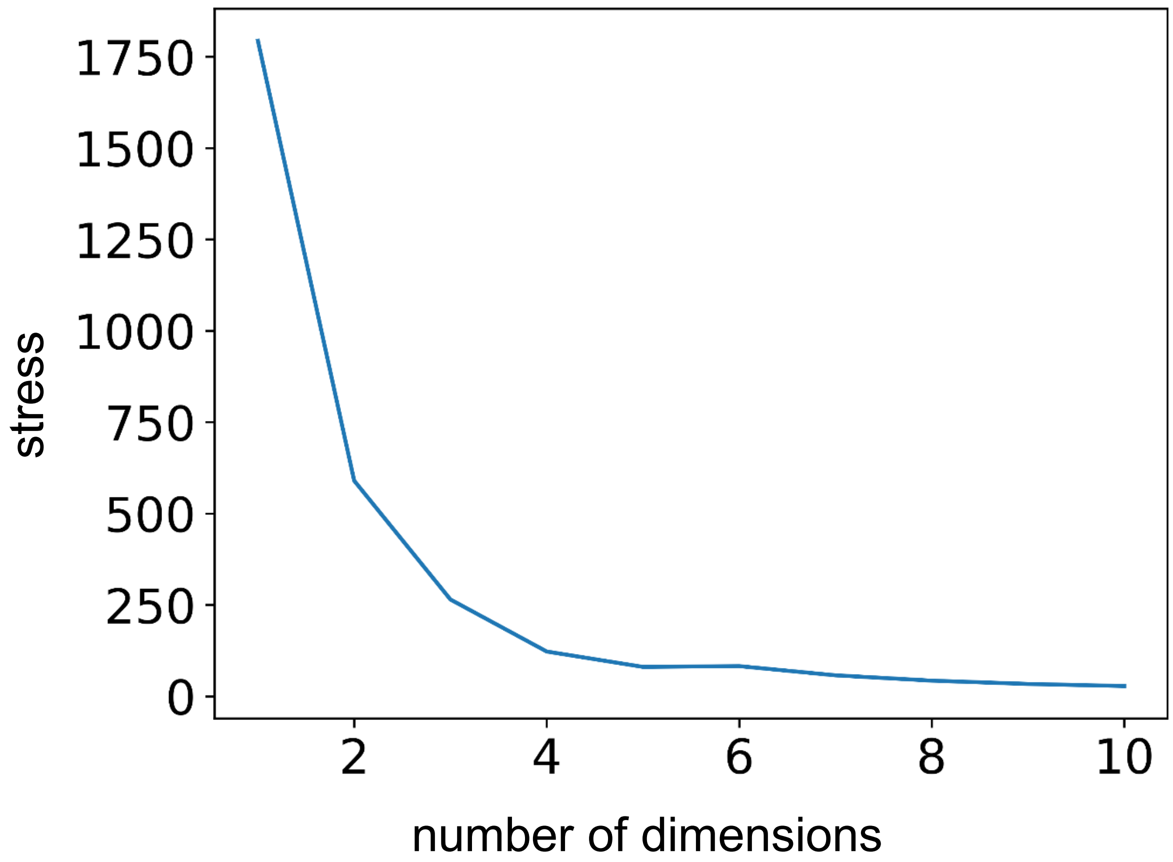

Supplementary Figure 6: Scree plot of the stresses to the number of dimensions in the multidimensional scaling. The stress is defined as the difference between given dissimilarities and distances in the embedding space and declines with an increase in the number of dimensions. According to the scree criterion, an optimal dimension seems to be four.
