# Supplementary material for: Revealing Relationships Among Cognitive Functions Using Functional Connectivity and a Large-Scale Meta-Analysis Database
Source: Front Hum Neurosci. 2020 Jan 10;13:457. doi: 10.3389/fnhum.2019.00457 (PMC6965330; doi:10.3389/fnhum.2019.00457)

presented in the following pages

Supplementary Figure 7: Relational mapping of cognitive functions, focusing on each cognitive function. In the panel for each cognitive function, only the connections linked to the cognitive function are shown. The positive and negative strengths are color-coded in red and blue, respectively.

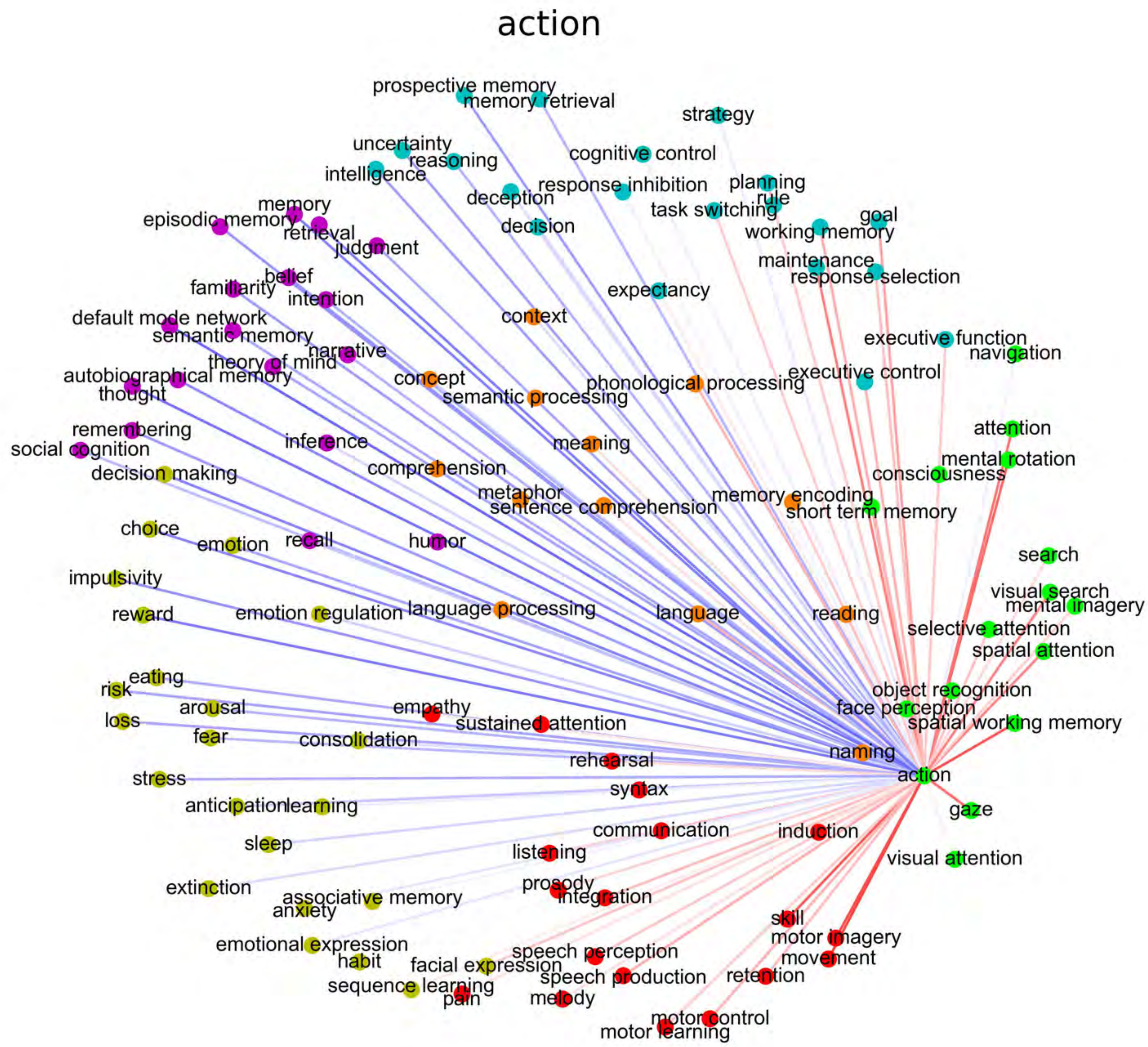

action
prospective memory
memory retrieval
strategy
uncertainty
reasoning
cognitive control
intelligence
response inhibition
planning
deception
rule
memory
task switching
goal
episodic memory
decision
working memory
retrieval
judgment
maintenance
response selection
belief
familiarity
intention
expectancy
default mode network
context
semantic memory
executive function
navigation
narrative
theory of mind
executive control
concept
phonological processing
autobiographical memory
thought
semantic processing
remembering
attention
inference
meaning
social cognition
decision making
mental rotation
comprehension
consciousness
metaphor
memory encoding
sentence comprehension
short term memory
choice
emotion
recall
humor
search
impulsivity
visual search
mental imagery
reward
emotion regulation
language processing
language
reading
selective attention
spatial attention
eating
risk
object recognition
arousal
empathy
face perception
loss
sustained attention
spatial working memory
fear
consolidation
naming
rehearsal
stress
action
syntax
anticipation
learning
gaze
communication
induction
sleep
listening
visual attention
extinction
prosody
integration
associative memory
anxiety
skill
motor imagery
emotional expression
speech perception
movement
habit
facial expression
speech production
retention
sequence learning
pain
melody
motor control
motor learning

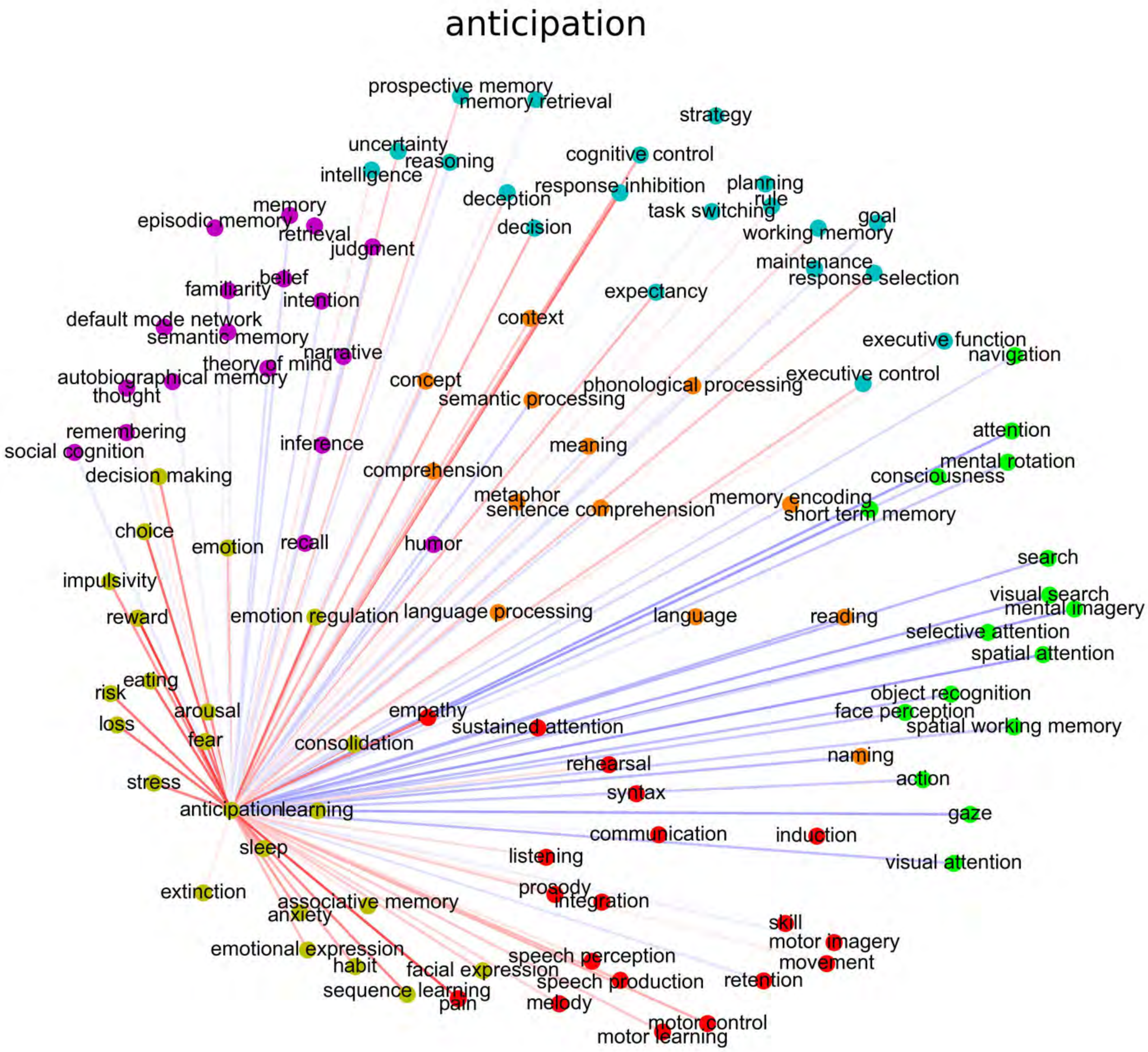
anticipation
prospective memory
memory retrieval
strategy
uncertainty
reasoning
cognitive control
intelligence
response inhibition
planning
deception
rule
task switching
memory
episodic memory
retrieval
decision
goal
working memory
judgment
maintenance
response selection
belief
familiarity
intention
expectancy
context
default mode network
semantic memory
executive function
navigation
narrative
theory of mind
autobiographical memory
concept
phonological processing
executive control
thought
semantic processing
remembering
attention
social cognition
inference
meaning
mental rotation
decision making
comprehension
consciousness
metaphor
memory encoding
sentence comprehension
short term memory
choice
emotion
recall
humor
search
impulsivity
visual search
mental imagery
reward
emotion regulation
language processing
language
reading
selective attention
spatial attention
eating
risk
object recognition
arousal
empathy
face perception
loss
sustained attention
spatial working memory
fear
consolidation
naming
rehearsal
stress
action
syntax
anticipation
learning
gaze
communication
induction
sleep
listening
visual attention
extinction
prosody
integration
associative memory
anxiety
skill
motor imagery
emotional expression
speech perception
habit
facial expression
movement
speech production
retention
sequence learning
pain
melody
motor control
motor learning

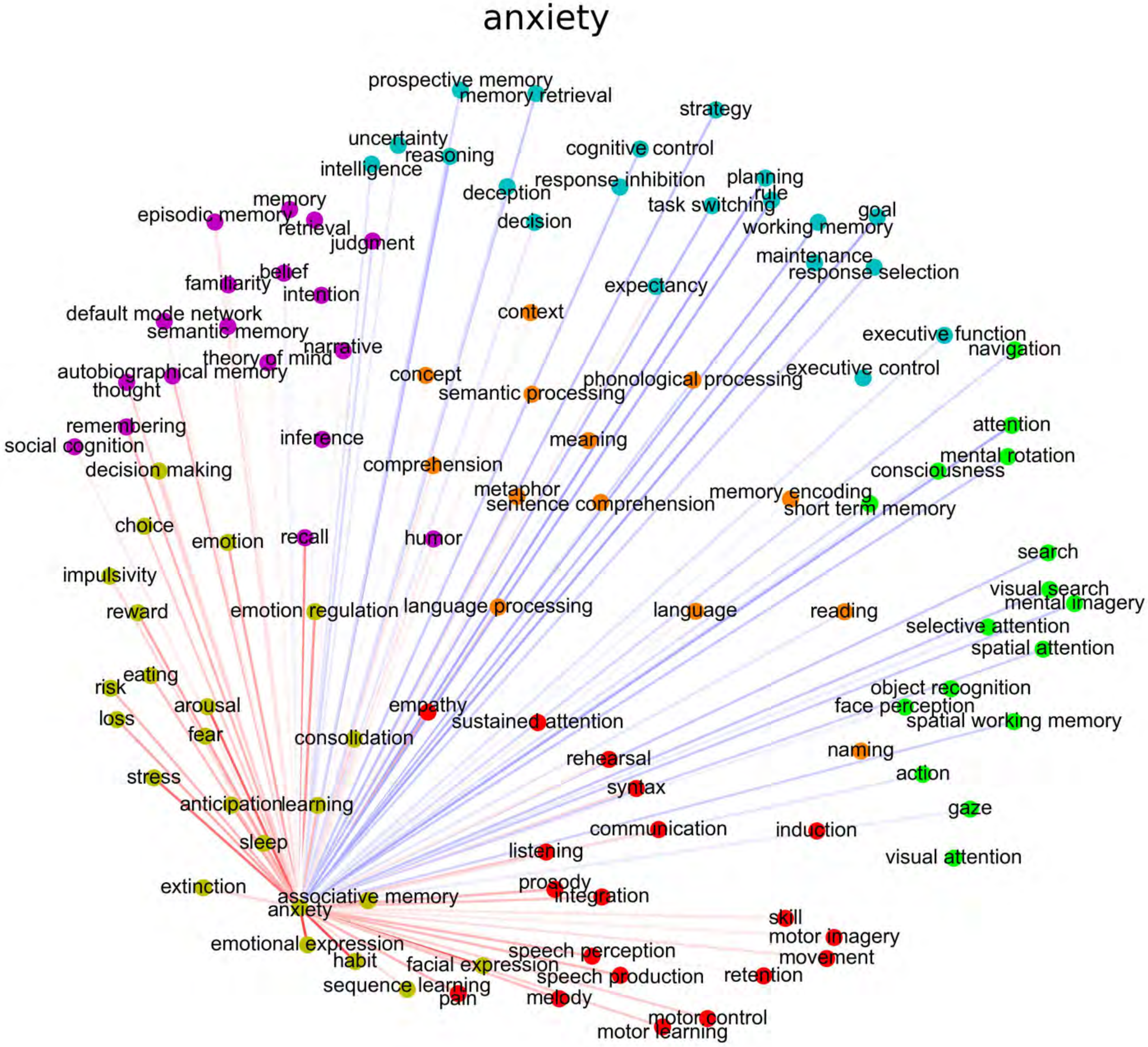
anxiety
prospective memory
memory retrieval
strategy
uncertainty
reasoning
cognitive control
intelligence
response inhibition
planning
deception
rule
task switching
goal
decision
working memory
memory
episodic memory
retrieval
judgment
maintenance
response selection
belief
familiarity
intention
expectancy
context
default mode network
semantic memory
executive function
navigation
narrative
theory of mind
executive control
concept
phonological processing
autobiographical memory
thought
semantic processing
remembering
social cognition
inference
meaning
attention
mental rotation
decision making
comprehension
consciousness
metaphor
memory encoding
sentence comprehension
short term memory
choice
emotion
recall
humor
search
impulsivity
visual search
mental imagery
reward
emotion regulation
language processing
language
reading
selective attention
spatial attention
eating
risk
object recognition
arousal
empathy
face perception
loss
sustained attention
spatial working memory
fear
consolidation
rehearsal
naming
stress
action
syntax
anticipation
learning
communication
induction
gaze
sleep
listening
visual attention
extinction
prosody
integration
associative memory
anxiety
skill
motor imagery
emotional expression
speech perception
movement
habit
facial expression
speech production
retention
sequence learning
pain
melody
motor control
motor learning

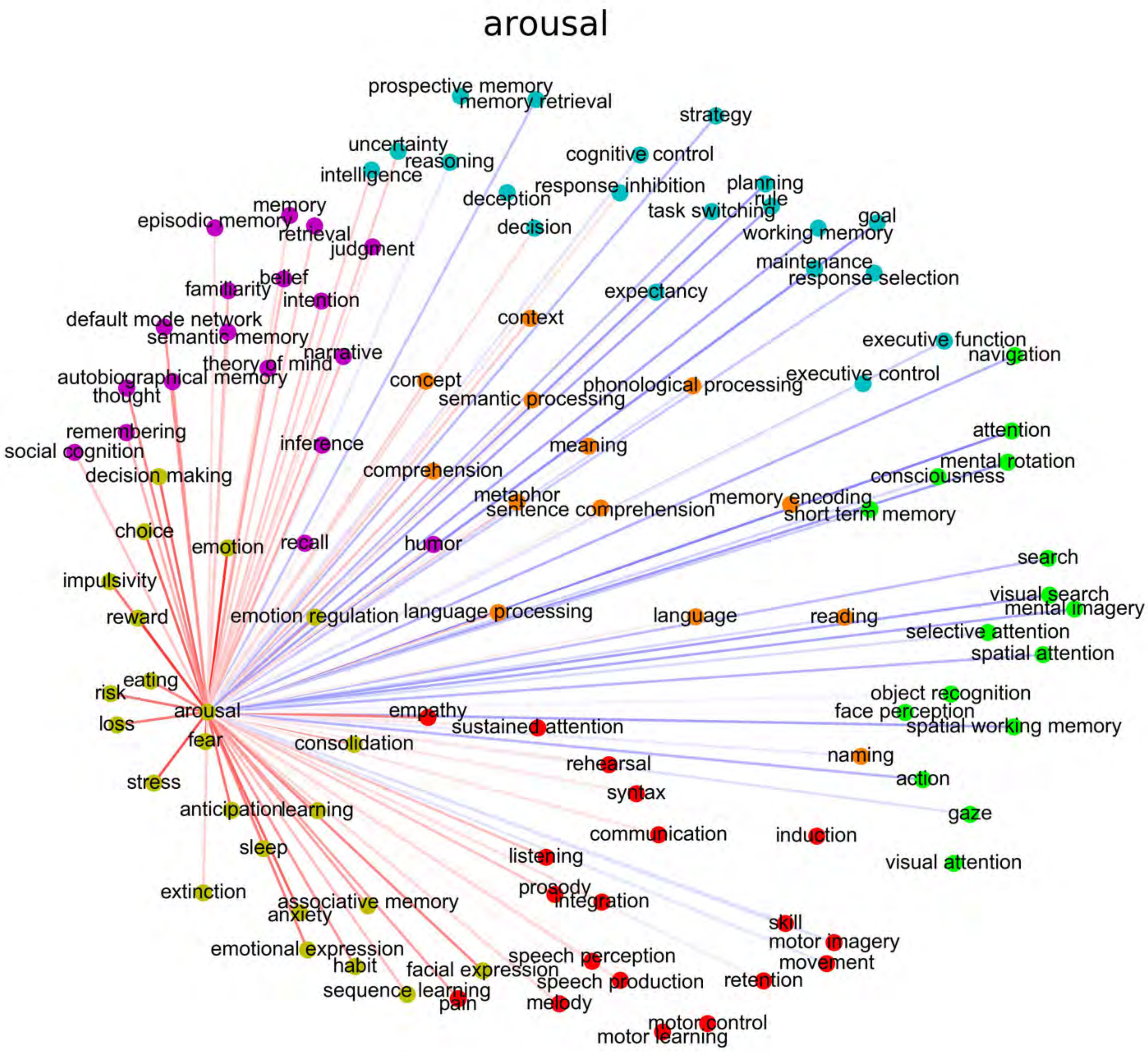
arousal
prospective memory
memory retrieval
strategy
uncertainty
reasoning
cognitive control
intelligence
response inhibition
planning
rule
deception
task switching
memory
episodic memory
retrieval
decision
goal
working memory
judgment
maintenance
response selection
belief
familiarity
intention
expectancy
context
default mode network
semantic memory
executive function
navigation
narrative
theory of mind
concept
executive control
autobiographical memory
phonological processing
thought
semantic processing
remembering
meaning
attention
social cognition
inference
mental rotation
decision making
comprehension
consciousness
metaphor
memory encoding
sentence comprehension
short term memory
choice
emotion
recall
humor
search
impulsivity
visual search
mental imagery
reward
emotion regulation
language processing
language
reading
selective attention
spatial attention
eating
object recognition
risk
arousal
empathy
face perception
loss
sustained attention
spatial working memory
fear
consolidation
naming
rehearsal
stress
action
syntax
anticipation
learning
gaze
communication
induction
sleep
listening
visual attention
extinction
prosody
integration
associative memory
anxiety
skill
motor imagery
emotional expression
speech perception
movement
habit
facial expression
speech production
retention
sequence learning
pain
melody
motor control
motor learning

attention

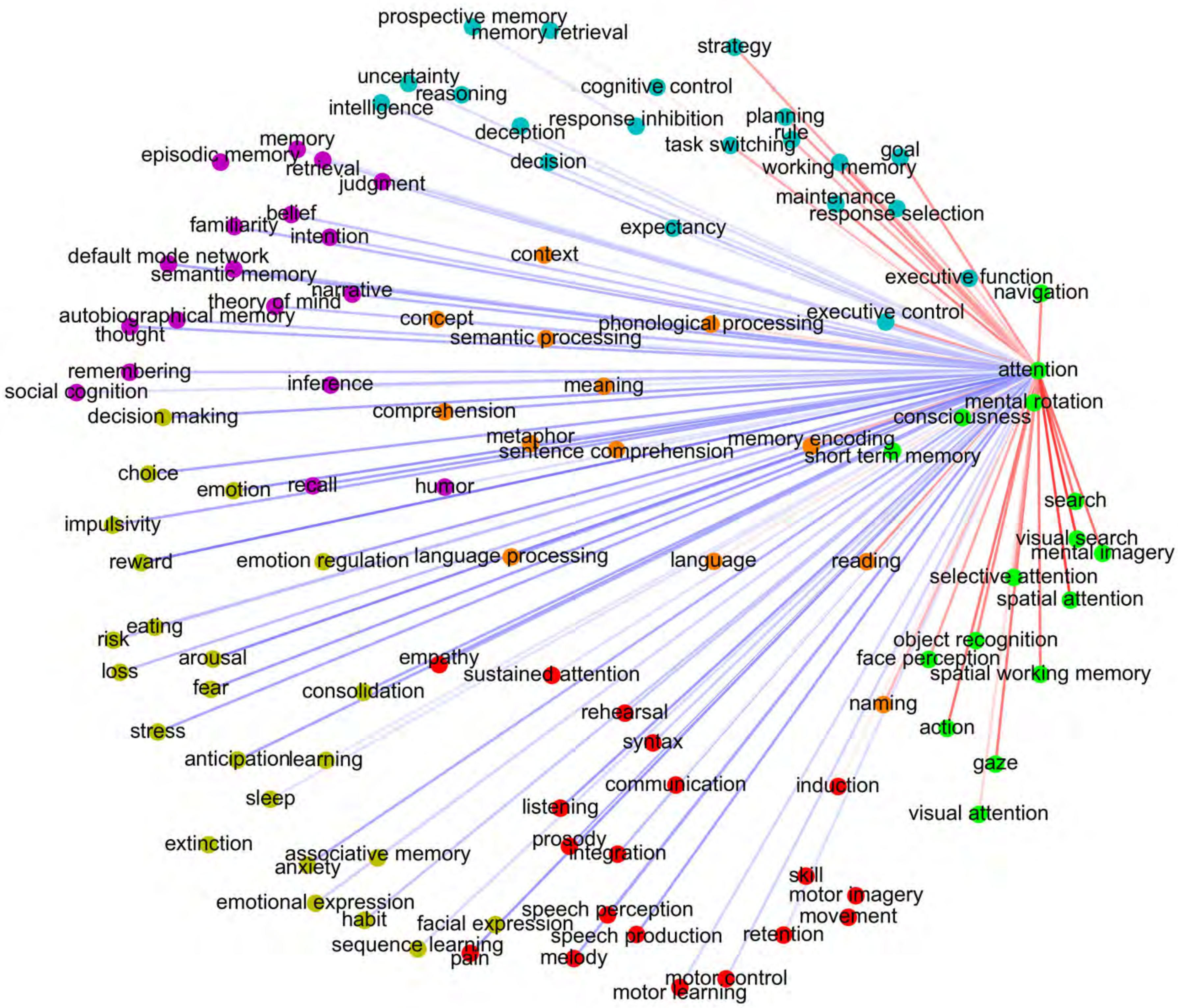

prospective memory
memory retrieval
strategy
uncertainty
reasoning
cognitive control
intelligence
response inhibition
planning
rule
deception
memory
episodic memory
retrieval
task switching
goal
decision
working memory
judgment
maintenance
response selection
belief
familiarity
intention
expectancy
default mode network
context
semantic memory
executive function
navigation
narrative
theory of mind
executive control
autobiographical memory
concept
phonological processing
thought
semantic processing
remembering
attention
social cognition
inference
meaning
decision making
mental rotation
comprehension
consciousness
metaphor
memory encoding
sentence comprehension
short term memory
choice
emotion
recall
humor
search
impulsivity
visual search
mental imagery
reward
emotion regulation
language processing
language
reading
selective attention
spatial attention
eating
risk
object recognition
arousal
empathy
face perception
loss
sustained attention
spatial working memory
fear
consolidation
rehearsal
naming
stress
action
syntax
anticipation
learning
gaze
communication
induction
sleep
listening
visual attention
extinction
prosody
integration
associative memory
anxiety
skill
motor imagery
emotional expression
speech perception
habit
movement
facial expression
speech production
retention
sequence learning
pain
melody
motor control
motor learning

autobiographical memory

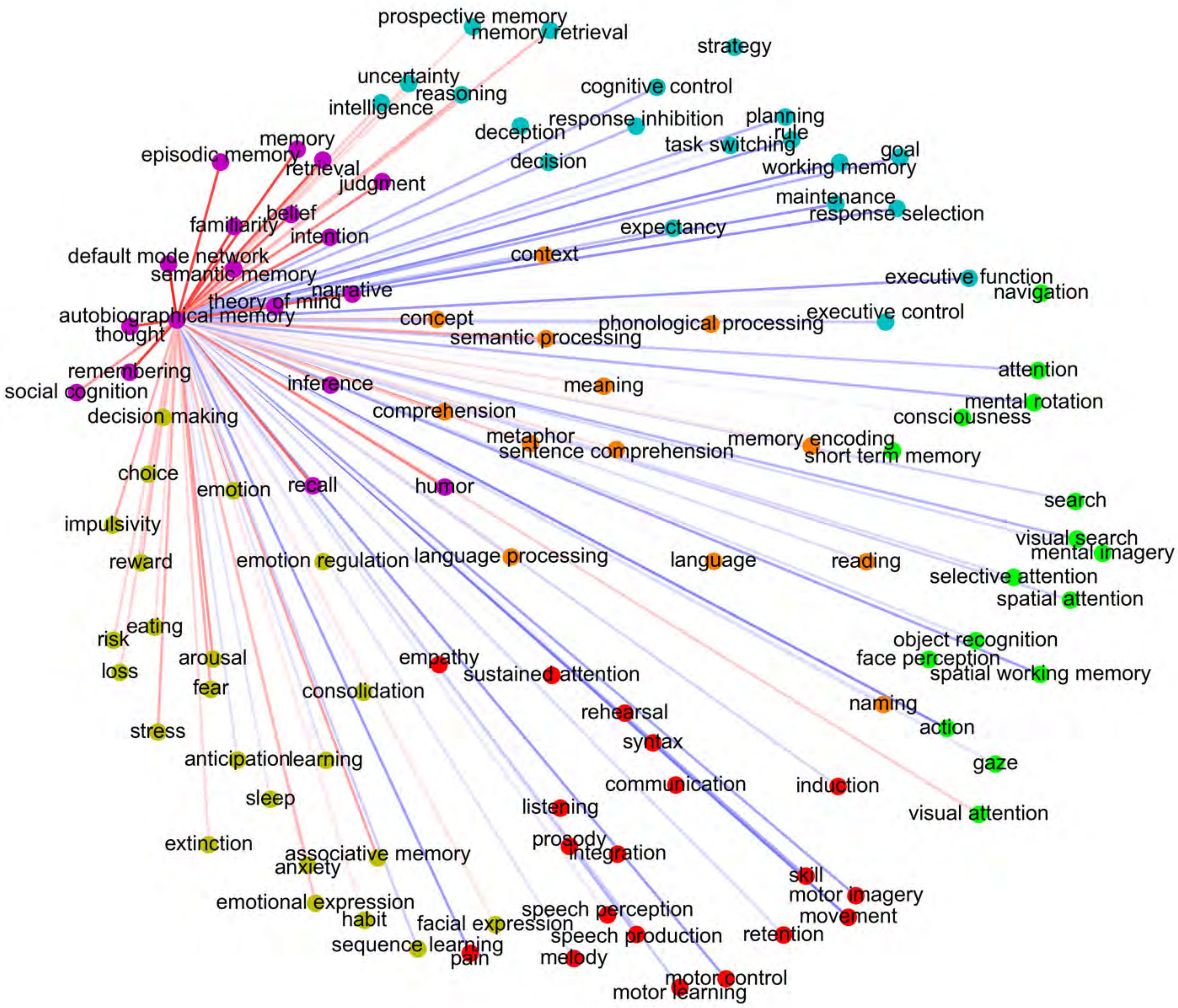

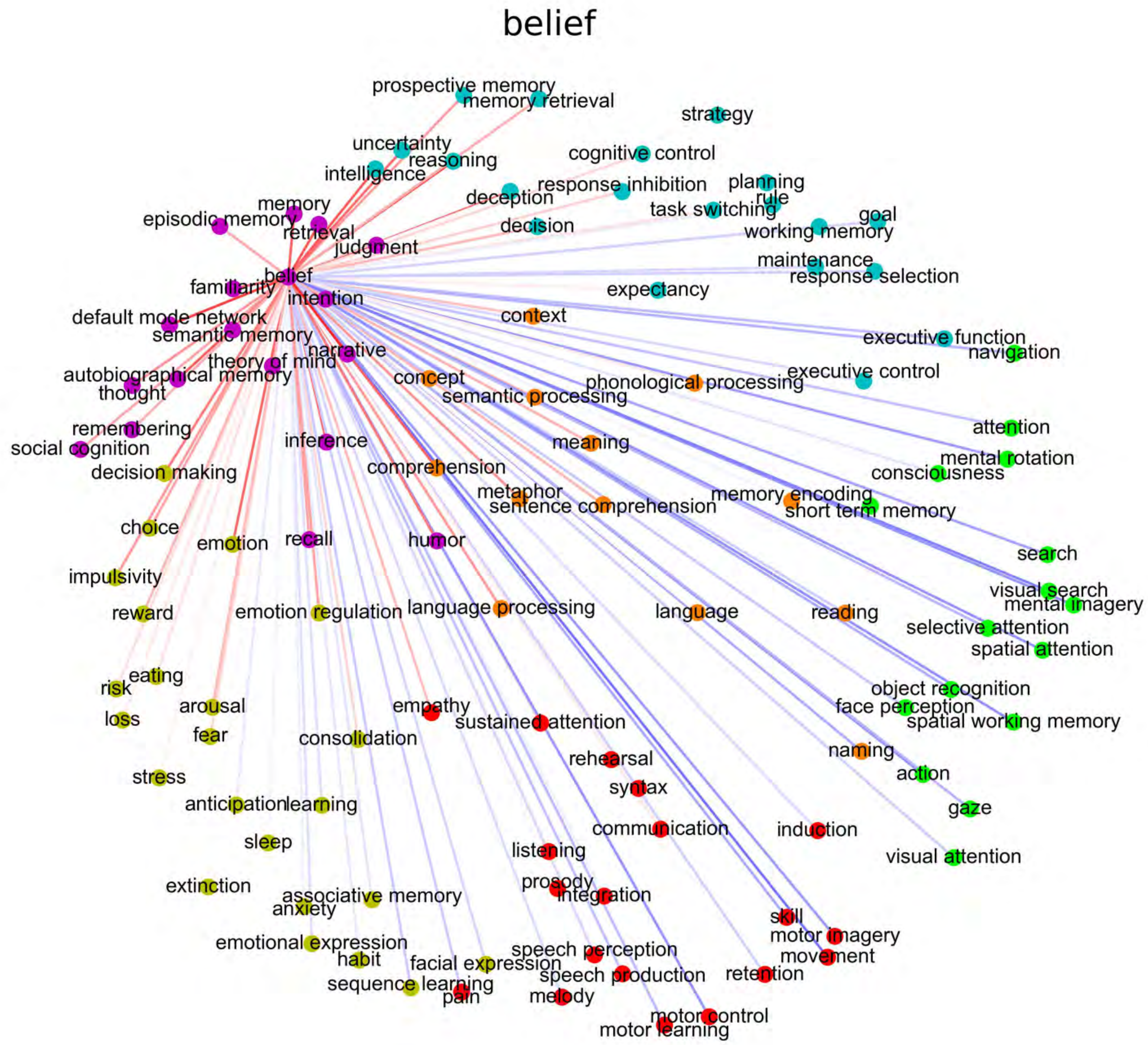
belief
prospective memory
memory retrieval
strategy
uncertainty
reasoning
cognitive control
intelligence
response inhibition
planning
rule
memory
deception
task switching
goal
episodic memory
retrieval
decision
working memory
judgment
maintenance
belief
response selection
familiarity
intention
expectancy
default mode network
context
semantic memory
executive function
navigation
narrative
theory of mind
executive control
autobiographical memory
concept
phonological processing
thought
semantic processing
remembering
attention
social cognition
inference
meaning
mental rotation
decision making
comprehension
consciousness
metaphor
memory encoding
sentence comprehension
short term memory
choice
emotion
recall
humor
search
impulsivity
visual search
mental imagery
reward
emotion regulation
language processing
language
reading
selective attention
spatial attention
eating
risk
object recognition
arousal
empathy
face perception
loss
sustained attention
spatial working memory
fear
consolidation
rehearsal
naming
stress
action
syntax
anticipation
learning
gaze
communication
induction
sleep
listening
visual attention
extinction
prosody
integration
associative memory
anxiety
skill
motor imagery
emotional expression
speech perception
movement
habit
facial expression
speech production
retention
sequence learning
pain
melody
motor control
motor learning

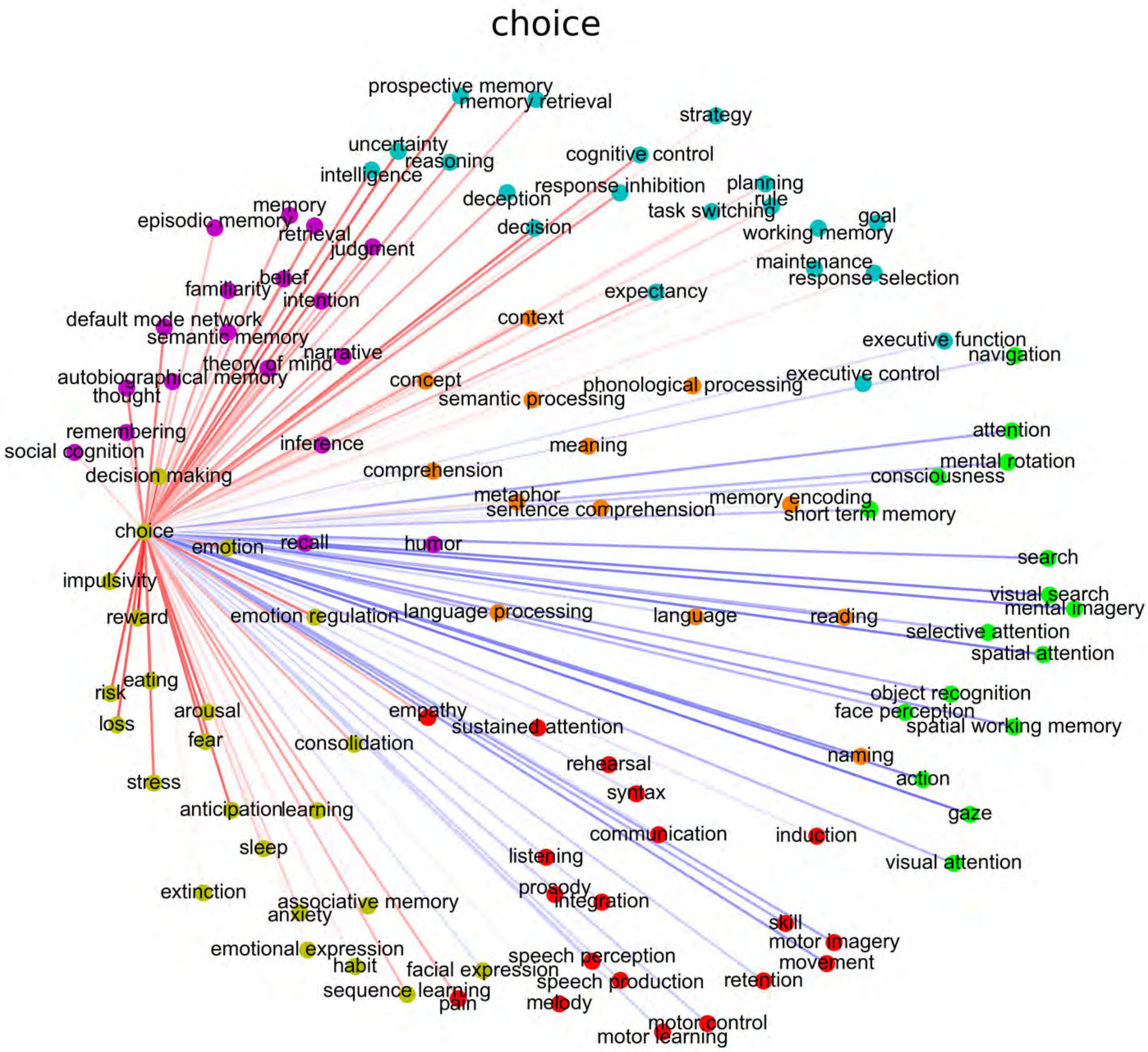
choice
prospective memory
memory retrieval
strategy
uncertainty
reasoning
intelligence
cognitive control
response inhibition
planning
rule
deception
task switching
goal
working memory
decision
memory
episodic memory
retrieval
judgment
maintenance
response selection
belief
familiarity
intention
expectancy
context
default mode network
semantic memory
executive function
navigation
narrative
theory of mind
concept
executive control
autobiographical memory
thought
phonological processing
semantic processing
remembering
social cognition
inference
meaning
attention
decision making
comprehension
mental rotation
consciousness
metaphor
memory encoding
sentence comprehension
short term memory
emotion
recall
humor
search
impulsivity
visual search
mental imagery
reward
emotion regulation
language processing
language
reading
selective attention
spatial attention
eating
risk
arousal
object recognition
loss
empathy
face perception
fear
sustained attention
spatial working memory
consolidation
rehearsal
naming
stress
action
syntax
anticipation
learning
gaze
sleep
communication
induction
listening
visual attention
extinction
prosody
integration
associative memory
anxiety
skill
motor imagery
emotional expression
movement
habit
speech perception
facial expression
speech production
retention
sequence learning
pain
melody
motor control
motor learning

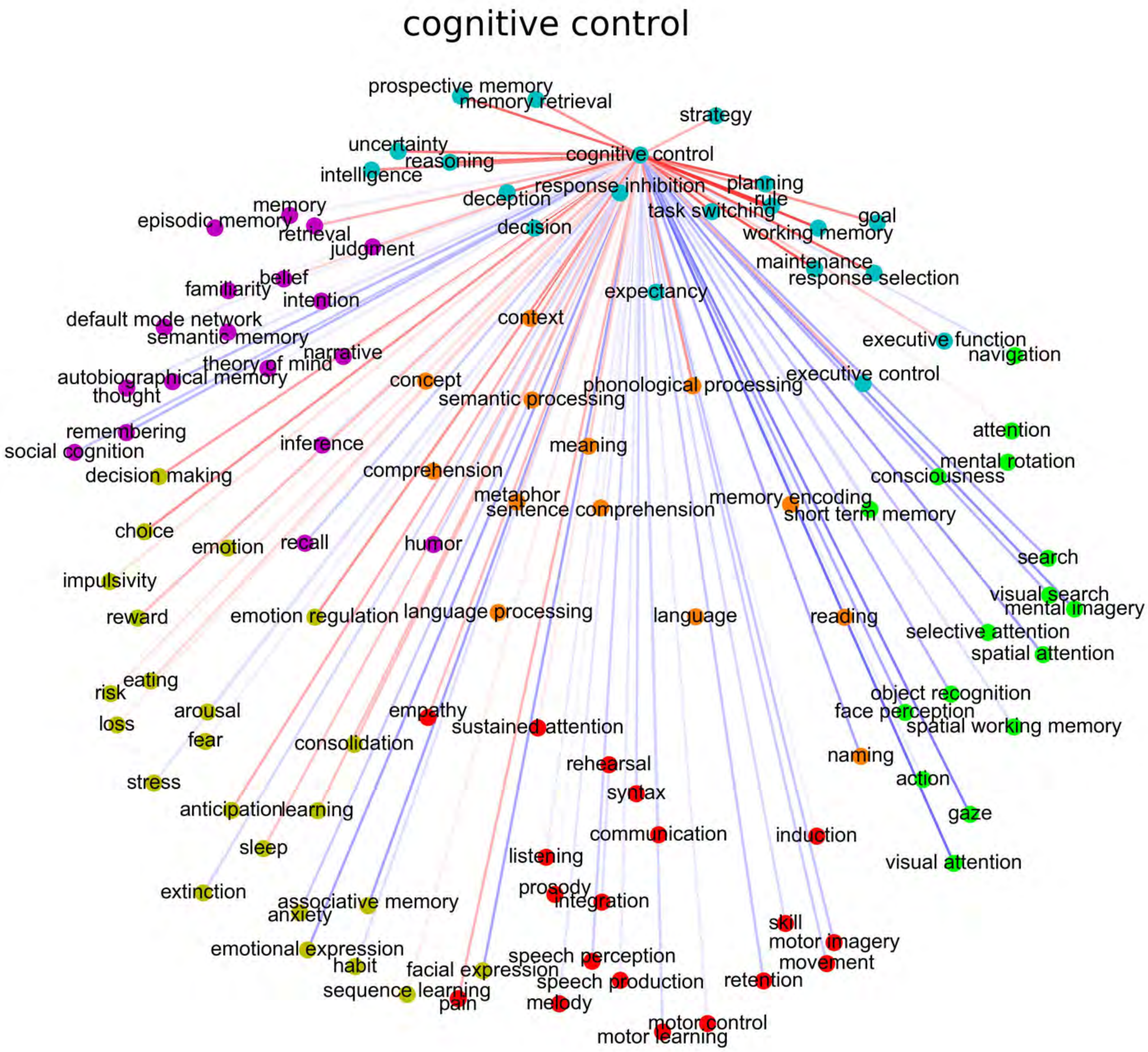
cognitive control
prospective memory
memory retrieval
strategy
uncertainty
reasoning
cognitive control
intelligence
planning
response inhibition
rule
deception
memory
task switching
goal
episodic memory
retrieval
decision
working memory
judgment
maintenance
belief
response selection
familiarity
intention
expectancy
default mode network
context
semantic memory
executive function
navigation
narrative
theory of mind
executive control
concept
phonological processing
autobiographical memory
thought
semantic processing
remembering
attention
inference
meaning
social cognition
mental rotation
decision making
comprehension
consciousness
metaphor
memory encoding
sentence comprehension
short term memory
choice
emotion
recall
humor
search
impulsivity
visual search
mental imagery
reward
emotion regulation
language processing
language
reading
selective attention
spatial attention
eating
risk
arousal
object recognition
empathy
face perception
loss
sustained attention
spatial working memory
fear
consolidation
naming
rehearsal
stress
action
syntax
anticipation
learning
gaze
communication
induction
sleep
listening
visual attention
extinction
prosody
integration
associative memory
anxiety
skill
motor imagery
emotional expression
speech perception
habit
movement
facial expression
speech production
retention
sequence learning
pain
melody
motor control
motor learning

communication

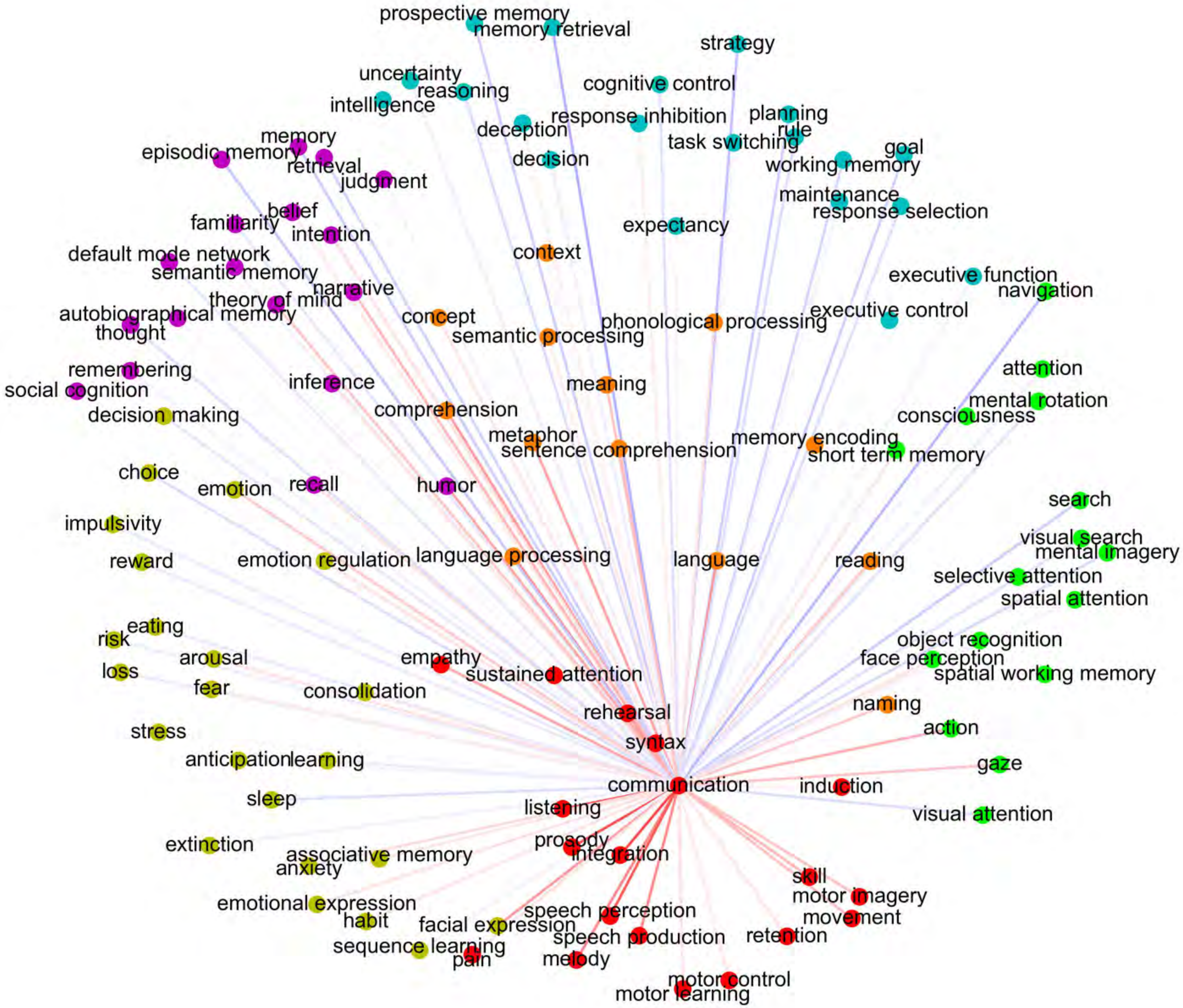

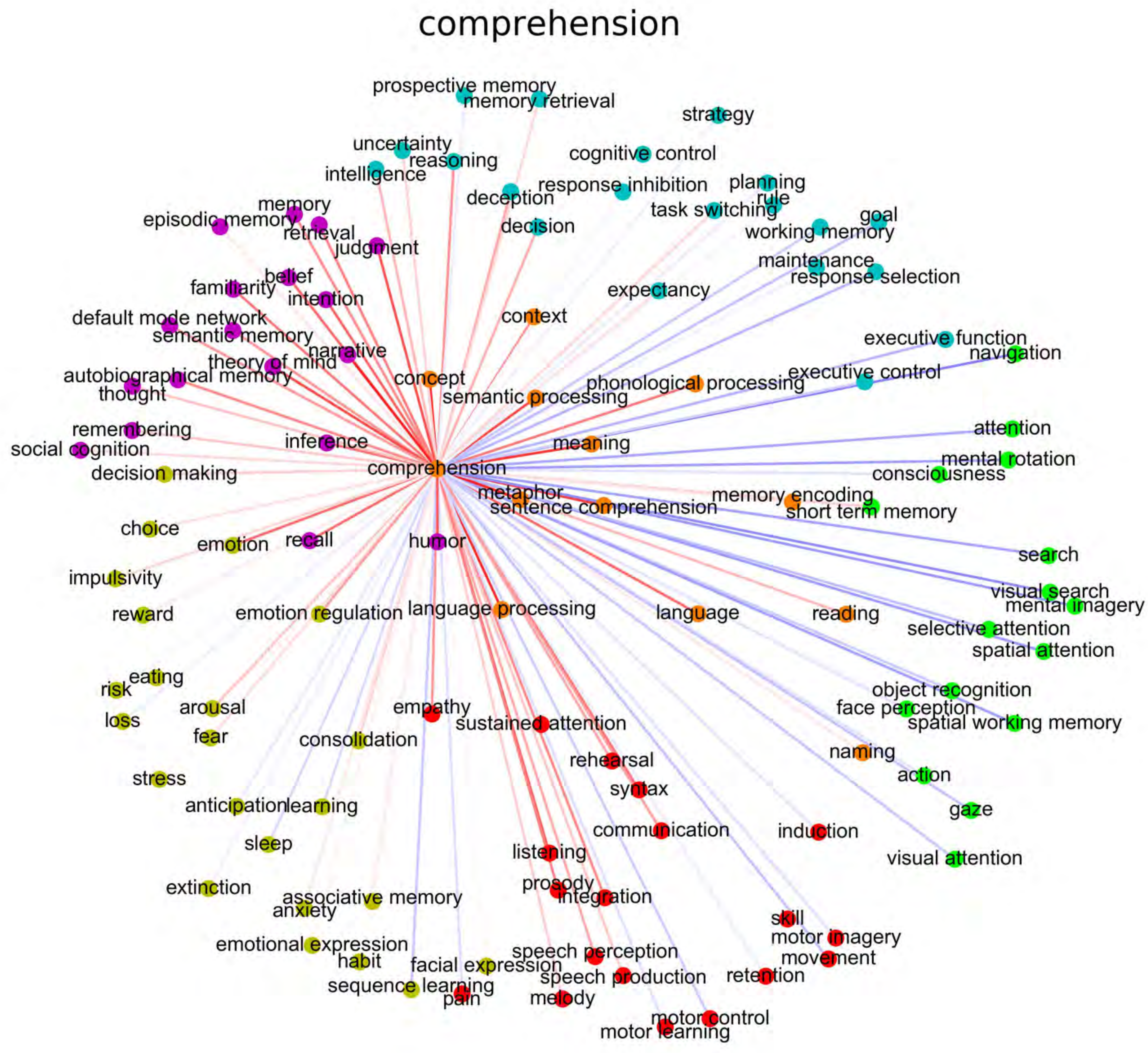
comprehension
prospective memory
memory retrieval
strategy
uncertainty
reasoning
intelligence
cognitive control
response inhibition
planning
rule
deception
task switching
goal
working memory
decision
memory
episodic memory
retrieval
judgment
maintenance
response selection
belief
familiarity
intention
expectancy
context
default mode network
semantic memory
executive function
navigation
narrative
theory of mind
concept
executive control
autobiographical memory
thought
phonological processing
semantic processing
remembering
attention
social cognition
inference
meaning
mental rotation
decision making
comprehension
consciousness
metaphor
memory encoding
sentence comprehension
short term memory
choice
emotion
recall
humor
search
impulsivity
visual search
mental imagery
reward
emotion regulation
language processing
language
reading
selective attention
spatial attention
eating
risk
object recognition
arousal
empathy
face perception
loss
sustained attention
spatial working memory
fear
consolidation
rehearsal
naming
stress
action
syntax
anticipation
learning
gaze
sleep
communication
induction
listening
visual attention
extinction
prosody
integration
associative memory
anxiety
skill
motor imagery
emotional expression
speech perception
movement
habit
facial expression
speech production
retention
sequence learning
pain
melody
motor control
motor learning

concept

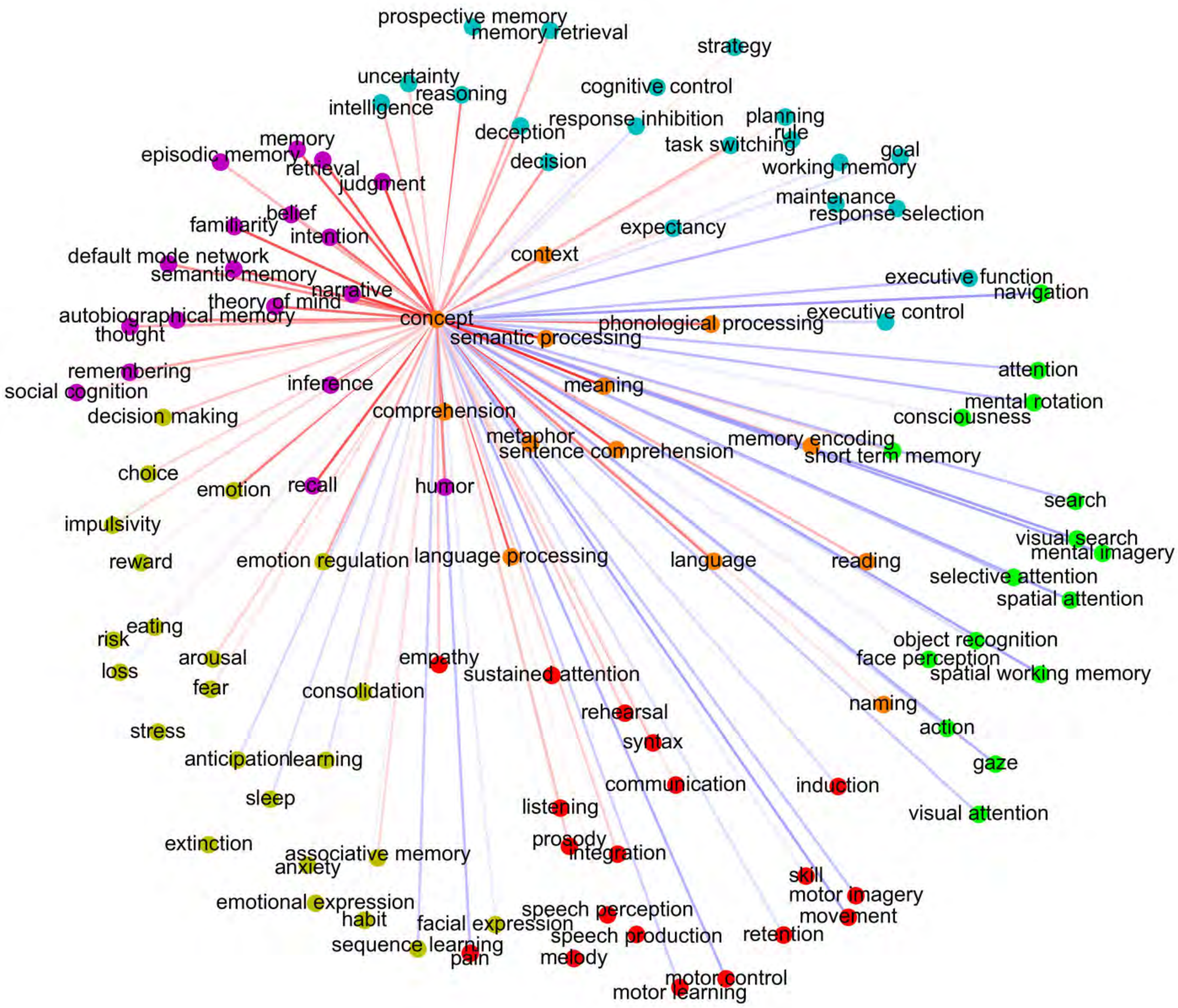

prospective memory
memory retrieval
strategy
uncertainty
reasoning
cognitive control
intelligence
deception
response inhibition
planning
rule
memory
episodic memory
retrieval
task switching
goal
decision
working memory
judgment
maintenance
belief
response selection
familiarity
intention
expectancy
context
default mode network
semantic memory
executive function
navigation
narrative
theory of mind
executive control
autobiographical memory
concept
phonological processing
thought
semantic processing
remembering
attention
inference
meaning
social cognition
decision making
mental rotation
comprehension
consciousness
metaphor
memory encoding
sentence comprehension
short term memory
choice
emotion
recall
humor
search
impulsivity
visual search
mental imagery
reward
emotion regulation
language processing
language
reading
selective attention
spatial attention
eating
risk
object recognition
arousal
empathy
face perception
loss
sustained attention
spatial working memory
fear
consolidation
rehearsal
naming
action
stress
syntax
anticipation
learning
gaze
communication
induction
sleep
listening
visual attention
extinction
prosody
integration
associative memory
anxiety
skill
motor imagery
emotional expression
speech perception
movement
habit
facial expression
speech production
retention
sequence learning
pain
melody
motor control
motor learning

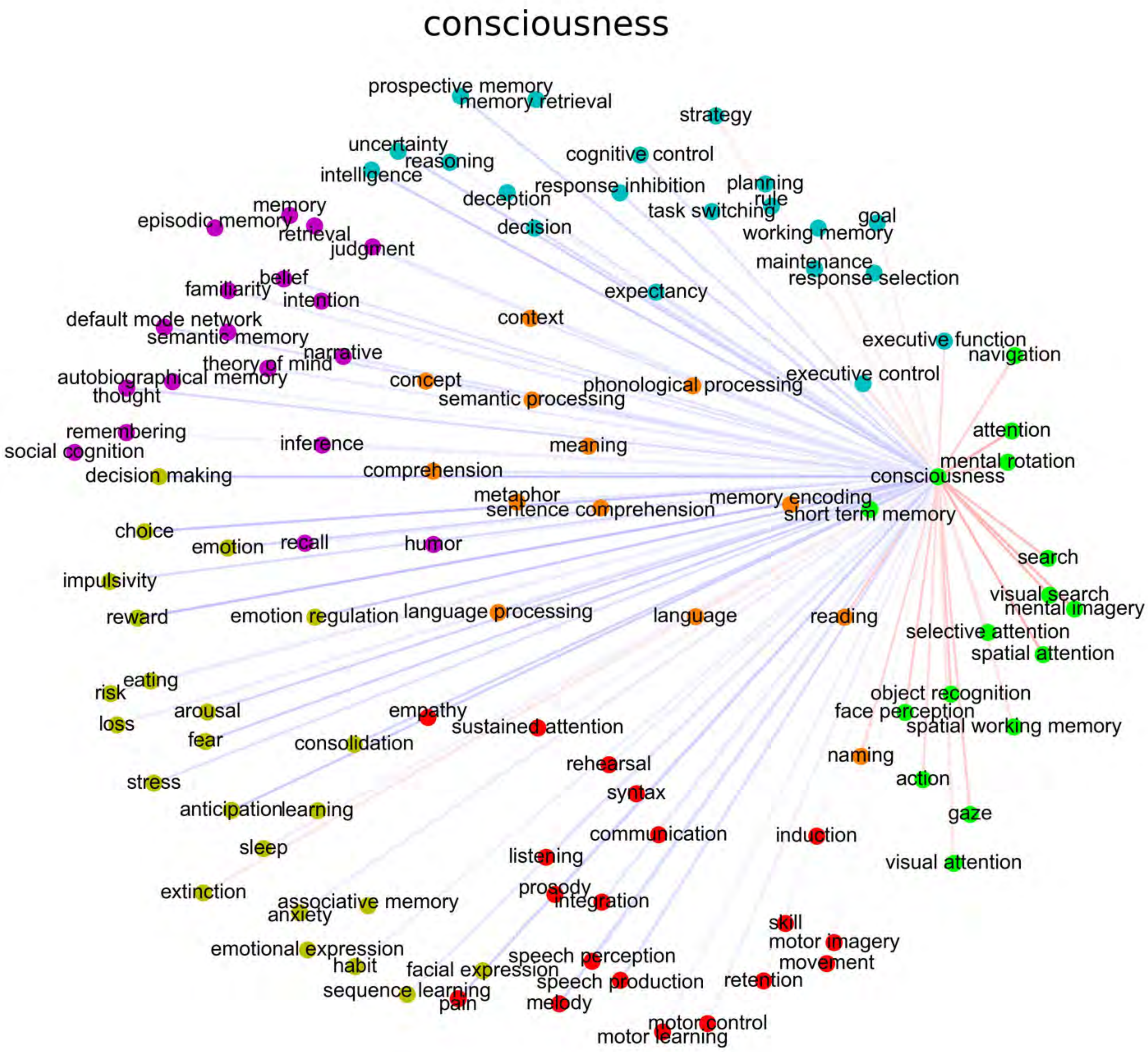
consciousness
prospective memory
memory retrieval
strategy
uncertainty
reasoning
cognitive control
intelligence
deception
response inhibition
planning
rule
memory
episodic memory
retrieval
decision
task switching
goal
working memory
judgment
maintenance
response selection
belief
familiarity
intention
expectancy
default mode network
semantic memory
context
executive function
navigation
narrative
theory of mind
autobiographical memory
concept
phonological processing
executive control
thought
semantic processing
remembering
attention
social cognition
inference
meaning
mental rotation
decision making
comprehension
consciousness
metaphor
sentence comprehension
memory encoding
short term memory
choice
emotion
recall
humor
search
impulsivity
visual search
mental imagery
reward
emotion regulation
language processing
language
reading
selective attention
spatial attention
eating
risk
arousal
object recognition
loss
empathy
face perception
fear
sustained attention
spatial working memory
consolidation
rehearsal
naming
stress
action
syntax
anticipation
learning
gaze
sleep
communication
induction
listening
visual attention
extinction
prosody
integration
associative memory
anxiety
skill
motor imagery
emotional expression
movement
habit
speech perception
facial expression
speech production
retention
sequence learning
pain
melody
motor control
motor learning

consolidation

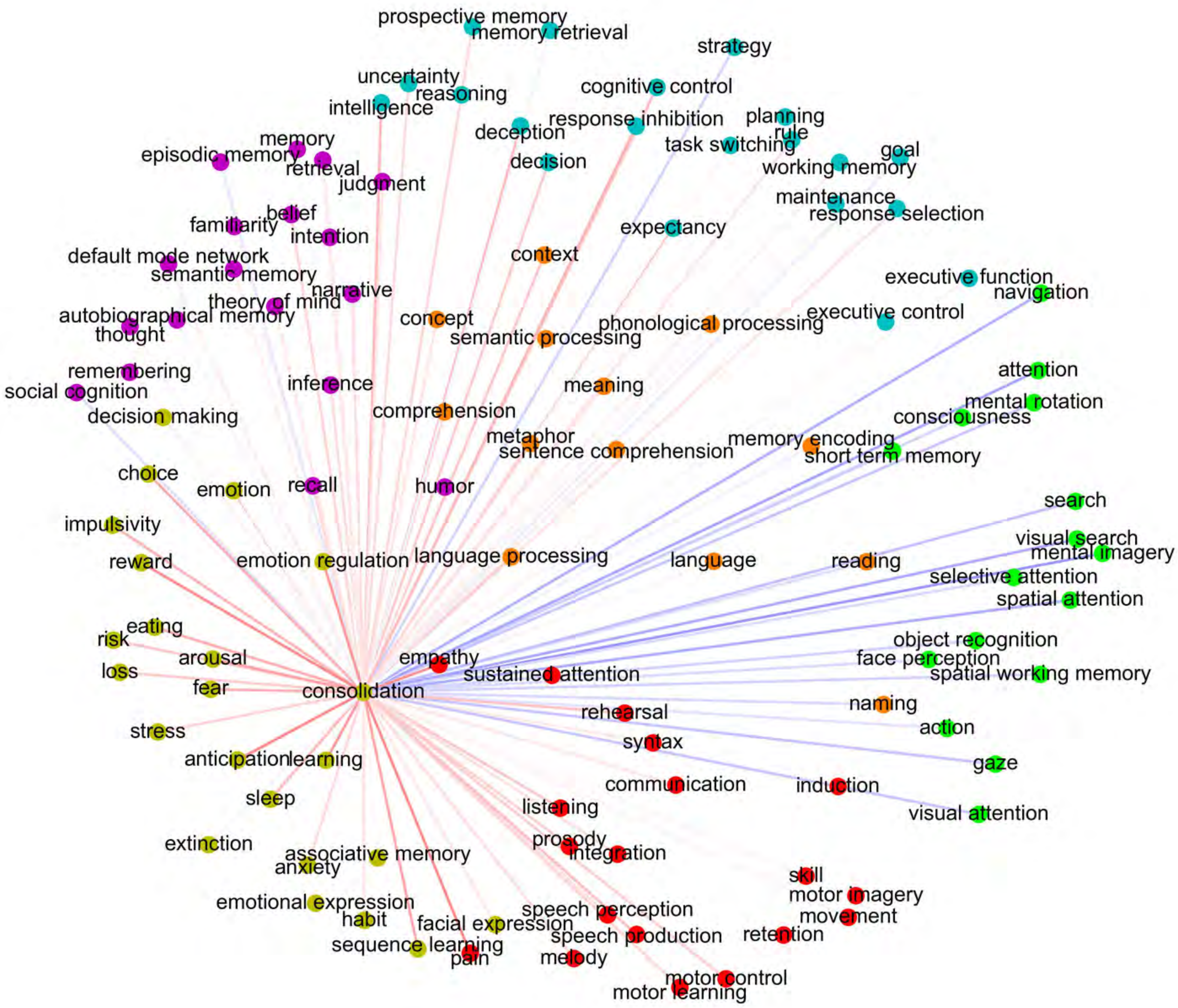

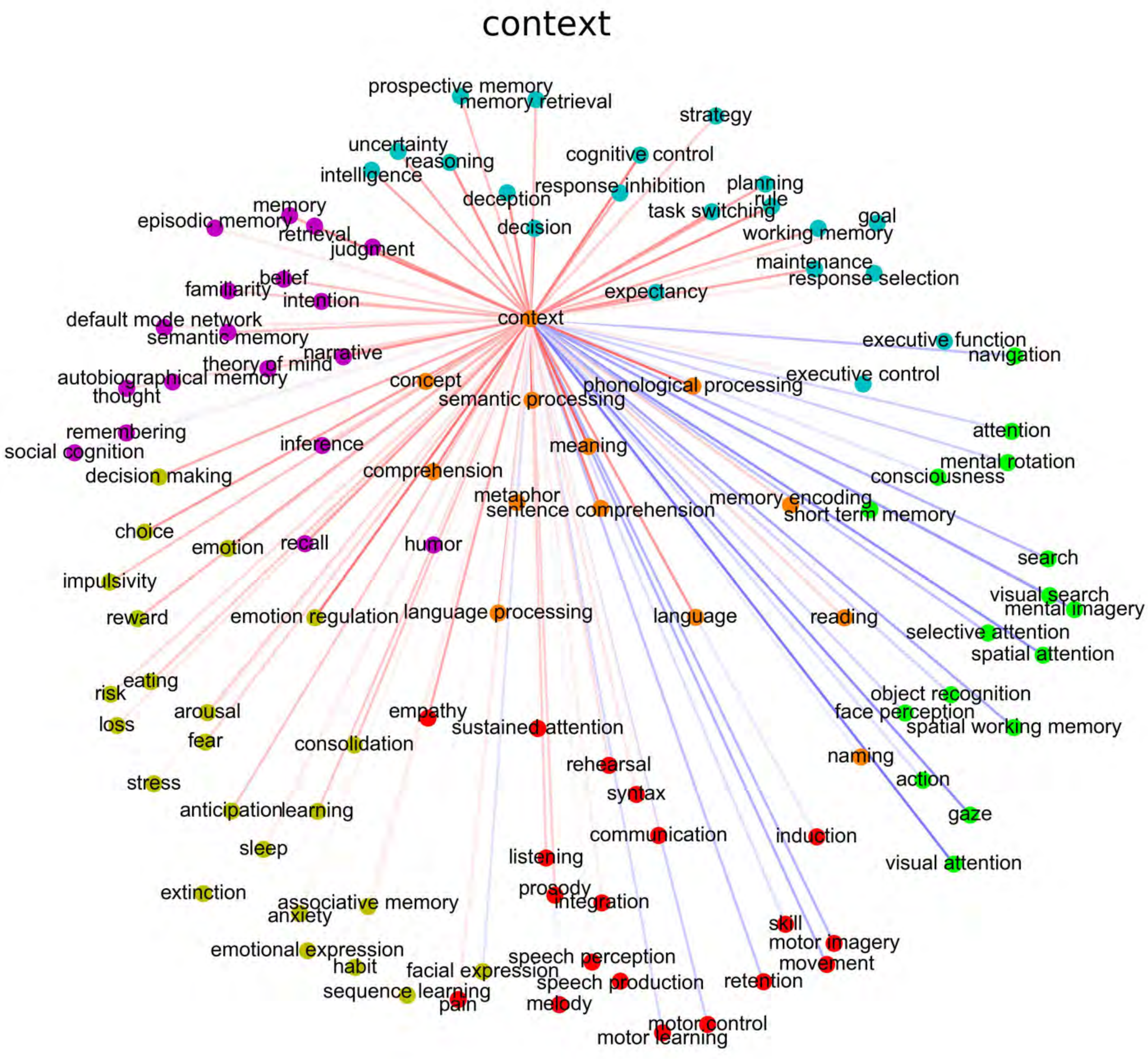
context
prospective memory
memory retrieval
strategy
uncertainty
reasoning
cognitive control
intelligence
response inhibition
planning
deception
rule
task switching
memory
goal
episodic memory
retrieval
decision
working memory
judgment
maintenance
belief
response selection
familiarity
intention
expectancy
default mode network
context
semantic memory
executive function
narrative
navigation
theory of mind
autobiographical memory
concept
executive control
thought
phonological processing
semantic processing
remembering
attention
social cognition
inference
meaning
mental rotation
decision making
comprehension
consciousness
metaphor
memory encoding
sentence comprehension
short term memory
choice
emotion
recall
humor
search
impulsivity
visual search
mental imagery
reward
emotion regulation
language processing
language
reading
selective attention
spatial attention
eating
risk
object recognition
arousal
empathy
face perception
loss
sustained attention
spatial working memory
fear
consolidation
rehearsal
naming
stress
syntax
action
anticipation
learning
gaze
communication
induction
sleep
listening
visual attention
extinction
prosody
associative memory
integration
anxiety
skill
motor imagery
emotional expression
speech perception
habit
movement
facial expression
speech production
retention
sequence learning
pain
melody
motor control
motor learning

deception

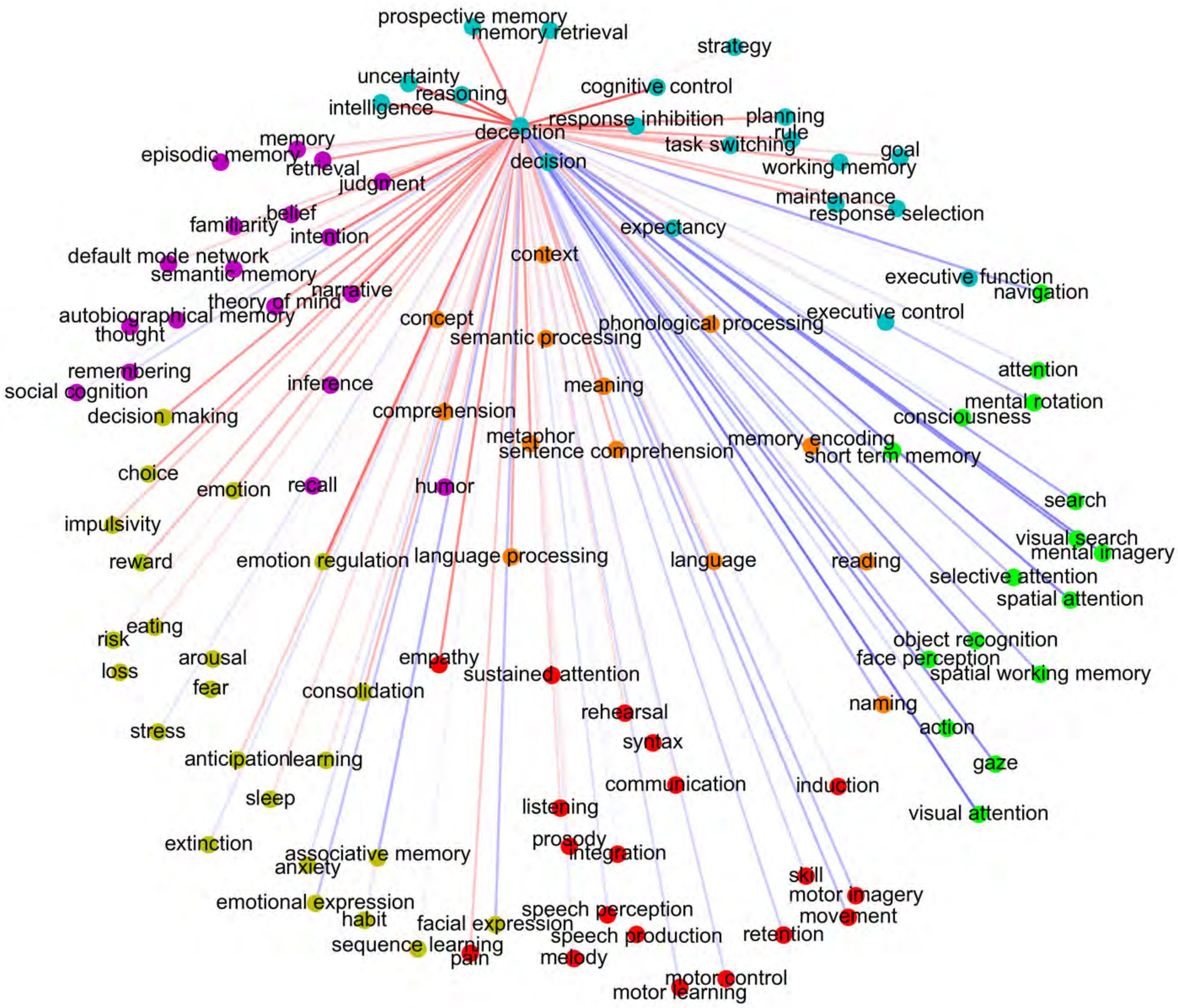
prospective memory
memory retrieval
strategy
uncertainty
reasoning
cognitive control
intelligence
response inhibition
planning
rule
deception
task switching
goal
memory
episodic memory
retrieval
decision
working memory
judgment
maintenance
response selection
belief
familiarity
intention
expectancy
default mode network
context
semantic memory
executive function
navigation
narrative
theory of mind
executive control
autobiographical memory
concept
phonological processing
thought
semantic processing
remembering
attention
inference
meaning
social cognition
mental rotation
decision making
comprehension
consciousness
metaphor
memory encoding
sentence comprehension
short term memory
choice
emotion
recall
humor
search
impulsivity
visual search
mental imagery
reward
emotion regulation
language processing
language
reading
selective attention
spatial attention
eating
risk
object recognition
arousal
empathy
face perception
loss
sustained attention
spatial working memory
fear
consolidation
naming
rehearsal
stress
action
syntax
anticipation
learning
gaze
communication
induction
sleep
listening
visual attention
extinction
prosody
integration
associative memory
anxiety
skill
motor imagery
emotional expression
speech perception
movement
habit
facial expression
speech production
retention
sequence learning
pain
melody
motor control
motor learning

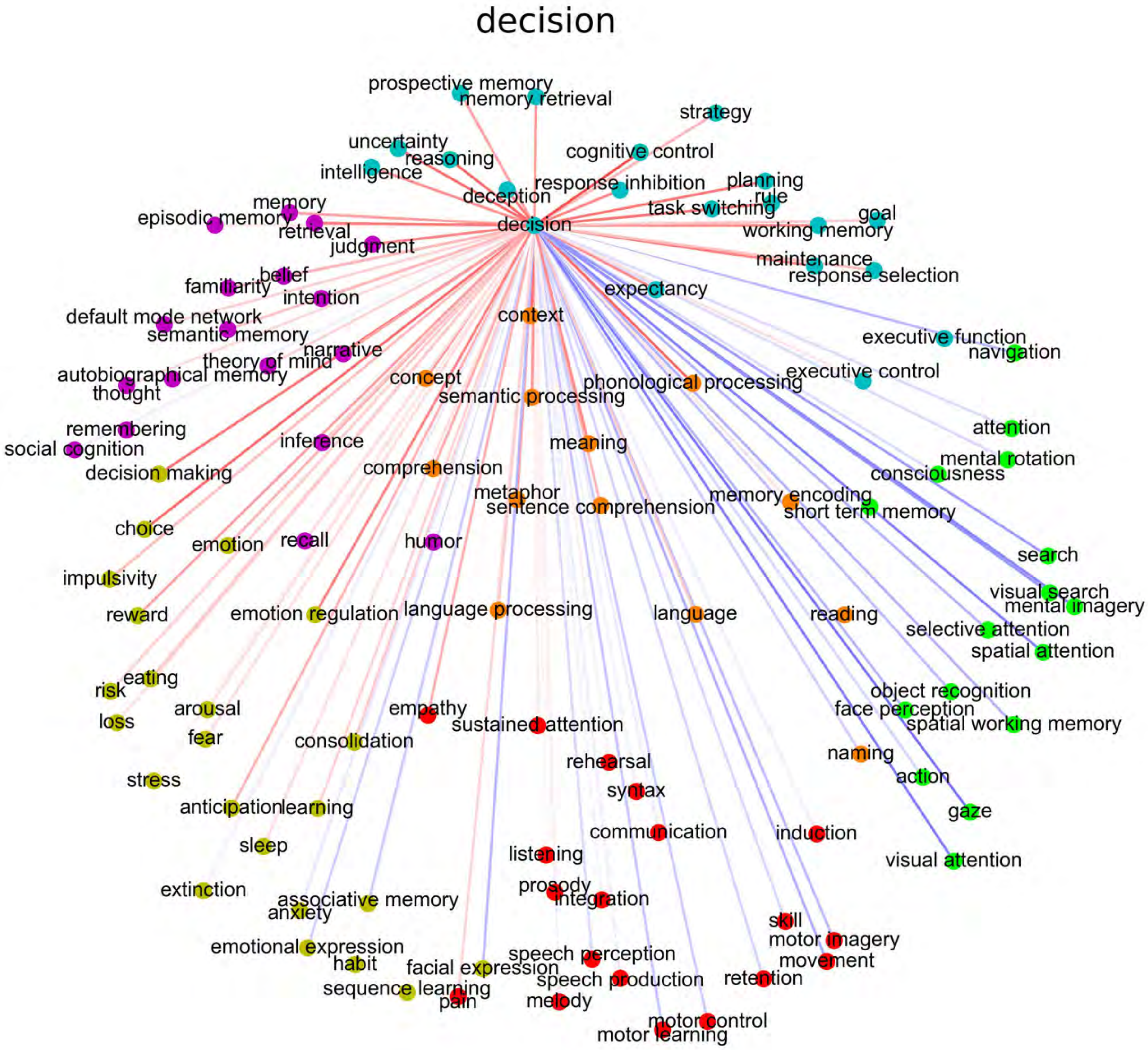
decision
prospective memory
memory retrieval
strategy
uncertainty
reasoning
cognitive control
intelligence
response inhibition
planning
rule
deception
task switching
goal
memory
episodic memory
retrieval
decision
working memory
judgment
maintenance
response selection
belief
familiarity
intention
expectancy
default mode network
context
semantic memory
executive function
navigation
narrative
theory of mind
executive control
autobiographical memory
concept
phonological processing
thought
semantic processing
remembering
attention
social cognition
inference
meaning
decision making
comprehension
mental rotation
consciousness
metaphor
memory encoding
sentence comprehension
short term memory
choice
emotion
recall
humor
search
impulsivity
visual search
mental imagery
reward
emotion regulation
language processing
language
reading
selective attention
spatial attention
risk
eating
object recognition
arousal
empathy
face perception
loss
sustained attention
spatial working memory
fear
consolidation
rehearsal
naming
stress
action
syntax
anticipation
learning
gaze
communication
induction
sleep
listening
visual attention
extinction
prosody
integration
associative memory
anxiety
skill
motor imagery
emotional expression
speech perception
movement
habit
facial expression
speech production
retention
sequence learning
pain
melody
motor control
motor learning

decision making

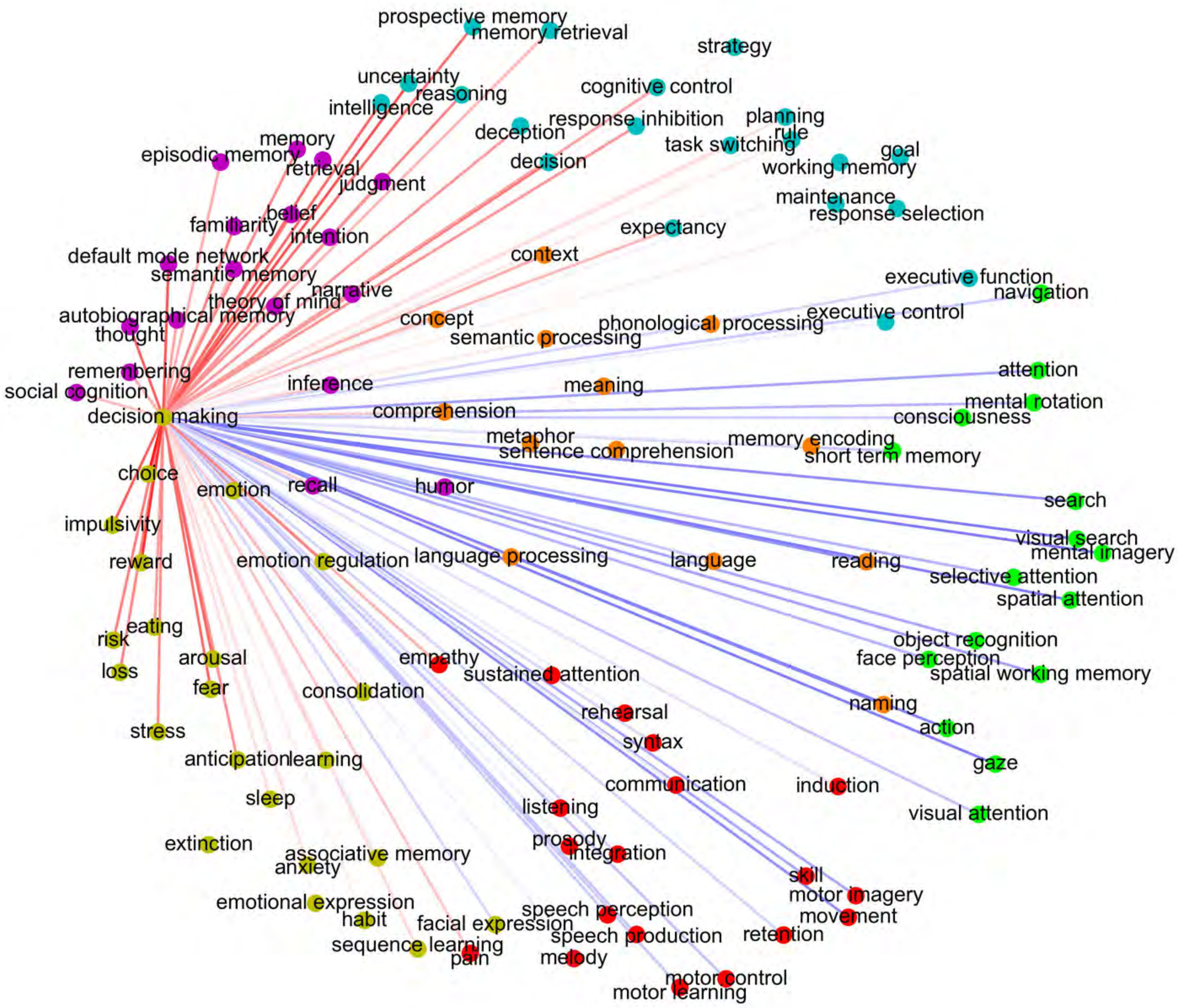

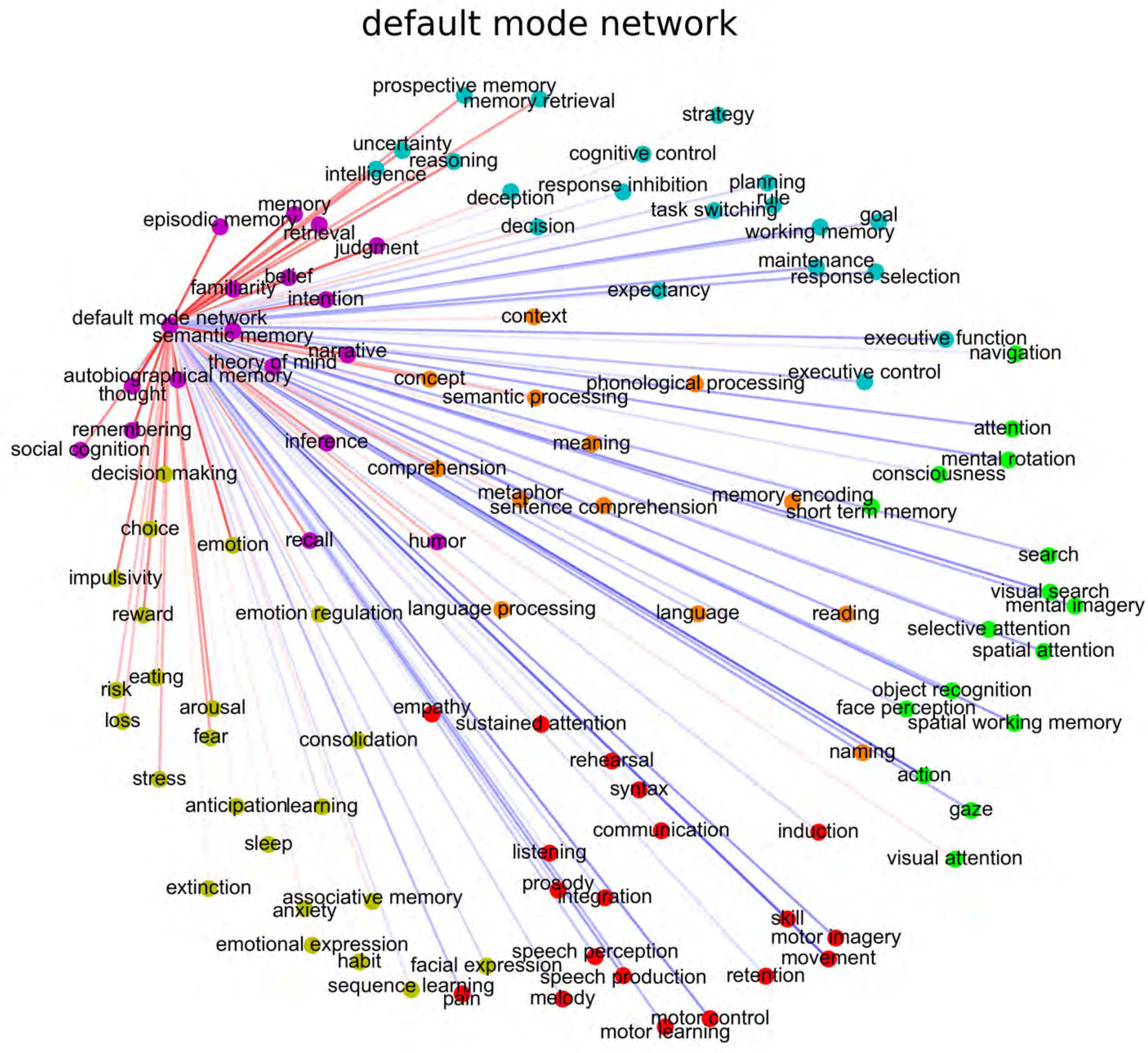
default mode network
prospective memory
memory retrieval
strategy
uncertainty
reasoning
cognitive control
intelligence
planning
response inhibition
deception
rule
task switching
goal
memory
episodic memory
retrieval
decision
working memory
judgment
maintenance
response selection
belief
familiarity
intention
expectancy
default mode network
context
semantic memory
executive function
navigation
narrative
theory of mind
executive control
autobiographical memory
concept
phonological processing
thought
semantic processing
attention
remembering
social cognition
inference
meaning
mental rotation
consciousness
decision making
comprehension
metaphor
memory encoding
sentence comprehension
short term memory
choice
emotion
recall
humor
search
impulsivity
visual search
mental imagery
reward
emotion regulation
language processing
language
reading
selective attention
spatial attention
eating
risk
arousal
object recognition
empathy
face perception
loss
sustained attention
spatial working memory
fear
consolidation
rehearsal
naming
stress
syntax
action
anticipation
learning
gaze
communication
induction
sleep
listening
visual attention
extinction
prosody
integration
associative memory
anxiety
skill
motor imagery
emotional expression
speech perception
habit
movement
facial expression
speech production
retention
sequence learning
pain
melody
motor control
motor learning

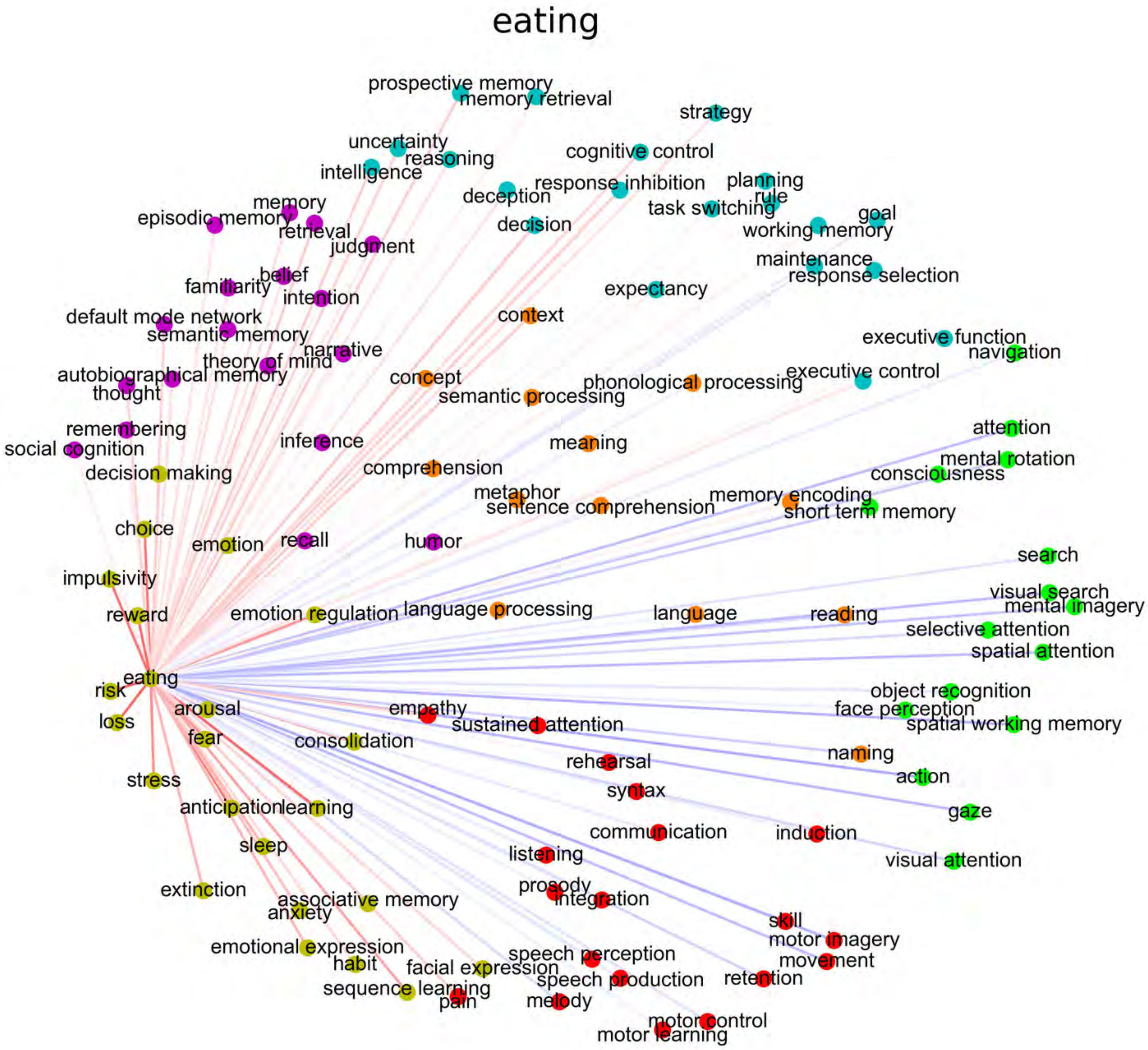
eating
prospective memory
memory retrieval
strategy
uncertainty
reasoning
cognitive control
intelligence
planning
response inhibition
deception
rule
memory
task switching
goal
episodic memory
retrieval
decision
working memory
judgment
maintenance
response selection
belief
familiarity
intention
expectancy
default mode network
context
semantic memory
executive function
navigation
narrative
theory of mind
concept
executive control
autobiographical memory
phonological processing
thought
semantic processing
remembering
attention
inference
meaning
social cognition
mental rotation
decision making
comprehension
consciousness
metaphor
memory encoding
sentence comprehension
short term memory
choice
emotion
recall
humor
search
impulsivity
visual search
mental imagery
reward
emotion regulation
language processing
language
reading
selective attention
spatial attention
eating
risk
object recognition
arousal
empathy
face perception
loss
sustained attention
spatial working memory
fear
consolidation
naming
rehearsal
action
stress
syntax
anticipation
learning
gaze
communication
induction
sleep
listening
visual attention
extinction
prosody
integration
associative memory
anxiety
skill
motor imagery
emotional expression
speech perception
movement
habit
facial expression
speech production
retention
sequence learning
pain
melody
motor control
motor learning

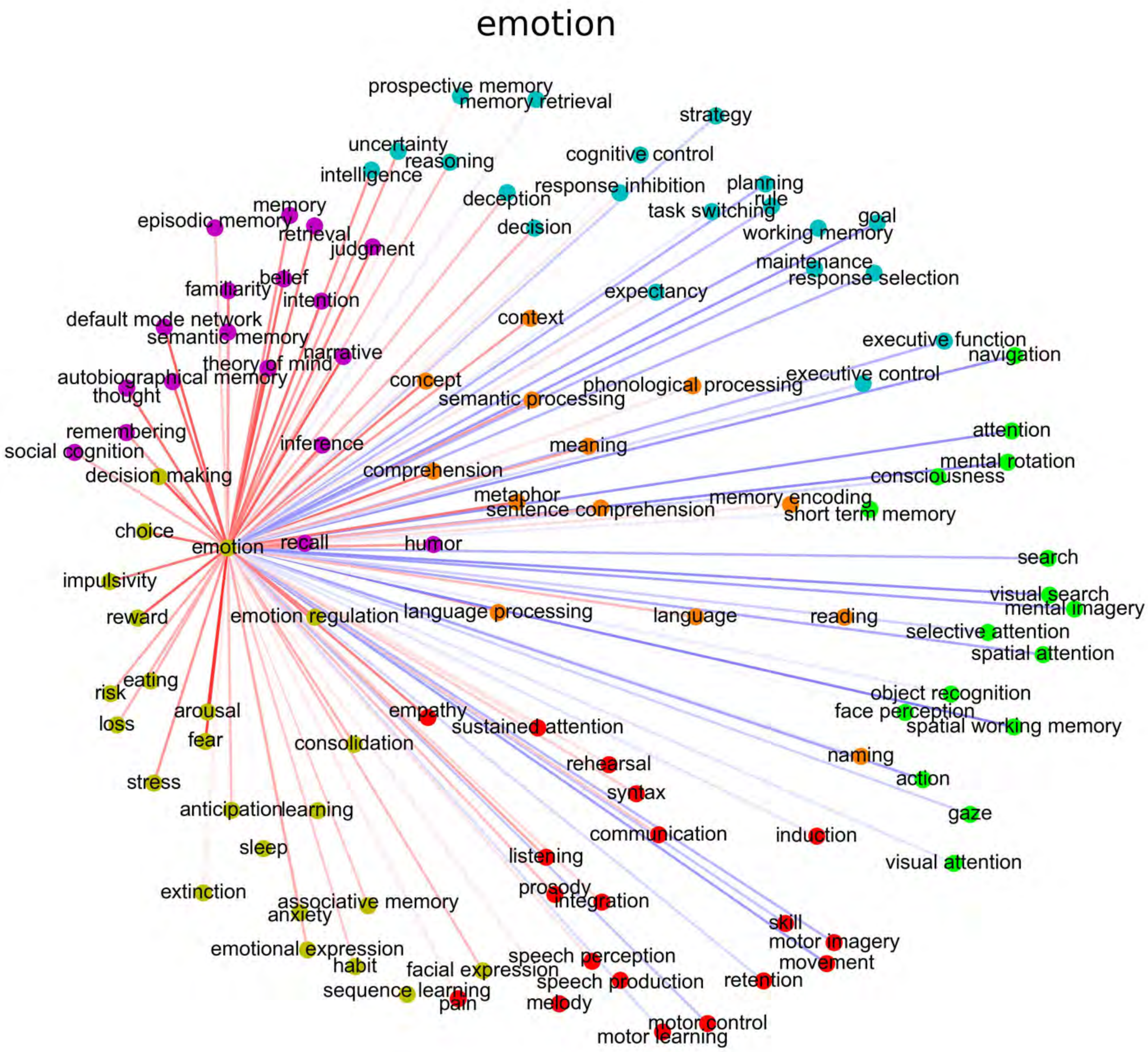
emotion
prospective memory
memory retrieval
strategy
uncertainty
reasoning
cognitive control
intelligence
deception
response inhibition
planning
rule
task switching
goal
working memory
memory
episodic memory
retrieval
decision
judgment
maintenance
response selection
familiarity
belief
intention
expectancy
context
default mode network
semantic memory
executive function
navigation
narrative
theory of mind
concept
executive control
autobiographical memory
thought
phonological processing
semantic processing
remembering
social cognition
inference
meaning
attention
decision making
comprehension
mental rotation
consciousness
metaphor
sentence comprehension
memory encoding
short term memory
choice
emotion
recall
humor
search
impulsivity
visual search
mental imagery
reward
emotion regulation
language processing
language
reading
selective attention
spatial attention
risk
eating
object recognition
arousal
empathy
face perception
loss
sustained attention
spatial working memory
fear
consolidation
rehearsal
naming
action
stress
syntax
anticipation
learning
gaze
communication
induction
sleep
listening
visual attention
extinction
prosody
integration
associative memory
anxiety
skill
motor imagery
emotional expression
movement
habit
speech perception
facial expression
speech production
retention
sequence learning
pain
melody
motor control
motor learning

emotion regulation

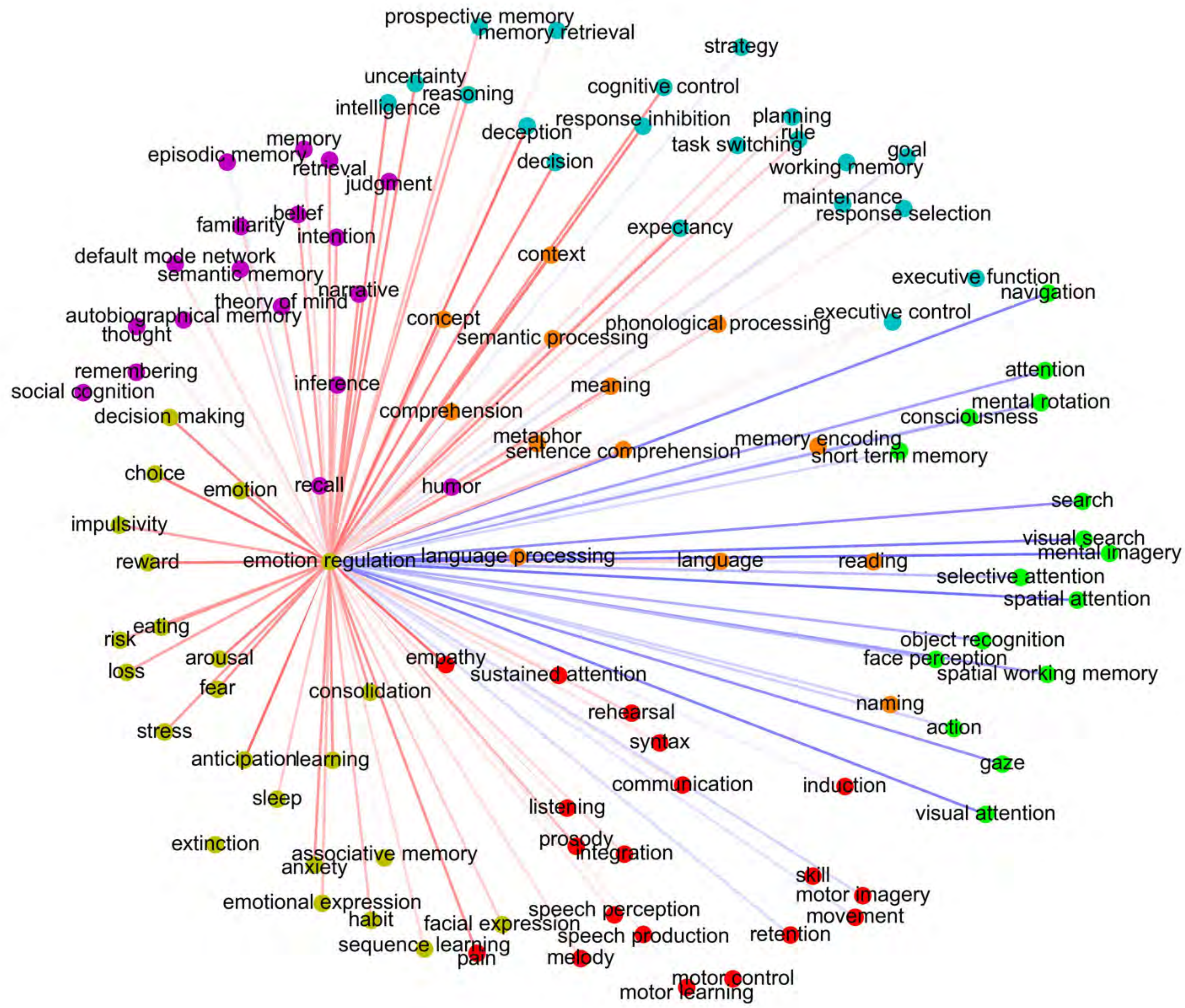

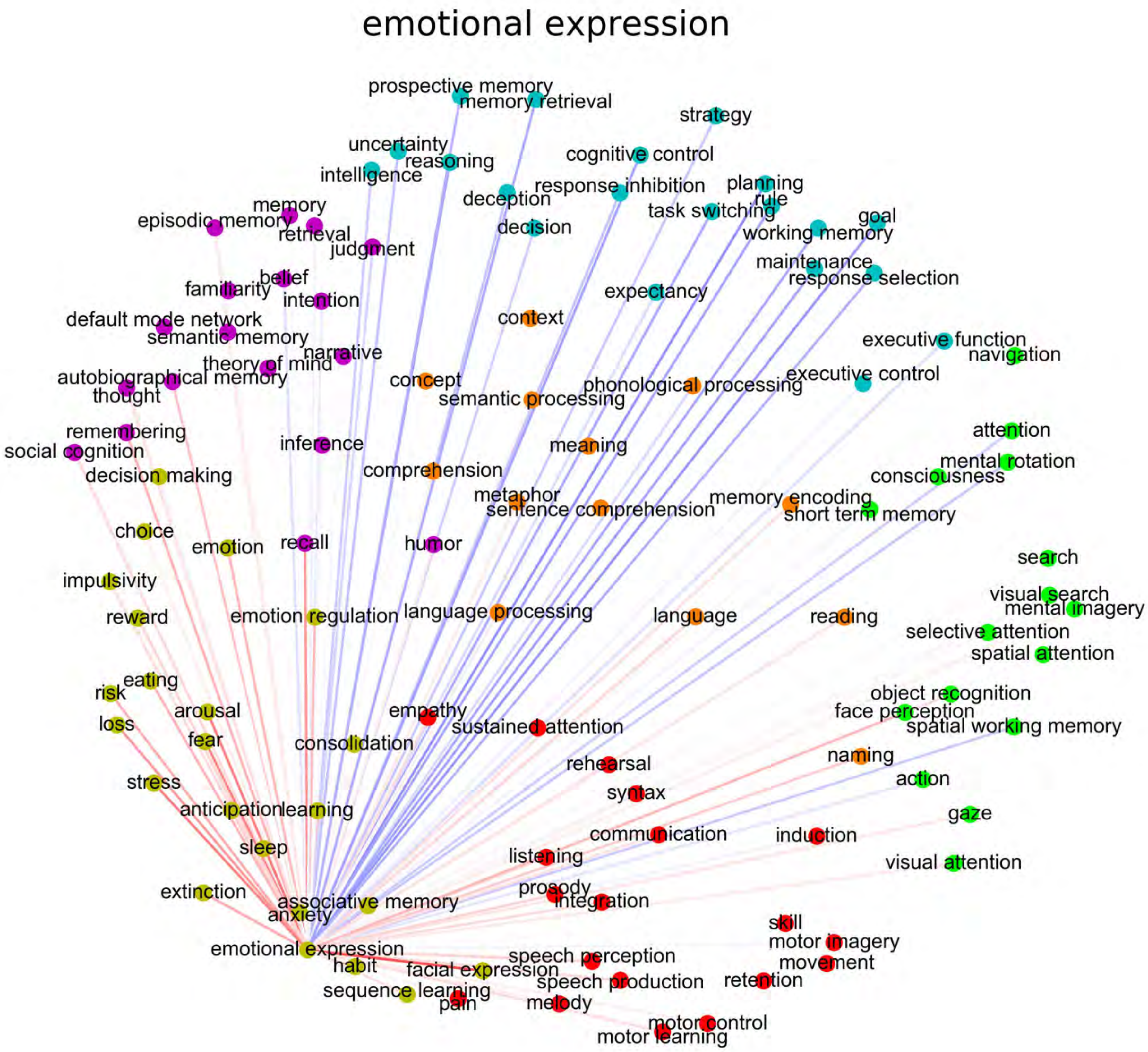

emotional expression
prospective memory
memory retrieval
strategy
uncertainty
reasoning
cognitive control
intelligence
response inhibition
planning
deception
rule
memory
task switching
episodic memory
goal
retrieval
decision
working memory
judgment
maintenance
belief
response selection
familiarity
intention
expectancy
default mode network
context
semantic memory
executive function
navigation
narrative
theory of mind
concept
executive control
autobiographical memory
phonological processing
thought
semantic processing
remembering
attention
inference
meaning
social cognition
mental rotation
decision making
consciousness
comprehension
metaphor
memory encoding
sentence comprehension
short term memory
choice
emotion
recall
humor
search
impulsivity
visual search
mental imagery
reward
emotion regulation
language processing
language
reading
selective attention
spatial attention
eating
risk
object recognition
arousal
empathy
face perception
loss
sustained attention
spatial working memory
fear
consolidation
rehearsal
naming
stress
action
syntax
anticipation
learning
gaze
communication
induction
sleep
listening
visual attention
extinction
prosody
integration
associative memory
anxiety
skill
motor imagery
emotional expression
speech perception
movement
habit
facial expression
speech production
retention
sequence learning
pain
melody
motor control
motor learning

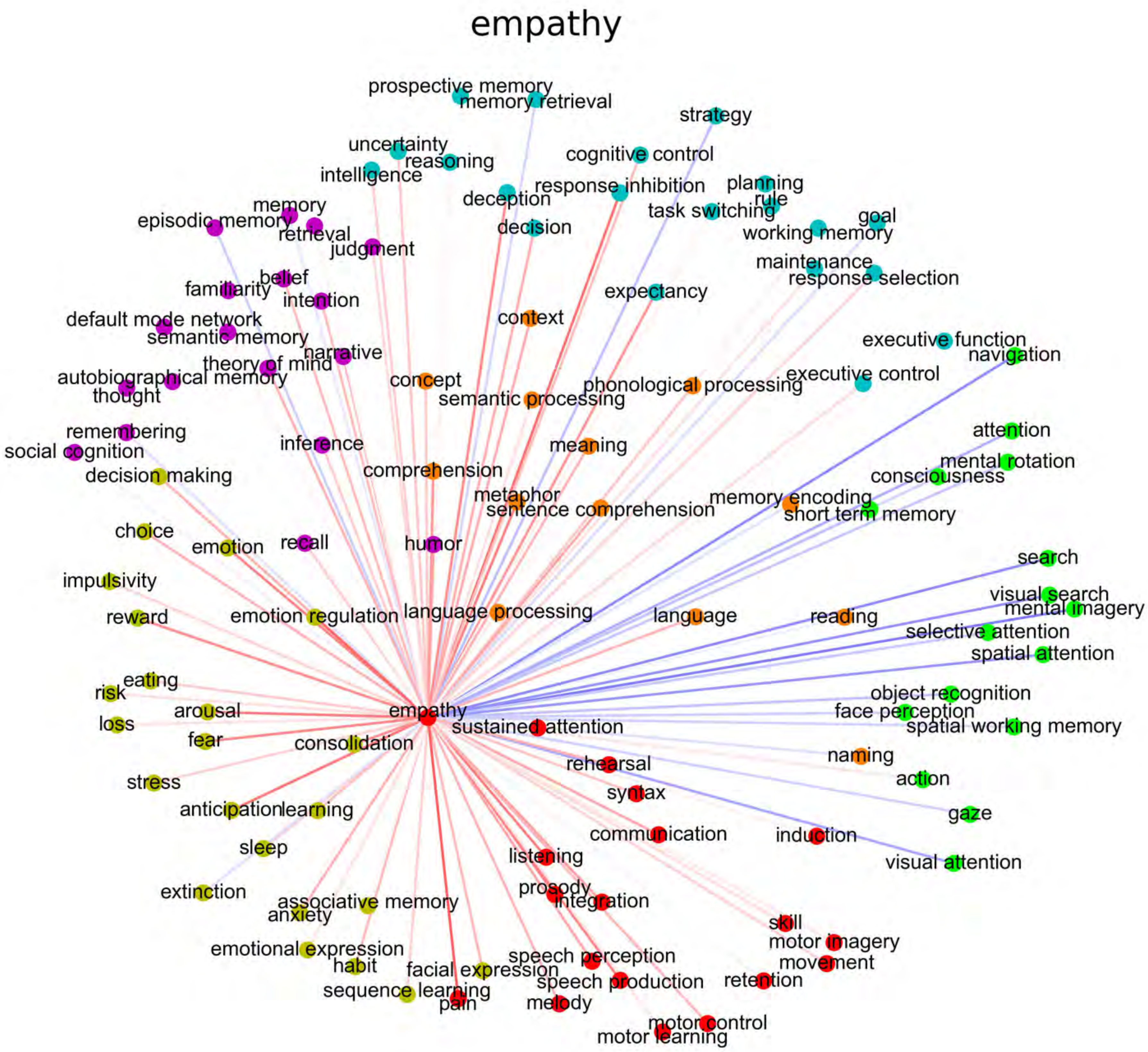
empathy
prospective memory
memory retrieval
strategy
uncertainty
reasoning
cognitive control
intelligence
planning
rule
deception
response inhibition
task switching
memory
episodic memory
retrieval
decision
goal
working memory
judgment
maintenance
response selection
belief
familiarity
intention
expectancy
default mode network
semantic memory
context
executive function
navigation
narrative
theory of mind
autobiographical memory
thought
concept
semantic processing
phonological processing
executive control
remembering
social cognition
inference
meaning
attention
mental rotation
consciousness
decision making
comprehension
metaphor
sentence comprehension
memory encoding
short term memory
choice
emotion
recall
humor
search
impulsivity
visual search
mental imagery
reward
emotion regulation
language processing
language
reading
selective attention
spatial attention
eating
risk
arousal
loss
fear
consolidation
empathy
sustained attention
object recognition
face perception
spatial working memory
naming
rehearsal
action
stress
syntax
anticipation
learning
gaze
communication
induction
sleep
listening
visual attention
extinction
prosody
integration
associative memory
anxiety
skill
motor imagery
emotional expression
habit
movement
facial expression
speech perception
speech production
retention
sequence learning
pain
melody
motor control
motor learning

episodic memory

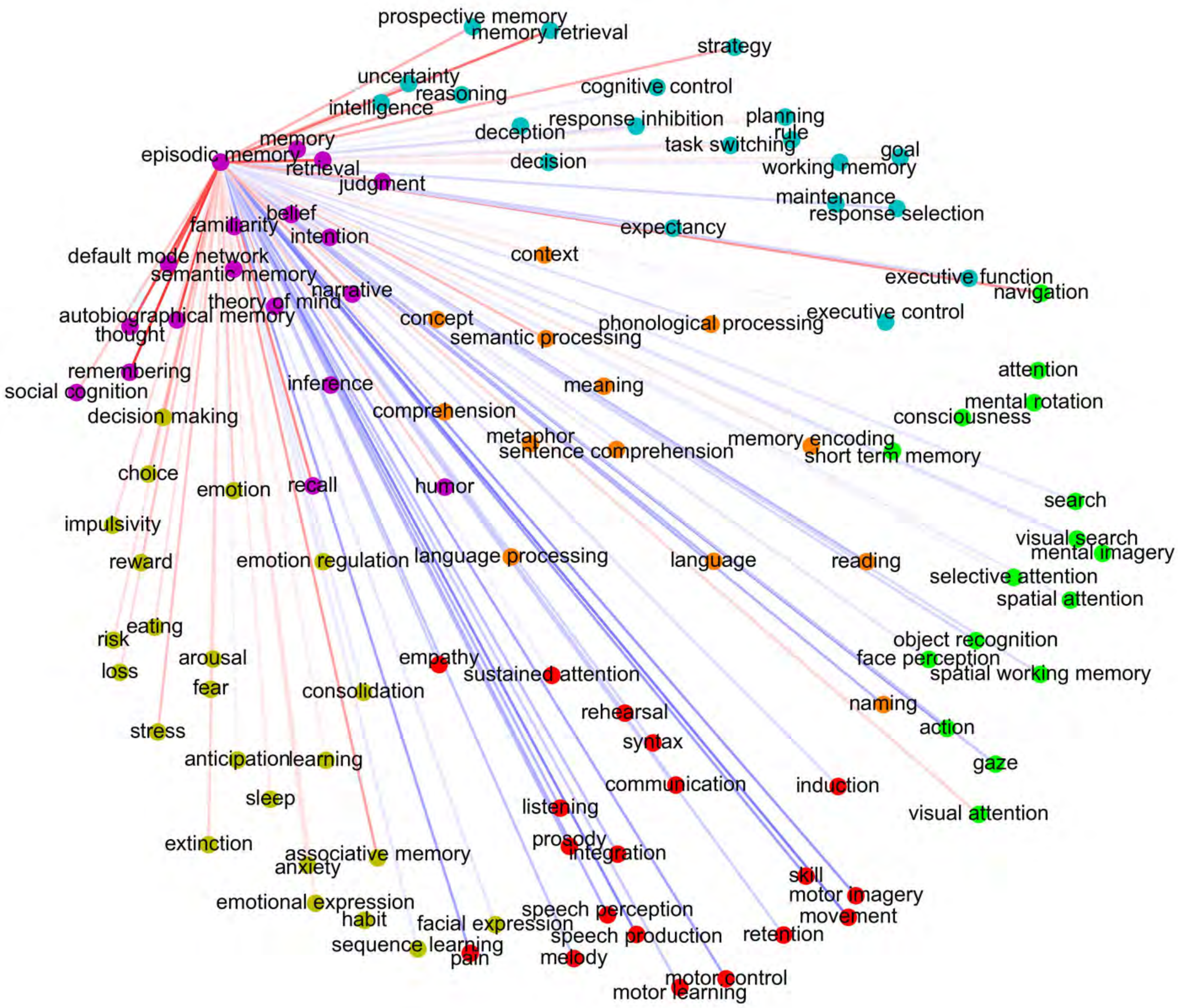

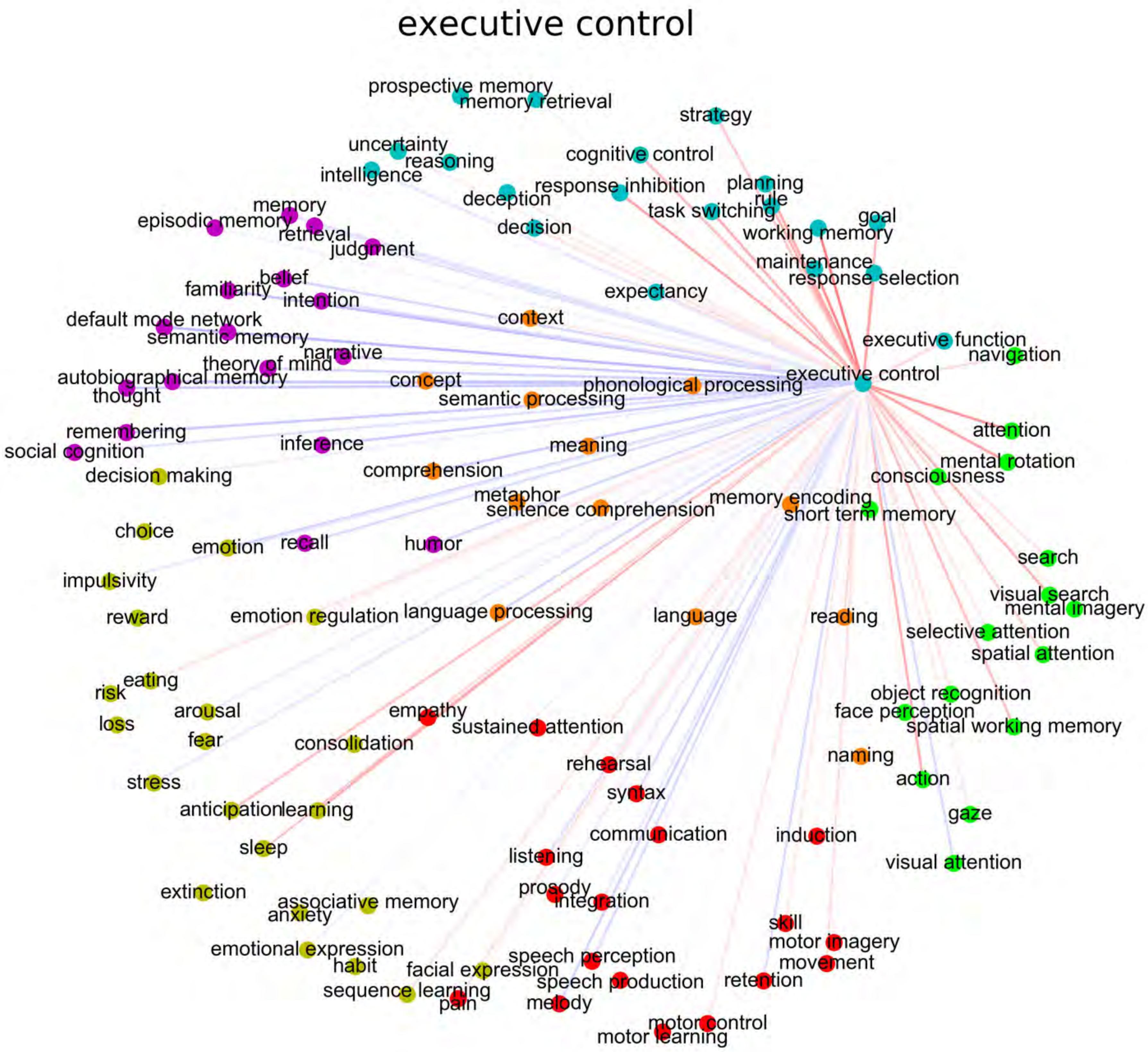
executive control
prospective memory
memory retrieval
strategy
uncertainty
reasoning
cognitive control
intelligence
response inhibition
planning
deception
rule
task switching
goal
memory
episodic memory
retrieval
decision
working memory
judgment
maintenance
response selection
belief
familiarity
intention
expectancy
default mode network
context
semantic memory
executive function
navigation
narrative
theory of mind
executive control
autobiographical memory
concept
phonological processing
thought
semantic processing
attention
remembering
inference
meaning
social cognition
mental rotation
decision making
comprehension
consciousness
metaphor
memory encoding
sentence comprehension
short term memory
choice
emotion
recall
humor
search
impulsivity
visual search
mental imagery
reward
emotion regulation
language processing
language
reading
selective attention
spatial attention
eating
risk
object recognition
arousal
empathy
face perception
loss
sustained attention
spatial working memory
fear
consolidation
naming
rehearsal
stress
action
syntax
anticipation
learning
gaze
communication
induction
sleep
listening
visual attention
extinction
prosody
associative memory
integration
anxiety
skill
motor imagery
emotional expression
speech perception
movement
habit
facial expression
speech production
retention
sequence learning
pain
melody
motor control
motor learning

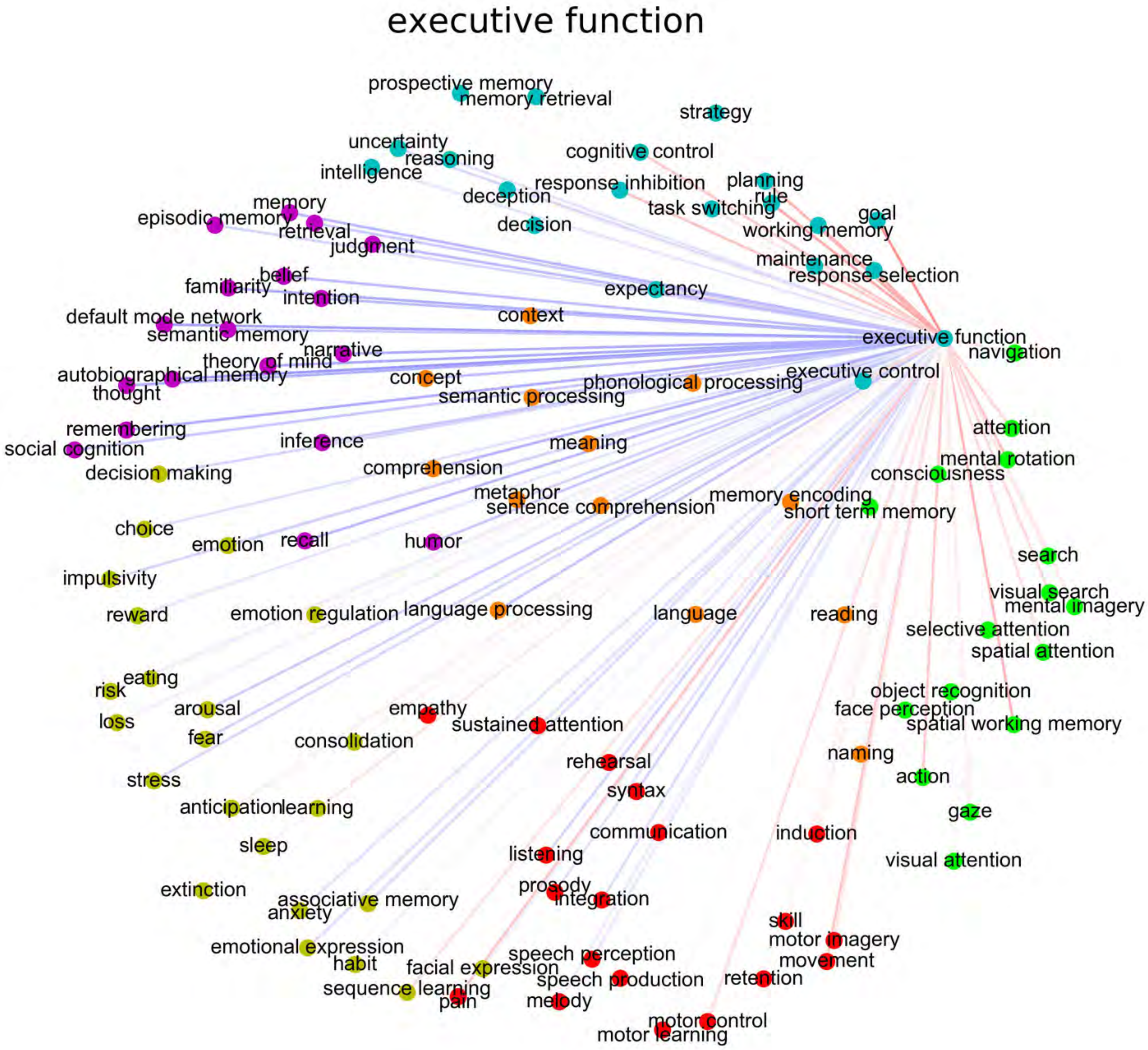
executive function
prospective memory
memory retrieval
strategy
uncertainty
reasoning
intelligence
cognitive control
response inhibition
planning
rule
deception
task switching
goal
memory
episodic memory
retrieval
decision
working memory
judgment
maintenance
response selection
belief
familiarity
intention
expectancy
default mode network
semantic memory
context
executive function
navigation
narrative
theory of mind
autobiographical memory
concept
phonological processing
executive control
thought
semantic processing
remembering
social cognition
inference
meaning
attention
decision making
comprehension
mental rotation
consciousness
metaphor
sentence comprehension
memory encoding
short term memory
choice
emotion
recall
humor
impulsivity
search
visual search
mental imagery
reward
emotion regulation
language processing
language
reading
selective attention
spatial attention
eating
risk
arousal
loss
empathy
sustained attention
object recognition
face perception
spatial working memory
fear
consolidation
rehearsal
naming
stress
syntax
action
anticipation
learning
gaze
communication
induction
sleep
listening
visual attention
extinction
prosody
integration
associative memory
anxiety
skill
motor imagery
emotional expression
habit
speech perception
facial expression
movement
speech production
retention
sequence learning
pain
melody
motor control
motor learning

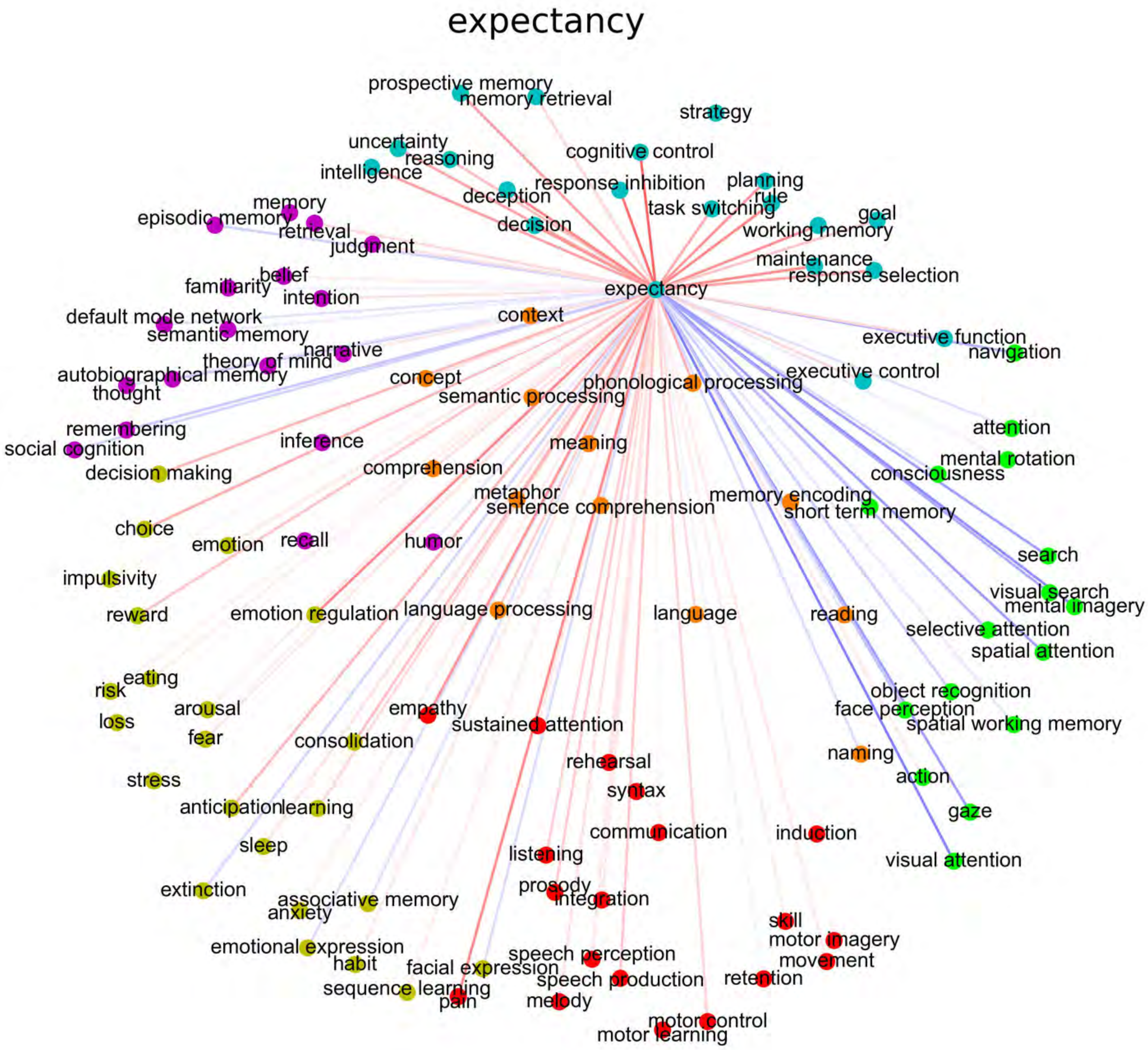
expectancy
prospective memory
memory retrieval
strategy
uncertainty
reasoning
cognitive control
intelligence
response inhibition
planning
deception
rule
memory
task switching
goal
episodic memory
retrieval
decision
working memory
judgment
maintenance
response selection
belief
familiarity
intention
expectancy
context
default mode network
semantic memory
executive function
navigation
narrative
theory of mind
executive control
autobiographical memory
concept
phonological processing
thought
semantic processing
attention
remembering
inference
meaning
social cognition
mental rotation
decision making
comprehension
consciousness
metaphor
memory encoding
sentence comprehension
short term memory
choice
emotion
recall
humor
search
impulsivity
visual search
mental imagery
reward
emotion regulation
language processing
language
reading
selective attention
spatial attention
eating
risk
object recognition
arousal
empathy
face perception
loss
sustained attention
spatial working memory
fear
consolidation
rehearsal
naming
stress
action
syntax
anticipation
learning
gaze
communication
induction
sleep
listening
visual attention
extinction
prosody
integration
associative memory
anxiety
skill
motor imagery
emotional expression
speech perception
movement
habit
facial expression
speech production
retention
sequence learning
pain
melody
motor control
motor learning

extinction

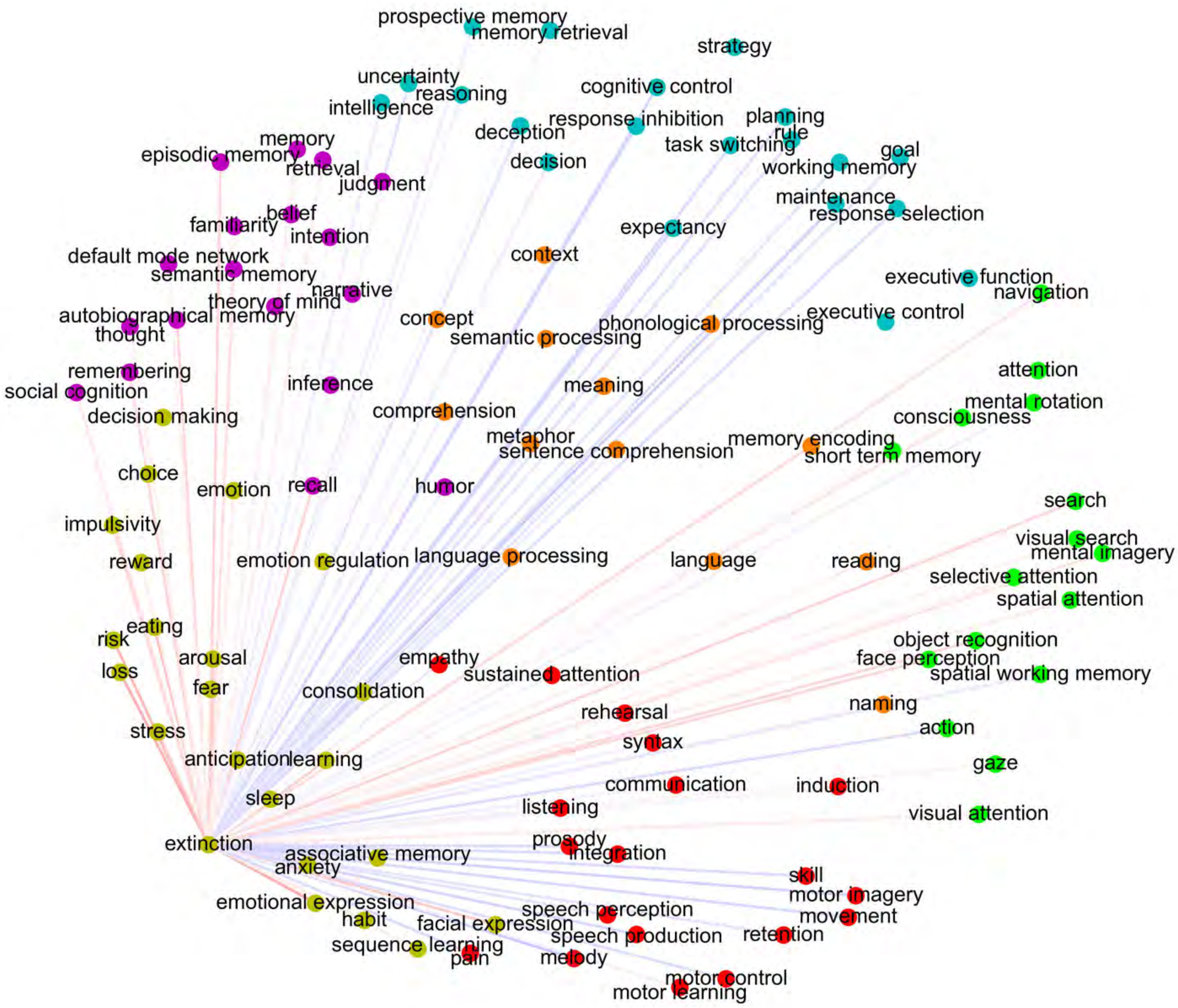
prospective memory
memory retrieval
strategy
uncertainty
reasoning
cognitive control
intelligence
planning
response inhibition
deception
rule
task switching
goal
memory
episodic memory
retrieval
decision
working memory
judgment
maintenance
response selection
belief
familiarity
intention
expectancy
context
default mode network
semantic memory
executive function
navigation
narrative
theory of mind
concept
phonological processing
executive control
autobiographical memory
thought
semantic processing
attention
remembering
inference
meaning
social cognition
mental rotation
comprehension
consciousness
decision making
metaphor
memory encoding
sentence comprehension
short term memory
choice
emotion
recall
humor
search
impulsivity
visual search
mental imagery
reward
emotion regulation
language processing
language
reading
selective attention
spatial attention
eating
risk
object recognition
arousal
empathy
face perception
loss
sustained attention
spatial working memory
fear
consolidation
rehearsal
naming
stress
syntax
action
anticipation
learning
gaze
communication
induction
sleep
listening
visual attention
extinction
prosody
integration
associative memory
anxiety
skill
motor imagery
emotional expression
speech perception
movement
habit
facial expression
speech production
retention
sequence learning
pain
melody
motor control
motor learning

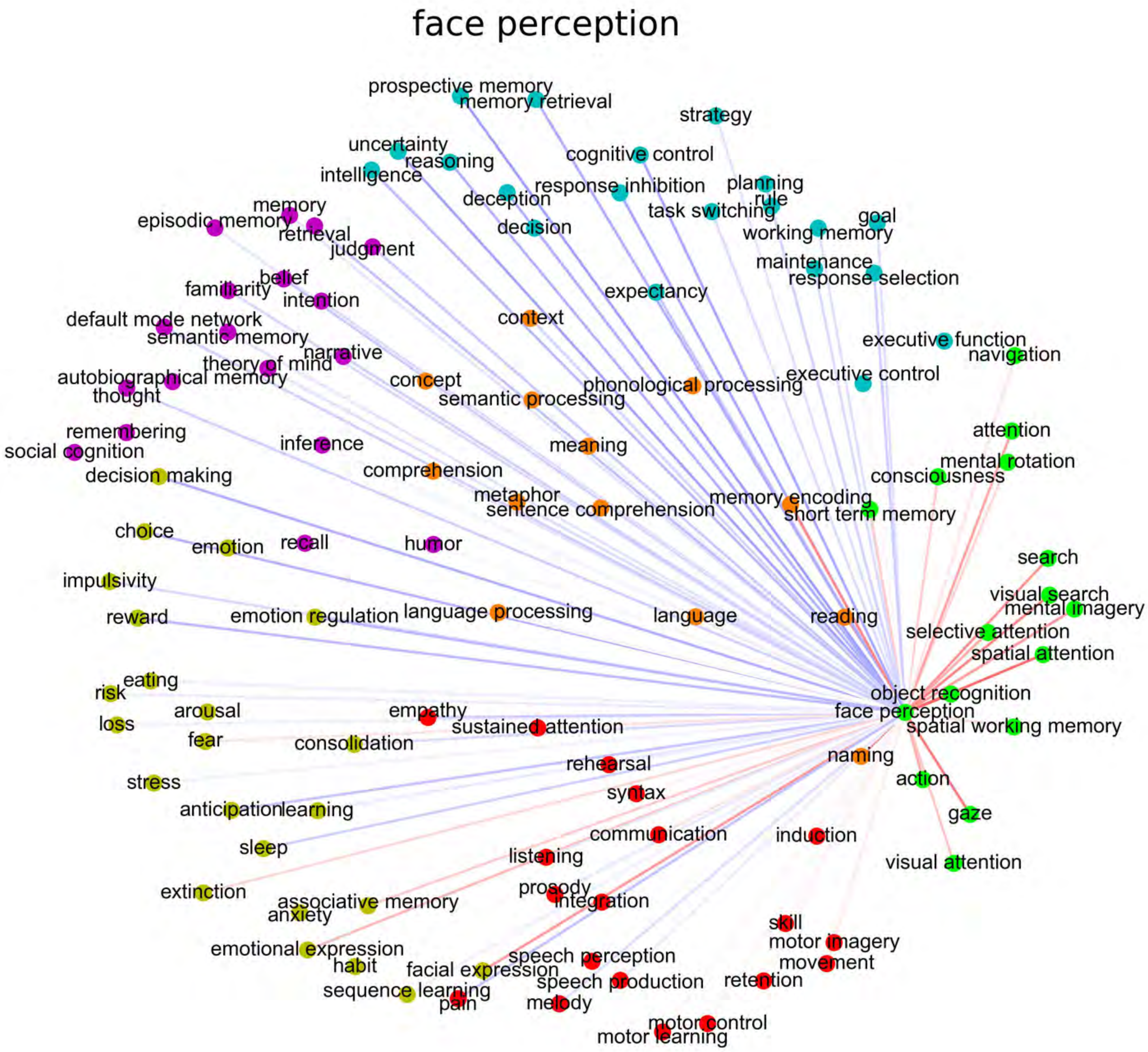
face perception
prospective memory
memory retrieval
strategy
uncertainty
reasoning
cognitive control
intelligence
planning
response inhibition
deception
rule
task switching
goal
memory
episodic memory
retrieval
decision
working memory
judgment
maintenance
response selection
belief
familiarity
intention
expectancy
context
default mode network
semantic memory
executive function
navigation
narrative
theory of mind
executive control
autobiographical memory
concept
phonological processing
thought
semantic processing
remembering
attention
inference
meaning
social cognition
mental rotation
decision making
comprehension
consciousness
metaphor
memory encoding
sentence comprehension
short term memory
choice
emotion
recall
humor
search
impulsivity
visual search
mental imagery
reward
emotion regulation
language processing
language
reading
selective attention
spatial attention
eating
risk
object recognition
arousal
empathy
face perception
loss
sustained attention
spatial working memory
fear
consolidation
naming
rehearsal
stress
action
syntax
anticipation
learning
gaze
communication
induction
sleep
listening
visual attention
extinction
prosody
integration
associative memory
anxiety
skill
motor imagery
emotional expression
habit
speech perception
movement
facial expression
speech production
retention
sequence learning
pain
melody
motor control
motor learning

facial expression

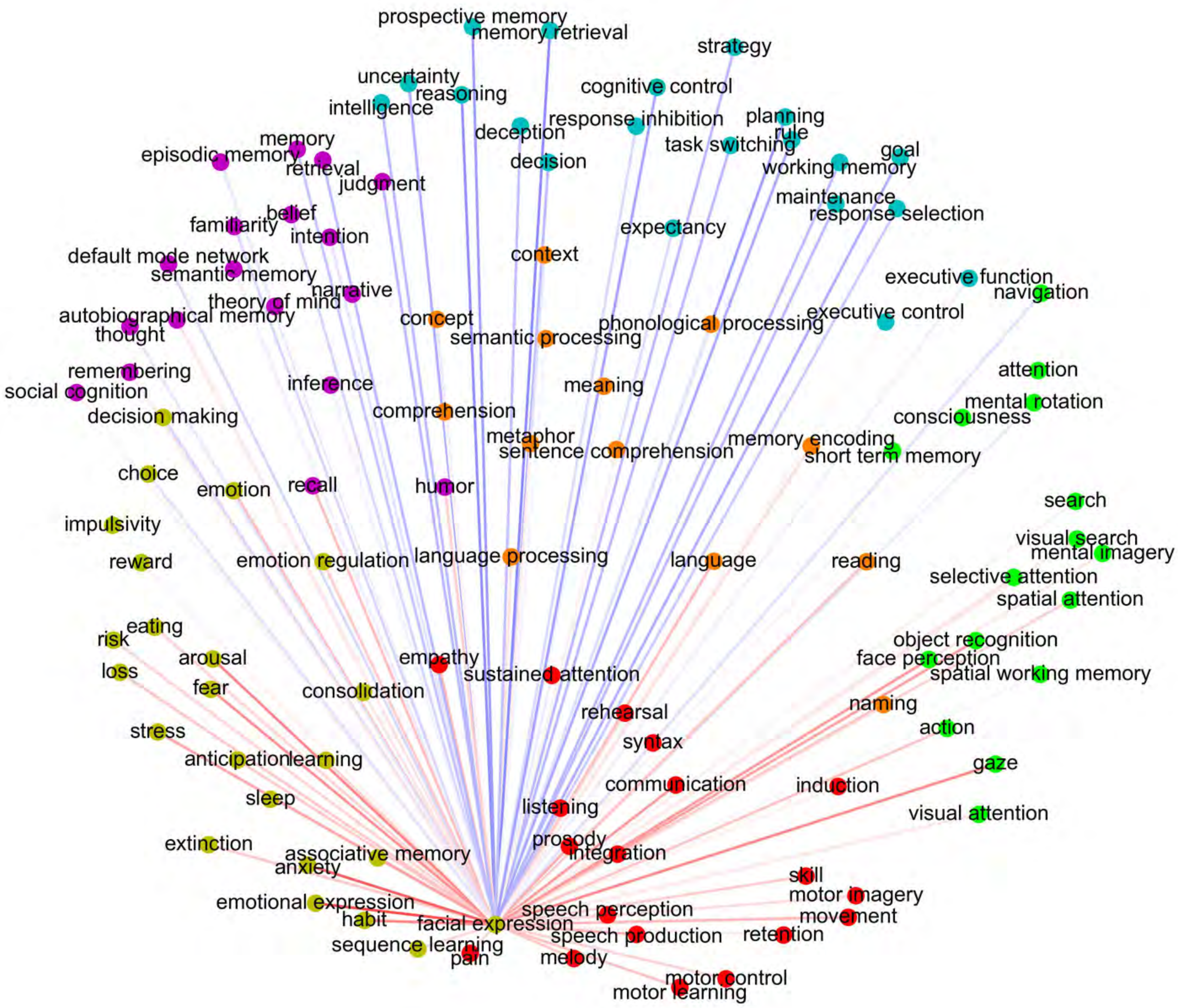

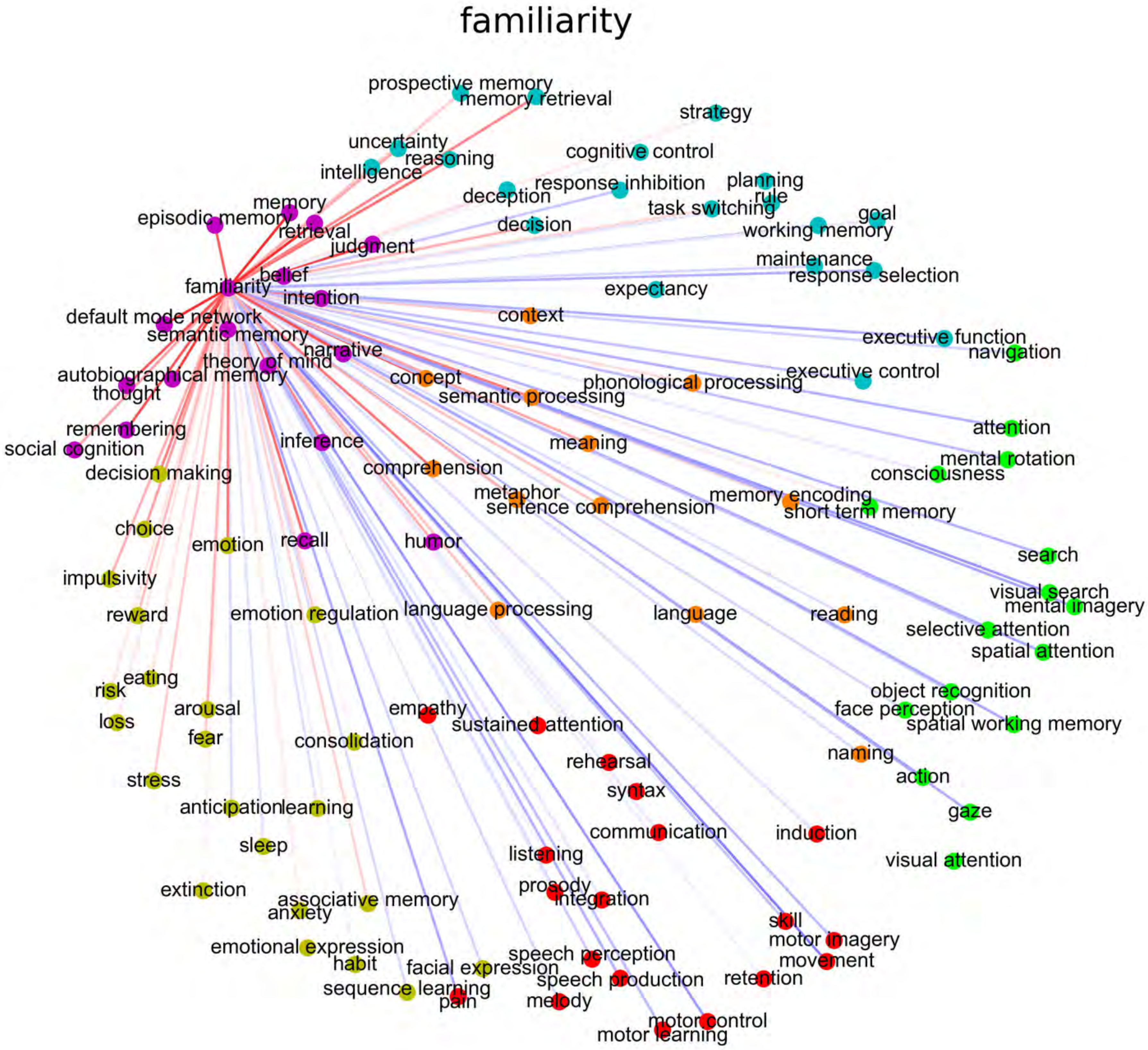
familiarity
prospective memory
memory retrieval
strategy
uncertainty
reasoning
cognitive control
intelligence
deception
response inhibition
planning
rule
memory
episodic memory
retrieval
task switching
goal
decision
working memory
judgment
maintenance
belief
response selection
familiarity
expectancy
intention
default mode network
context
semantic memory
executive function
navigation
narrative
theory of mind
executive control
autobiographical memory
concept
phonological processing
thought
semantic processing
remembering
attention
social cognition
inference
meaning
mental rotation
decision making
comprehension
consciousness
metaphor
memory encoding
sentence comprehension
short term memory
choice
emotion
recall
humor
search
impulsivity
visual search
mental imagery
reward
emotion regulation
language processing
language
reading
selective attention
spatial attention
risk
eating
object recognition
arousal
face perception
loss
empathy
sustained attention
spatial working memory
fear
consolidation
rehearsal
naming
stress
action
syntax
anticipation
learning
gaze
communication
induction
sleep
listening
visual attention
extinction
prosody
integration
associative memory
anxiety
skill
motor imagery
emotional expression
habit
speech perception
movement
facial expression
speech production
retention
sequence learning
pain
melody
motor control
motor learning

fear

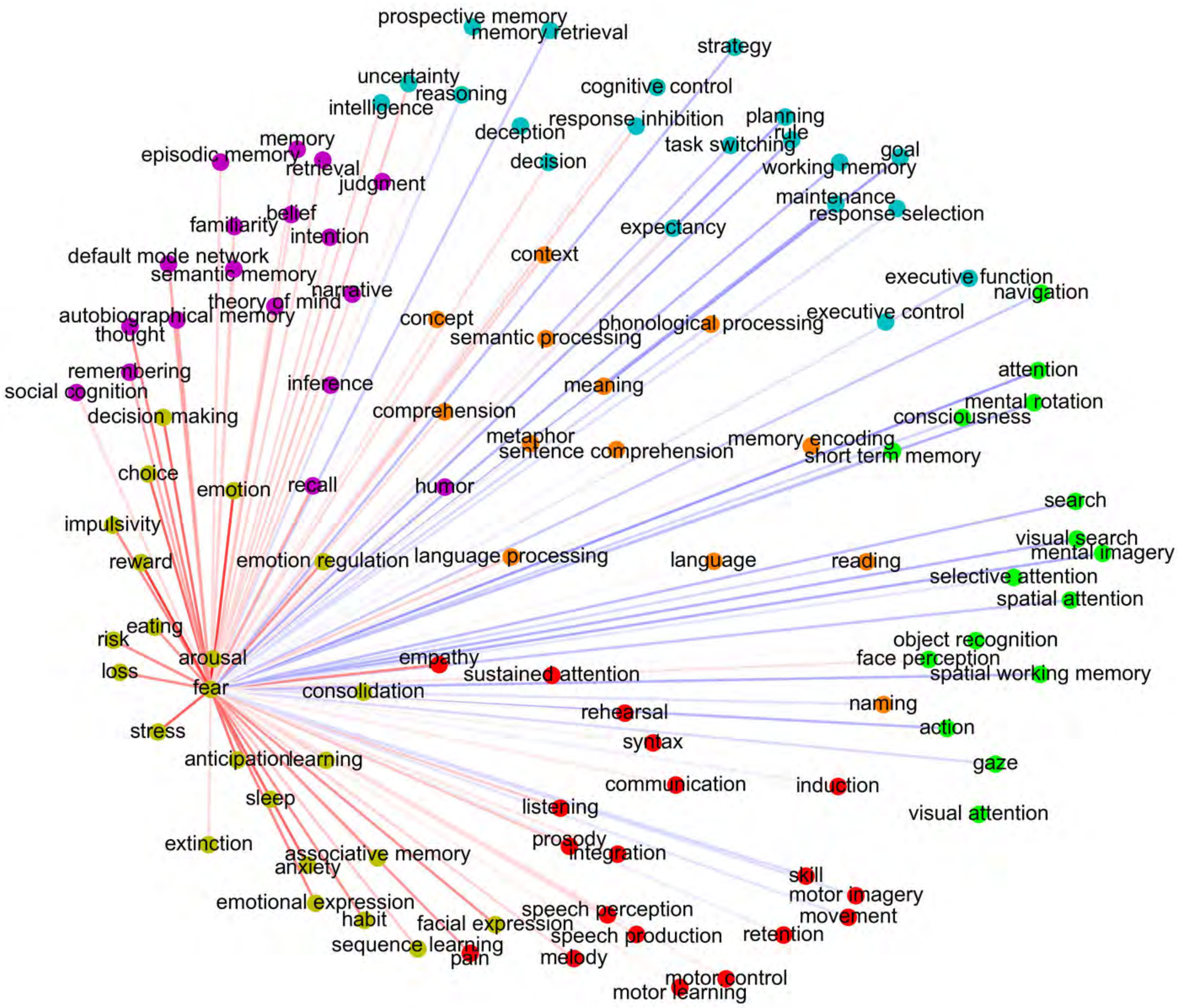
prospective memory
memory retrieval
strategy
uncertainty
reasoning
cognitive control
intelligence
response inhibition
planning
rule
deception
task switching
memory
episodic memory
retrieval
decision
goal
working memory
judgment
maintenance
response selection
belief
familiarity
intention
expectancy
default mode network
context
semantic memory
executive function
navigation
narrative
theory of mind
concept
executive control
autobiographical memory
phonological processing
thought
semantic processing
remembering
attention
social cognition
inference
meaning
mental rotation
decision making
comprehension
consciousness
metaphor
memory encoding
sentence comprehension
short term memory
choice
emotion
recall
humor
search
impulsivity
visual search
mental imagery
reward
emotion regulation
language processing
language
reading
selective attention
spatial attention
eating
risk
object recognition
arousal
empathy
face perception
loss
sustained attention
spatial working memory
fear
consolidation
naming
rehearsal
stress
action
syntax
anticipation
learning
gaze
communication
induction
sleep
listening
visual attention
extinction
prosody
integration
associative memory
anxiety
skill
motor imagery
emotional expression
speech perception
movement
habit
facial expression
speech production
retention
sequence learning
pain
melody
motor control
motor learning

gaze

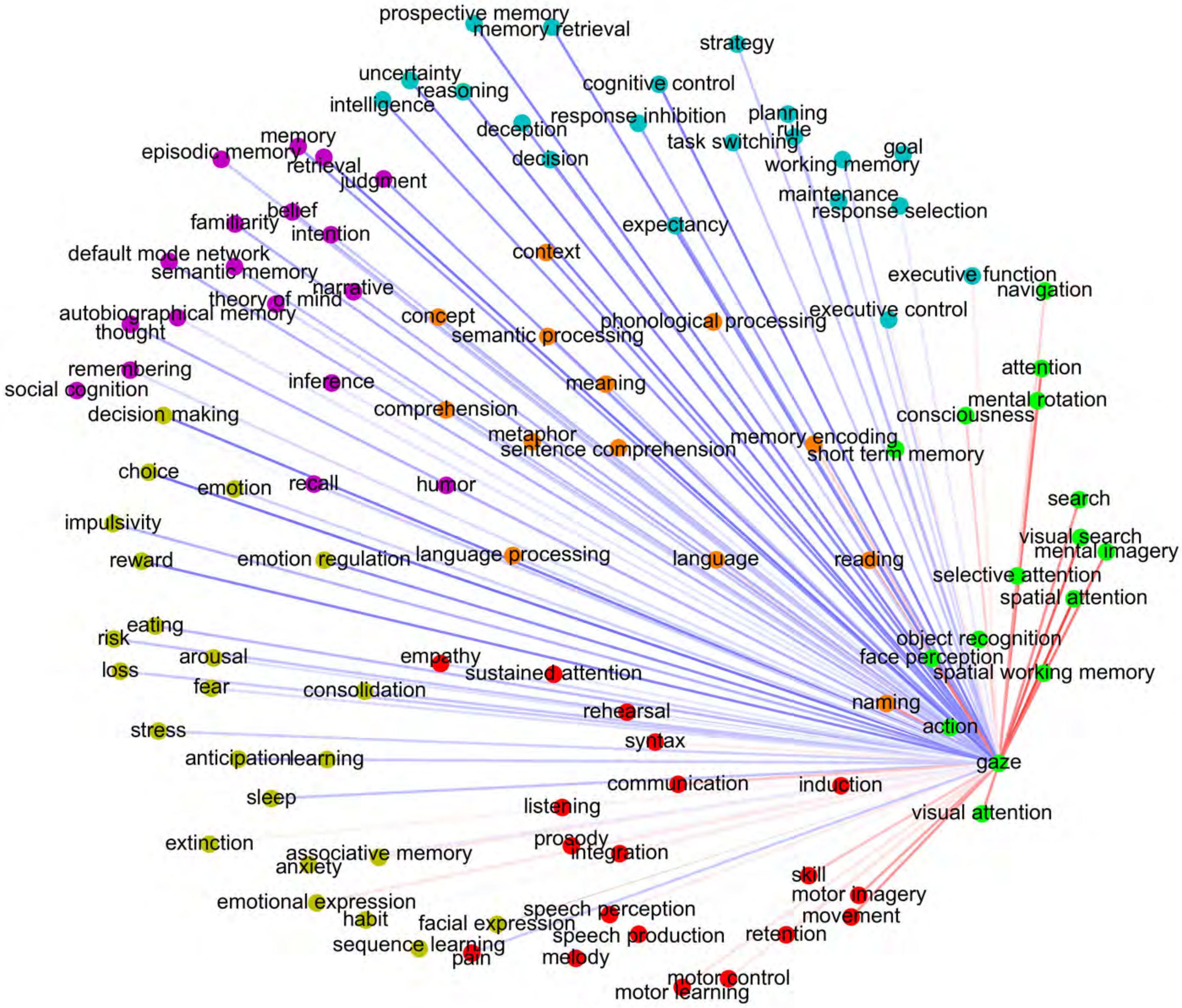

prospective memory
memory retrieval
strategy
uncertainty
reasoning
cognitive control
intelligence
response inhibition
planning
deception
rule
task switching
goal
memory
episodic memory
retrieval
decision
working memory
judgment
maintenance
response selection
belief
familiarity
intention
expectancy
default mode network
context
semantic memory
executive function
navigation
narrative
theory of mind
concept
phonological processing
executive control
autobiographical memory
thought
semantic processing
attention
remembering
inference
meaning
social cognition
mental rotation
decision making
comprehension
consciousness
metaphor
memory encoding
sentence comprehension
short term memory
choice
recall
humor
emotion
search
impulsivity
visual search
mental imagery
reward
emotion regulation
language processing
language
reading
selective attention
spatial attention
eating
risk
object recognition
arousal
empathy
face perception
loss
sustained attention
spatial working memory
fear
consolidation
rehearsal
naming
action
stress
syntax
anticipation
learning
gaze
communication
induction
sleep
listening
visual attention
extinction
prosody
associative memory
integration
anxiety
skill
motor imagery
emotional expression
movement
habit
speech perception
facial expression
speech production
retention
sequence learning
pain
melody
motor control
motor learning

goal

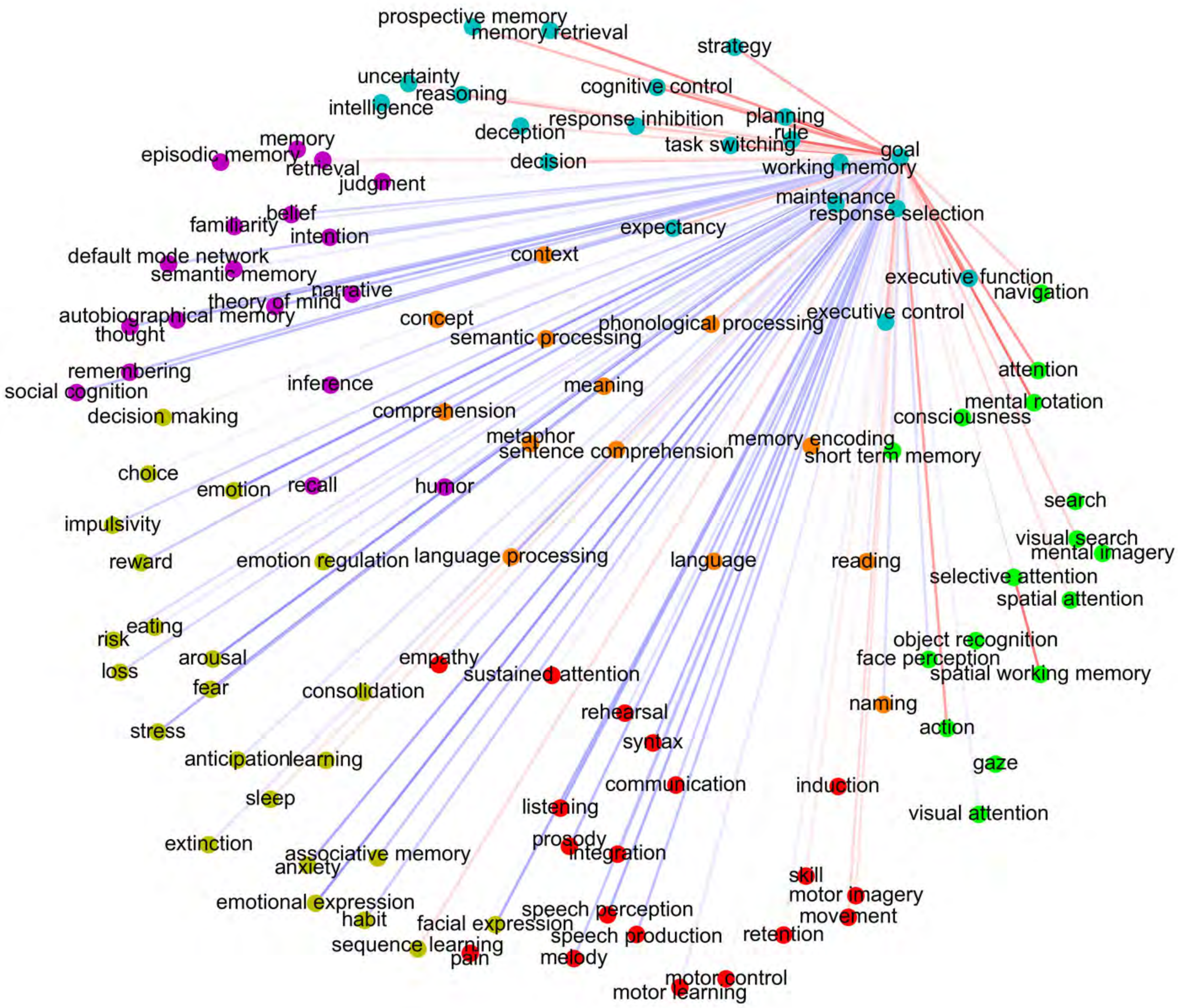
prospective memory
memory retrieval
strategy
uncertainty
reasoning
cognitive control
intelligence
planning
response inhibition
rule
deception
task switching
goal
memory
episodic memory
retrieval
decision
working memory
judgment
maintenance
response selection
belief
familiarity
intention
expectancy
default mode network
context
semantic memory
executive function
navigation
narrative
theory of mind
executive control
autobiographical memory
concept
phonological processing
thought
semantic processing
remembering
attention
social cognition
inference
meaning
mental rotation
decision making
comprehension
consciousness
metaphor
memory encoding
sentence comprehension
short term memory
choice
emotion
recall
humor
search
impulsivity
visual search
mental imagery
reward
emotion regulation
language processing
language
reading
selective attention
spatial attention
eating
risk
object recognition
arousal
empathy
face perception
loss
sustained attention
spatial working memory
fear
consolidation
naming
rehearsal
stress
action
syntax
anticipation
learning
gaze
communication
induction
sleep
listening
visual attention
extinction
prosody
integration
associative memory
anxiety
skill
motor imagery
emotional expression
habit
speech perception
movement
facial expression
speech production
retention
sequence learning
pain
melody
motor control
motor learning

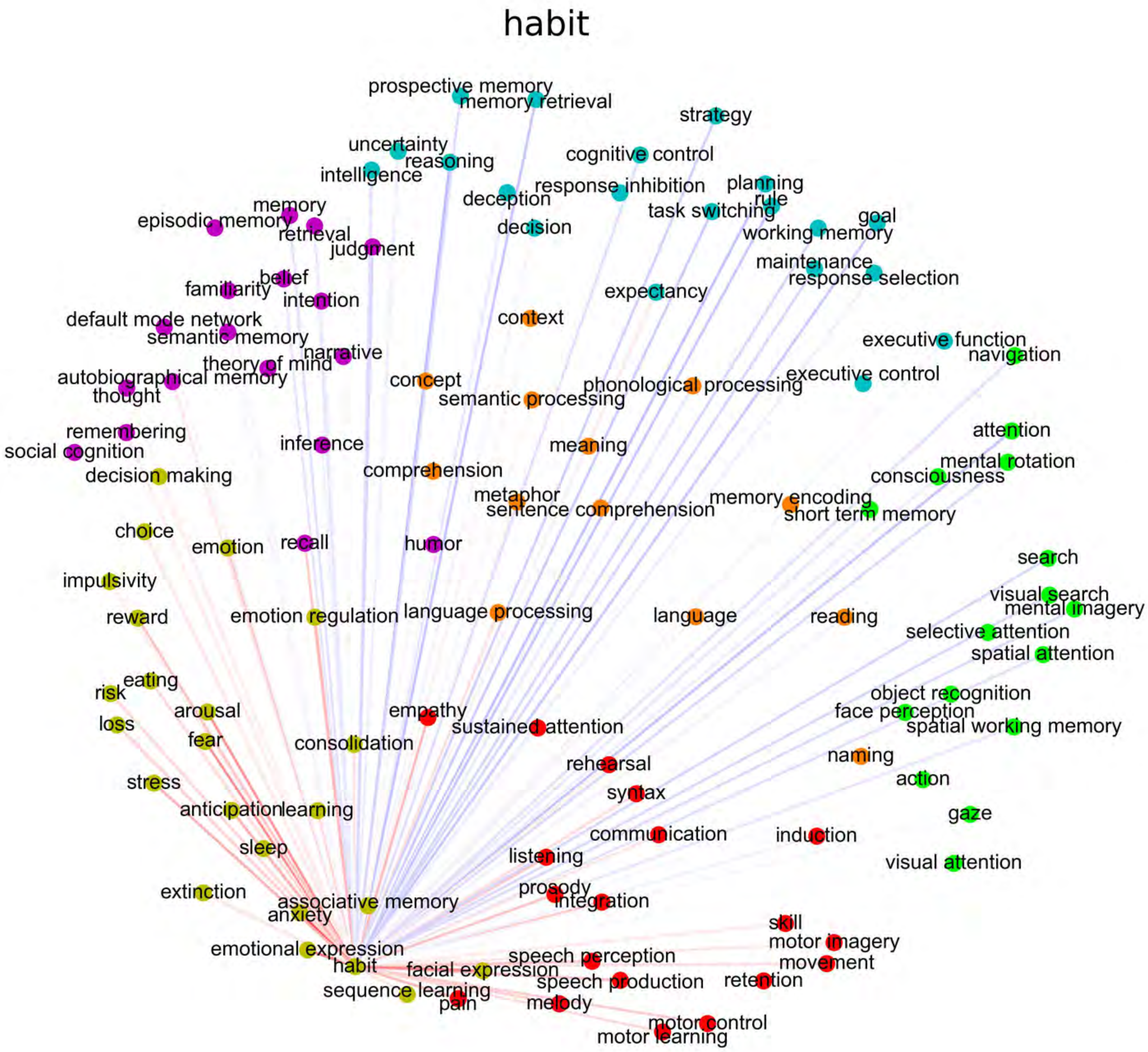
habit
prospective memory
memory retrieval
strategy
uncertainty
reasoning
cognitive control
intelligence
response inhibition
planning
rule
deception
task switching
memory
episodic memory
retrieval
decision
goal
working memory
judgment
maintenance
response selection
belief
familiarity
intention
expectancy
default mode network
context
semantic memory
executive function
navigation
narrative
theory of mind
concept
executive control
autobiographical memory
phonological processing
thought
semantic processing
remembering
attention
social cognition
inference
meaning
mental rotation
decision making
comprehension
consciousness
metaphor
memory encoding
sentence comprehension
short term memory
choice
emotion
recall
humor
search
impulsivity
visual search
mental imagery
reward
emotion regulation
language processing
language
reading
selective attention
spatial attention
risk
eating
object recognition
arousal
empathy
face perception
loss
sustained attention
spatial working memory
fear
consolidation
naming
rehearsal
stress
action
syntax
anticipation
learning
gaze
communication
induction
sleep
listening
visual attention
extinction
prosody
integration
associative memory
anxiety
skill
motor imagery
emotional expression
speech perception
movement
habit
facial expression
speech production
retention
sequence learning
pain
melody
motor control
motor learning

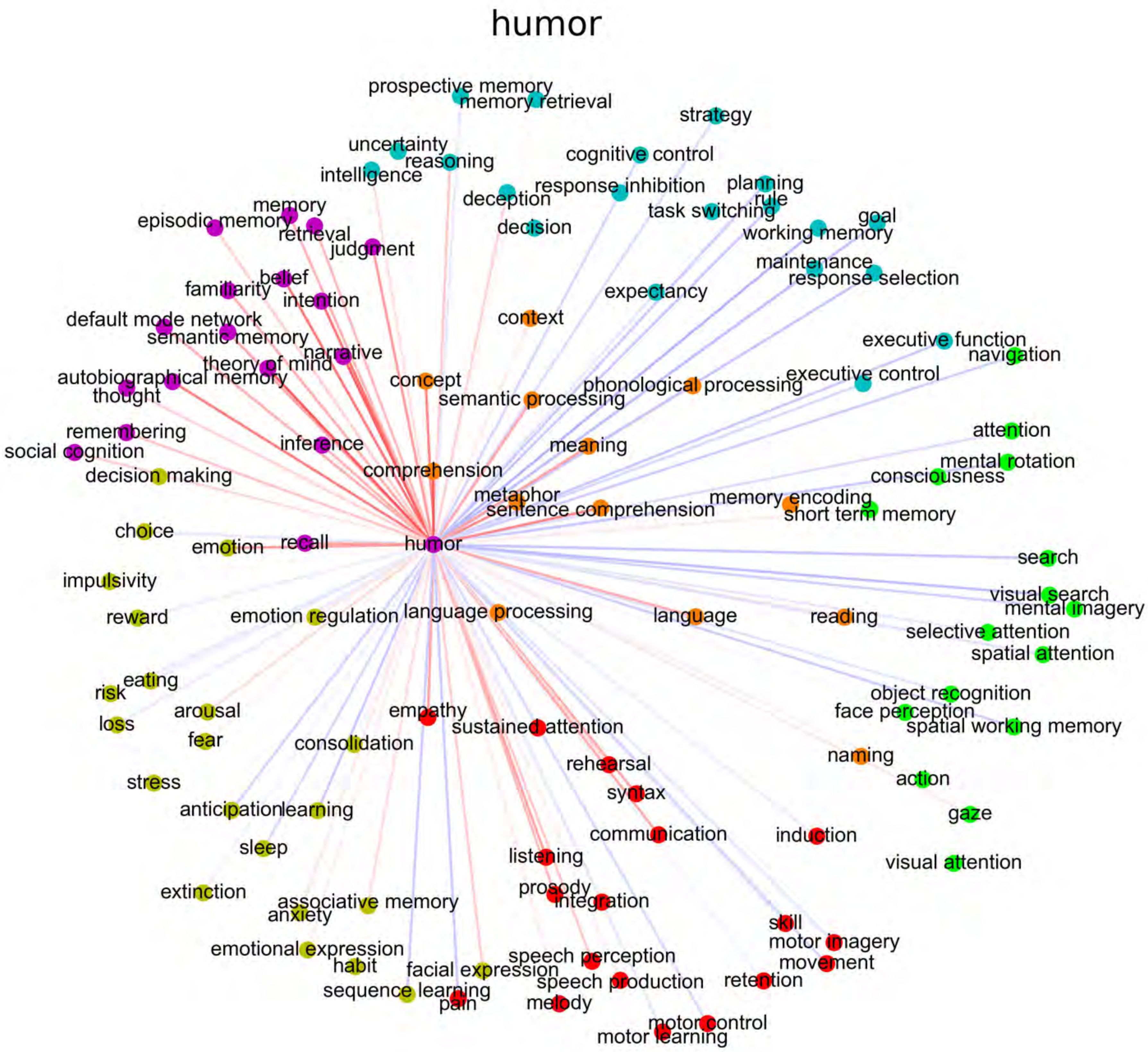
humor
prospective memory
memory retrieval
strategy
uncertainty
reasoning
cognitive control
intelligence
response inhibition
planning
deception
rule
memory
task switching
goal
episodic memory
retrieval
decision
working memory
judgment
maintenance
belief
response selection
familiarity
intention
expectancy
default mode network
context
semantic memory
executive function
narrative
navigation
theory of mind
concept
executive control
autobiographical memory
phonological processing
thought
semantic processing
remembering
attention
inference
meaning
social cognition
mental rotation
comprehension
decision making
consciousness
metaphor
memory encoding
sentence comprehension
short term memory
choice
recall
humor
emotion
search
impulsivity
visual search
mental imagery
reward
emotion regulation
language processing
language
reading
selective attention
spatial attention
eating
object recognition
risk
arousal
empathy
face perception
loss
sustained attention
spatial working memory
fear
consolidation
rehearsal
naming
stress
action
syntax
anticipation
learning
gaze
communication
induction
sleep
listening
visual attention
extinction
prosody
integration
associative memory
anxiety
skill
motor imagery
emotional expression
speech perception
movement
habit
facial expression
speech production
retention
sequence learning
pain
melody
motor control
motor learning

impulsivity

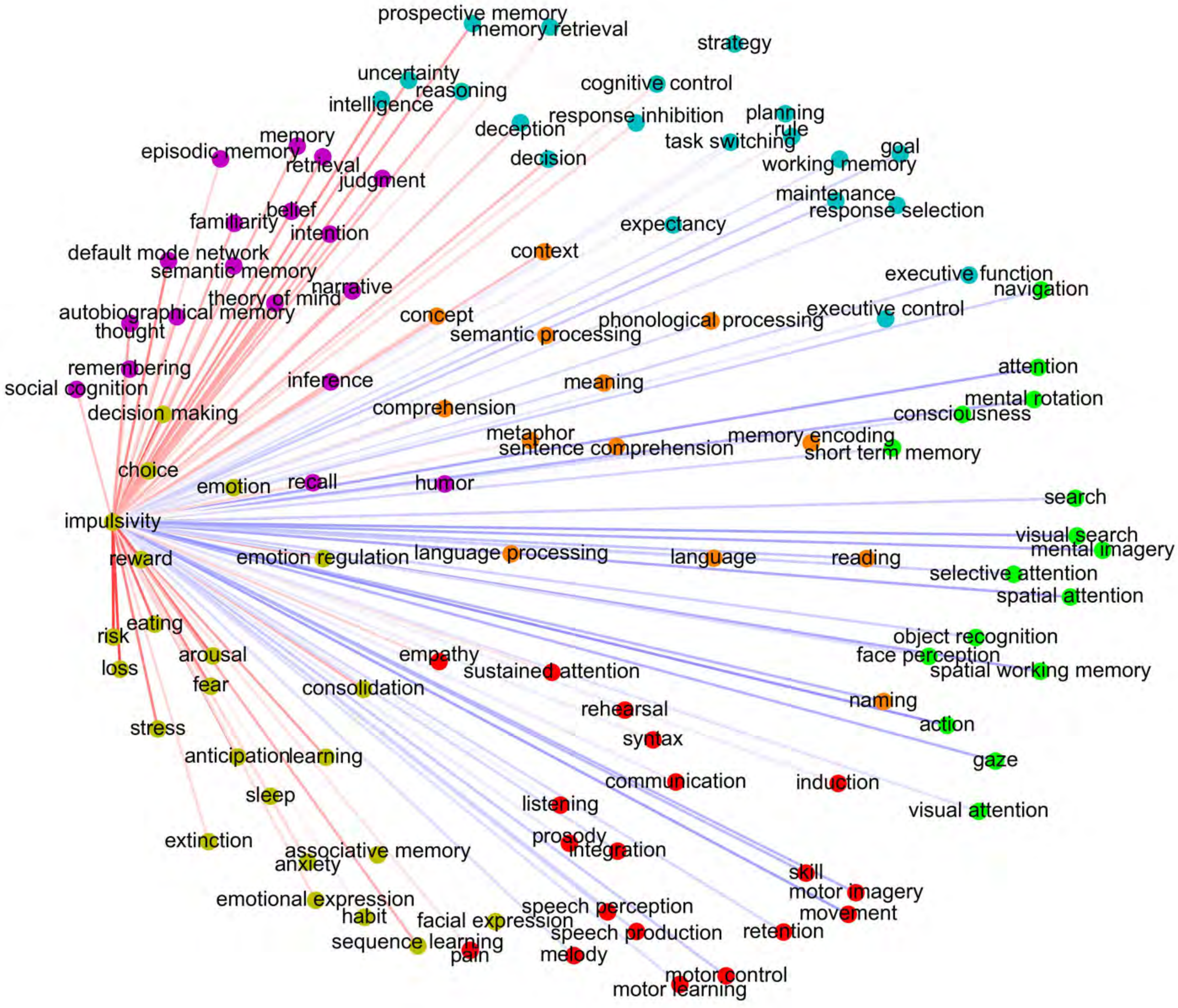

induction

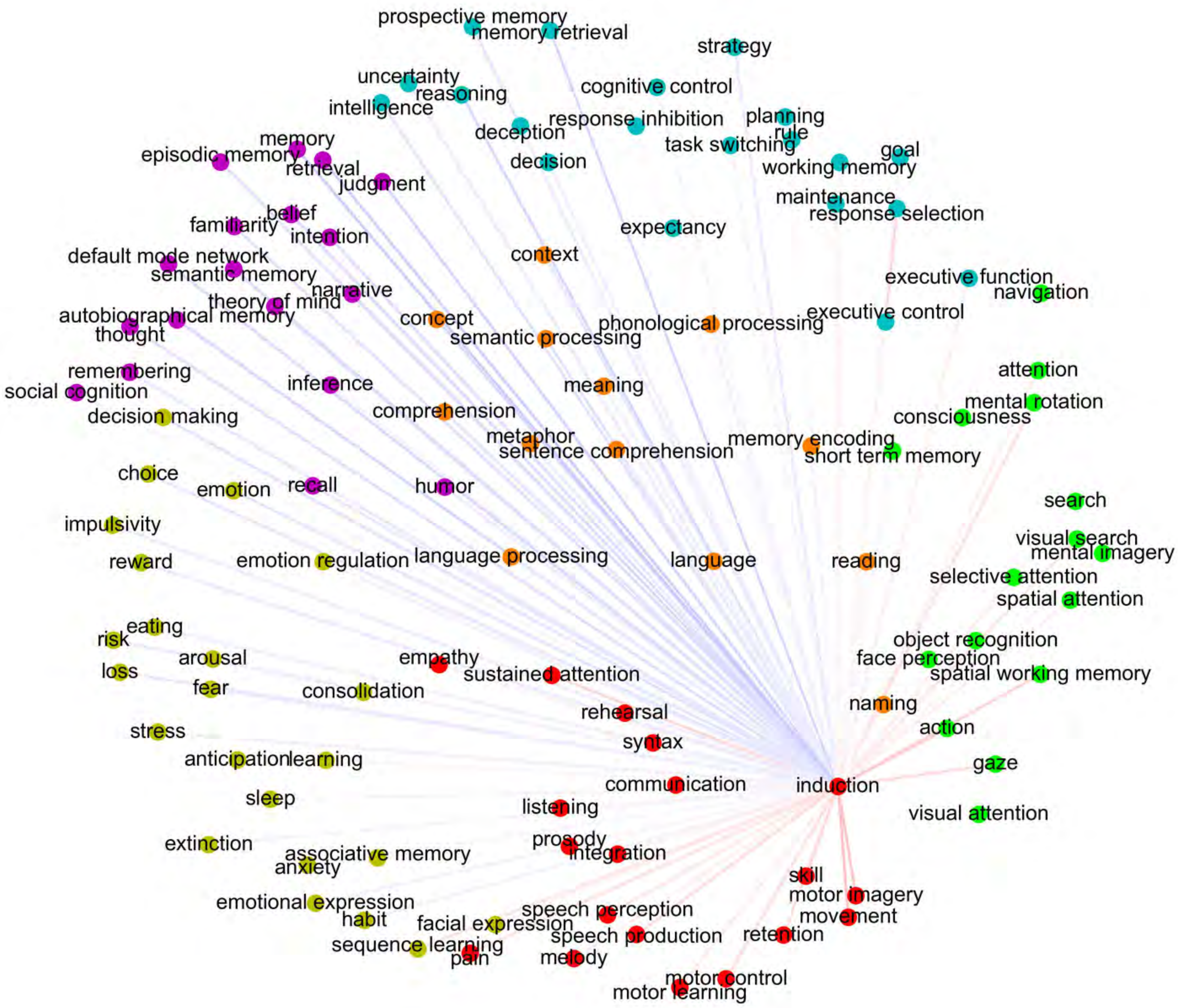
prospective memory
memory retrieval
strategy
uncertainty
reasoning
intelligence
cognitive control
response inhibition
planning
rule
task switching
goal
working memory
deception
decision
memory
episodic memory
retrieval
judgment
maintenance
response selection
belief
familiarity
intention
expectancy
context
default mode network
semantic memory
narrative
theory of mind
executive function
navigation
autobiographical memory
thought
concept
semantic processing
phonological processing
executive control
remembering
social cognition
inference
meaning
attention
mental rotation
consciousness
decision making
comprehension
metaphor
sentence comprehension
memory encoding
short term memory
choice
emotion
recall
humor
search
impulsivity
visual search
mental imagery
reward
emotion regulation
language processing
language
reading
selective attention
spatial attention
risk
eating
arousal
object recognition
face perception
spatial working memory
loss
fear
empathy
sustained attention
consolidation
rehearsal
naming
stress
syntax
action
anticipation
learning
gaze
communication
induction
sleep
listening
visual attention
extinction
prosody
integration
associative memory
anxiety
skill
motor imagery
emotional expression
speech perception
movement
habit
facial expression
speech production
retention
sequence learning
pain
melody
motor control
motor learning

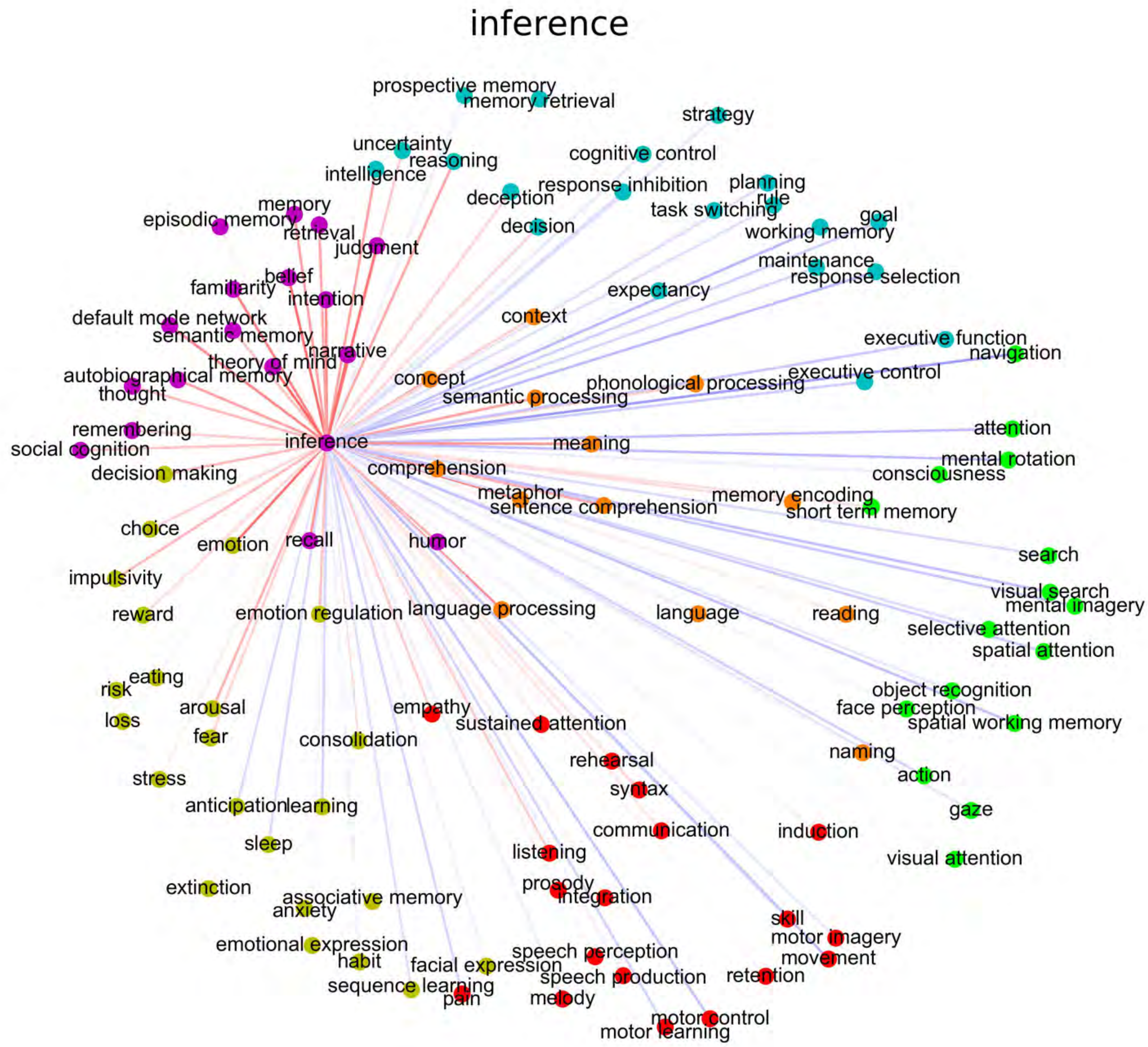
inference
prospective memory
memory retrieval
strategy
uncertainty
reasoning
cognitive control
intelligence
response inhibition
planning
rule
deception
task switching
goal
memory
episodic memory
retrieval
decision
working memory
judgment
maintenance
response selection
belief
familiarity
intention
expectancy
default mode network
context
semantic memory
executive function
navigation
narrative
theory of mind
executive control
concept
phonological processing
autobiographical memory
thought
semantic processing
remembering
attention
inference
meaning
social cognition
mental rotation
decision making
comprehension
consciousness
metaphor
memory encoding
sentence comprehension
short term memory
choice
emotion
recall
humor
search
impulsivity
visual search
mental imagery
reward
emotion regulation
language processing
language
reading
selective attention
spatial attention
object recognition
eating
risk
face perception
arousal
empathy
spatial working memory
loss
sustained attention
fear
consolidation
rehearsal
naming
action
stress
syntax
anticipation
learning
gaze
communication
induction
sleep
listening
visual attention
extinction
prosody
integration
associative memory
anxiety
skill
motor imagery
emotional expression
speech perception
movement
habit
facial expression
speech production
retention
sequence learning
pain
melody
motor control
motor learning

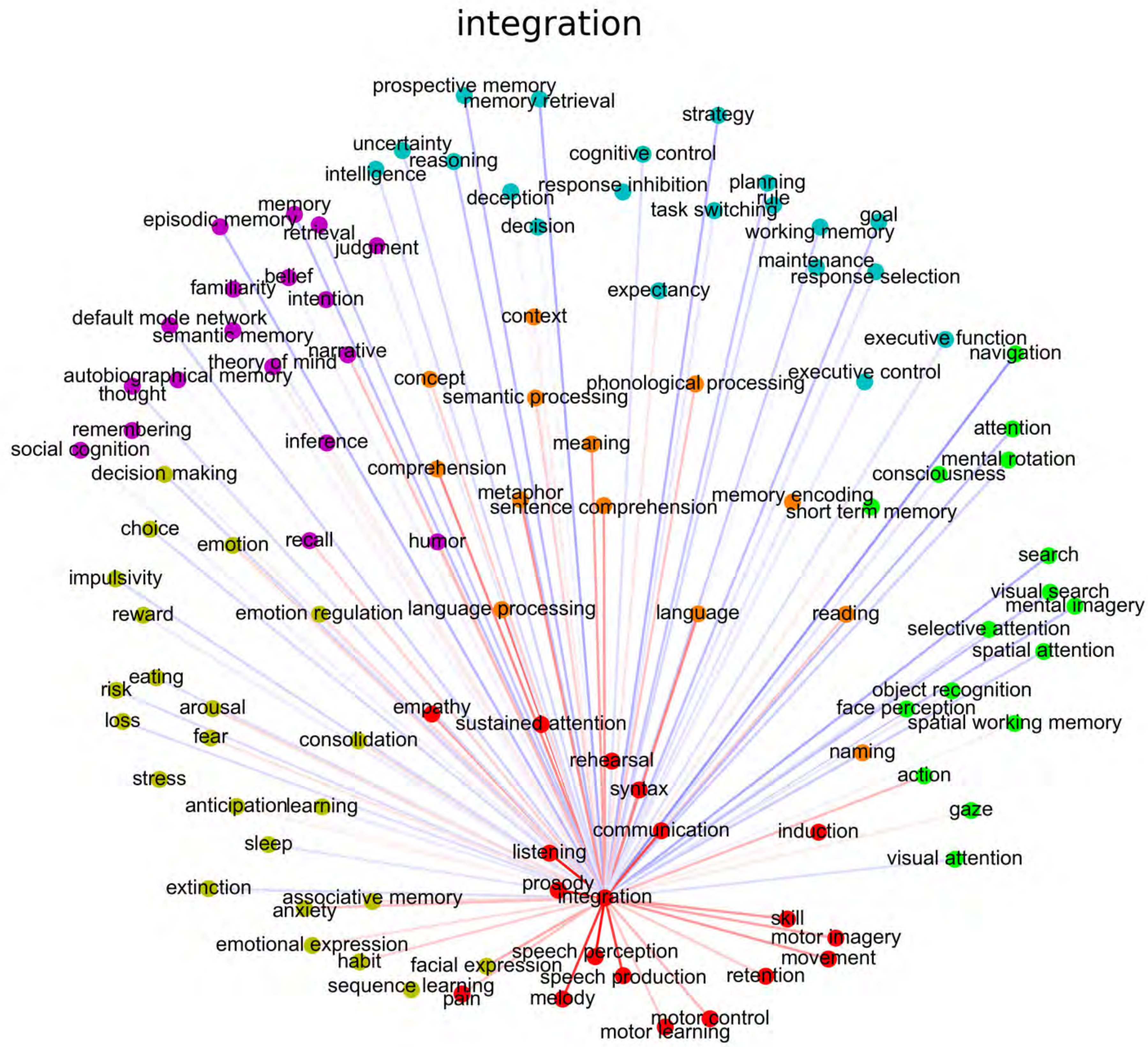
integration
prospective memory
memory retrieval
strategy
uncertainty
reasoning
cognitive control
intelligence
response inhibition
planning
rule
deception
task switching
goal
memory
episodic memory
retrieval
decision
working memory
judgment
maintenance
response selection
belief
familiarity
intention
expectancy
context
default mode network
semantic memory
executive function
navigation
narrative
theory of mind
concept
phonological processing
executive control
autobiographical memory
thought
semantic processing
remembering
social cognition
inference
meaning
attention
mental rotation
decision making
comprehension
consciousness
metaphor
sentence comprehension
memory encoding
short term memory
choice
emotion
recall
humor
search
impulsivity
visual search
mental imagery
reward
emotion regulation
language processing
language
reading
selective attention
spatial attention
eating
risk
arousal
object recognition
empathy
face perception
loss
sustained attention
spatial working memory
fear
consolidation
rehearsal
naming
stress
action
syntax
anticipation
learning
gaze
communication
induction
sleep
listening
visual attention
extinction
prosody
integration
associative memory
anxiety
skill
motor imagery
emotional expression
habit
speech perception
movement
facial expression
speech production
retention
sequence learning
pain
melody
motor control
motor learning

intelligence

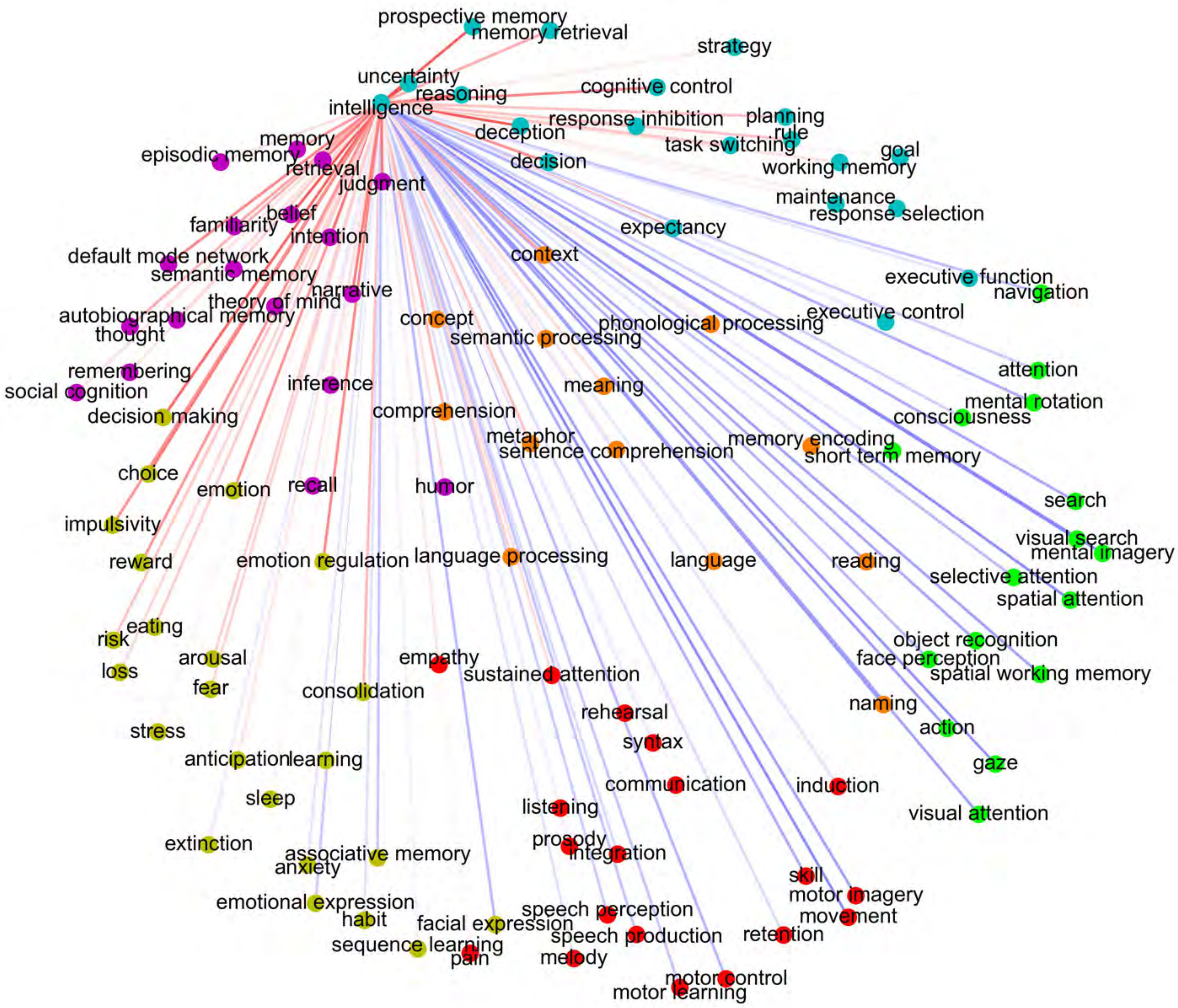

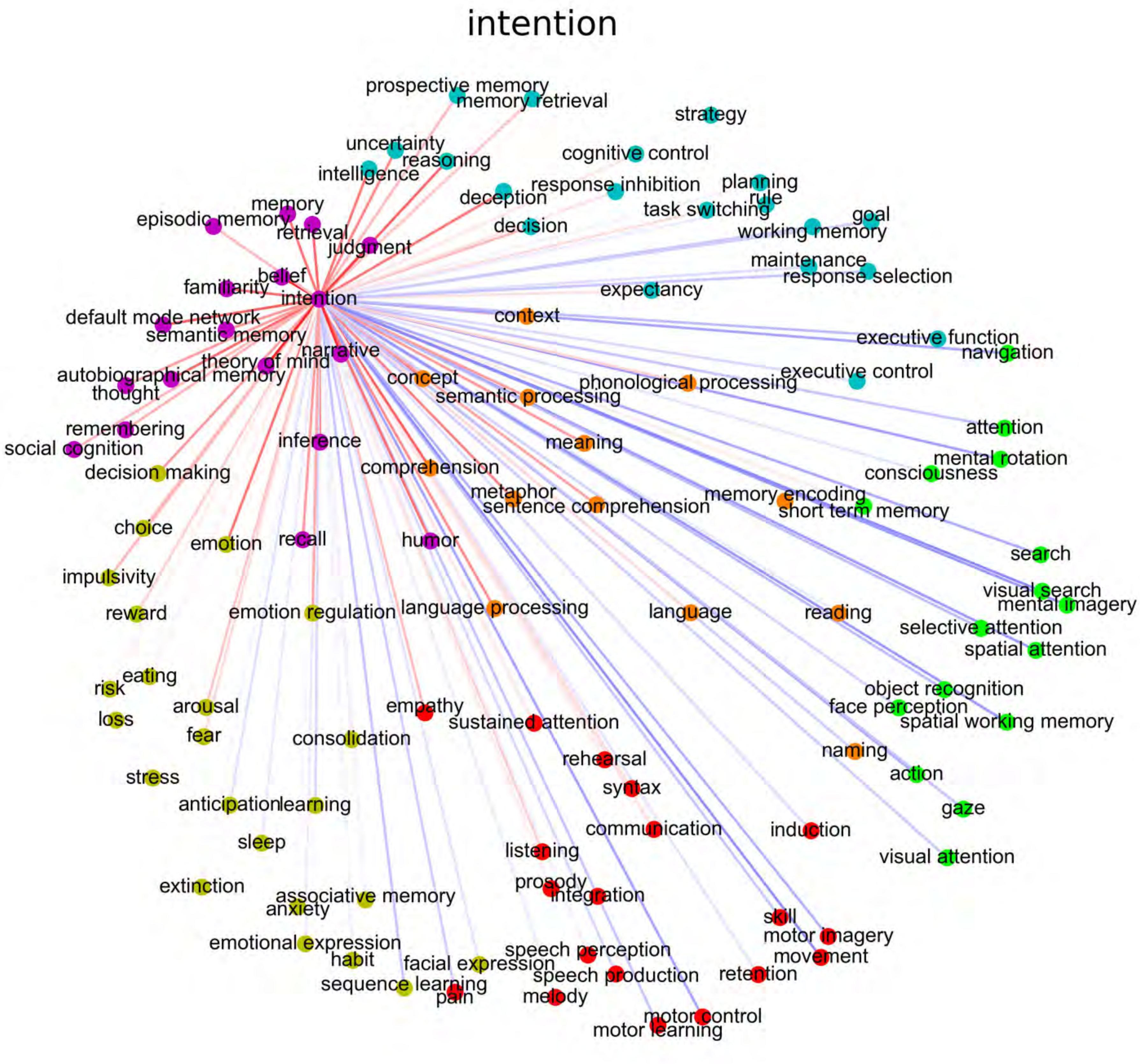
intention
prospective memory
memory retrieval
strategy
uncertainty
reasoning
cognitive control
intelligence
response inhibition
planning
rule
deception
task switching
memory
episodic memory
retrieval
decision
goal
working memory
judgment
maintenance
belief
response selection
familiarity
intention
expectancy
default mode network
context
semantic memory
executive function
navigation
narrative
theory of mind
autobiographical memory
thought
concept
executive control
phonological processing
semantic processing
remembering
attention
social cognition
inference
meaning
decision making
mental rotation
comprehension
consciousness
metaphor
memory encoding
sentence comprehension
short term memory
choice
emotion
recall
humor
search
impulsivity
visual search
mental imagery
reward
emotion regulation
language processing
language
reading
selective attention
spatial attention
object recognition
eating
risk
arousal
empathy
face perception
loss
sustained attention
spatial working memory
fear
consolidation
rehearsal
naming
action
stress
syntax
anticipation
learning
gaze
communication
induction
sleep
listening
visual attention
extinction
prosody
integration
associative memory
anxiety
skill
motor imagery
emotional expression
speech perception
movement
habit
facial expression
speech production
retention
sequence learning
pain
melody
motor control
motor learning

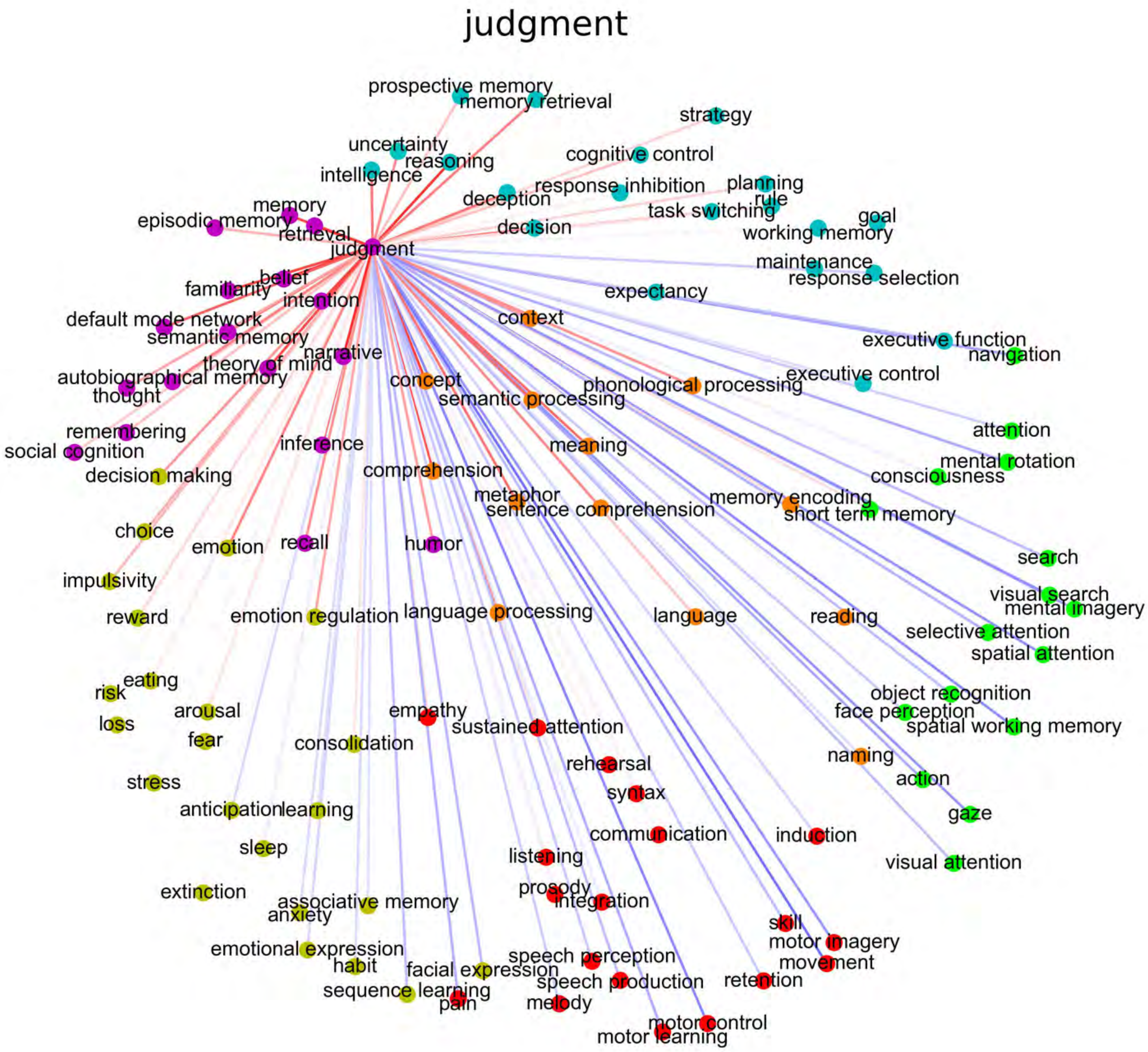
judgment
prospective memory
memory retrieval
strategy
uncertainty
reasoning
cognitive control
intelligence
response inhibition
planning
rule
deception
task switching
memory
goal
episodic memory
retrieval
decision
working memory
judgment
maintenance
response selection
belief
familiarity
intention
expectancy
default mode network
context
semantic memory
executive function
navigation
narrative
theory of mind
executive control
autobiographical memory
concept
phonological processing
thought
semantic processing
remembering
attention
social cognition
inference
meaning
decision making
comprehension
mental rotation
consciousness
metaphor
memory encoding
sentence comprehension
short term memory
choice
emotion
recall
humor
search
impulsivity
visual search
mental imagery
reward
emotion regulation
language processing
language
reading
selective attention
spatial attention
eating
risk
object recognition
arousal
empathy
face perception
loss
sustained attention
spatial working memory
fear
consolidation
naming
rehearsal
stress
action
syntax
anticipation
learning
gaze
communication
induction
sleep
listening
visual attention
extinction
prosody
integration
associative memory
anxiety
skill
motor imagery
emotional expression
movement
speech perception
habit
facial expression
speech production
retention
sequence learning
pain
melody
motor control
motor learning

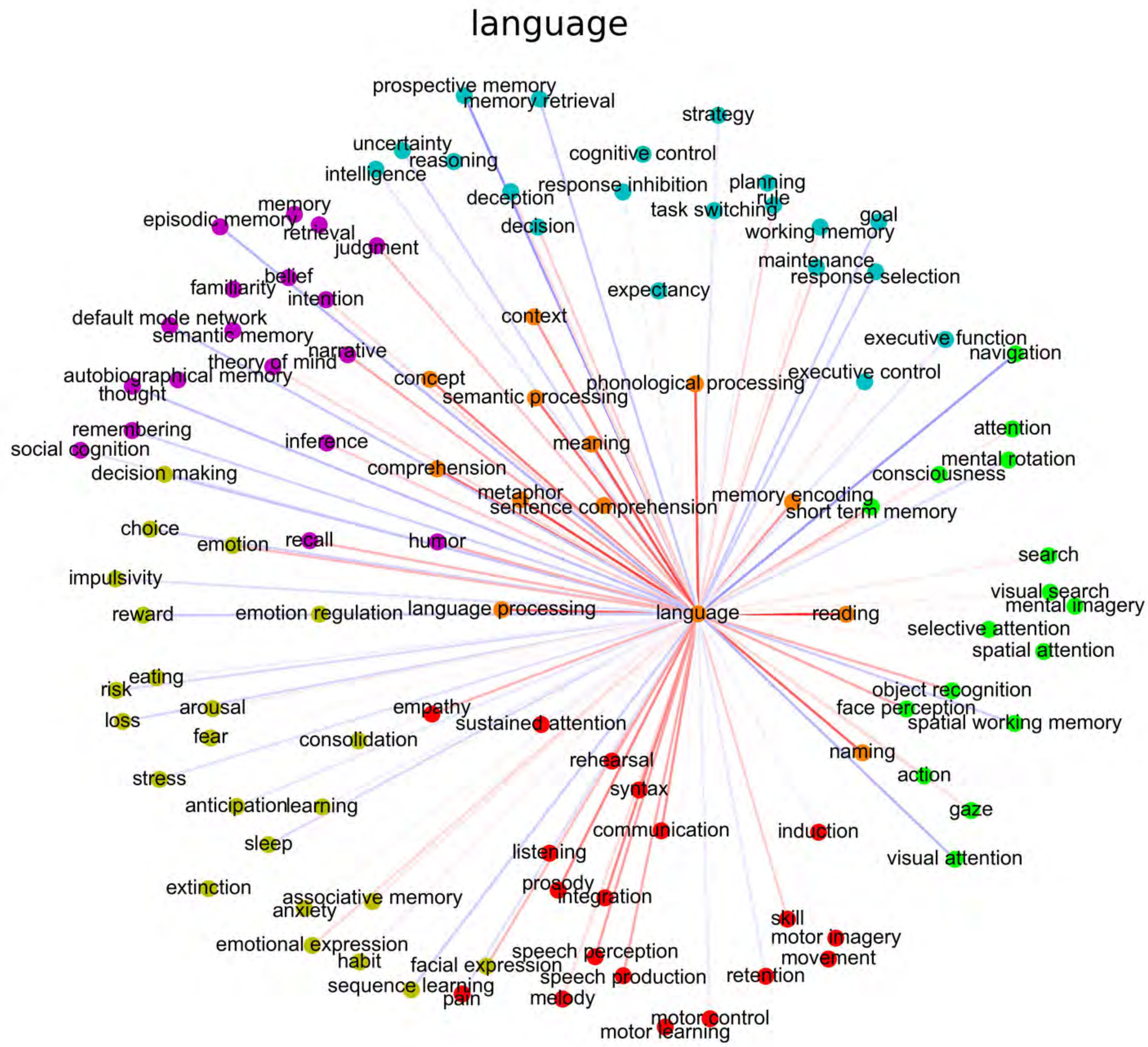
language
prospective memory
memory retrieval
strategy
uncertainty
reasoning
cognitive control
intelligence
response inhibition
planning
rule
deception
task switching
goal
memory
episodic memory
retrieval
decision
working memory
judgment
maintenance
response selection
belief
familiarity
intention
expectancy
context
default mode network
semantic memory
executive function
navigation
narrative
theory of mind
executive control
autobiographical memory
concept
phonological processing
thought
semantic processing
remembering
attention
meaning
social cognition
inference
mental rotation
decision making
comprehension
consciousness
metaphor
memory encoding
sentence comprehension
short term memory
choice
emotion
recall
humor
search
impulsivity
visual search
mental imagery
reward
emotion regulation
language processing
language
reading
selective attention
spatial attention
eating
risk
object recognition
arousal
empathy
face perception
loss
sustained attention
spatial working memory
fear
consolidation
rehearsal
naming
stress
action
syntax
anticipation
learning
gaze
communication
induction
sleep
listening
visual attention
extinction
prosody
integration
associative memory
anxiety
skill
motor imagery
emotional expression
movement
speech perception
habit
facial expression
speech production
retention
sequence learning
pain
melody
motor control
motor learning

language processing

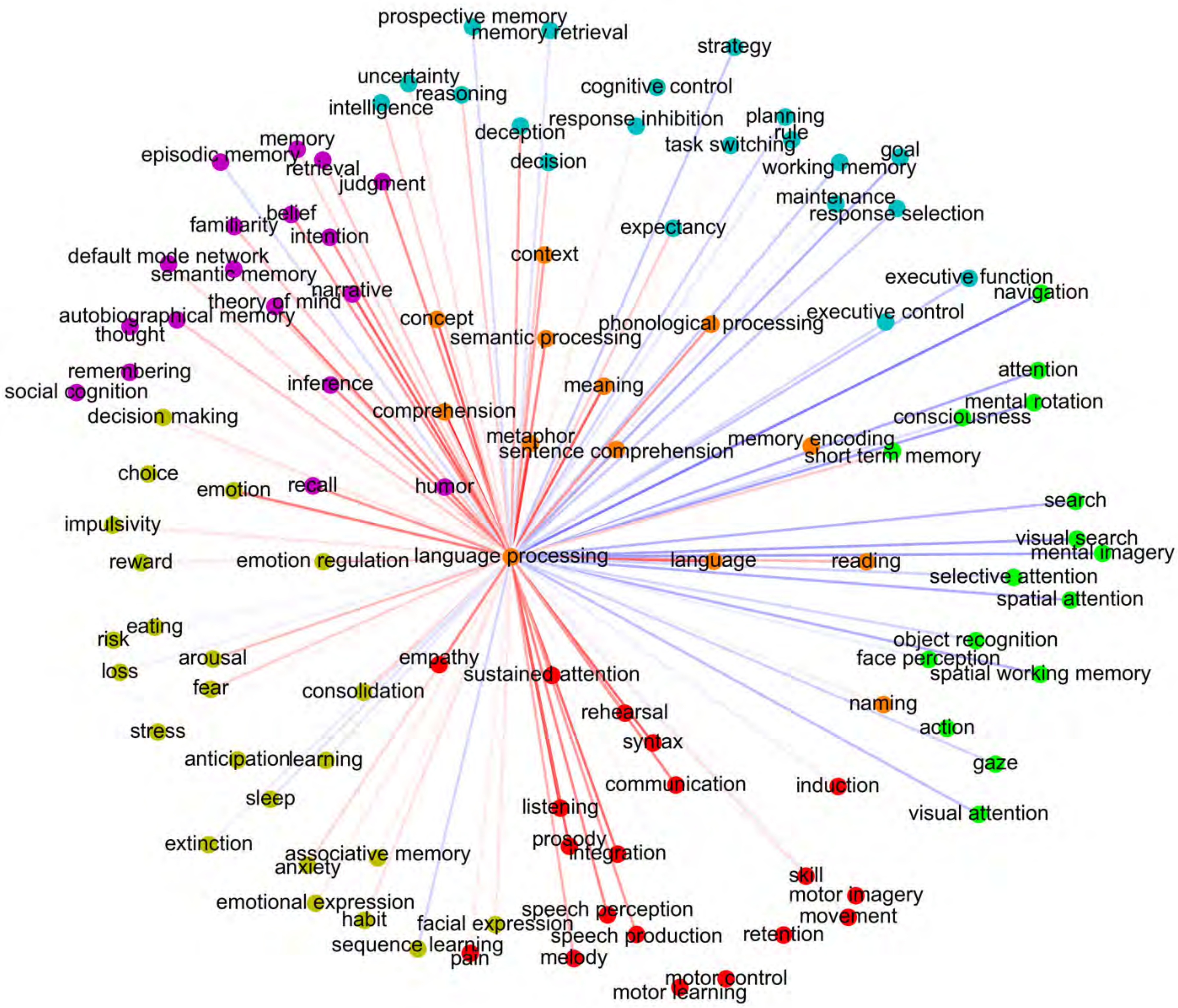

learning

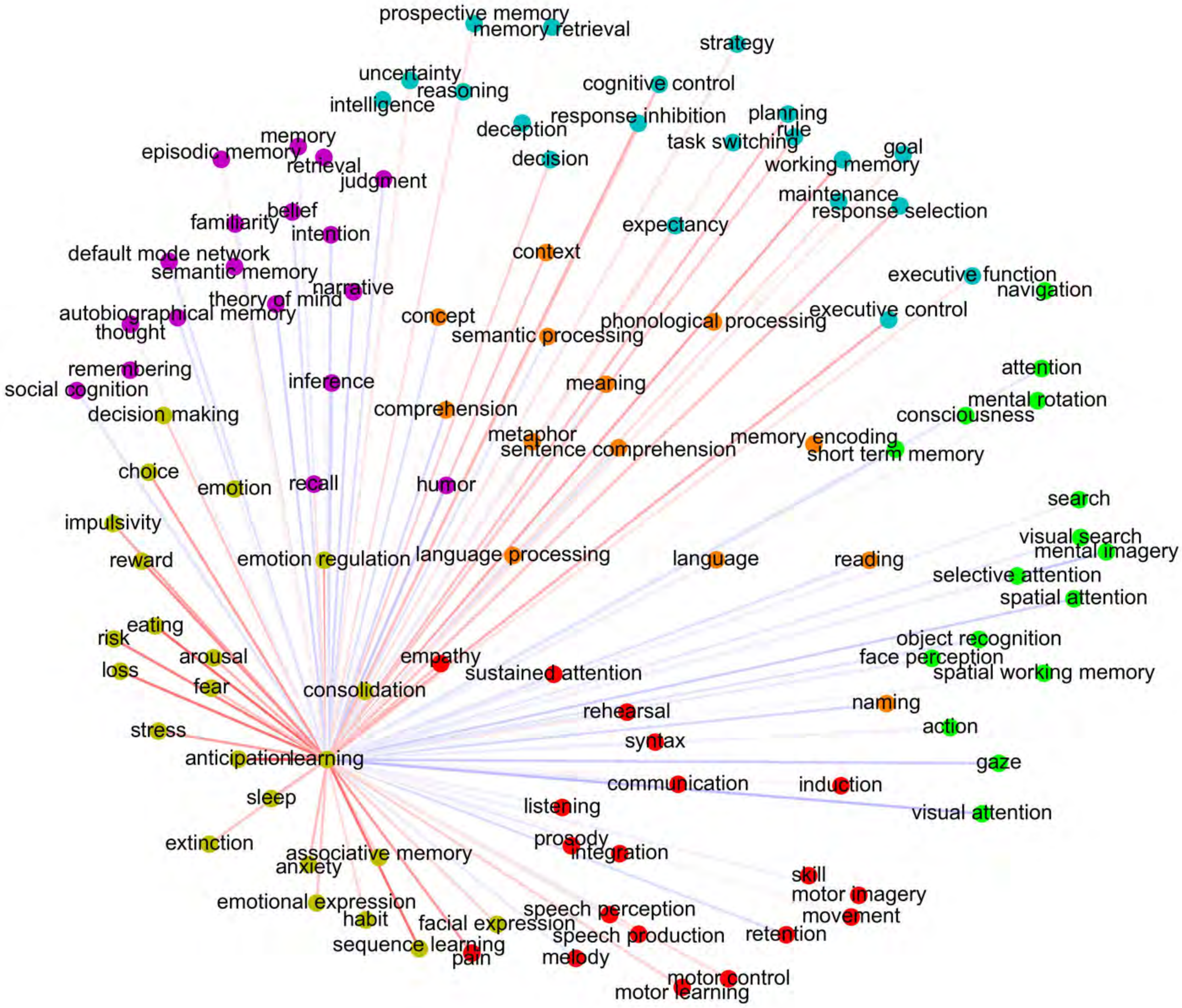
prospective memory
memory retrieval
strategy
uncertainty
reasoning
cognitive control
intelligence
deception
response inhibition
planning
rule
task switching
goal
working memory
decision
memory
episodic memory
retrieval
judgment
maintenance
response selection
belief
familiarity
intention
expectancy
context
default mode network
semantic memory
executive function
navigation
narrative
theory of mind
concept
phonological processing
executive control
autobiographical memory
thought
semantic processing
remembering
social cognition
inference
meaning
attention
comprehension
mental rotation
decision making
consciousness
metaphor
sentence comprehension
memory encoding
short term memory
choice
emotion
recall
humor
search
impulsivity
visual search
mental imagery
reward
emotion regulation
language processing
language
reading
selective attention
spatial attention
eating
risk
arousal
empathy
object recognition
face perception
loss
sustained attention
spatial working memory
fear
consolidation
rehearsal
naming
stress
action
syntax
anticipation
learning
gaze
communication
induction
sleep
listening
visual attention
extinction
prosody
integration
associative memory
anxiety
skill
motor imagery
emotional expression
speech perception
movement
habit
facial expression
speech production
retention
sequence learning
pain
melody
motor control
motor learning

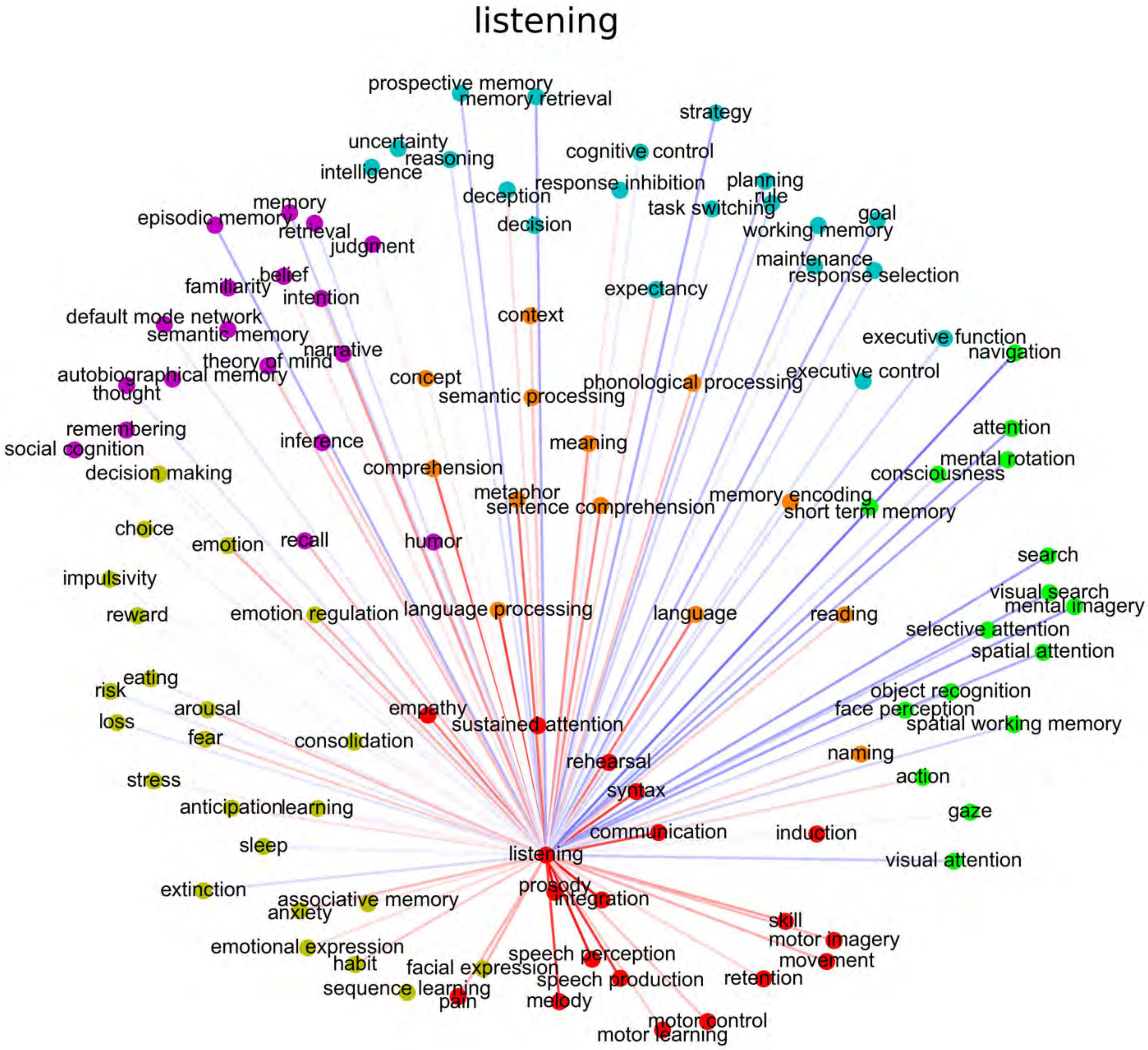
listening
prospective memory
memory retrieval
strategy
uncertainty
reasoning
intelligence
cognitive control
response inhibition
planning
rule
deception
task switching
goal
memory
episodic memory
retrieval
decision
working memory
judgment
maintenance
response selection
belief
familiarity
intention
expectancy
default mode network
semantic memory
context
executive function
navigation
narrative
theory of mind
concept
executive control
autobiographical memory
thought
phonological processing
semantic processing
remembering
social cognition
inference
meaning
attention
mental rotation
consciousness
decision making
comprehension
metaphor
sentence comprehension
memory encoding
short term memory
choice
emotion
recall
humor
search
impulsivity
visual search
mental imagery
reward
emotion regulation
language processing
language
reading
selective attention
spatial attention
eating
risk
arousal
empathy
object recognition
face perception
loss
fear
sustained attention
consolidation
spatial working memory
rehearsal
naming
stress
syntax
action
anticipation
learning
gaze
communication
induction
sleep
listening
visual attention
extinction
prosody
integration
associative memory
anxiety
skill
motor imagery
emotional expression
speech perception
movement
habit
facial expression
speech production
retention
sequence learning
pain
melody
motor control
motor learning

loss

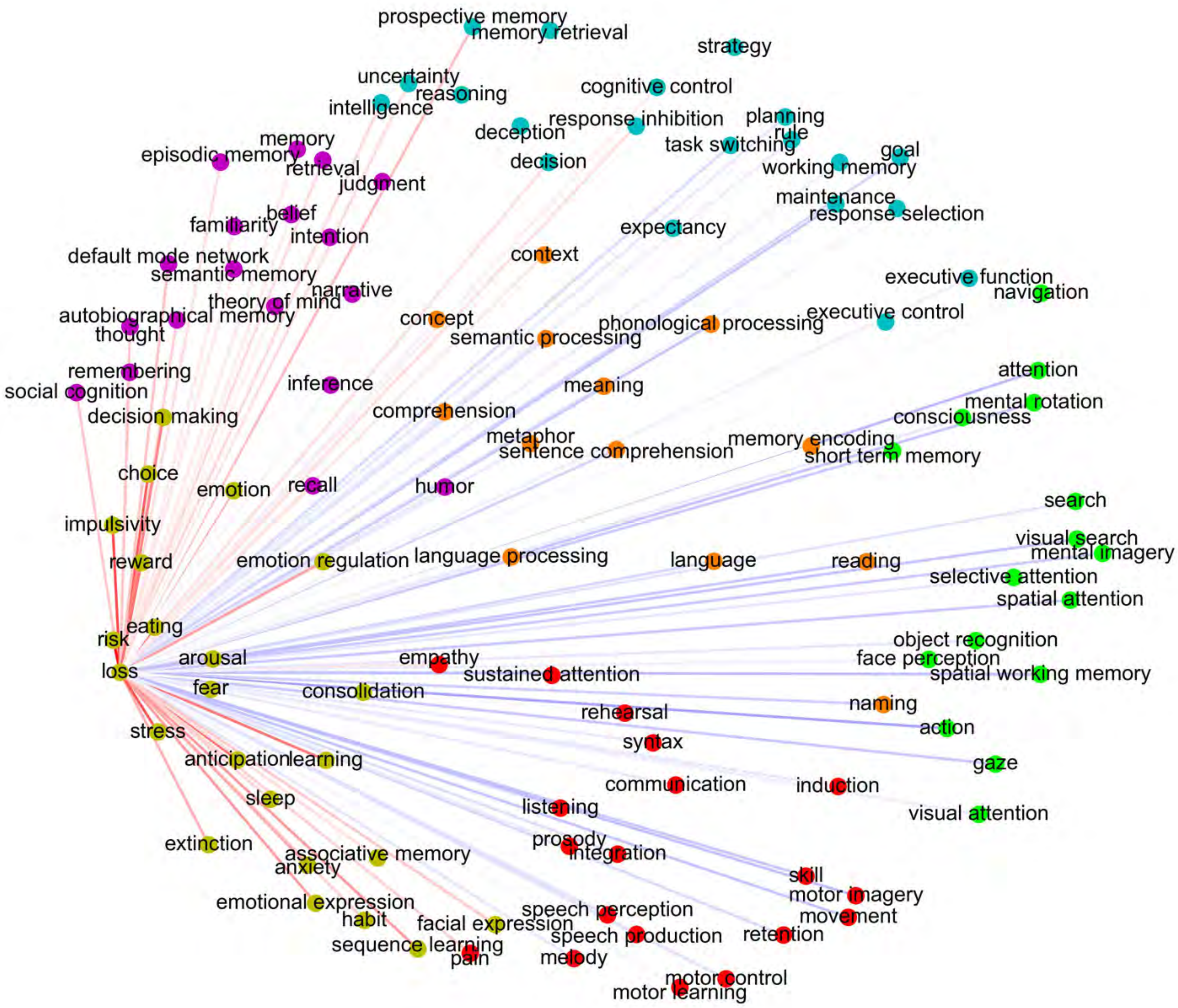

prospective memory
memory retrieval
strategy
uncertainty
reasoning
cognitive control
intelligence
response inhibition
planning
deception
rule
task switching
memory
episodic memory
retrieval
goal
decision
working memory
judgment
belief
maintenance
familiarity
intention
response selection
expectancy
default mode network
context
semantic memory
executive function
narrative
navigation
theory of mind
concept
executive control
autobiographical memory
phonological processing
thought
semantic processing
remembering
attention
social cognition
inference
meaning
decision making
comprehension
mental rotation
consciousness
metaphor
memory encoding
sentence comprehension
short term memory
choice
emotion
recall
humor
search
impulsivity
visual search
reward
emotion regulation
language processing
language
reading
mental imagery
selective attention
spatial attention
eating
risk
object recognition
arousal
empathy
face perception
loss
sustained attention
spatial working memory
fear
consolidation
naming
rehearsal
action
stress
syntax
anticipation
learning
gaze
communication
induction
sleep
listening
visual attention
extinction
prosody
integration
associative memory
anxiety
skill
motor imagery
emotional expression
speech perception
habit
movement
facial expression
speech production
retention
sequence learning
pain
melody
motor control
motor learning

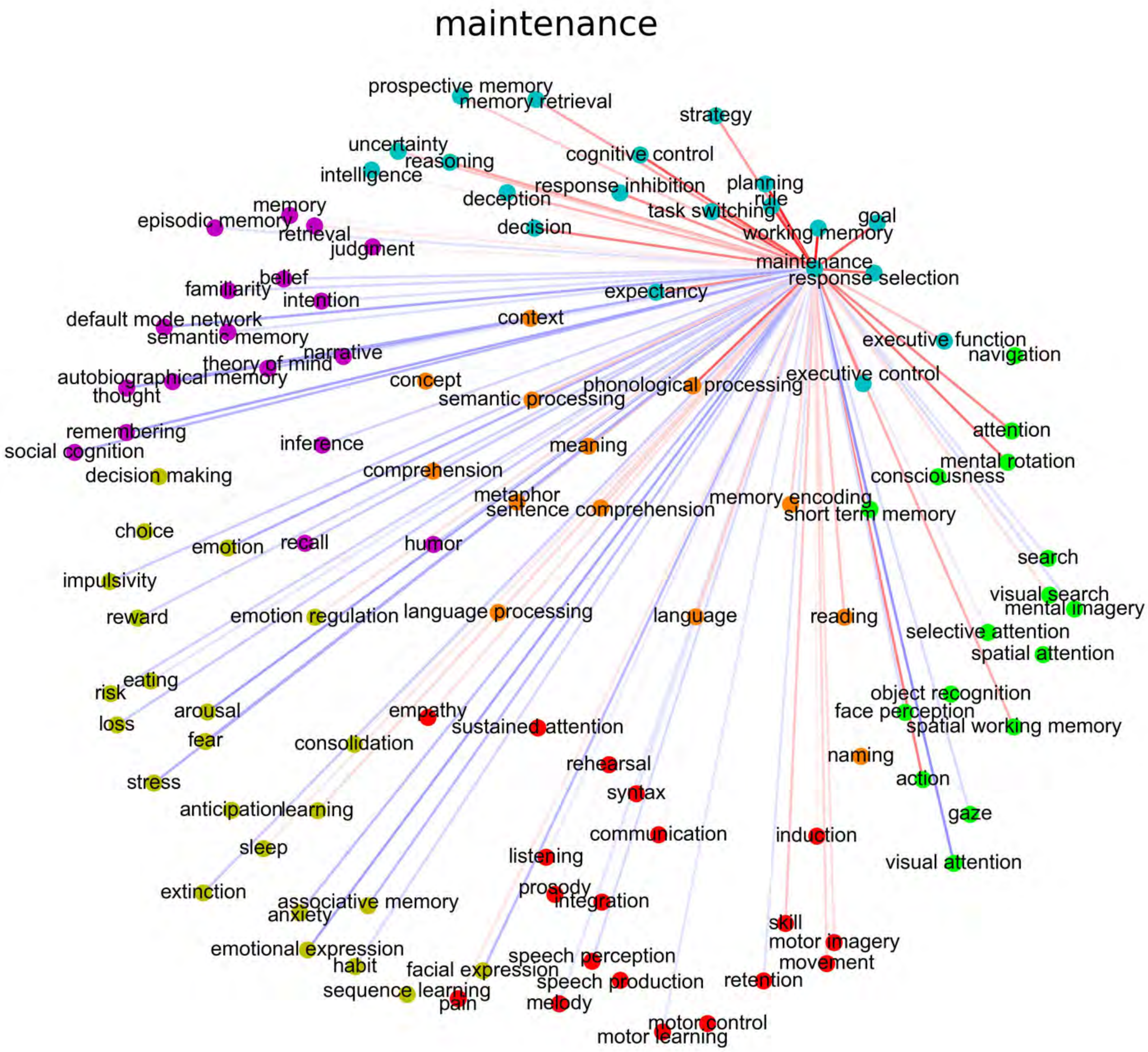
maintenance
prospective memory
memory retrieval
strategy
uncertainty
reasoning
cognitive control
intelligence
response inhibition
planning
rule
deception
task switching
goal
memory
episodic memory
retrieval
decision
working memory
judgment
maintenance
response selection
belief
familiarity
intention
expectancy
default mode network
context
semantic memory
executive function
navigation
narrative
theory of mind
autobiographical memory
executive control
concept
phonological processing
thought
semantic processing
remembering
attention
social cognition
inference
meaning
mental rotation
decision making
comprehension
consciousness
metaphor
memory encoding
sentence comprehension
short term memory
choice
emotion
recall
humor
search
impulsivity
visual search
mental imagery
reward
emotion regulation
language processing
language
reading
selective attention
spatial attention
risk
eating
object recognition
arousal
empathy
face perception
loss
sustained attention
spatial working memory
fear
consolidation
rehearsal
naming
stress
syntax
action
anticipation
learning
gaze
communication
induction
sleep
listening
visual attention
extinction
prosody
integration
associative memory
anxiety
skill
motor imagery
emotional expression
movement
habit
speech perception
facial expression
speech production
retention
sequence learning
pain
melody
motor control
motor learning

meaning

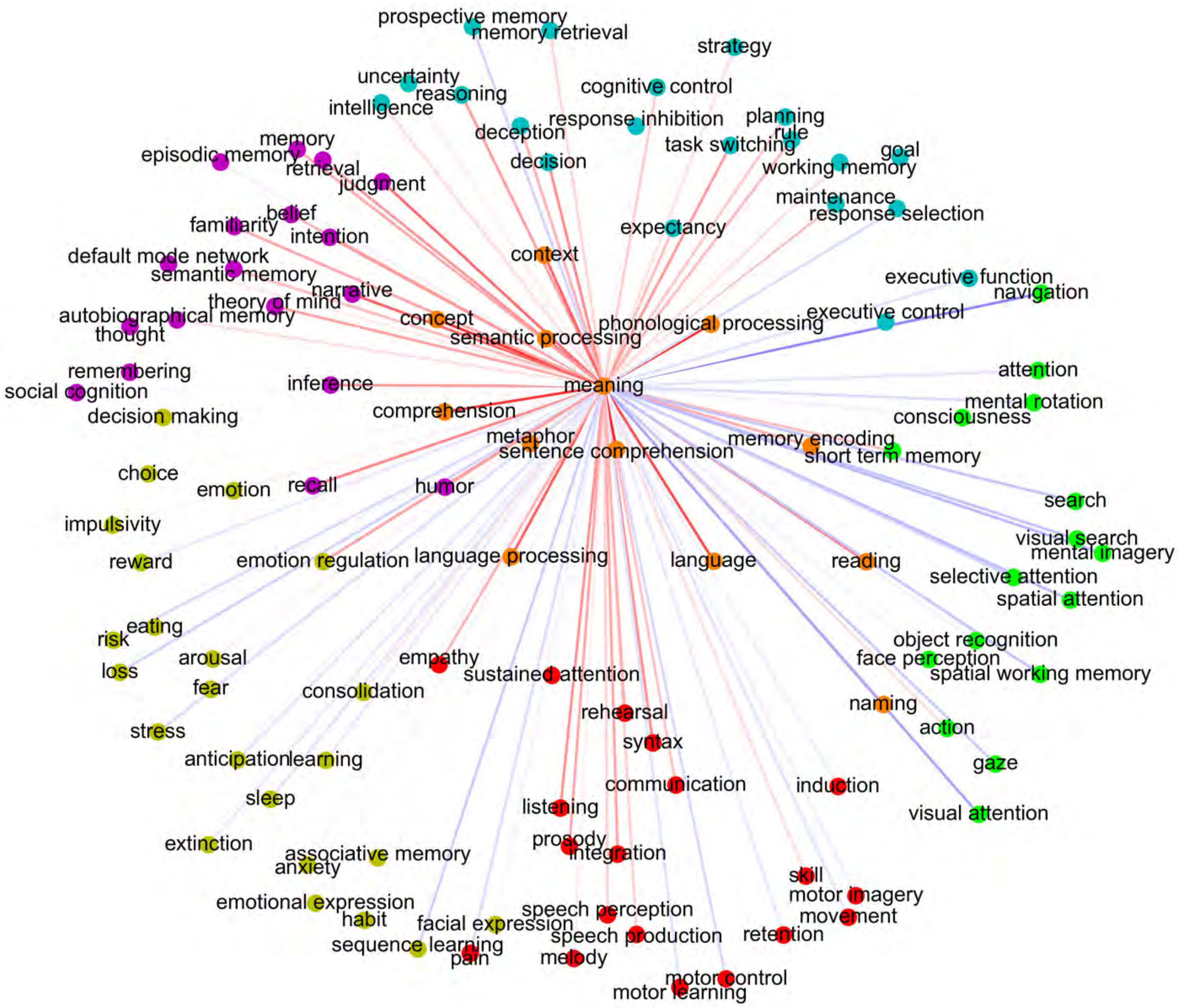

prospective memory
memory retrieval
strategy
uncertainty
reasoning
cognitive control
intelligence
response inhibition
planning
deception
rule
task switching
goal
memory
episodic memory
retrieval
decision
working memory
judgment
maintenance
belief
response selection
familiarity
intention
expectancy
default mode network
context
semantic memory
executive function
narrative
navigation
theory of mind
executive control
autobiographical memory
concept
phonological processing
thought
semantic processing
remembering
attention
inference
meaning
social cognition
mental rotation
decision making
comprehension
consciousness
metaphor
memory encoding
sentence comprehension
short term memory
choice
emotion
recall
humor
search
impulsivity
visual search
mental imagery
reward
emotion regulation
language processing
language
reading
selective attention
spatial attention
eating
risk
object recognition
arousal
empathy
face perception
loss
sustained attention
spatial working memory
fear
consolidation
rehearsal
naming
stress
action
syntax
anticipation
learning
gaze
communication
induction
sleep
listening
visual attention
extinction
prosody
integration
associative memory
anxiety
skill
motor imagery
emotional expression
speech perception
movement
habit
facial expression
speech production
retention
sequence learning
pain
melody
motor control
motor learning

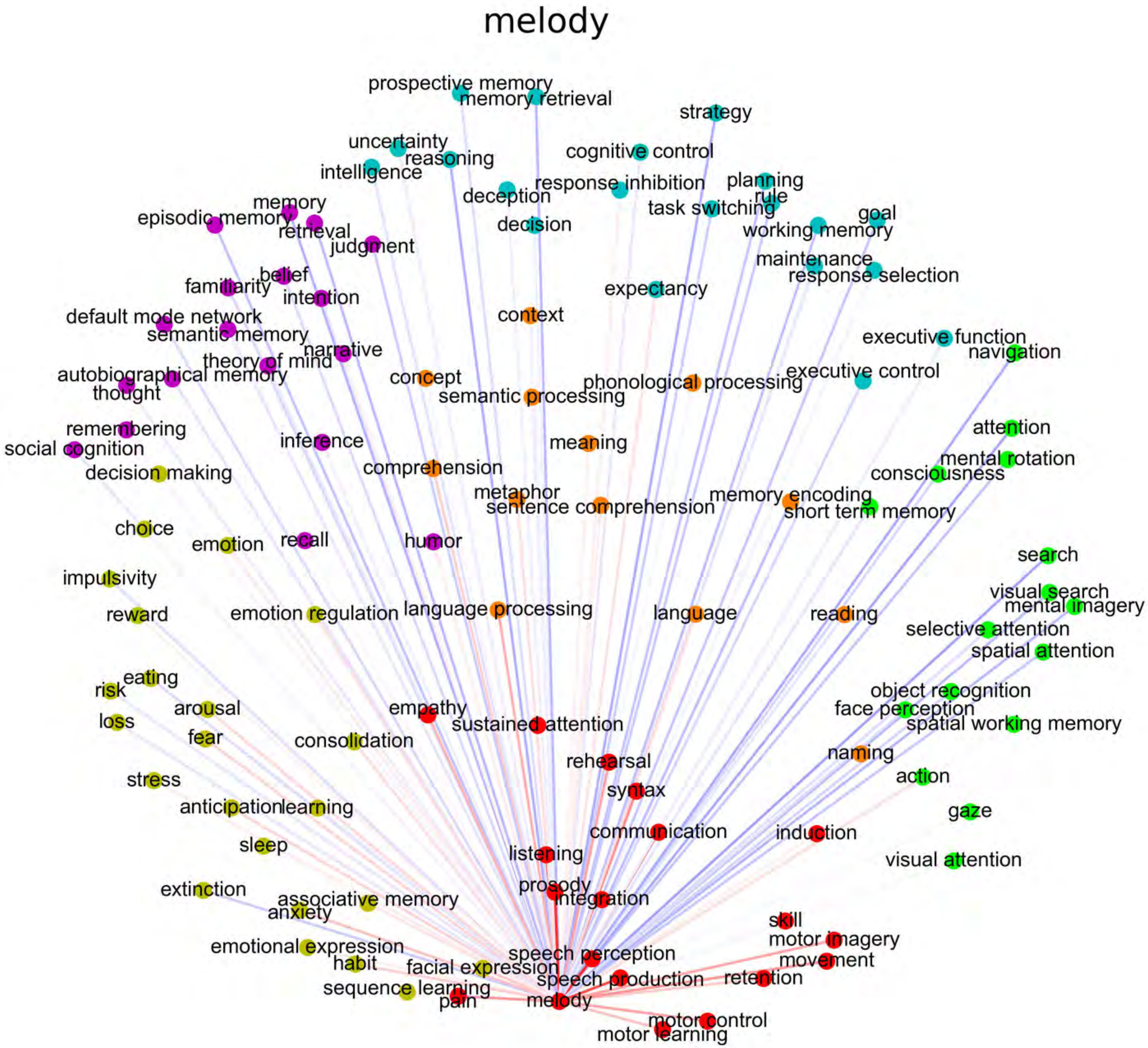
melody
prospective memory
memory retrieval
strategy
uncertainty
reasoning
cognitive control
intelligence
response inhibition
planning
deception
rule
memory
task switching
goal
episodic memory
retrieval
decision
working memory
judgment
maintenance
response selection
belief
familiarity
intention
expectancy
default mode network
context
semantic memory
executive function
navigation
narrative
theory of mind
concept
phonological processing
executive control
autobiographical memory
thought
semantic processing
remembering
attention
inference
meaning
social cognition
mental rotation
decision making
comprehension
consciousness
metaphor
memory encoding
sentence comprehension
short term memory
choice
emotion
recall
humor
search
impulsivity
visual search
mental imagery
reward
emotion regulation
language processing
language
reading
selective attention
spatial attention
eating
risk
object recognition
arousal
empathy
face perception
loss
sustained attention
spatial working memory
fear
consolidation
rehearsal
naming
stress
syntax
action
anticipation
learning
gaze
communication
induction
sleep
listening
visual attention
extinction
prosody
associative memory
integration
anxiety
skill
motor imagery
emotional expression
speech perception
movement
habit
facial expression
speech production
retention
sequence learning
pain
melody
motor control
motor learning

memory

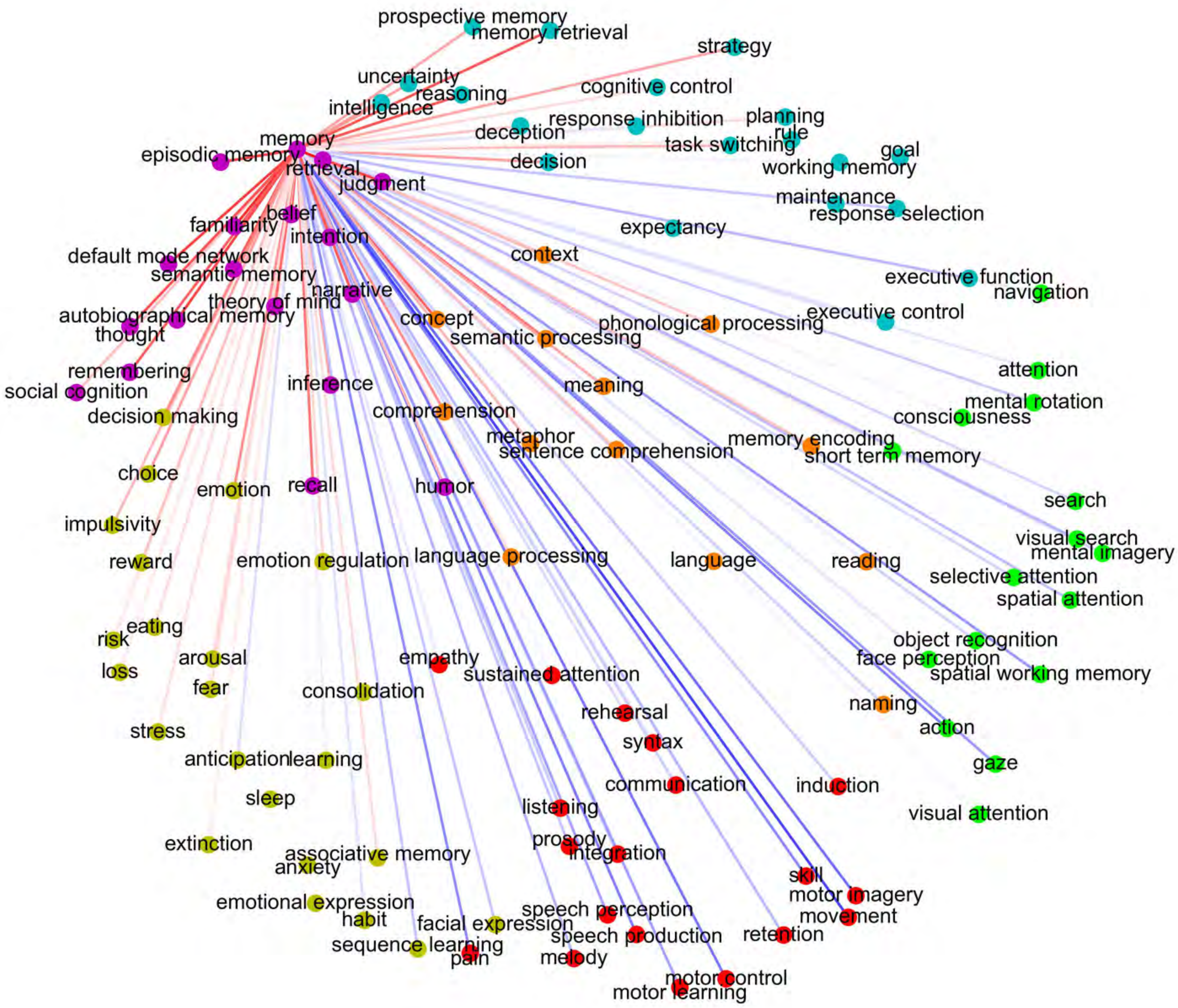

prospective memory
memory retrieval
strategy
uncertainty
reasoning
cognitive control
intelligence
response inhibition
planning
rule
deception
task switching
goal
memory
episodic memory
retrieval
decision
working memory
judgment
maintenance
response selection
belief
familiarity
intention
expectancy
default mode network
context
semantic memory
executive function
navigation
narrative
theory of mind
autobiographical memory
concept
executive control
phonological processing
thought
semantic processing
remembering
attention
social cognition
inference
meaning
mental rotation
decision making
comprehension
consciousness
metaphor
memory encoding
sentence comprehension
short term memory
choice
emotion
recall
humor
search
impulsivity
visual search
mental imagery
reward
emotion regulation
language processing
language
reading
selective attention
spatial attention
eating
risk
arousal
empathy
object recognition
face perception
loss
sustained attention
spatial working memory
fear
consolidation
naming
rehearsal
action
stress
syntax
anticipation
learning
gaze
communication
induction
sleep
listening
visual attention
extinction
prosody
integration
associative memory
anxiety
skill
motor imagery
emotional expression
movement
habit
speech perception
facial expression
speech production
retention
sequence learning
pain
melody
motor control
motor learning

memory retrieval

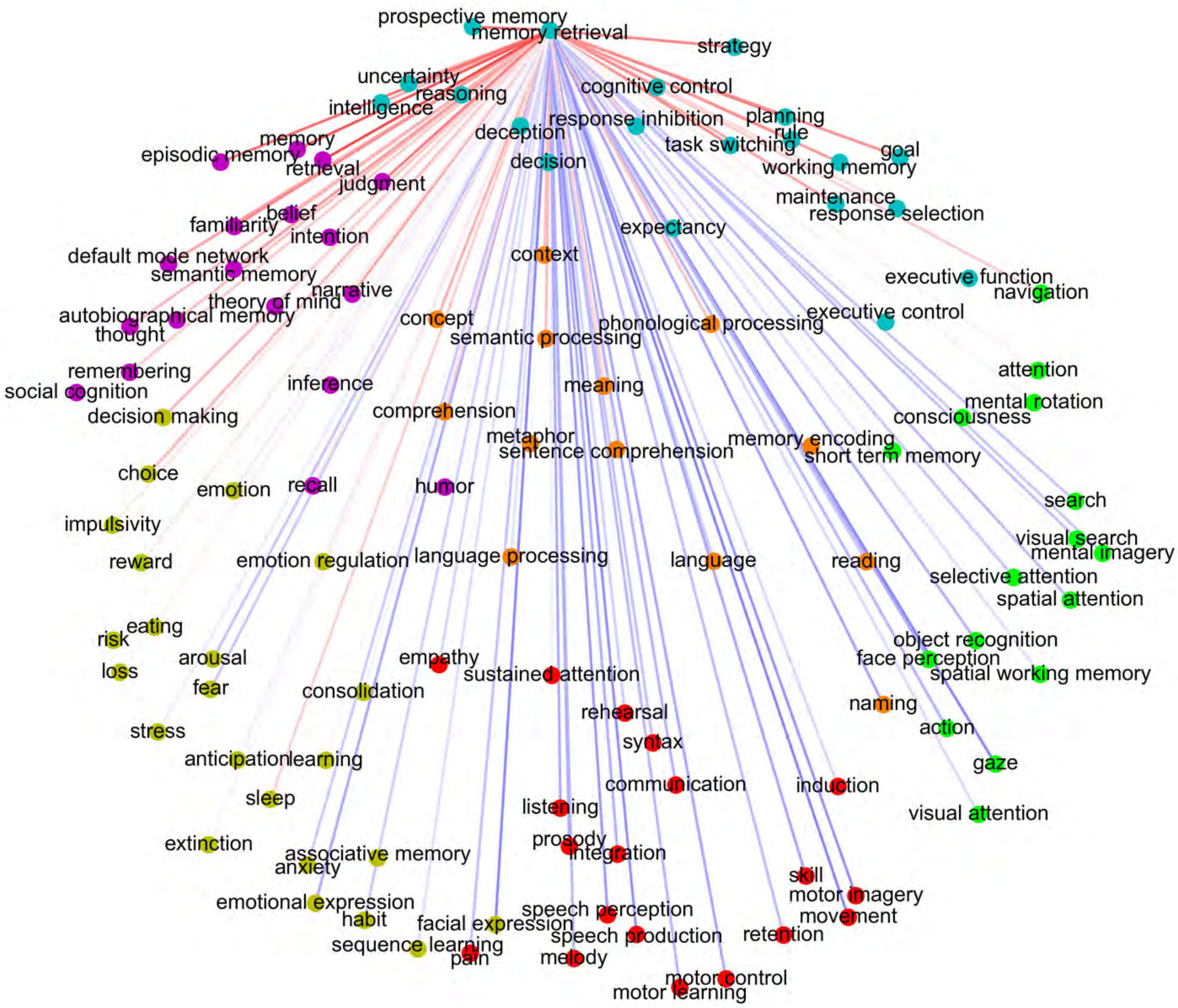

mental imagery

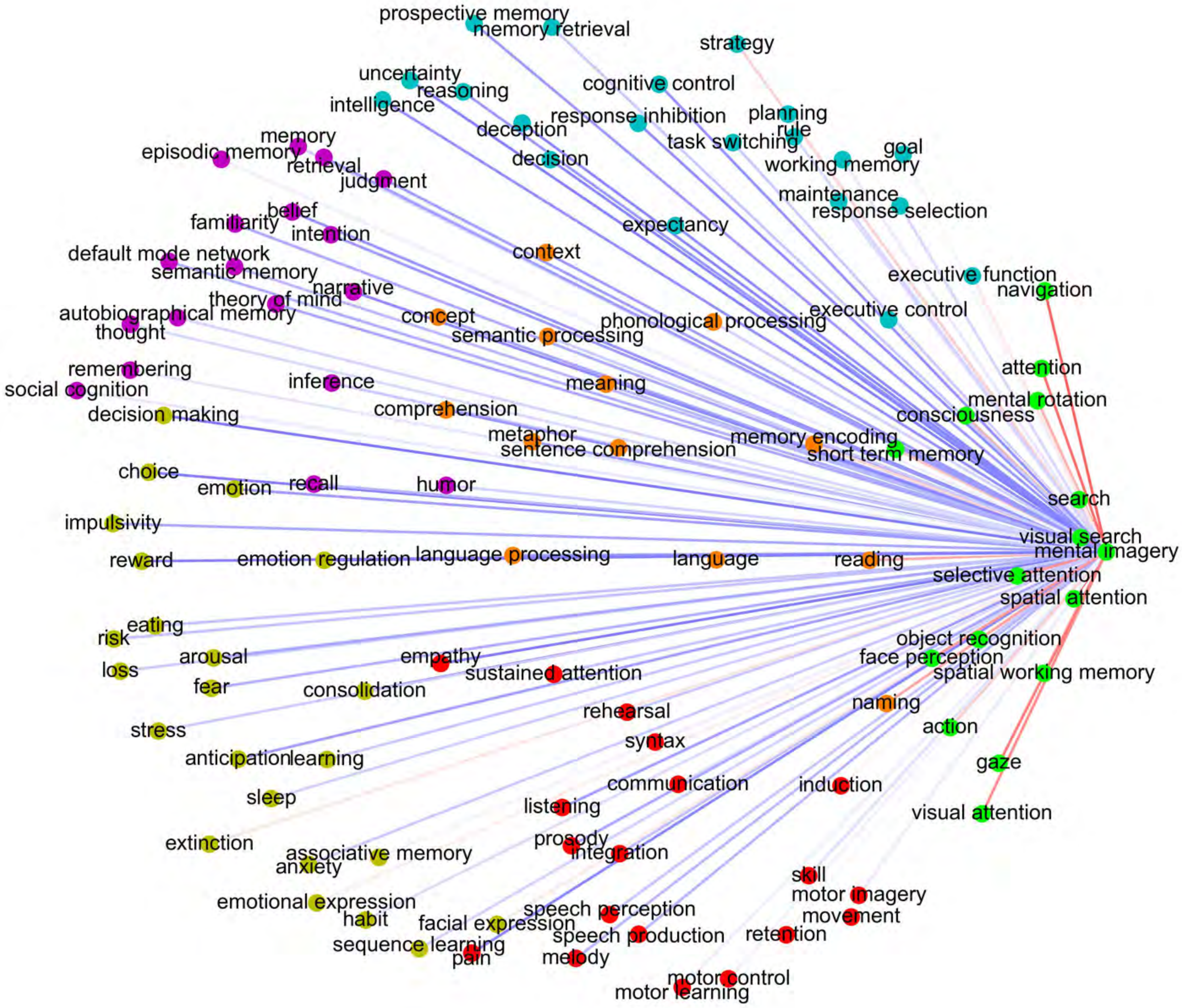

mental rotation

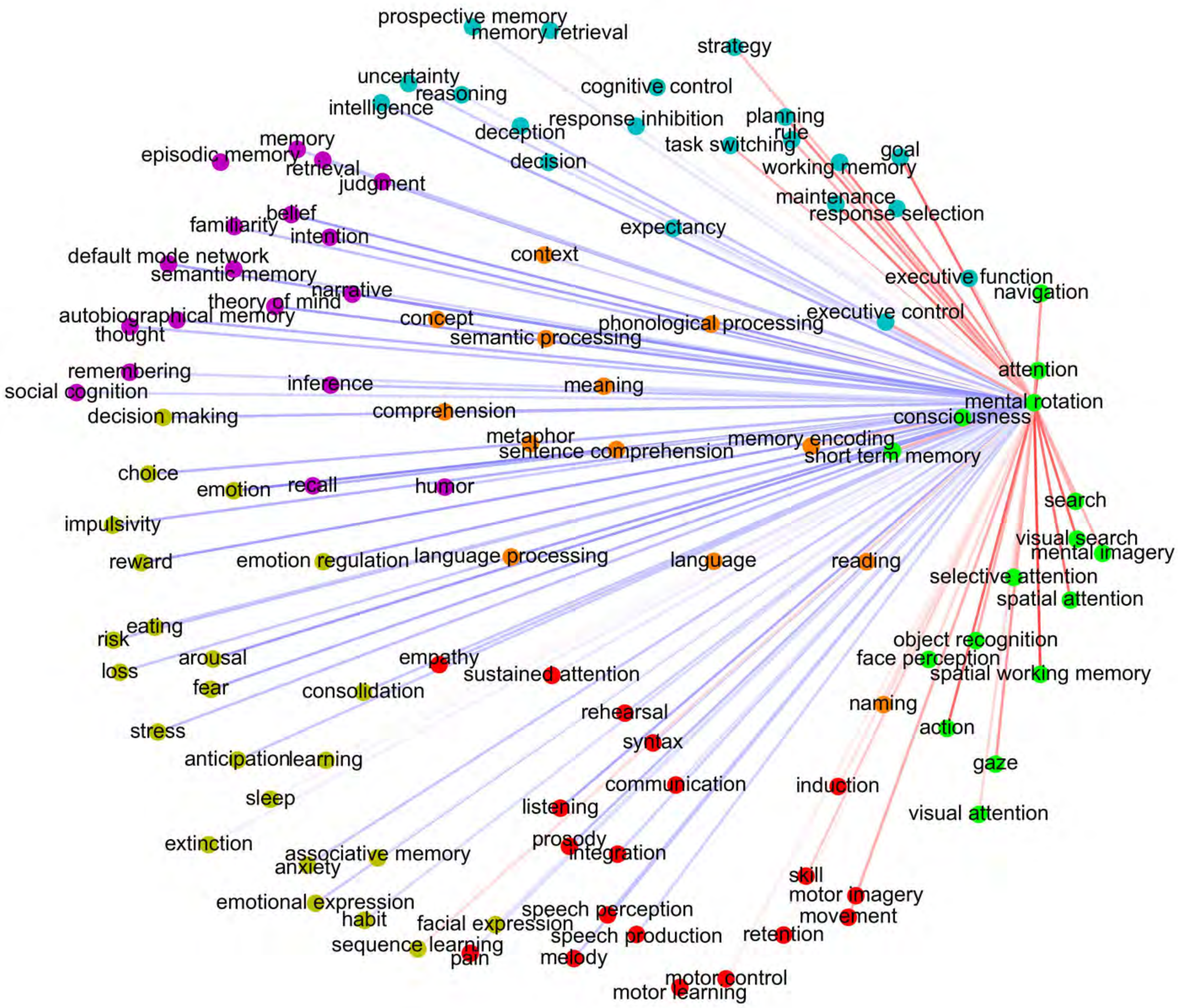

metaphor

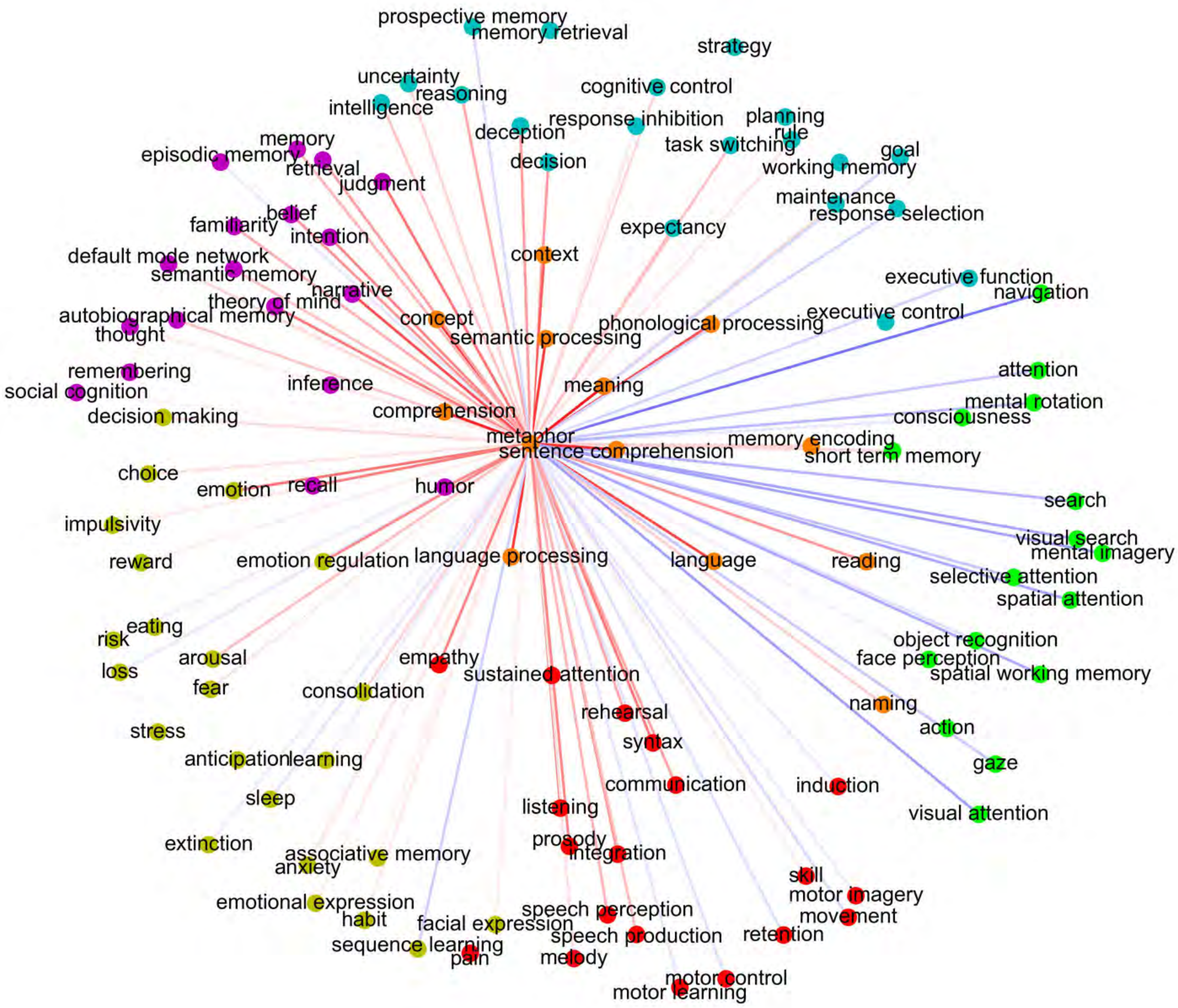

prospective memory
memory retrieval
strategy
uncertainty
reasoning
cognitive control
intelligence
response inhibition
planning
rule
deception
task switching
goal
memory
episodic memory
retrieval
decision
working memory
judgment
maintenance
response selection
belief
familiarity
intention
expectancy
context
default mode network
semantic memory
executive function
navigation
narrative
theory of mind
executive control
concept
phonological processing
autobiographical memory
thought
semantic processing
remembering
attention
social cognition
inference
meaning
mental rotation
decision making
comprehension
consciousness
metaphor
memory encoding
sentence comprehension
short term memory
choice
emotion
recall
humor
search
impulsivity
visual search
mental imagery
reward
emotion regulation
language processing
language
reading
selective attention
spatial attention
risk
eating
object recognition
arousal
empathy
face perception
loss
sustained attention
spatial working memory
fear
consolidation
rehearsal
naming
action
stress
syntax
anticipation
learning
gaze
communication
induction
sleep
listening
visual attention
extinction
prosody
integration
associative memory
anxiety
skill
motor imagery
emotional expression
movement
habit
speech perception
facial expression
speech production
retention
sequence learning
pain
melody
motor control
motor learning

motor control

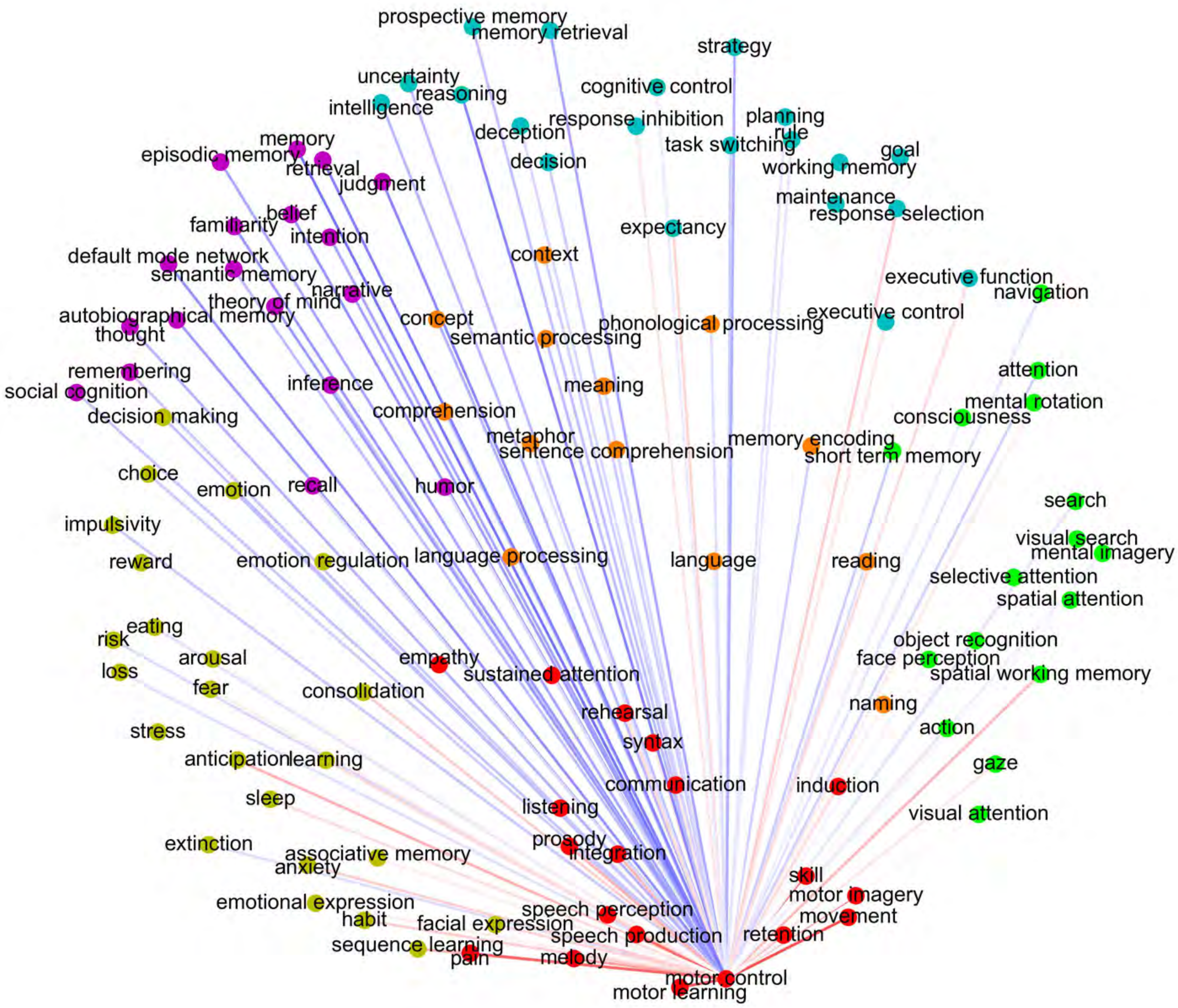

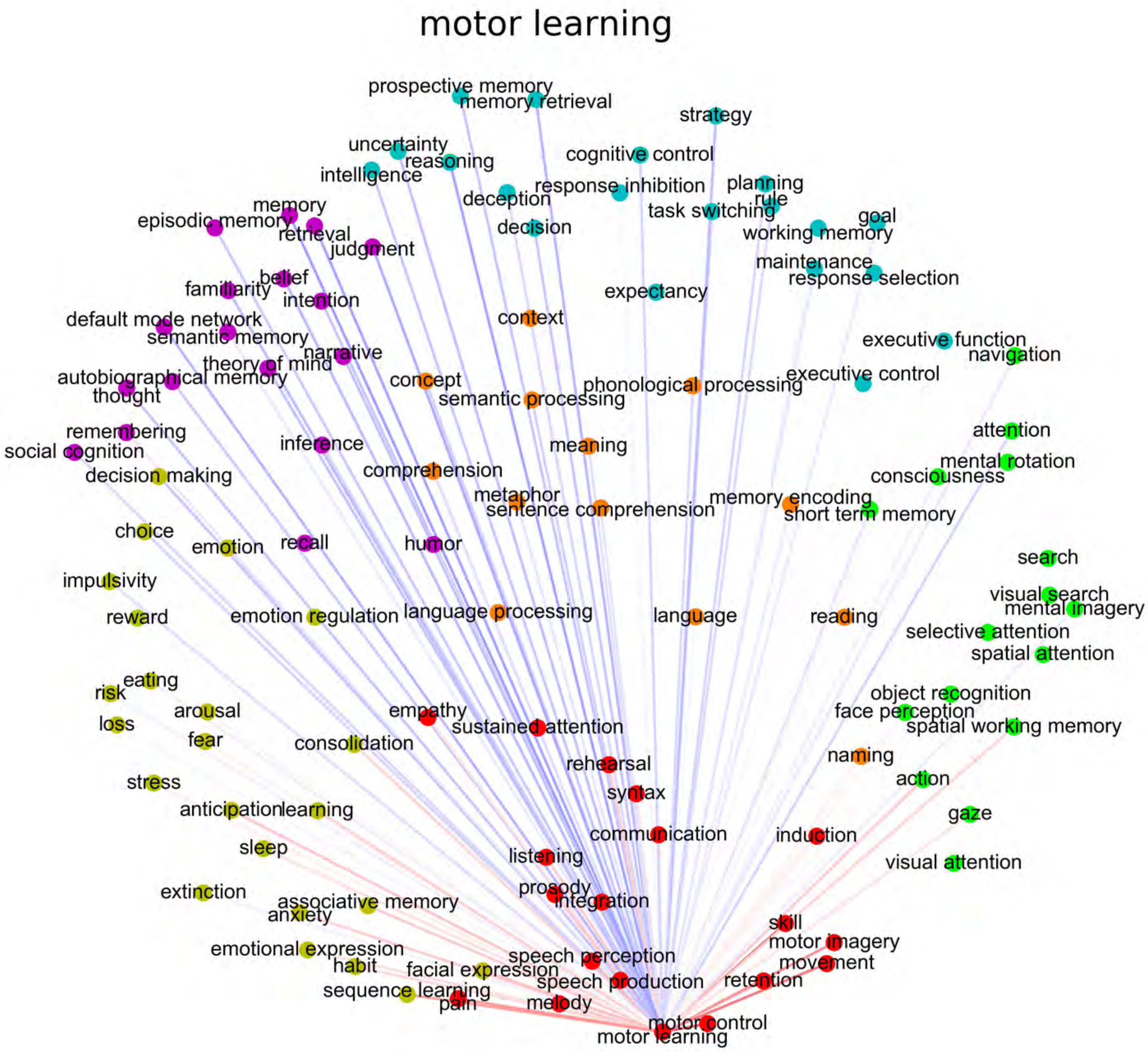
motor learning
prospective memory
memory retrieval
strategy
uncertainty
reasoning
intelligence
cognitive control
response inhibition
planning
rule
deception
task switching
memory
episodic memory
retrieval
decision
goal
working memory
judgment
maintenance
response selection
belief
familiarity
intention
expectancy
context
default mode network
semantic memory
executive function
navigation
narrative
theory of mind
concept
phonological processing
executive control
autobiographical memory
thought
semantic processing
remembering
social cognition
inference
meaning
attention
mental rotation
decision making
comprehension
consciousness
metaphor
sentence comprehension
memory encoding
short term memory
choice
emotion
recall
humor
search
impulsivity
visual search
mental imagery
reward
emotion regulation
language processing
language
reading
selective attention
spatial attention
eating
risk
arousal
object recognition
empathy
face perception
loss
sustained attention
spatial working memory
fear
consolidation
rehearsal
naming
stress
syntax
action
anticipation
learning
gaze
communication
induction
sleep
listening
visual attention
extinction
prosody
integration
associative memory
anxiety
skill
motor imagery
emotional expression
speech perception
movement
habit
facial expression
speech production
retention
sequence learning
pain
melody
motor control
motor learning

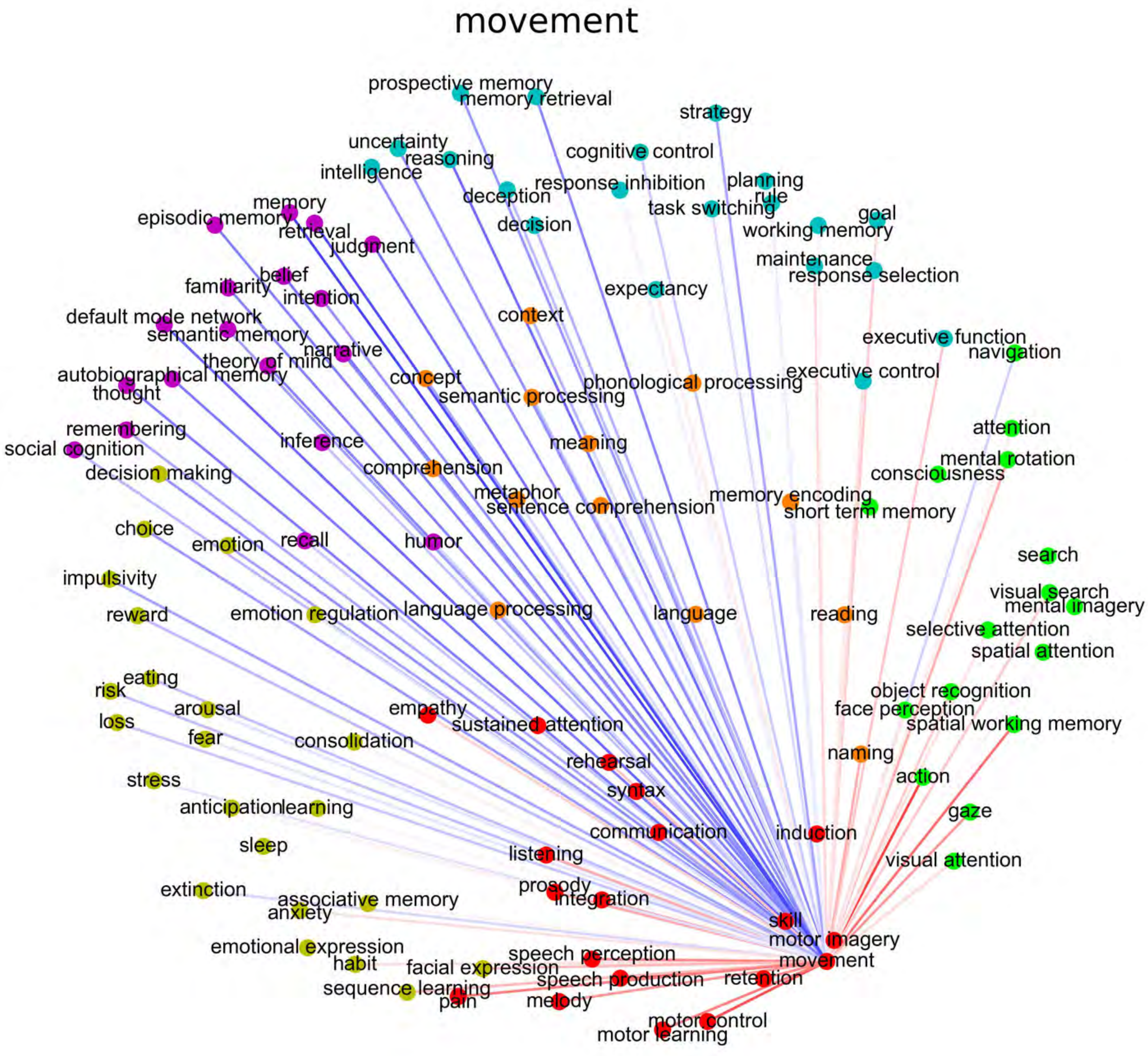
movement
prospective memory
memory retrieval
strategy
uncertainty
reasoning
cognitive control
intelligence
response inhibition
planning
rule
deception
task switching
goal
memory
episodic memory
decision
working memory
retrieval
judgment
maintenance
response selection
belief
familiarity
intention
expectancy
context
default mode network
semantic memory
executive function
navigation
narrative
theory of mind
concept
executive control
autobiographical memory
phonological processing
thought
semantic processing
attention
remembering
inference
meaning
mental rotation
social cognition
comprehension
consciousness
decision making
metaphor
memory encoding
sentence comprehension
short term memory
choice
emotion
recall
humor
search
impulsivity
visual search
mental imagery
reward
emotion regulation
language processing
language
reading
selective attention
spatial attention
eating
risk
object recognition
arousal
empathy
face perception
loss
sustained attention
spatial working memory
fear
consolidation
rehearsal
naming
stress
syntax
action
anticipation
learning
gaze
sleep
communication
induction
listening
visual attention
extinction
prosody
integration
associative memory
anxiety
skill
motor imagery
emotional expression
speech perception
habit
facial expression
speech production
retention
sequence learning
pain
melody
motor control
motor learning

naming

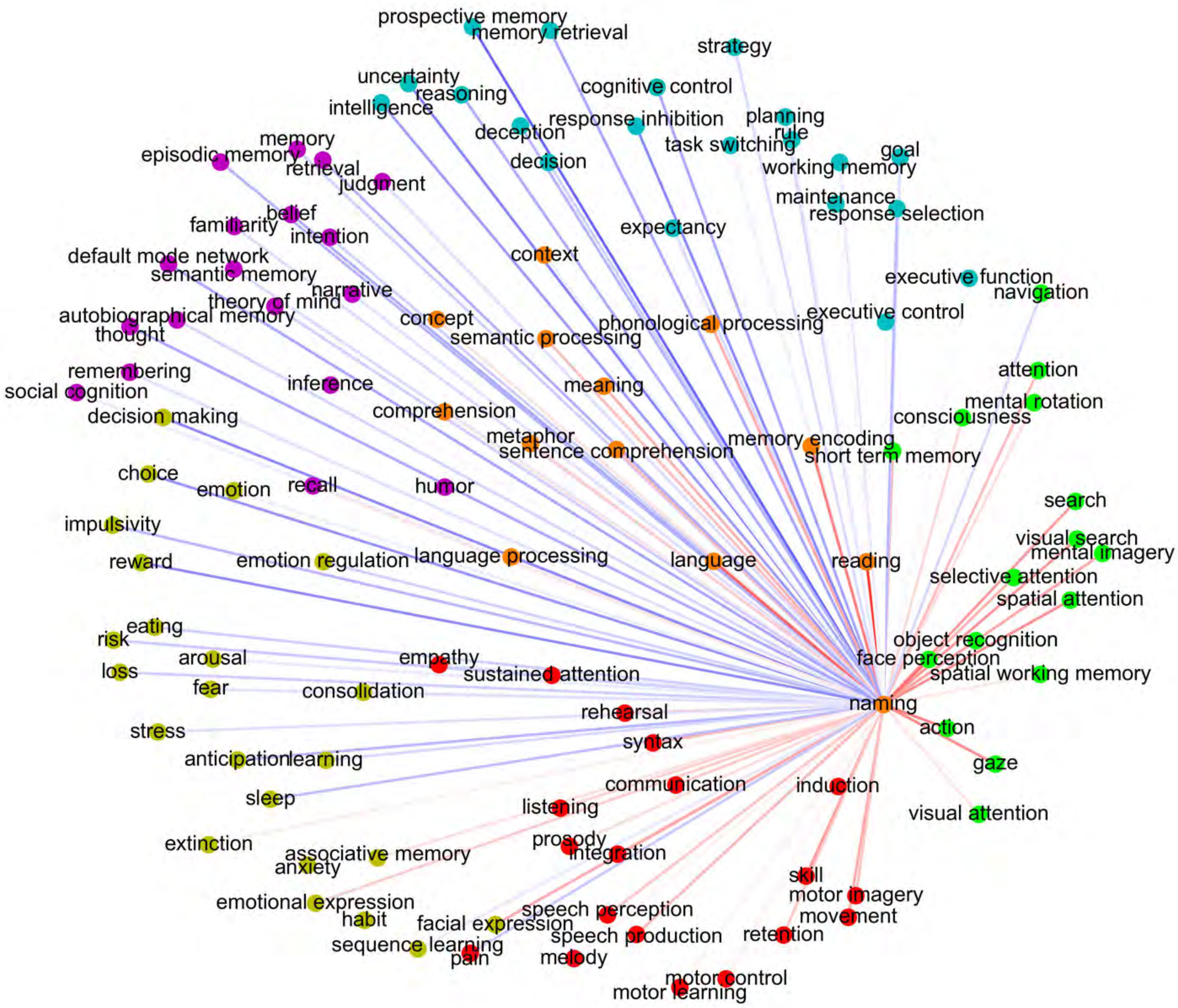
prospective memory
memory retrieval
strategy
uncertainty
reasoning
cognitive control
intelligence
response inhibition
planning
rule
deception
task switching
goal
memory
episodic memory
retrieval
decision
working memory
judgment
maintenance
response selection
belief
familiarity
intention
expectancy
context
default mode network
semantic memory
executive function
navigation
narrative
theory of mind
executive control
autobiographical memory
concept
phonological processing
thought
semantic processing
remembering
attention
inference
meaning
social cognition
mental rotation
decision making
comprehension
consciousness
metaphor
memory encoding
sentence comprehension
short term memory
choice
emotion
recall
humor
search
impulsivity
visual search
mental imagery
reward
emotion regulation
language processing
language
reading
selective attention
spatial attention
eating
risk
object recognition
arousal
empathy
face perception
loss
sustained attention
spatial working memory
fear
consolidation
naming
rehearsal
stress
action
syntax
anticipation
learning
gaze
communication
induction
sleep
listening
visual attention
extinction
prosody
associative memory
integration
anxiety
skill
motor imagery
emotional expression
speech perception
habit
facial expression
movement
speech production
retention
sequence learning
pain
melody
motor control
motor learning

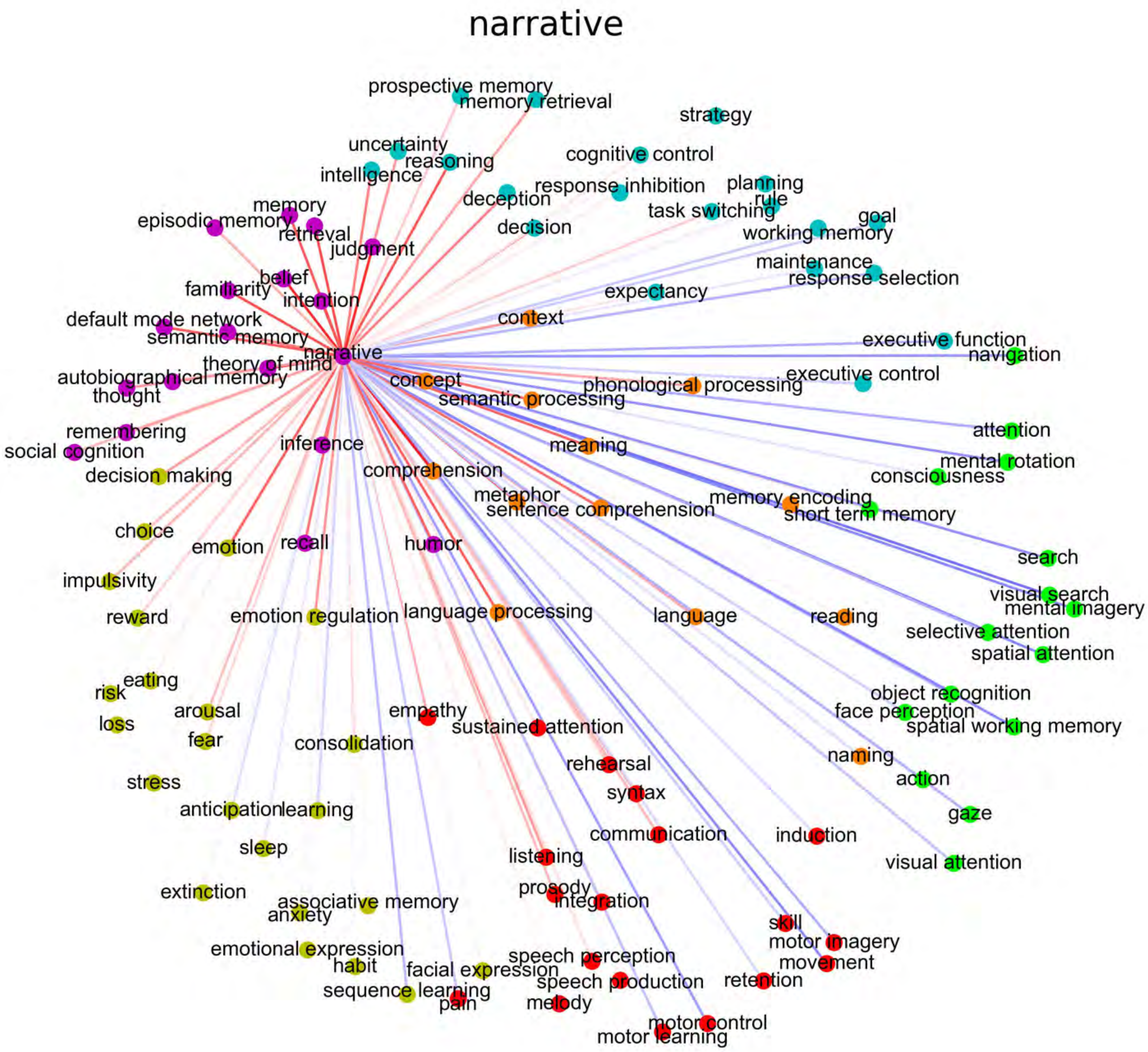
narrative
prospective memory
memory retrieval
strategy
uncertainty
reasoning
cognitive control
intelligence
response inhibition
planning
rule
deception
task switching
goal
memory
episodic memory
retrieval
decision
working memory
judgment
maintenance
response selection
belief
familiarity
intention
expectancy
default mode network
context
semantic memory
executive function
navigation
theory of mind
executive control
autobiographical memory
concept
phonological processing
thought
semantic processing
remembering
attention
social cognition
inference
meaning
mental rotation
decision making
comprehension
consciousness
metaphor
memory encoding
sentence comprehension
short term memory
choice
emotion
recall
humor
search
impulsivity
visual search
mental imagery
reward
emotion regulation
language processing
language
reading
selective attention
spatial attention
risk
eating
object recognition
arousal
empathy
face perception
loss
sustained attention
spatial working memory
fear
consolidation
naming
rehearsal
action
stress
syntax
anticipation
learning
gaze
communication
induction
sleep
listening
visual attention
extinction
prosody
integration
associative memory
anxiety
skill
motor imagery
emotional expression
speech perception
movement
habit
facial expression
speech production
retention
sequence learning
pain
melody
motor control
motor learning

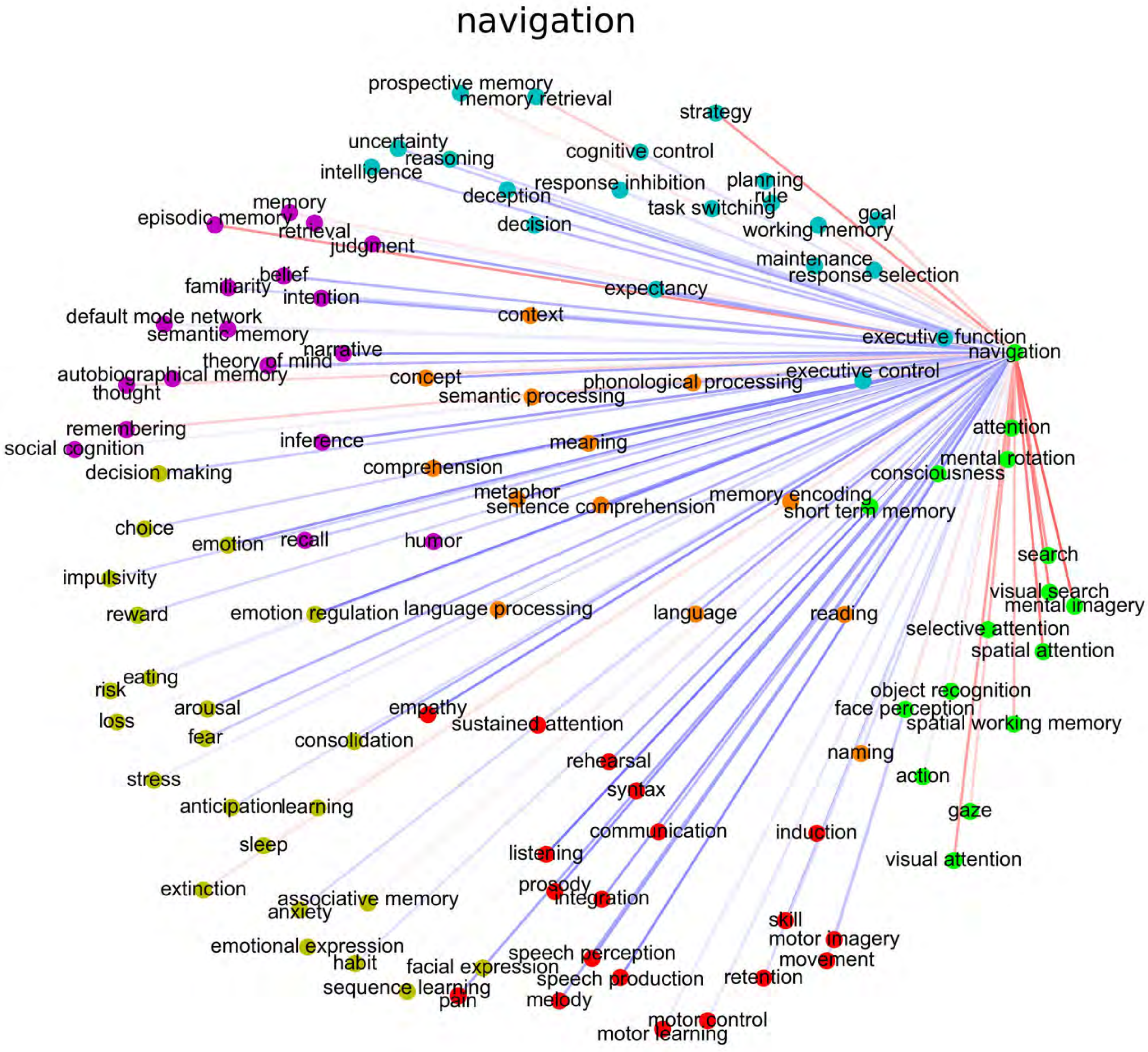
navigation
prospective memory
memory retrieval
strategy
uncertainty
reasoning
cognitive control
intelligence
response inhibition
planning
rule
deception
memory
task switching
goal
episodic memory
retrieval
decision
working memory
judgment
maintenance
response selection
belief
familiarity
intention
expectancy
default mode network
context
semantic memory
executive function
navigation
narrative
theory of mind
executive control
autobiographical memory
concept
phonological processing
thought
semantic processing
remembering
attention
inference
meaning
social cognition
mental rotation
decision making
comprehension
consciousness
metaphor
memory encoding
sentence comprehension
short term memory
choice
emotion
recall
humor
search
impulsivity
visual search
mental imagery
reward
emotion regulation
language processing
language
reading
selective attention
spatial attention
eating
risk
object recognition
arousal
empathy
face perception
loss
sustained attention
spatial working memory
fear
consolidation
rehearsal
naming
stress
syntax
action
anticipation
learning
gaze
communication
induction
sleep
listening
visual attention
extinction
prosody
integration
associative memory
anxiety
skill
motor imagery
emotional expression
speech perception
habit
facial expression
movement
speech production
retention
sequence learning
pain
melody
motor control
motor learning

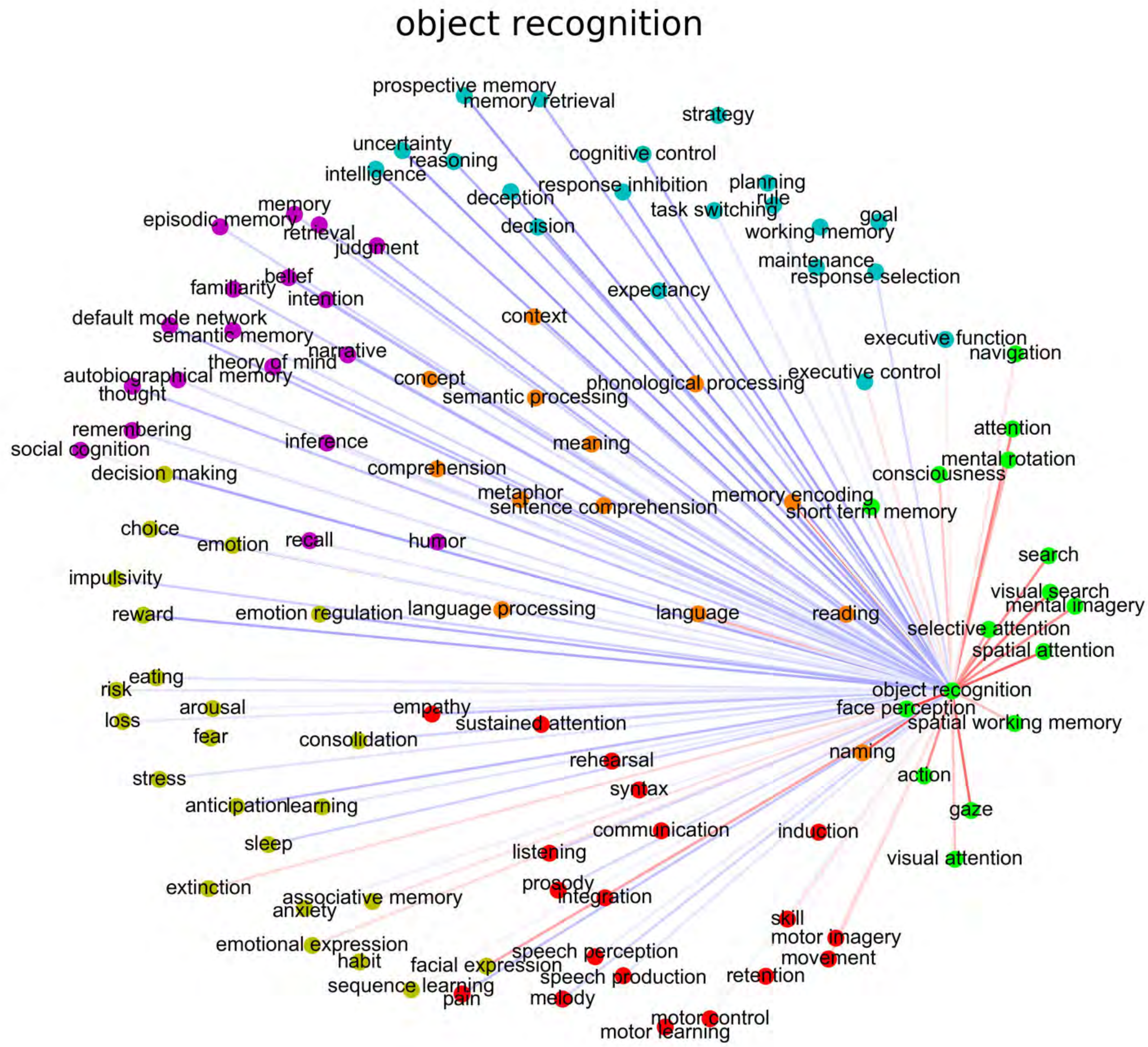
object recognition
prospective memory
memory retrieval
strategy
uncertainty
reasoning
cognitive control
intelligence
response inhibition
planning
rule
deception
task switching
goal
memory
episodic memory
retrieval
decision
working memory
judgment
maintenance
response selection
belief
familiarity
intention
expectancy
default mode network
context
semantic memory
executive function
navigation
narrative
theory of mind
concept
phonological processing
executive control
autobiographical memory
thought
semantic processing
remembering
attention
inference
meaning
social cognition
mental rotation
comprehension
consciousness
decision making
metaphor
memory encoding
sentence comprehension
short term memory
choice
emotion
recall
humor
search
impulsivity
visual search
mental imagery
reward
emotion regulation
language processing
language
reading
selective attention
spatial attention
eating
risk
object recognition
arousal
empathy
face perception
loss
sustained attention
spatial working memory
fear
consolidation
naming
rehearsal
stress
action
syntax
anticipation
learning
gaze
communication
induction
sleep
listening
visual attention
extinction
prosody
integration
associative memory
anxiety
skill
motor imagery
emotional expression
habit
speech perception
movement
facial expression
speech production
retention
sequence learning
pain
melody
motor control
motor learning

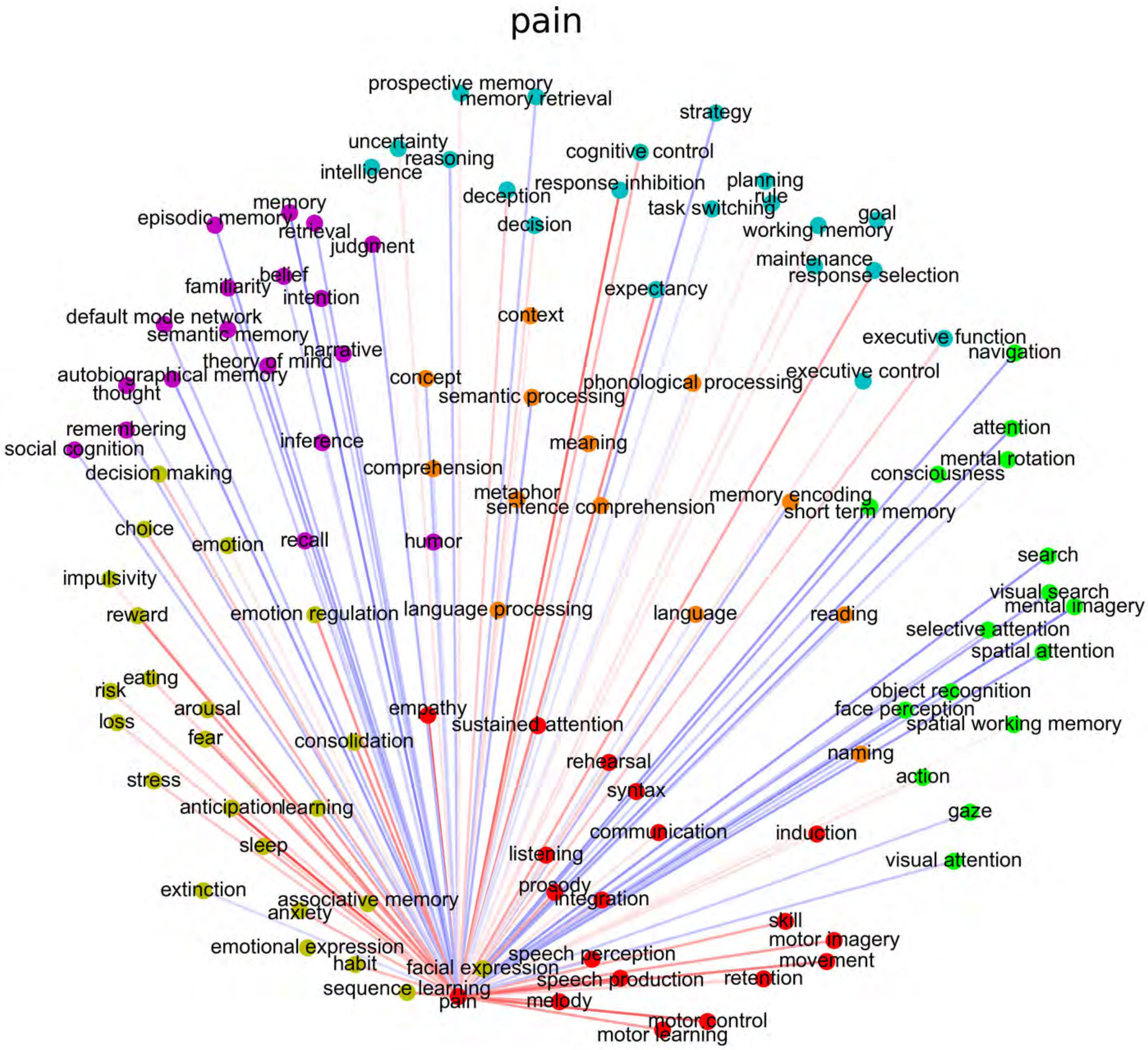
pain
prospective memory
memory retrieval
strategy
uncertainty
reasoning
intelligence
cognitive control
response inhibition
planning
rule
deception
task switching
goal
memory
episodic memory
retrieval
decision
working memory
judgment
maintenance
response selection
belief
familiarity
intention
expectancy
default mode network
semantic memory
context
executive function
navigation
narrative
theory of mind
concept
phonological processing
executive control
autobiographical memory
thought
semantic processing
remembering
social cognition
inference
meaning
attention
decision making
comprehension
mental rotation
consciousness
metaphor
sentence comprehension
memory encoding
short term memory
choice
emotion
recall
humor
search
impulsivity
visual search
mental imagery
reward
emotion regulation
language processing
language
reading
selective attention
spatial attention
eating
risk
arousal
object recognition
loss
empathy
face perception
fear
sustained attention
spatial working memory
consolidation
naming
rehearsal
stress
action
syntax
anticipation
learning
gaze
sleep
communication
induction
listening
visual attention
extinction
prosody
integration
associative memory
anxiety
skill
motor imagery
emotional expression
speech perception
movement
habit
facial expression
speech production
retention
sequence learning
pain
melody
motor control
motor learning

phonological processing

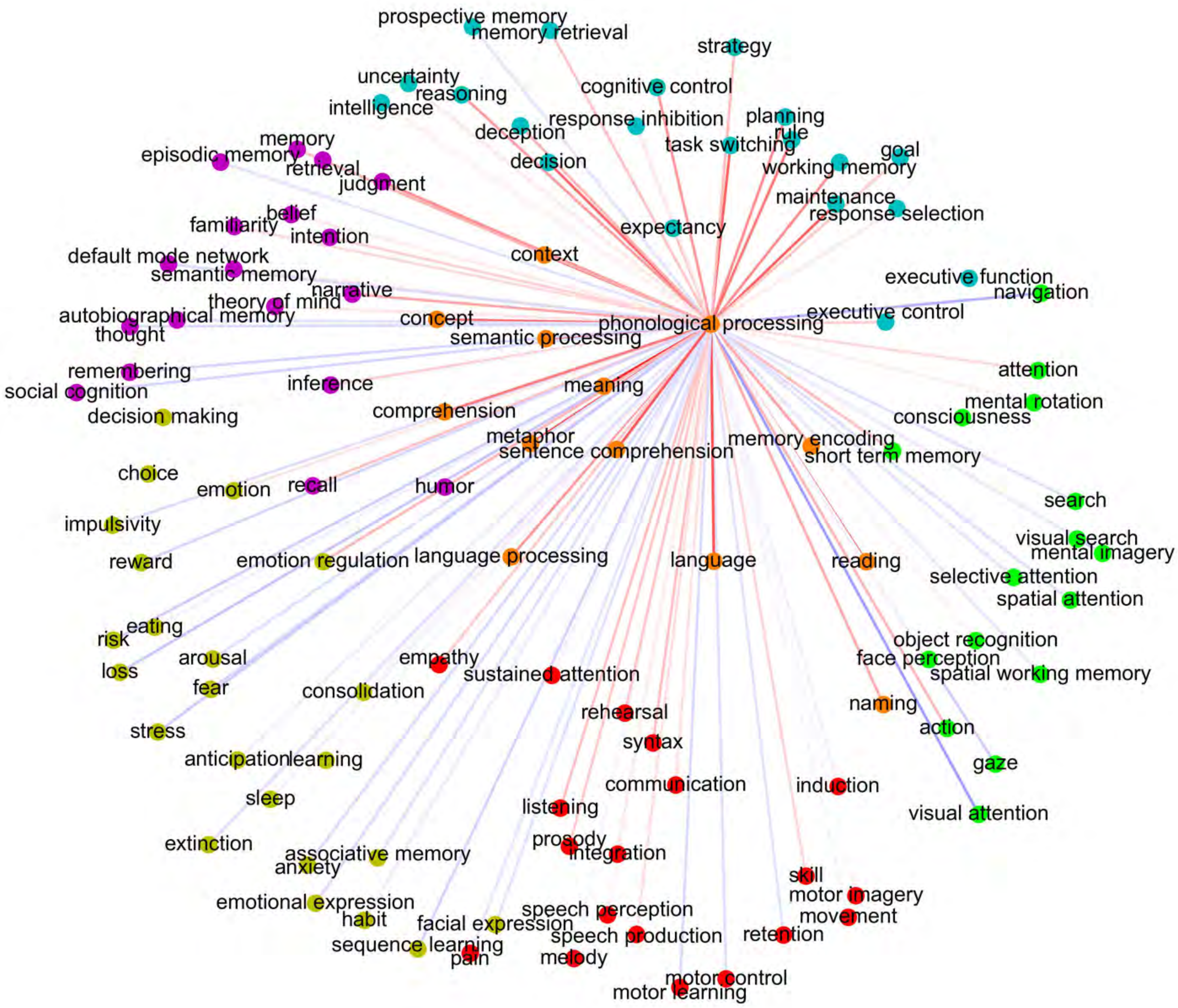

planning

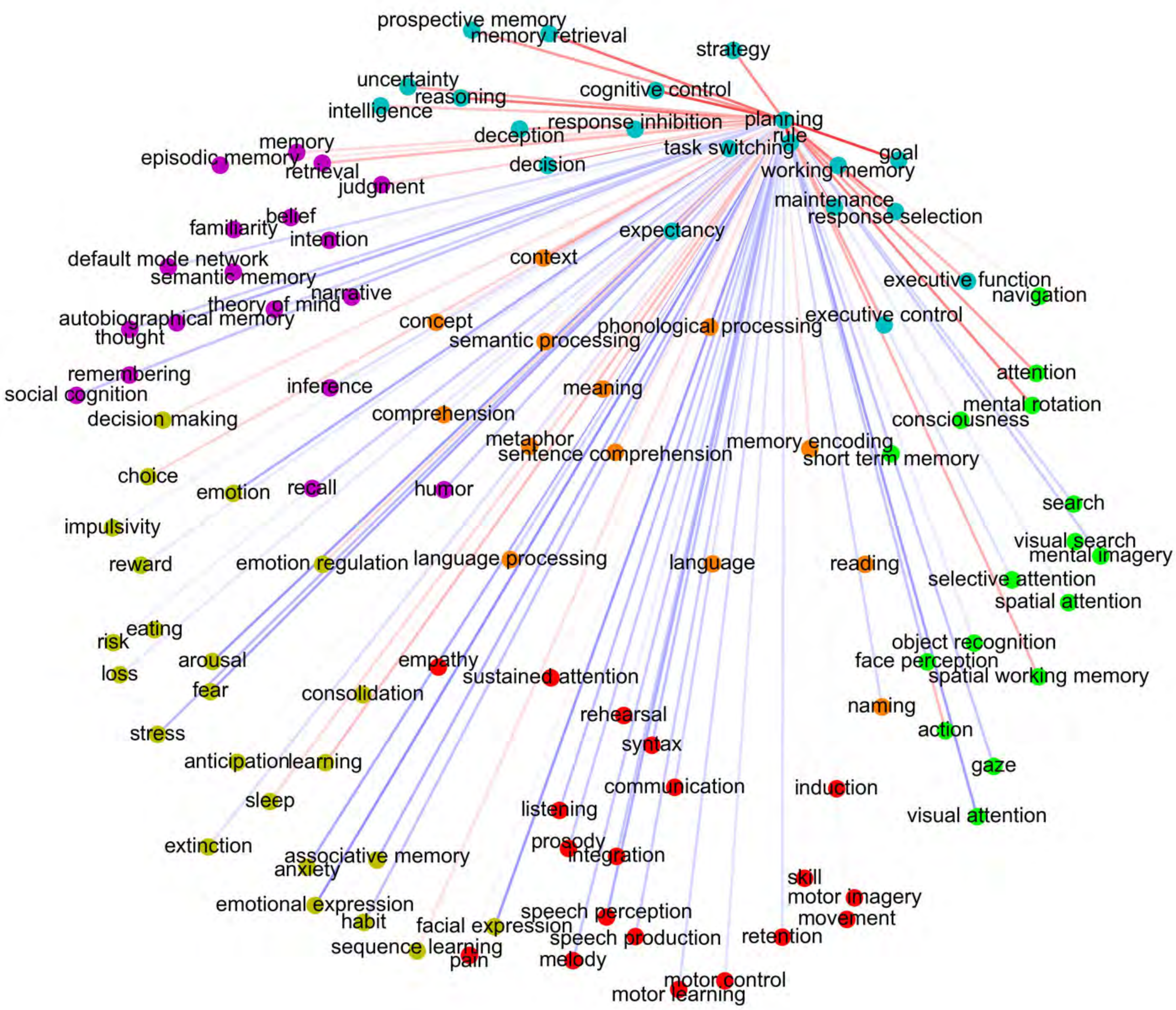
prospective memory
memory retrieval
strategy
uncertainty
reasoning
cognitive control
intelligence
planning
response inhibition
deception
rule
memory
episodic memory
retrieval
task switching
goal
decision
working memory
judgment
maintenance
response selection
belief
familiarity
intention
expectancy
context
default mode network
semantic memory
executive function
navigation
narrative
theory of mind
autobiographical memory
concept
phonological processing
executive control
thought
semantic processing
remembering
attention
social cognition
inference
meaning
decision making
comprehension
mental rotation
consciousness
metaphor
memory encoding
sentence comprehension
short term memory
choice
emotion
recall
humor
search
impulsivity
visual search
mental imagery
reward
emotion regulation
language processing
language
reading
selective attention
spatial attention
risk
eating
object recognition
arousal
empathy
face perception
loss
sustained attention
spatial working memory
fear
consolidation
rehearsal
naming
action
stress
syntax
anticipation
learning
gaze
sleep
communication
induction
listening
visual attention
extinction
prosody
integration
associative memory
anxiety
skill
motor imagery
emotional expression
speech perception
habit
movement
facial expression
speech production
retention
sequence learning
pain
melody
motor control
motor learning

prosody

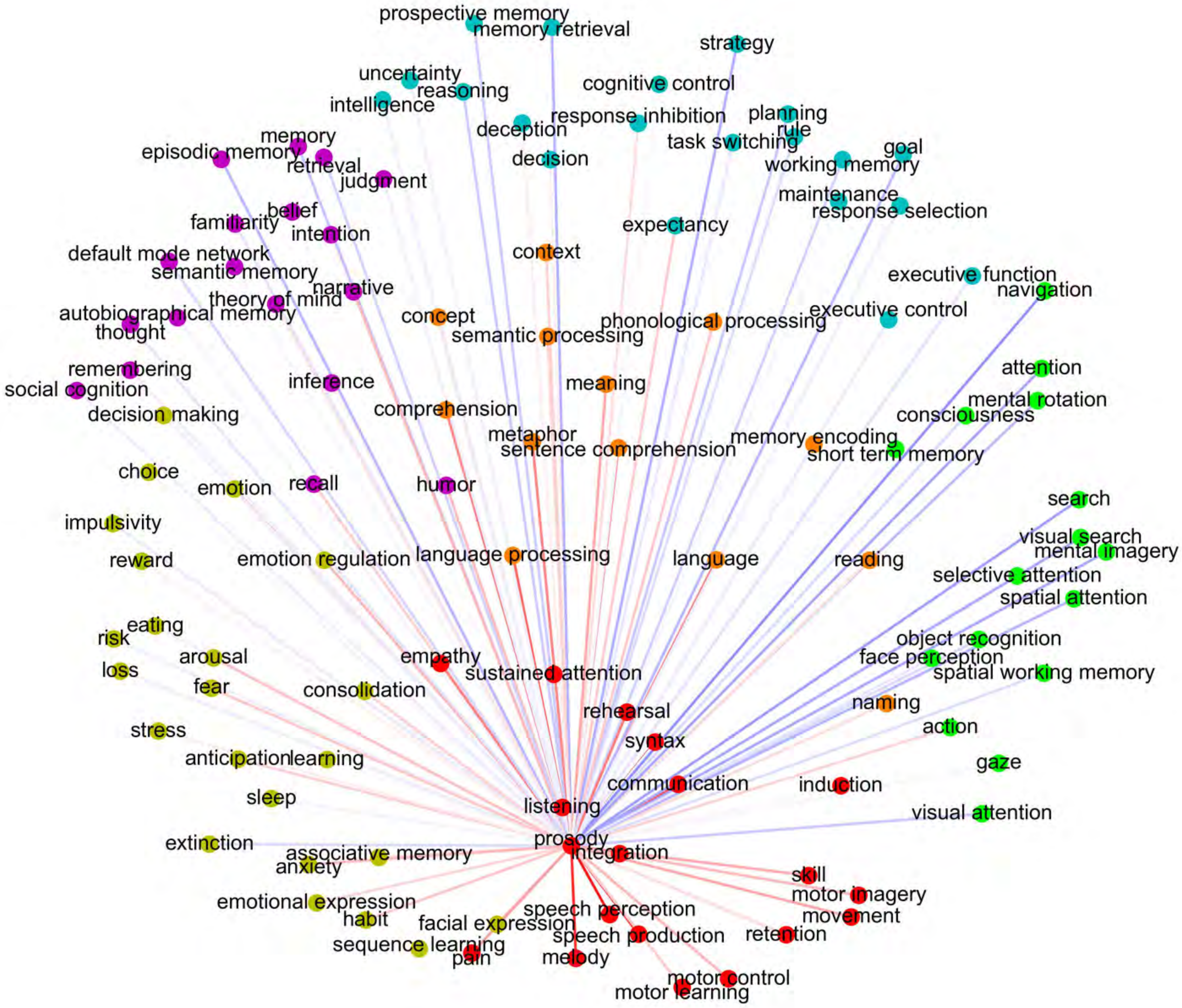

prospective memory
memory retrieval
strategy
uncertainty
reasoning
cognitive control
intelligence
response inhibition
planning
deception
rule
task switching
memory
episodic memory
retrieval
decision
goal
working memory
judgment
maintenance
response selection
belief
familiarity
intention
expectancy
default mode network
context
semantic memory
executive function
navigation
narrative
theory of mind
concept
phonological processing
executive control
autobiographical memory
semantic processing
thought
remembering
attention
meaning
social cognition
inference
mental rotation
decision making
comprehension
consciousness
metaphor
memory encoding
sentence comprehension
short term memory
choice
emotion
recall
humor
search
impulsivity
visual search
mental imagery
reward
emotion regulation
language processing
language
reading
selective attention
spatial attention
eating
risk
object recognition
arousal
empathy
face perception
loss
sustained attention
spatial working memory
fear
consolidation
naming
rehearsal
stress
action
syntax
anticipation
learning
gaze
communication
induction
sleep
listening
visual attention
extinction
prosody
integration
associative memory
anxiety
skill
motor imagery
emotional expression
movement
habit
speech perception
facial expression
speech production
retention
sequence learning
pain
melody
motor control
motor learning

prospective memory

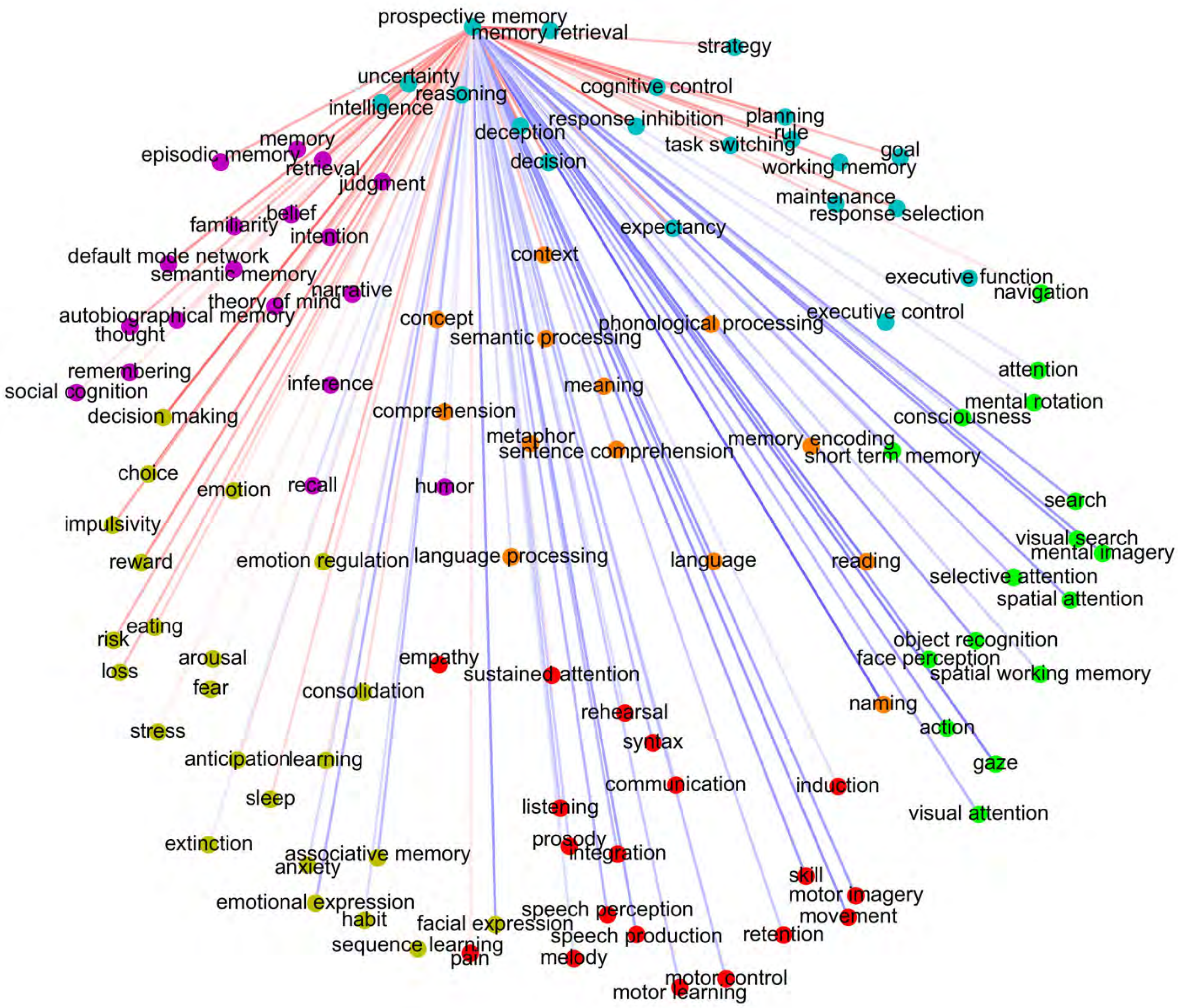

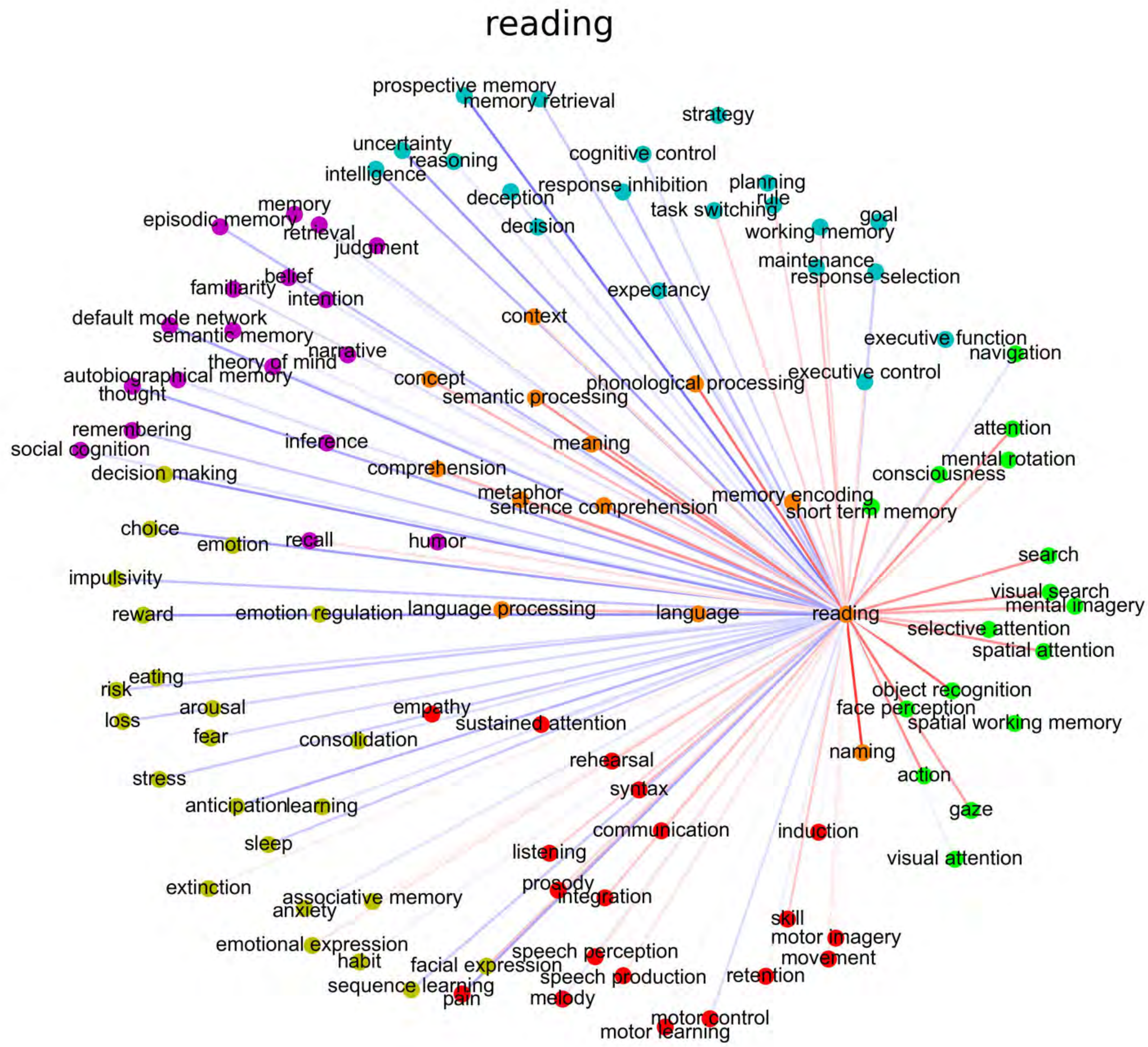
reading
prospective memory
memory retrieval
strategy
uncertainty
reasoning
cognitive control
intelligence
response inhibition
planning
rule
deception
task switching
goal
memory
episodic memory
retrieval
decision
working memory
judgment
maintenance
response selection
belief
familiarity
intention
expectancy
default mode network
context
semantic memory
executive function
narrative
navigation
theory of mind
concept
phonological processing
executive control
autobiographical memory
thought
semantic processing
remembering
attention
inference
meaning
social cognition
mental rotation
decision making
comprehension
consciousness
metaphor
memory encoding
sentence comprehension
short term memory
choice
emotion
recall
humor
search
impulsivity
visual search
mental imagery
reward
emotion regulation
language processing
language
selective attention
spatial attention
eating
risk
object recognition
arousal
empathy
face perception
loss
sustained attention
spatial working memory
fear
consolidation
naming
rehearsal
stress
action
syntax
anticipation
learning
gaze
communication
induction
sleep
listening
visual attention
extinction
prosody
integration
associative memory
anxiety
skill
motor imagery
emotional expression
habit
speech perception
movement
facial expression
speech production
retention
sequence learning
pain
melody
motor control
motor learning

reasoning

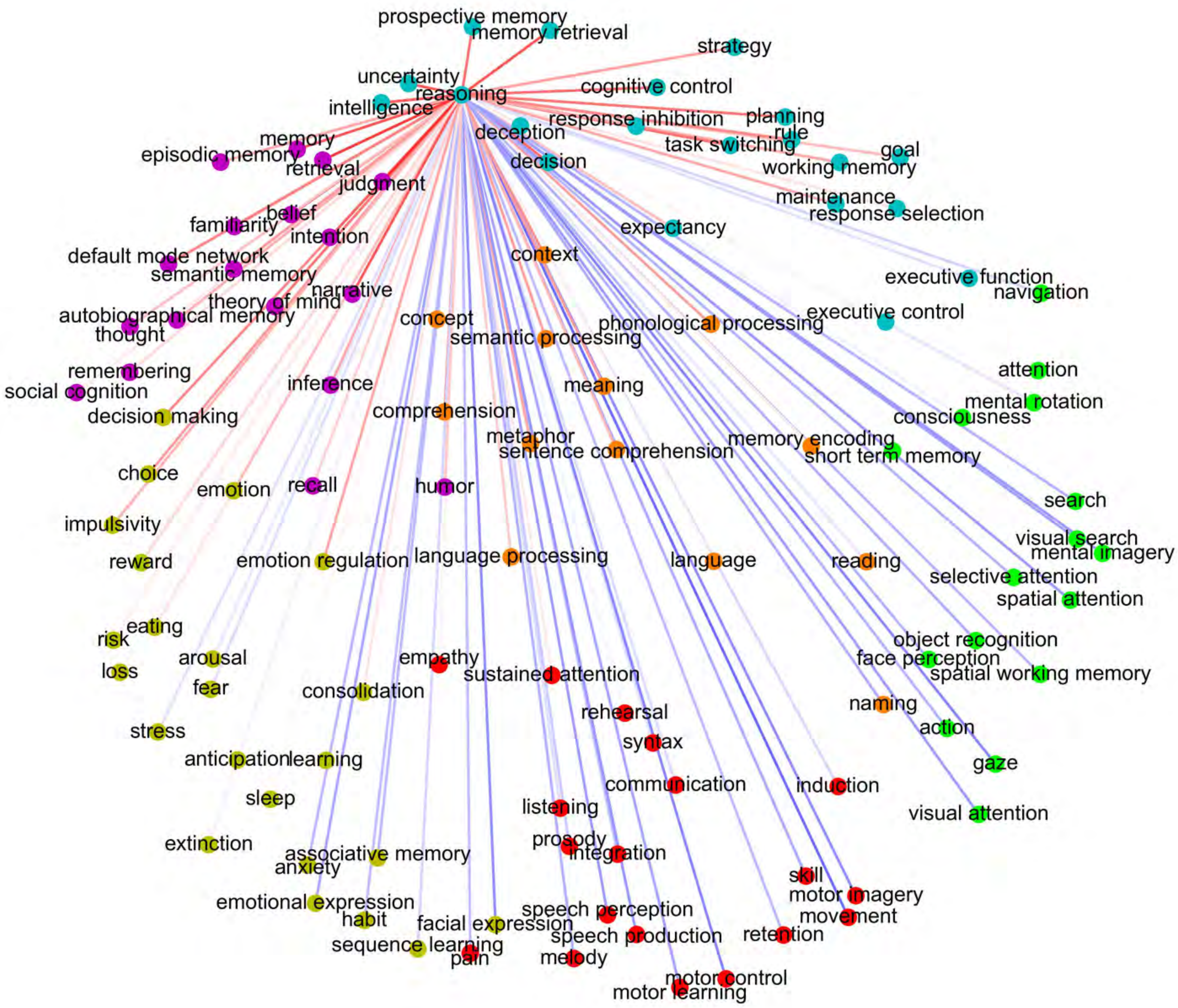
reasoning
prospective memory
memory retrieval
strategy
uncertainty
cognitive control
intelligence
planning
response inhibition
rule
deception
task switching
goal
memory
episodic memory
retrieval
decision
working memory
judgment
maintenance
response selection
belief
familiarity
intention
expectancy
default mode network
context
semantic memory
executive function
navigation
narrative
theory of mind
concept
executive control
autobiographical memory
phonological processing
thought
semantic processing
remembering
attention
inference
meaning
social cognition
mental rotation
decision making
comprehension
consciousness
metaphor
memory encoding
sentence comprehension
short term memory
choice
emotion
recall
humor
search
impulsivity
visual search
mental imagery
reward
emotion regulation
language processing
language
reading
selective attention
spatial attention
eating
risk
object recognition
arousal
empathy
face perception
loss
sustained attention
spatial working memory
fear
consolidation
rehearsal
naming
stress
syntax
action
anticipation
learning
gaze
communication
induction
sleep
listening
visual attention
extinction
prosody
integration
associative memory
anxiety
skill
motor imagery
emotional expression
speech perception
movement
habit
facial expression
speech production
retention
sequence learning
pain
melody
motor control
motor learning

recall

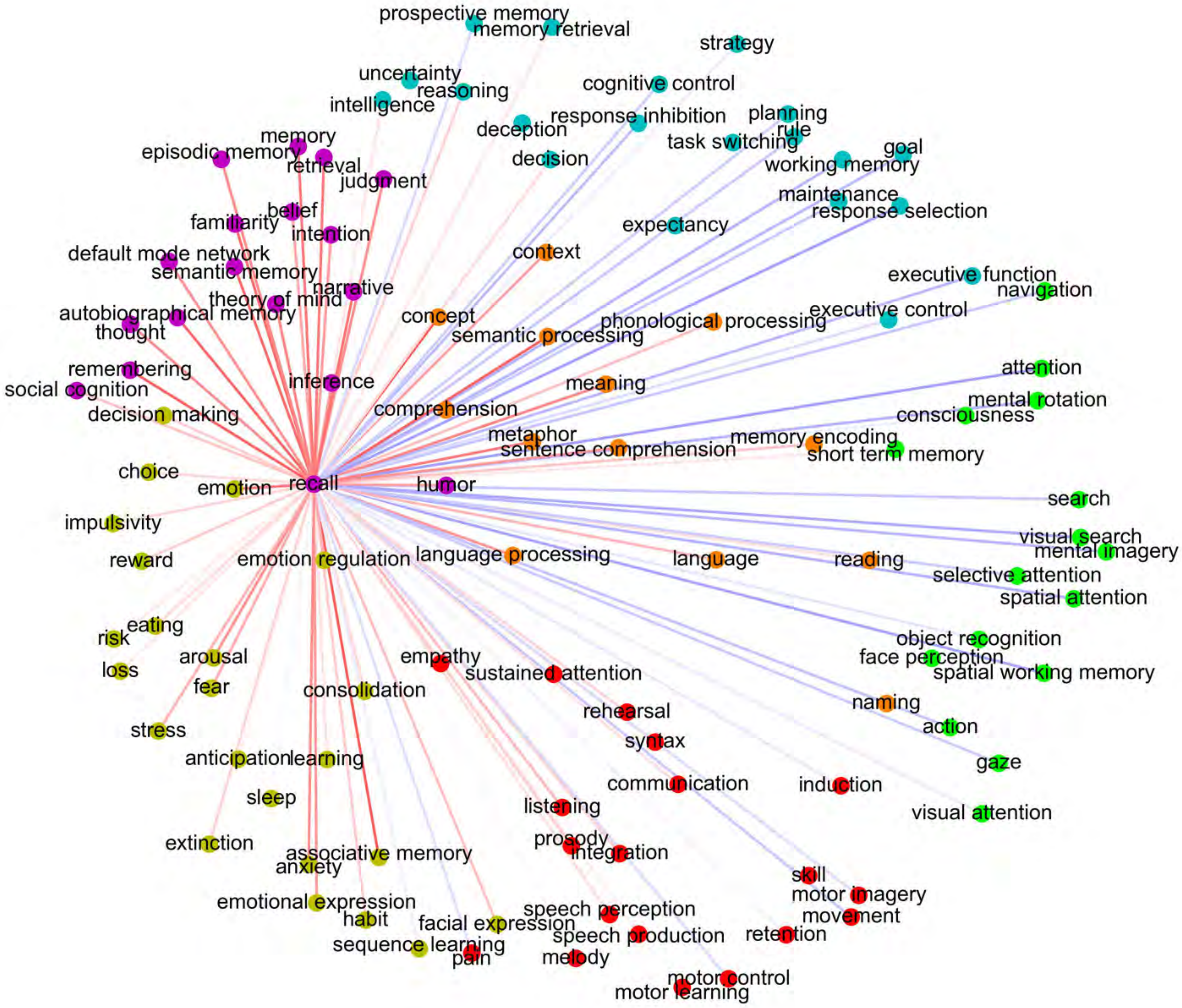
prospective memory
memory retrieval
strategy
uncertainty
reasoning
cognitive control
intelligence
deception
response inhibition
planning
rule
task switching
memory
episodic memory
retrieval
decision
goal
working memory
judgment
maintenance
response selection
belief
familiarity
intention
expectancy
default mode network
semantic memory
context
executive function
navigation
narrative
theory of mind
concept
executive control
autobiographical memory
thought
semantic processing
phonological processing
remembering
inference
meaning
attention
social cognition
decision making
comprehension
mental rotation
consciousness
metaphor
sentence comprehension
memory encoding
short term memory
choice
emotion
recall
humor
search
impulsivity
visual search
mental imagery
reward
emotion regulation
language processing
language
reading
selective attention
spatial attention
eating
risk
object recognition
arousal
empathy
face perception
loss
sustained attention
spatial working memory
fear
consolidation
rehearsal
naming
stress
action
syntax
anticipation
learning
gaze
communication
induction
sleep
listening
visual attention
extinction
prosody
integration
associative memory
anxiety
skill
motor imagery
emotional expression
speech perception
movement
habit
facial expression
speech production
retention
sequence learning
pain
melody
motor control
motor learning

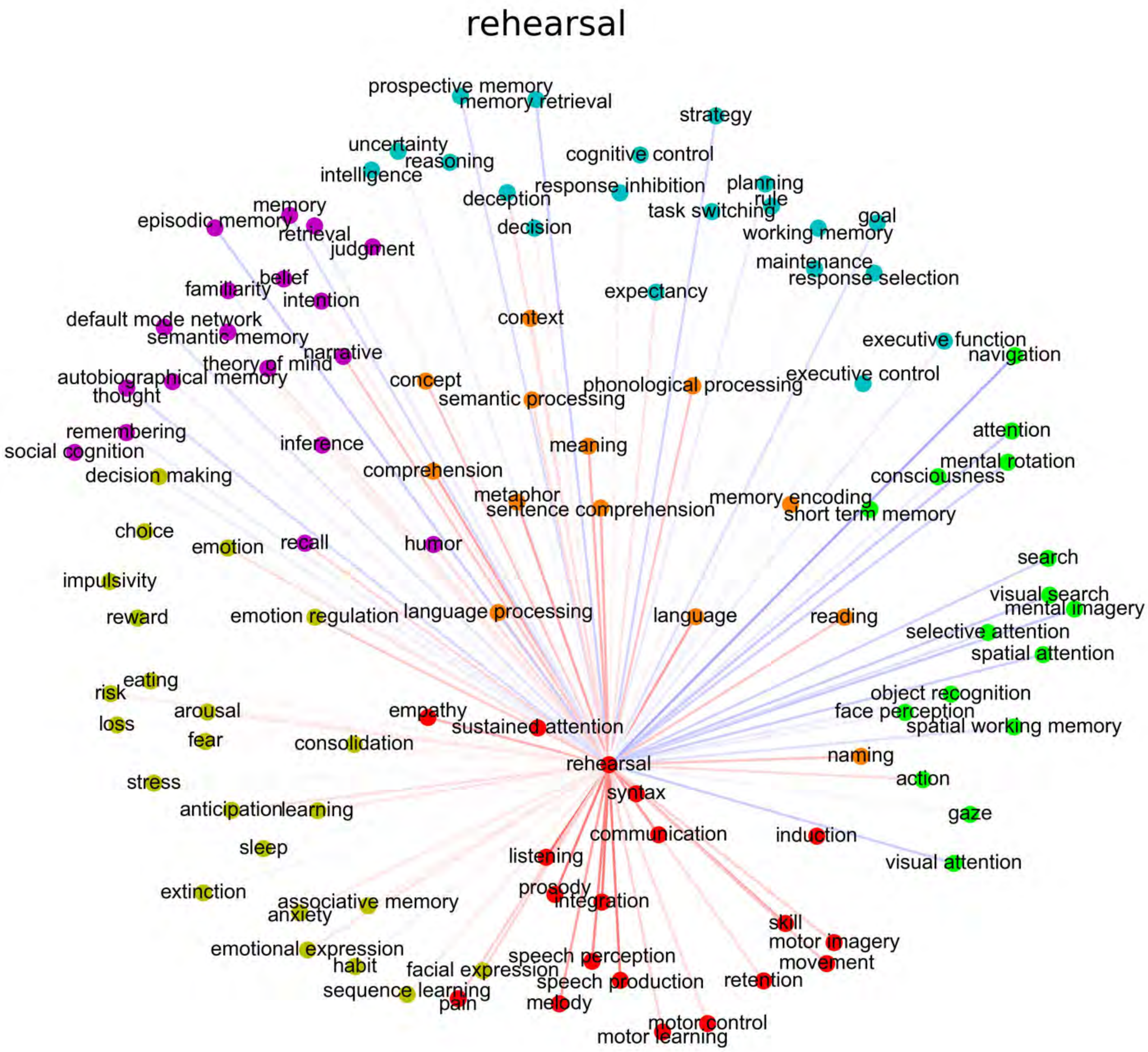
rehearsal
prospective memory
memory retrieval
strategy
uncertainty
reasoning
intelligence
cognitive control
response inhibition
planning
rule
deception
task switching
goal
working memory
decision
memory
episodic memory
retrieval
judgment
maintenance
response selection
belief
familiarity
intention
expectancy
context
default mode network
semantic memory
executive function
navigation
narrative
theory of mind
concept
autobiographical memory
thought
phonological processing
executive control
semantic processing
remembering
social cognition
inference
meaning
attention
mental rotation
decision making
comprehension
consciousness
metaphor
sentence comprehension
memory encoding
short term memory
choice
emotion
recall
humor
search
impulsivity
visual search
mental imagery
reward
emotion regulation
language processing
language
reading
selective attention
spatial attention
eating
risk
arousal
object recognition
empathy
face perception
loss
sustained attention
spatial working memory
fear
consolidation
naming
stress
syntax
action
anticipation
learning
gaze
sleep
communication
induction
listening
visual attention
extinction
prosody
integration
associative memory
anxiety
skill
motor imagery
emotional expression
habit
speech perception
movement
facial expression
speech production
retention
sequence learning
pain
melody
motor control
motor learning

remembering

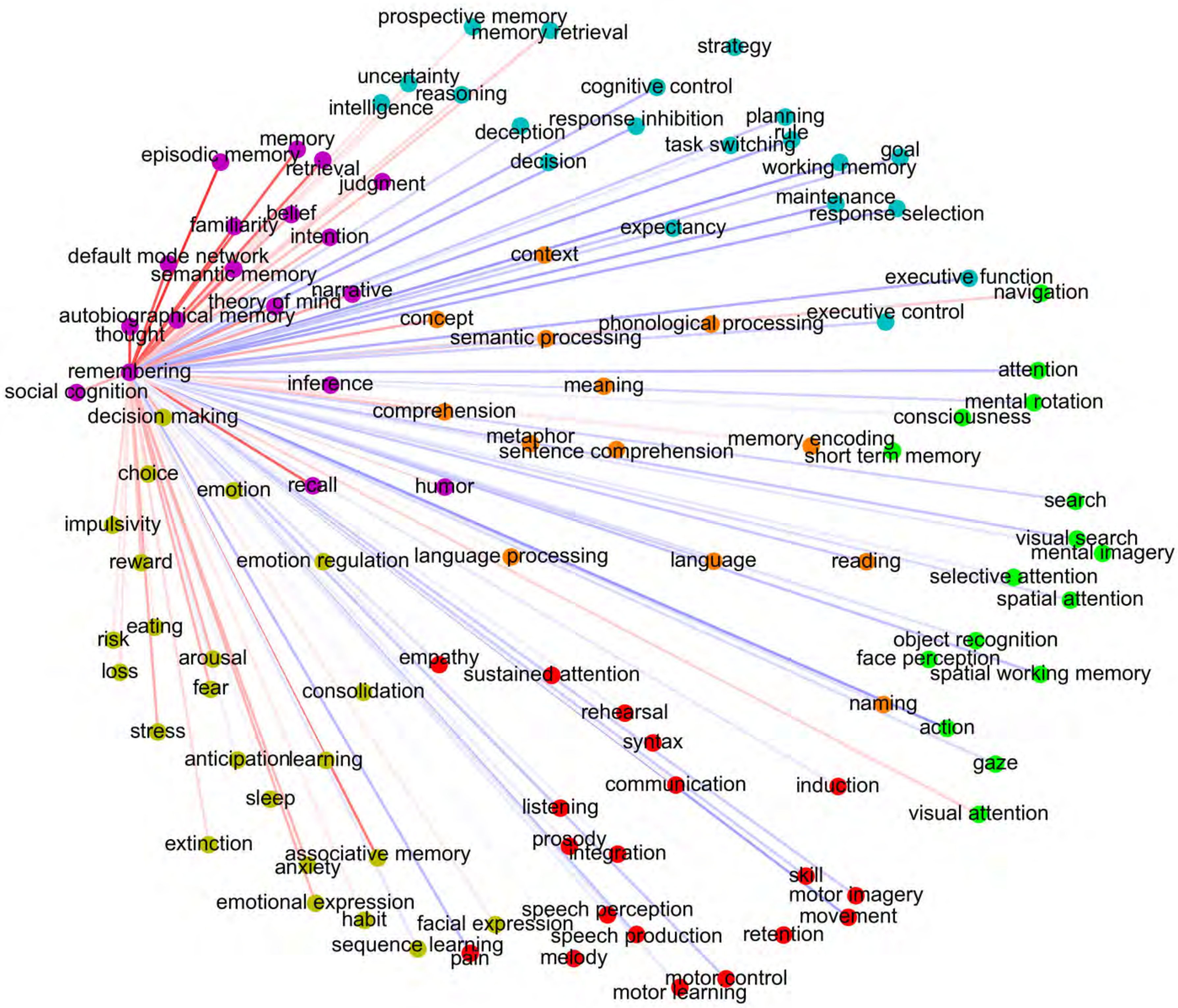

response inhibition

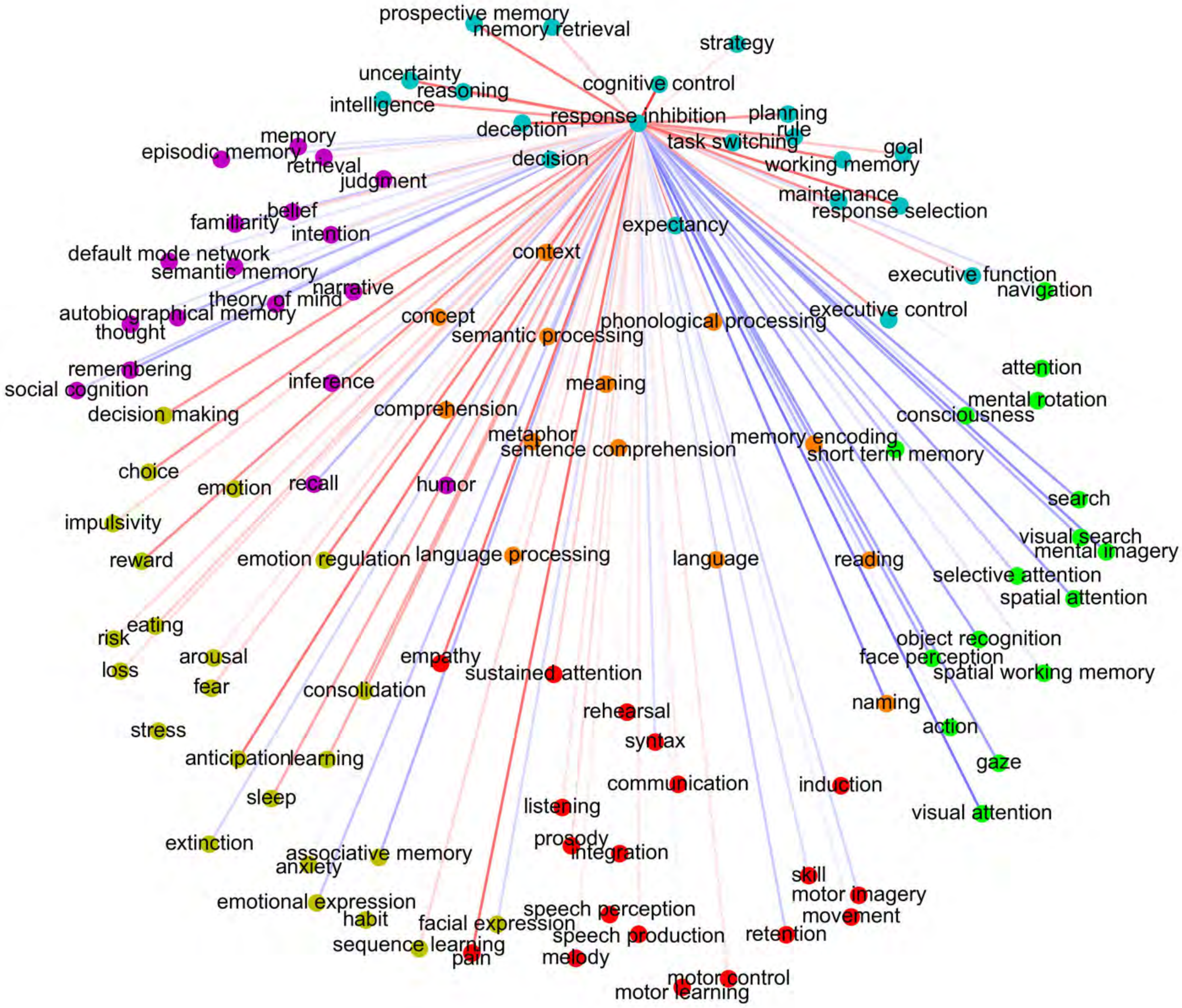

response selection

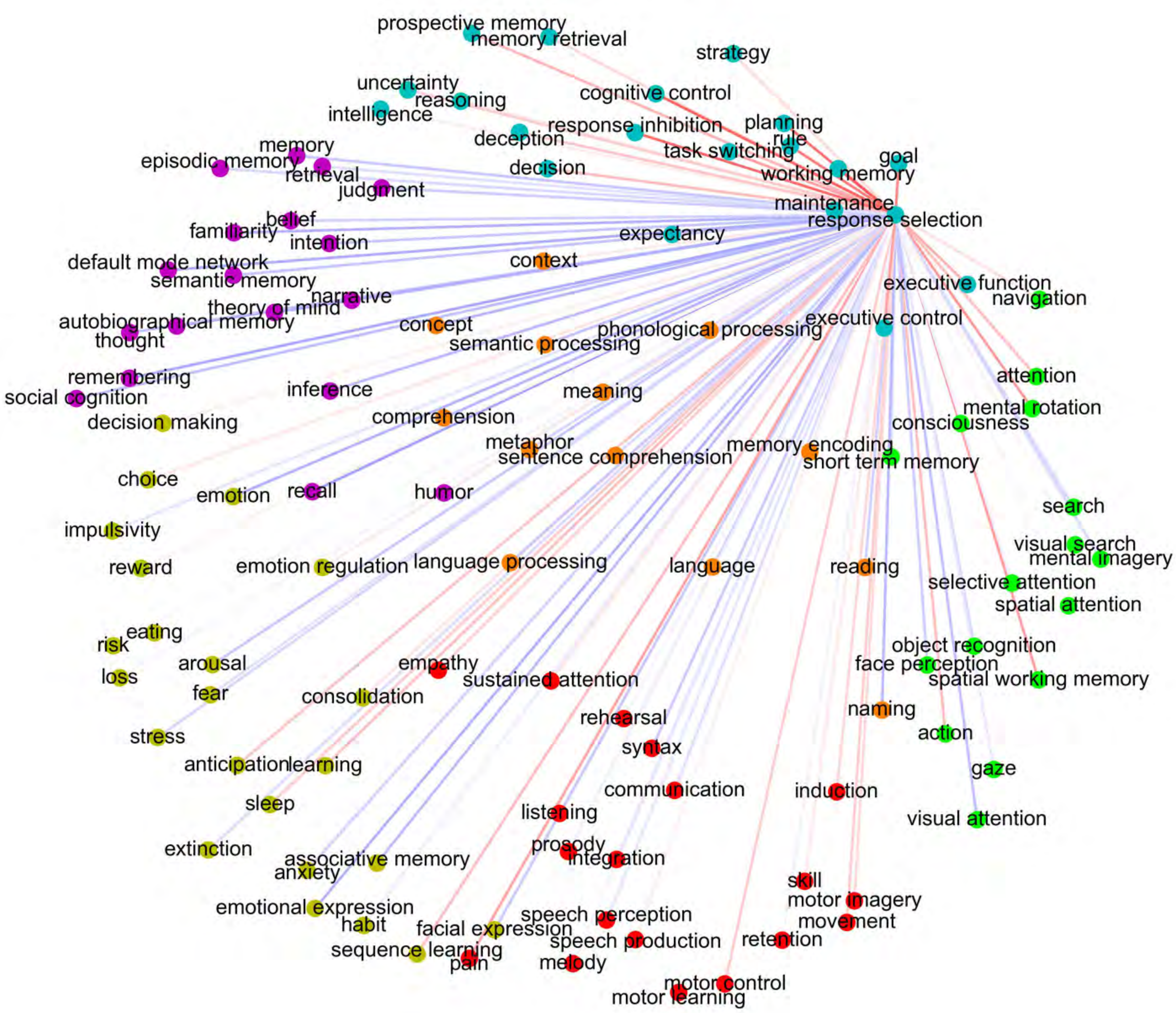

retention

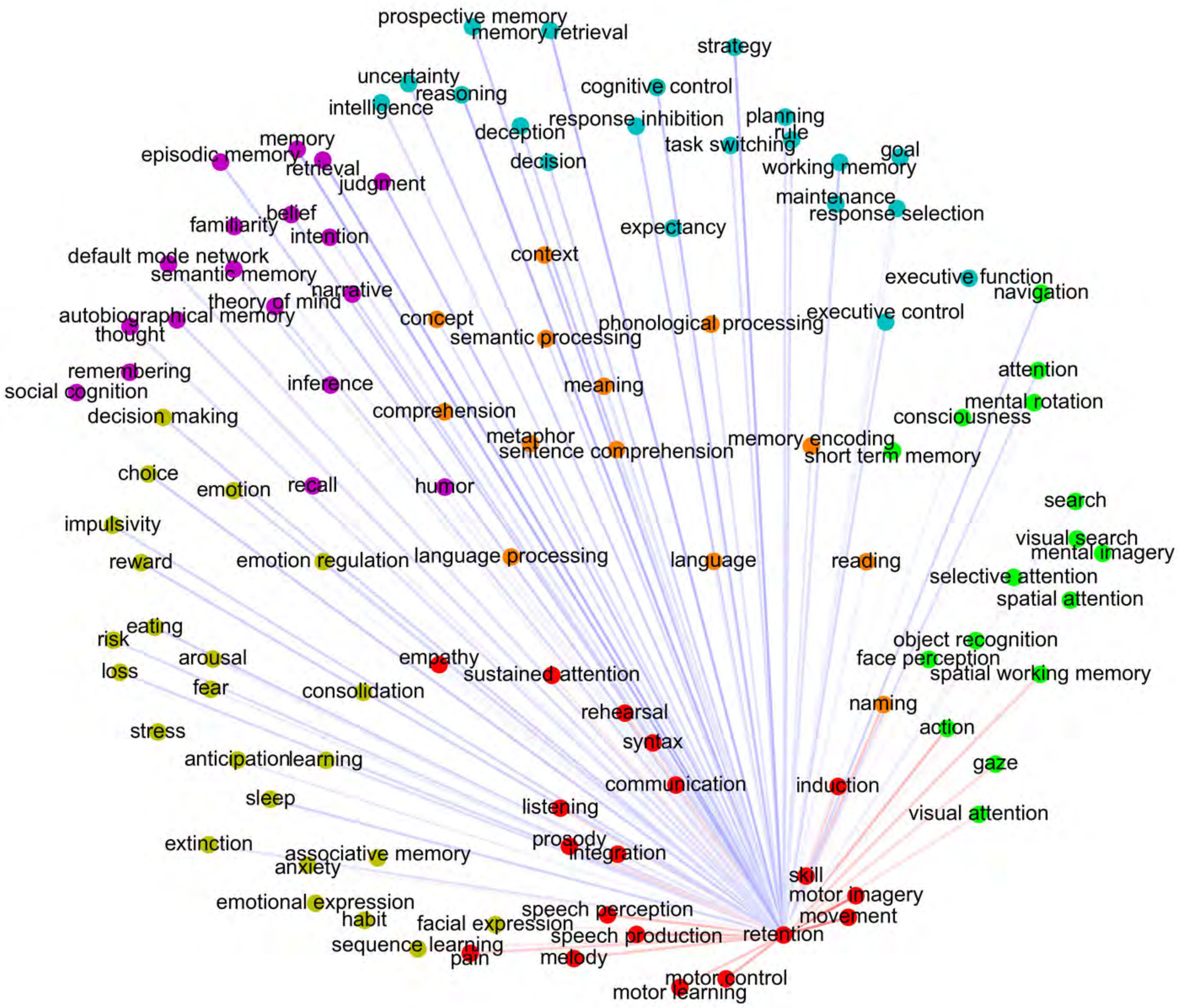

prospective memory
memory retrieval
strategy
uncertainty
reasoning
cognitive control
intelligence
response inhibition
planning
deception
rule
memory
task switching
episodic memory
goal
retrieval
decision
working memory
judgment
maintenance
belief
response selection
familiarity
intention
expectancy
context
default mode network
semantic memory
executive function
narrative
navigation
theory of mind
autobiographical memory
concept
phonological processing
executive control
thought
semantic processing
remembering
attention
inference
meaning
social cognition
mental rotation
decision making
comprehension
consciousness
metaphor
memory encoding
sentence comprehension
short term memory
choice
emotion
recall
humor
search
impulsivity
visual search
mental imagery
reward
emotion regulation
language processing
language
reading
selective attention
spatial attention
eating
risk
object recognition
arousal
empathy
face perception
loss
sustained attention
spatial working memory
fear
consolidation
rehearsal
naming
stress
syntax
action
anticipation
learning
gaze
communication
induction
sleep
listening
visual attention
extinction
prosody
integration
associative memory
anxiety
skill
motor imagery
emotional expression
speech perception
habit
movement
facial expression
speech production
retention
sequence learning
pain
melody
motor control
motor learning

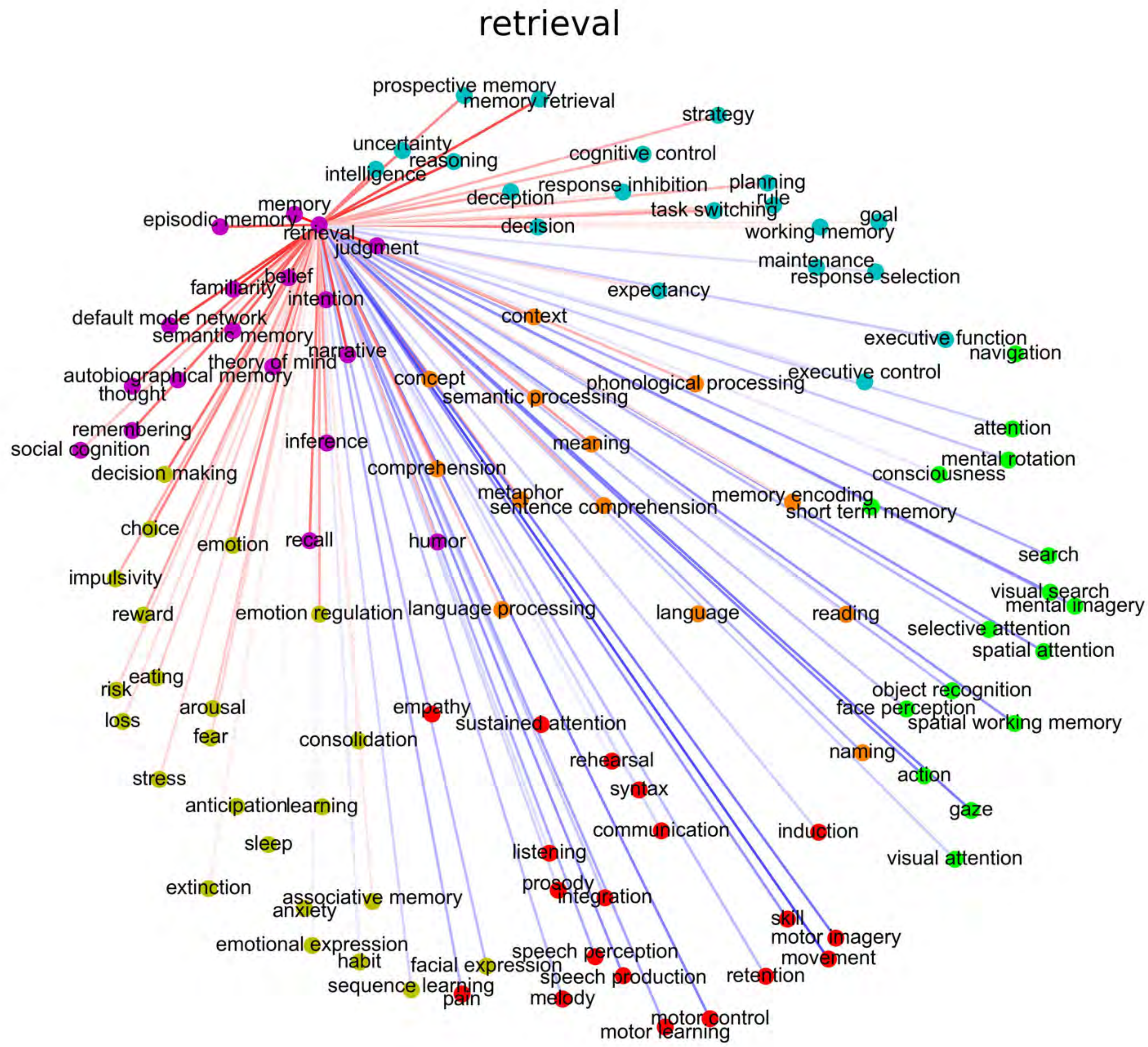
retrieval
prospective memory
memory retrieval
strategy
uncertainty
reasoning
cognitive control
intelligence
planning
response inhibition
rule
deception
task switching
goal
memory
episodic memory
retrieval
decision
working memory
judgment
maintenance
response selection
belief
familiarity
intention
expectancy
default mode network
context
semantic memory
executive function
navigation
narrative
theory of mind
autobiographical memory
concept
executive control
phonological processing
thought
semantic processing
remembering
attention
social cognition
inference
meaning
mental rotation
decision making
comprehension
consciousness
metaphor
memory encoding
sentence comprehension
short term memory
choice
emotion
recall
humor
search
impulsivity
visual search
mental imagery
reward
emotion regulation
language processing
language
reading
selective attention
spatial attention
eating
risk
object recognition
arousal
empathy
face perception
loss
sustained attention
spatial working memory
fear
consolidation
naming
rehearsal
action
stress
syntax
gaze
anticipation
learning
communication
induction
sleep
listening
visual attention
extinction
prosody
integration
associative memory
anxiety
skill
motor imagery
emotional expression
speech perception
movement
habit
facial expression
speech production
retention
sequence learning
pain
melody
motor control
motor learning

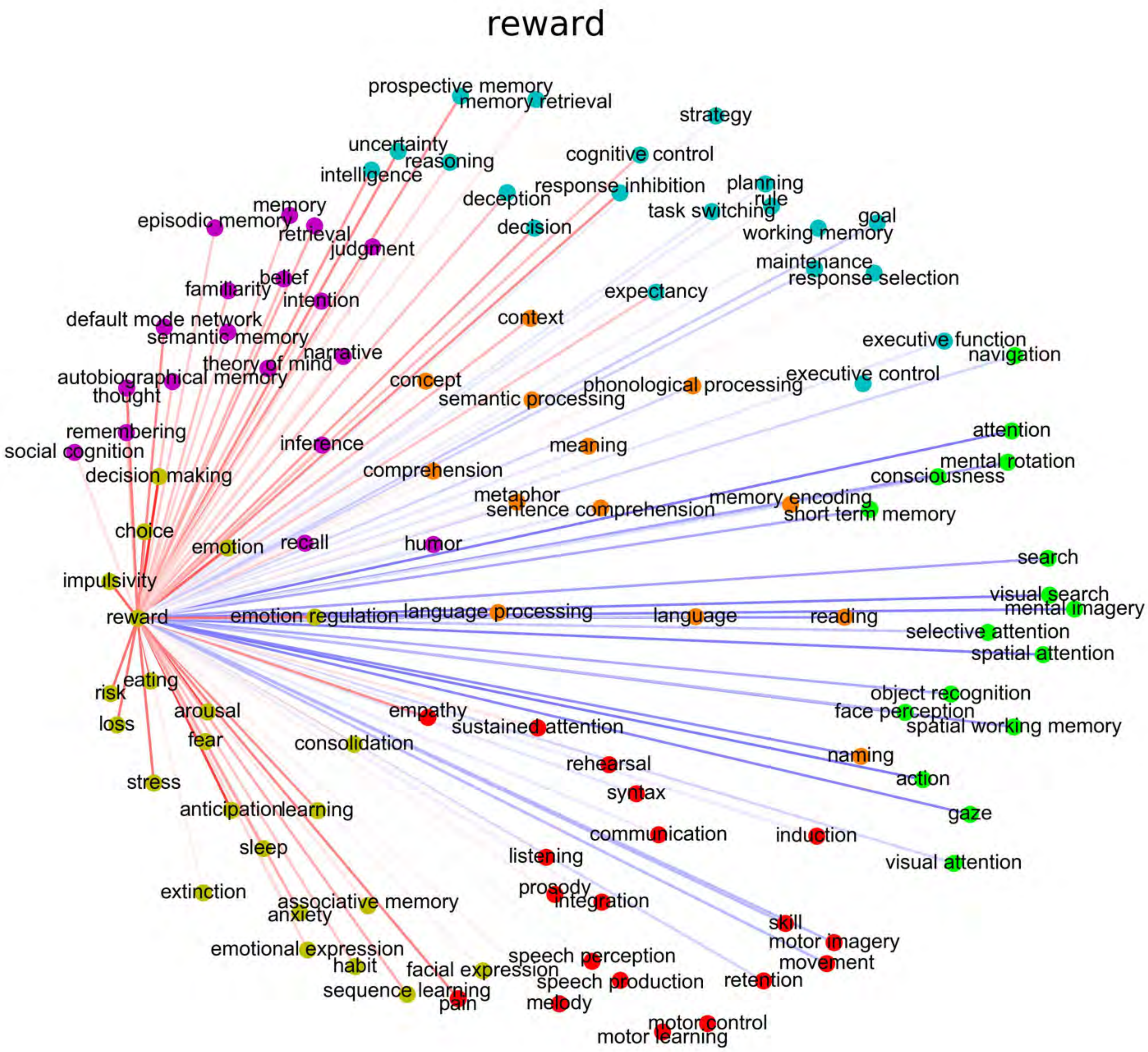
reward
prospective memory
memory retrieval
strategy
uncertainty
reasoning
cognitive control
intelligence
response inhibition
planning
rule
deception
task switching
goal
memory
episodic memory
retrieval
decision
working memory
judgment
maintenance
response selection
belief
familiarity
intention
expectancy
default mode network
context
semantic memory
executive function
narrative
navigation
theory of mind
executive control
autobiographical memory
concept
phonological processing
thought
semantic processing
remembering
attention
social cognition
inference
meaning
decision making
comprehension
mental rotation
consciousness
metaphor
memory encoding
sentence comprehension
short term memory
choice
emotion
recall
humor
search
impulsivity
visual search
mental imagery
reward
emotion regulation
language processing
language
reading
selective attention
spatial attention
eating
risk
object recognition
arousal
empathy
face perception
loss
sustained attention
spatial working memory
fear
consolidation
rehearsal
naming
stress
action
syntax
anticipation
learning
gaze
communication
induction
sleep
listening
visual attention
extinction
prosody
integration
associative memory
anxiety
skill
motor imagery
emotional expression
movement
speech perception
habit
facial expression
speech production
retention
sequence learning
pain
melody
motor control
motor learning

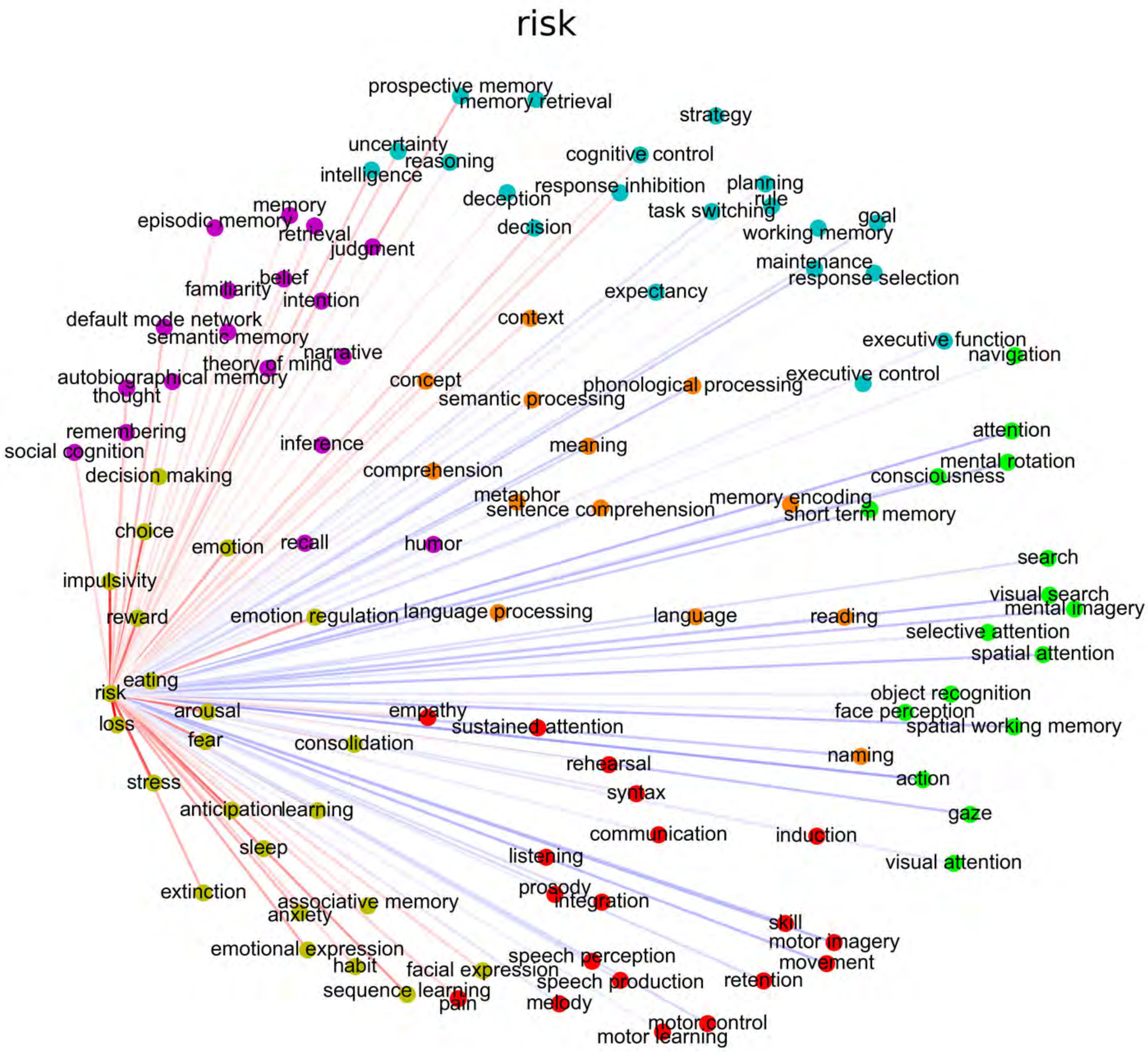
risk
prospective memory
memory retrieval
strategy
uncertainty
reasoning
cognitive control
intelligence
deception
response inhibition
planning
rule
memory
episodic memory
retrieval
task switching
goal
decision
working memory
judgment
maintenance
response selection
belief
familiarity
intention
expectancy
default mode network
semantic memory
context
executive function
navigation
narrative
theory of mind
autobiographical memory
concept
phonological processing
executive control
thought
semantic processing
remembering
social cognition
inference
meaning
attention
mental rotation
decision making
comprehension
consciousness
metaphor
memory encoding
sentence comprehension
short term memory
choice
emotion
recall
humor
search
impulsivity
visual search
mental imagery
reward
emotion regulation
language processing
language
reading
selective attention
spatial attention
eating
object recognition
face perception
empathy
arousal
sustained attention
spatial working memory
loss
fear
consolidation
naming
rehearsal
action
stress
syntax
gaze
anticipation
learning
communication
induction
sleep
listening
visual attention
extinction
prosody
integration
associative memory
anxiety
skill
motor imagery
emotional expression
movement
speech perception
habit
facial expression
speech production
retention
sequence learning
pain
melody
motor control
motor learning

rule

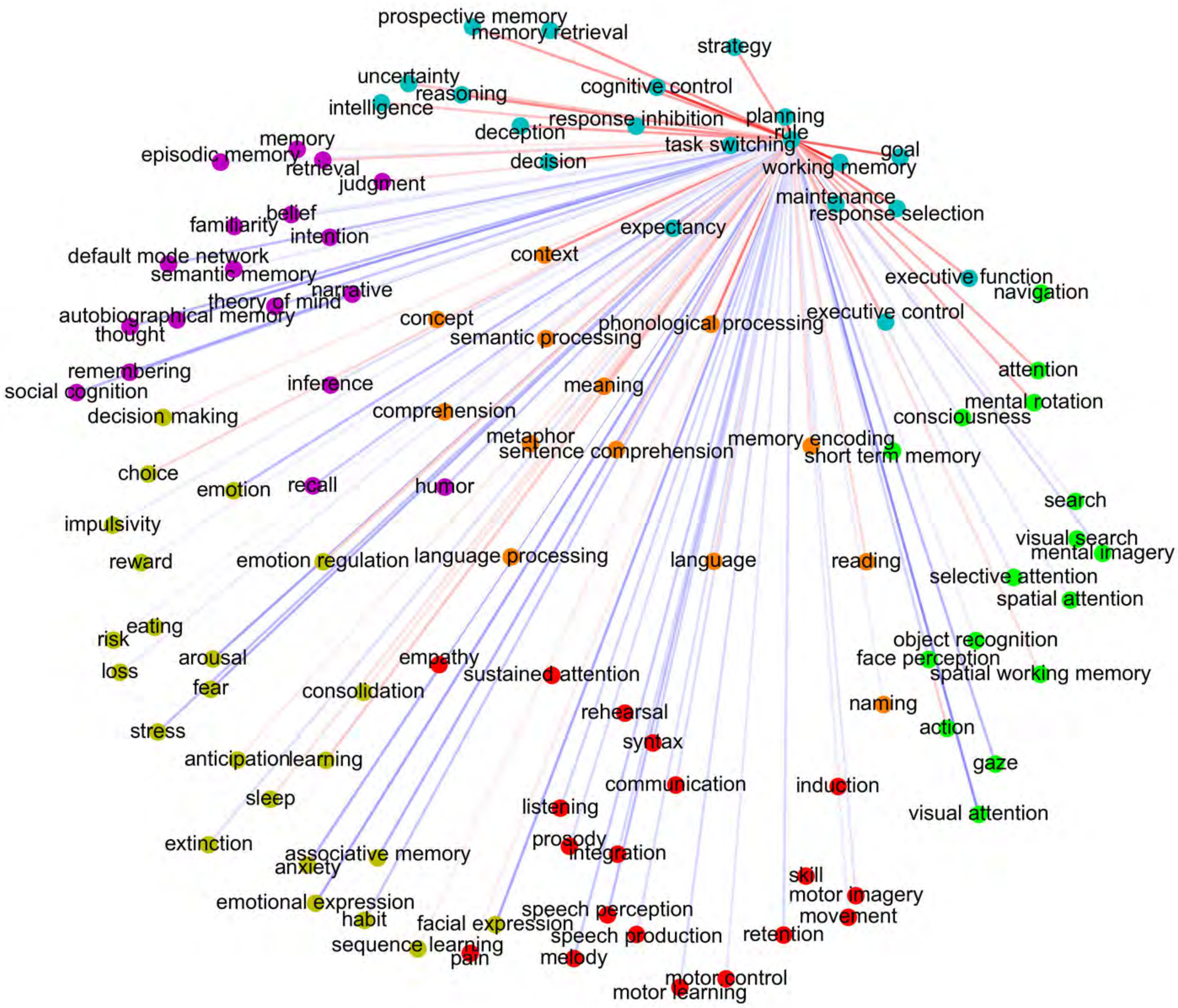

prospective memory
memory retrieval
strategy
uncertainty
reasoning
cognitive control
intelligence
planning
response inhibition
rule
deception
memory
task switching
goal
episodic memory
retrieval
decision
working memory
judgment
maintenance
response selection
belief
familiarity
intention
expectancy
default mode network
context
semantic memory
executive function
navigation
narrative
theory of mind
executive control
concept
phonological processing
autobiographical memory
thought
semantic processing
remembering
attention
social cognition
inference
meaning
mental rotation
decision making
comprehension
consciousness
metaphor
memory encoding
sentence comprehension
short term memory
choice
emotion
recall
humor
search
impulsivity
visual search
mental imagery
reward
emotion regulation
language processing
language
reading
selective attention
spatial attention
eating
risk
object recognition
arousal
empathy
face perception
loss
sustained attention
spatial working memory
fear
consolidation
naming
rehearsal
action
stress
syntax
anticipation
learning
gaze
communication
induction
sleep
listening
visual attention
extinction
prosody
integration
associative memory
anxiety
skill
motor imagery
emotional expression
speech perception
habit
movement
facial expression
speech production
retention
sequence learning
pain
melody
motor control
motor learning

search

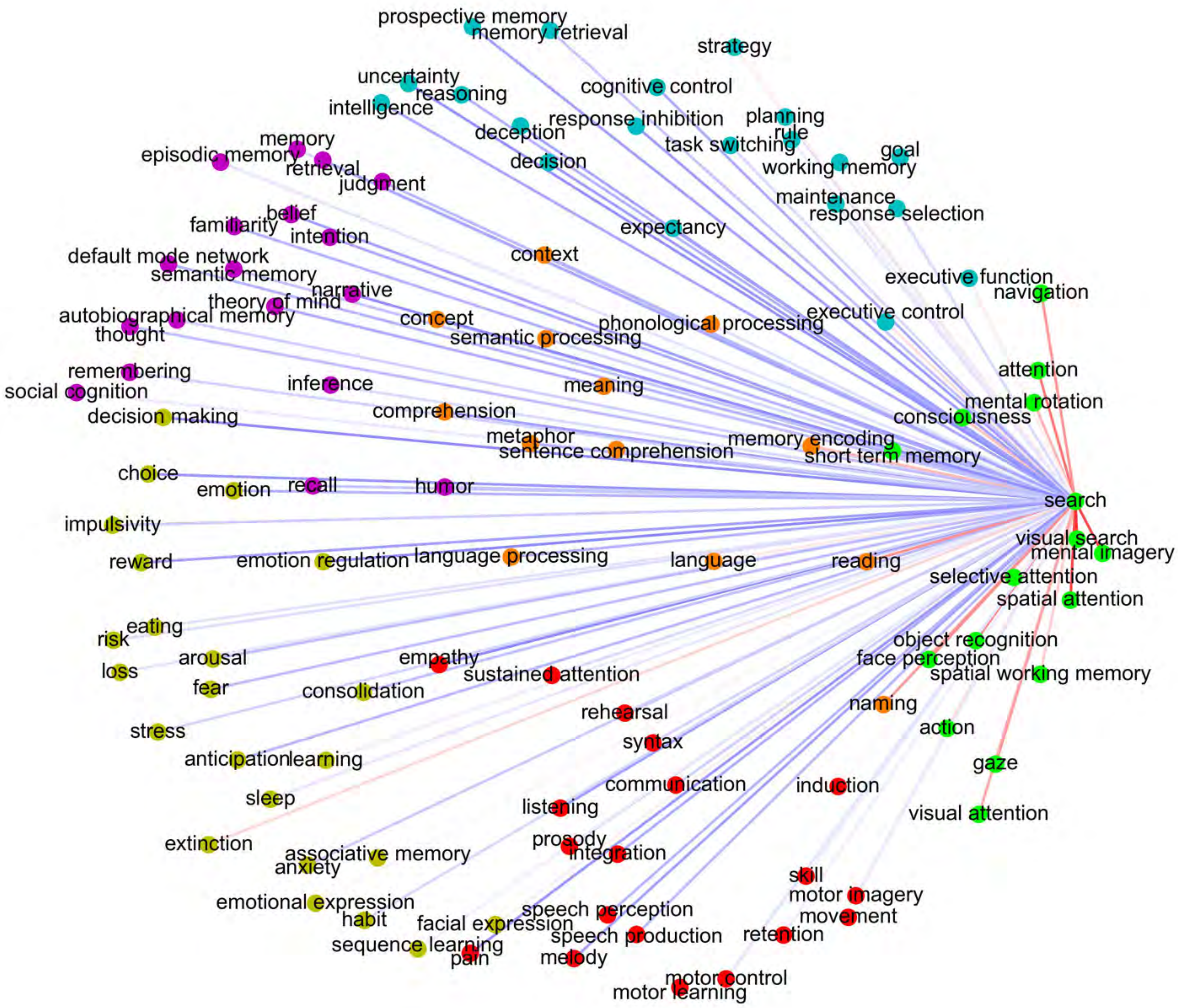
prospective memory
memory retrieval
strategy
uncertainty
reasoning
cognitive control
intelligence
planning
response inhibition
rule
deception
task switching
goal
memory
episodic memory
retrieval
decision
working memory
judgment
maintenance
belief
response selection
familiarity
intention
expectancy
default mode network
context
semantic memory
executive function
navigation
narrative
theory of mind
executive control
autobiographical memory
concept
phonological processing
thought
semantic processing
remembering
attention
inference
meaning
social cognition
mental rotation
decision making
comprehension
consciousness
metaphor
memory encoding
sentence comprehension
short term memory
choice
recall
humor
emotion
search
impulsivity
visual search
mental imagery
reward
emotion regulation
language processing
language
reading
selective attention
spatial attention
eating
risk
object recognition
arousal
empathy
face perception
loss
sustained attention
spatial working memory
fear
consolidation
rehearsal
naming
stress
action
syntax
anticipation
learning
gaze
communication
induction
sleep
listening
visual attention
extinction
prosody
integration
associative memory
anxiety
skill
motor imagery
emotional expression
speech perception
movement
habit
facial expression
speech production
retention
sequence learning
pain
melody
motor control
motor learning

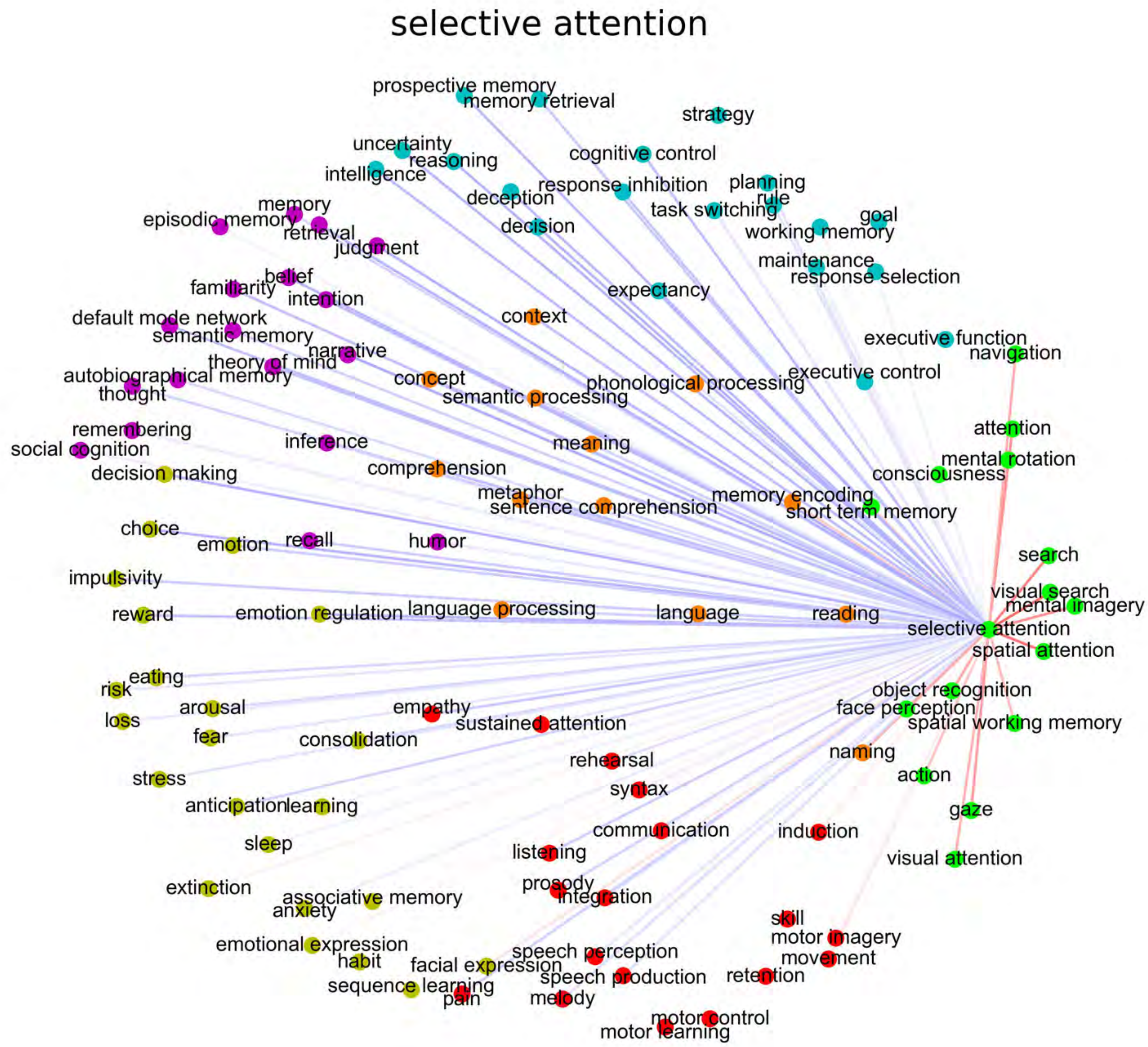
selective attention
prospective memory
memory retrieval
strategy
uncertainty
reasoning
cognitive control
intelligence
planning
response inhibition
rule
deception
task switching
goal
memory
episodic memory
retrieval
decision
working memory
judgment
maintenance
response selection
belief
familiarity
intention
expectancy
default mode network
context
semantic memory
executive function
navigation
narrative
theory of mind
executive control
autobiographical memory
concept
phonological processing
thought
semantic processing
attention
remembering
meaning
inference
social cognition
mental rotation
consciousness
decision making
comprehension
metaphor
memory encoding
sentence comprehension
short term memory
choice
emotion
recall
humor
search
impulsivity
visual search
mental imagery
reward
emotion regulation
language processing
language
reading
selective attention
spatial attention
eating
risk
arousal
object recognition
empathy
face perception
loss
sustained attention
spatial working memory
fear
consolidation
naming
rehearsal
stress
action
syntax
anticipation
learning
gaze
communication
induction
sleep
listening
visual attention
extinction
prosody
integration
associative memory
anxiety
skill
motor imagery
emotional expression
speech perception
movement
habit
facial expression
speech production
retention
sequence learning
pain
melody
motor control
motor learning

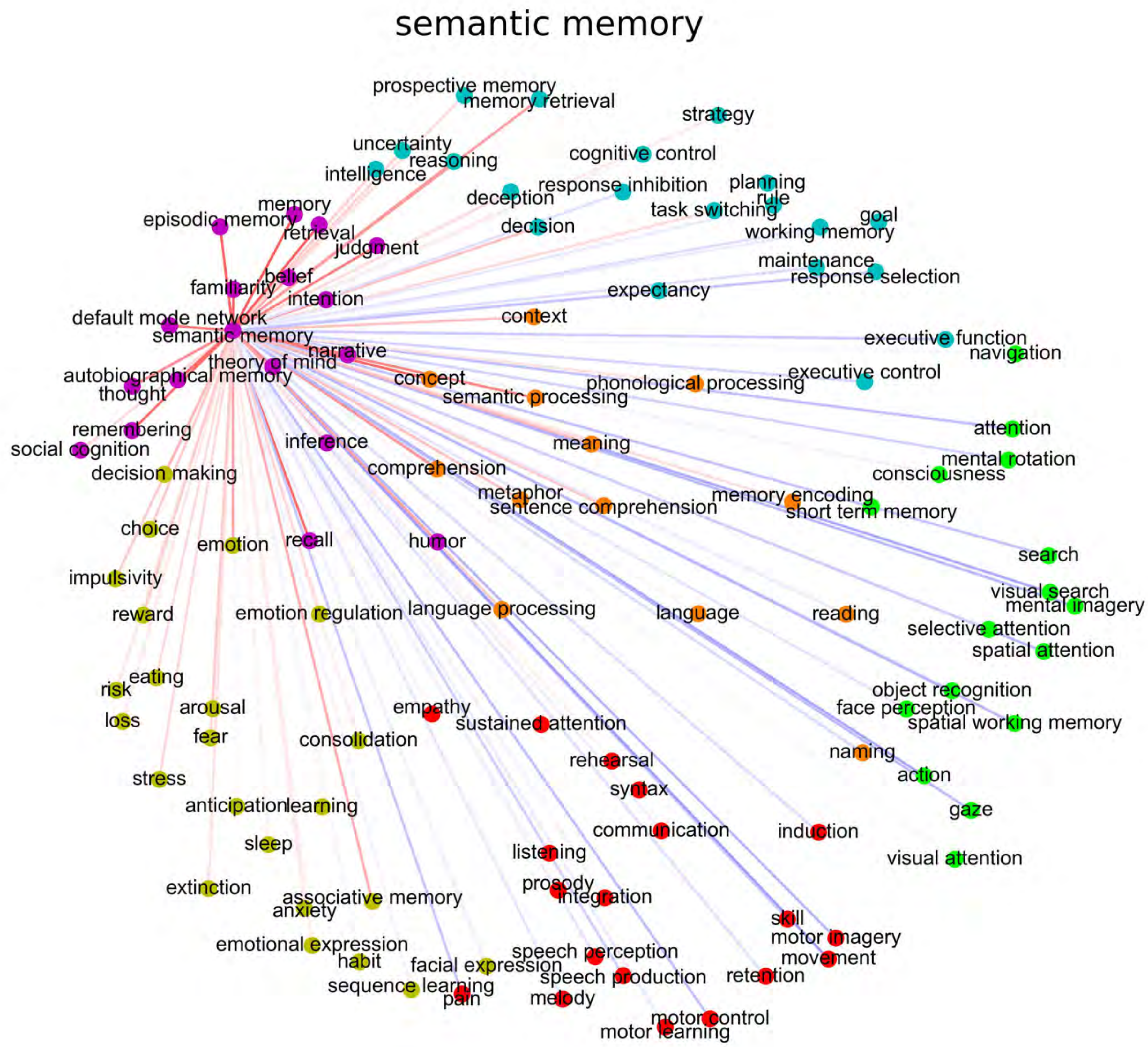
semantic memory
prospective memory
memory retrieval
strategy
uncertainty
reasoning
cognitive control
intelligence
planning
response inhibition
rule
deception
task switching
memory
goal
episodic memory
retrieval
decision
working memory
judgment
maintenance
response selection
belief
familiarity
intention
expectancy
default mode network
context
semantic memory
executive function
narrative
navigation
theory of mind
executive control
autobiographical memory
concept
phonological processing
thought
semantic processing
attention
remembering
meaning
social cognition
inference
mental rotation
decision making
comprehension
consciousness
metaphor
memory encoding
sentence comprehension
short term memory
choice
emotion
recall
humor
search
impulsivity
visual search
mental imagery
reward
emotion regulation
language processing
language
reading
selective attention
spatial attention
eating
risk
object recognition
arousal
face perception
loss
empathy
sustained attention
spatial working memory
fear
consolidation
rehearsal
naming
action
stress
syntax
anticipation
learning
gaze
communication
induction
sleep
listening
visual attention
extinction
prosody
integration
associative memory
anxiety
skill
motor imagery
emotional expression
speech perception
movement
habit
facial expression
speech production
retention
sequence learning
pain
melody
motor control
motor learning

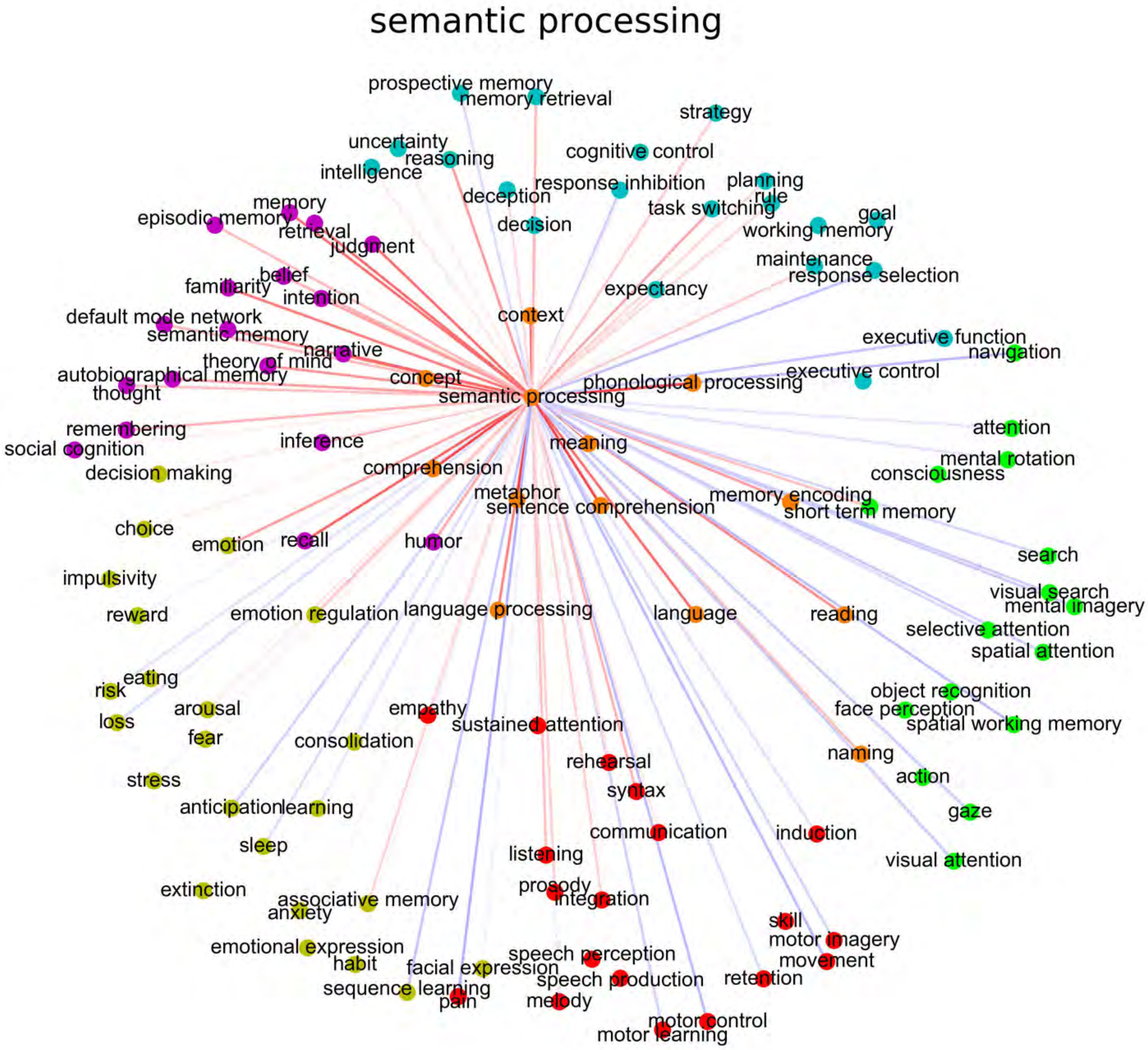
semantic processing
prospective memory
memory retrieval
strategy
uncertainty
reasoning
cognitive control
intelligence
response inhibition
planning
deception
rule
memory
task switching
goal
episodic memory
retrieval
decision
working memory
judgment
maintenance
belief
response selection
familiarity
intention
expectancy
context
default mode network
semantic memory
executive function
narrative
navigation
theory of mind
executive control
autobiographical memory
concept
phonological processing
thought
semantic processing
remembering
attention
social cognition
inference
meaning
mental rotation
decision making
comprehension
consciousness
metaphor
memory encoding
sentence comprehension
short term memory
choice
emotion
recall
humor
search
impulsivity
visual search
mental imagery
reward
emotion regulation
language processing
language
reading
selective attention
spatial attention
eating
risk
object recognition
arousal
empathy
face perception
loss
sustained attention
spatial working memory
fear
consolidation
rehearsal
naming
stress
syntax
action
anticipation
learning
gaze
communication
induction
sleep
listening
visual attention
extinction
prosody
integration
associative memory
anxiety
skill
motor imagery
emotional expression
speech perception
habit
movement
facial expression
speech production
retention
sequence learning
pain
melody
motor control
motor learning

sentence comprehension

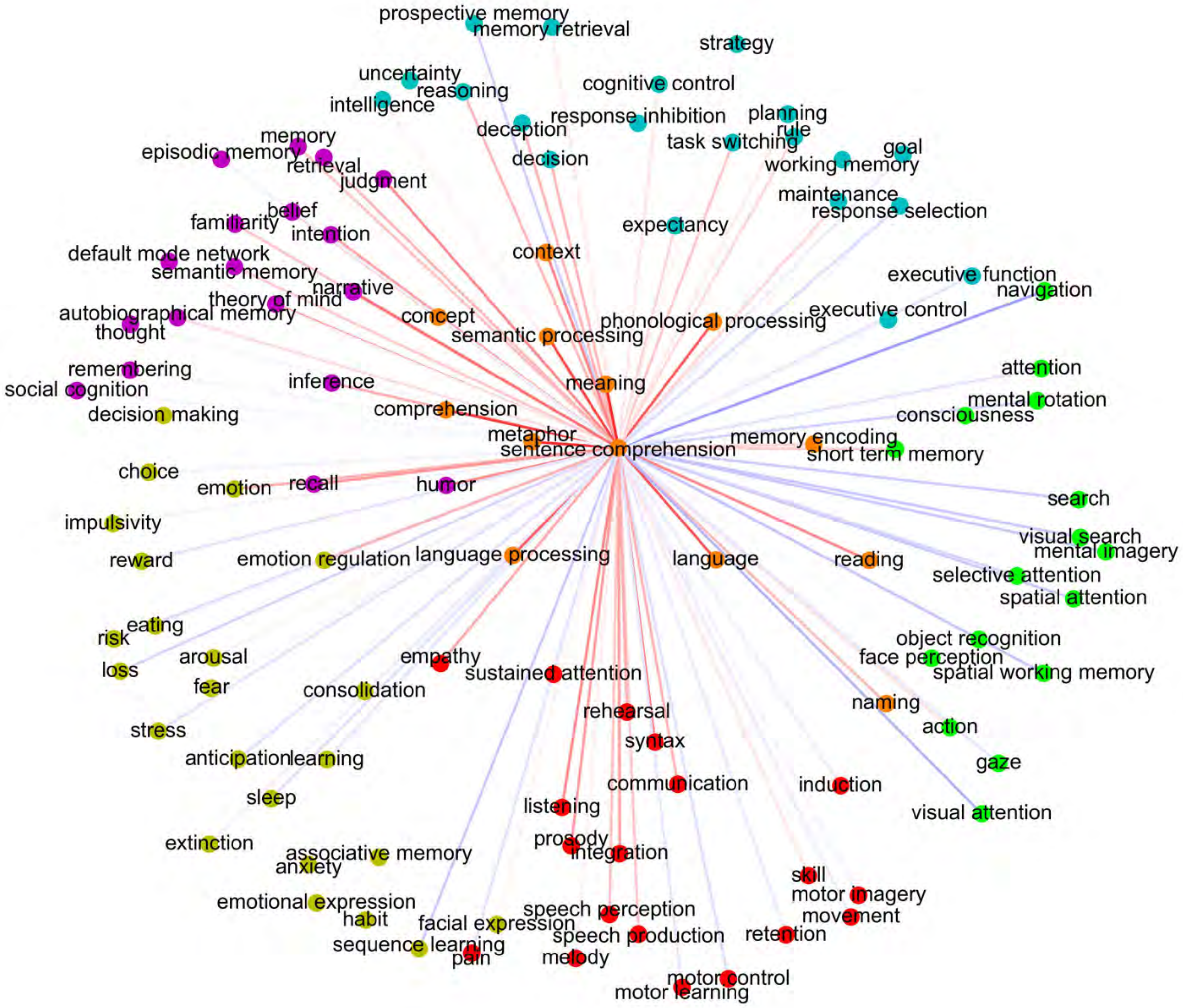

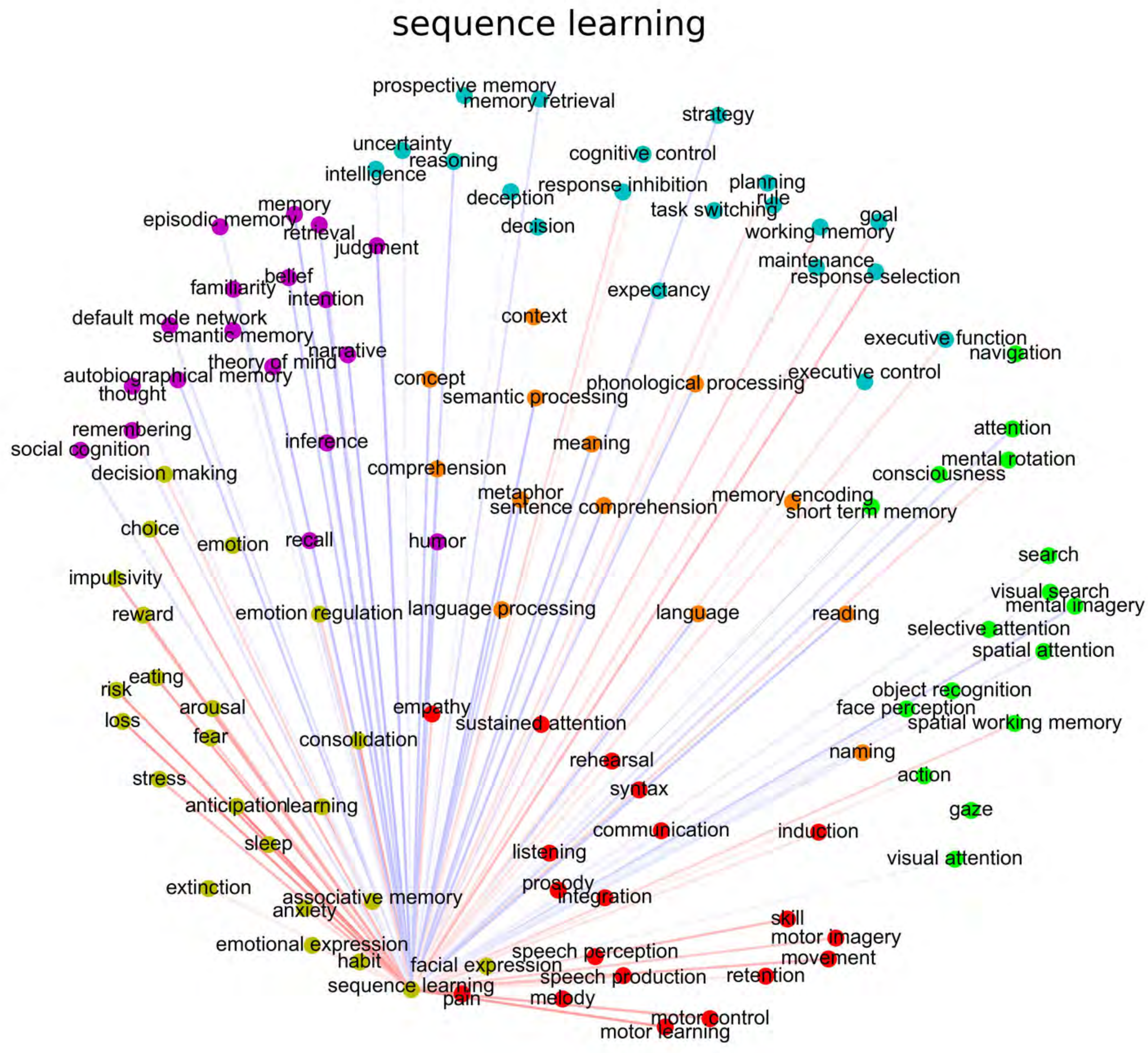
sequence learning
prospective memory
memory retrieval
strategy
uncertainty
reasoning
cognitive control
intelligence
planning
response inhibition
rule
deception
task switching
goal
memory
episodic memory
retrieval
decision
working memory
judgment
maintenance
response selection
belief
familiarity
intention
expectancy
context
default mode network
semantic memory
executive function
navigation
narrative
theory of mind
executive control
concept
phonological processing
autobiographical memory
thought
semantic processing
remembering
attention
inference
meaning
social cognition
mental rotation
decision making
comprehension
consciousness
metaphor
memory encoding
sentence comprehension
short term memory
choice
emotion
recall
humor
search
impulsivity
visual search
mental imagery
reward
emotion regulation
language processing
language
reading
selective attention
spatial attention
eating
risk
object recognition
arousal
empathy
face perception
loss
sustained attention
spatial working memory
fear
consolidation
naming
rehearsal
stress
action
syntax
anticipation
learning
gaze
communication
induction
sleep
listening
visual attention
extinction
prosody
integration
associative memory
anxiety
skill
motor imagery
emotional expression
speech perception
habit
movement
facial expression
speech production
retention
sequence learning
pain
melody
motor control
motor learning

short term memory

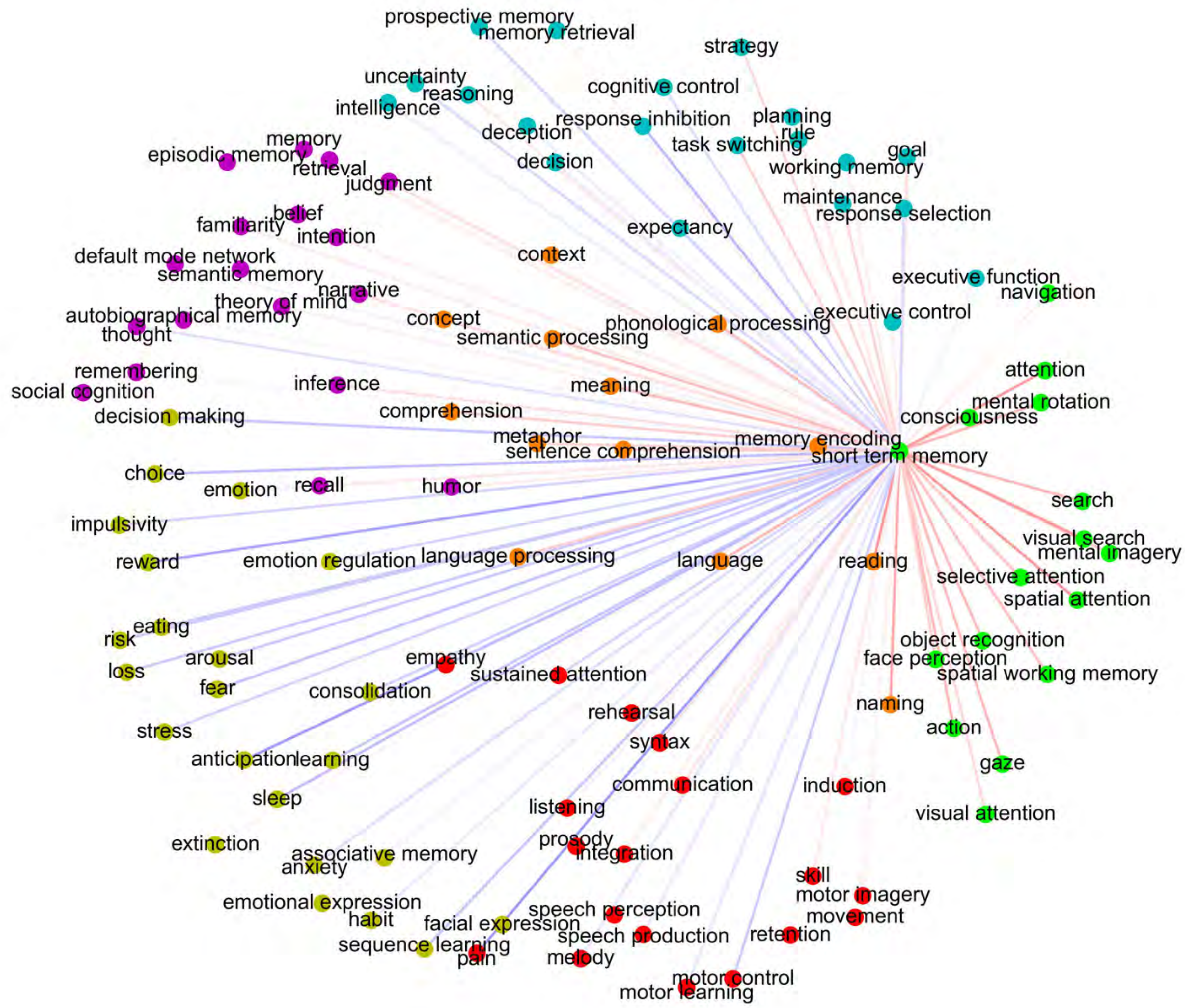

skill

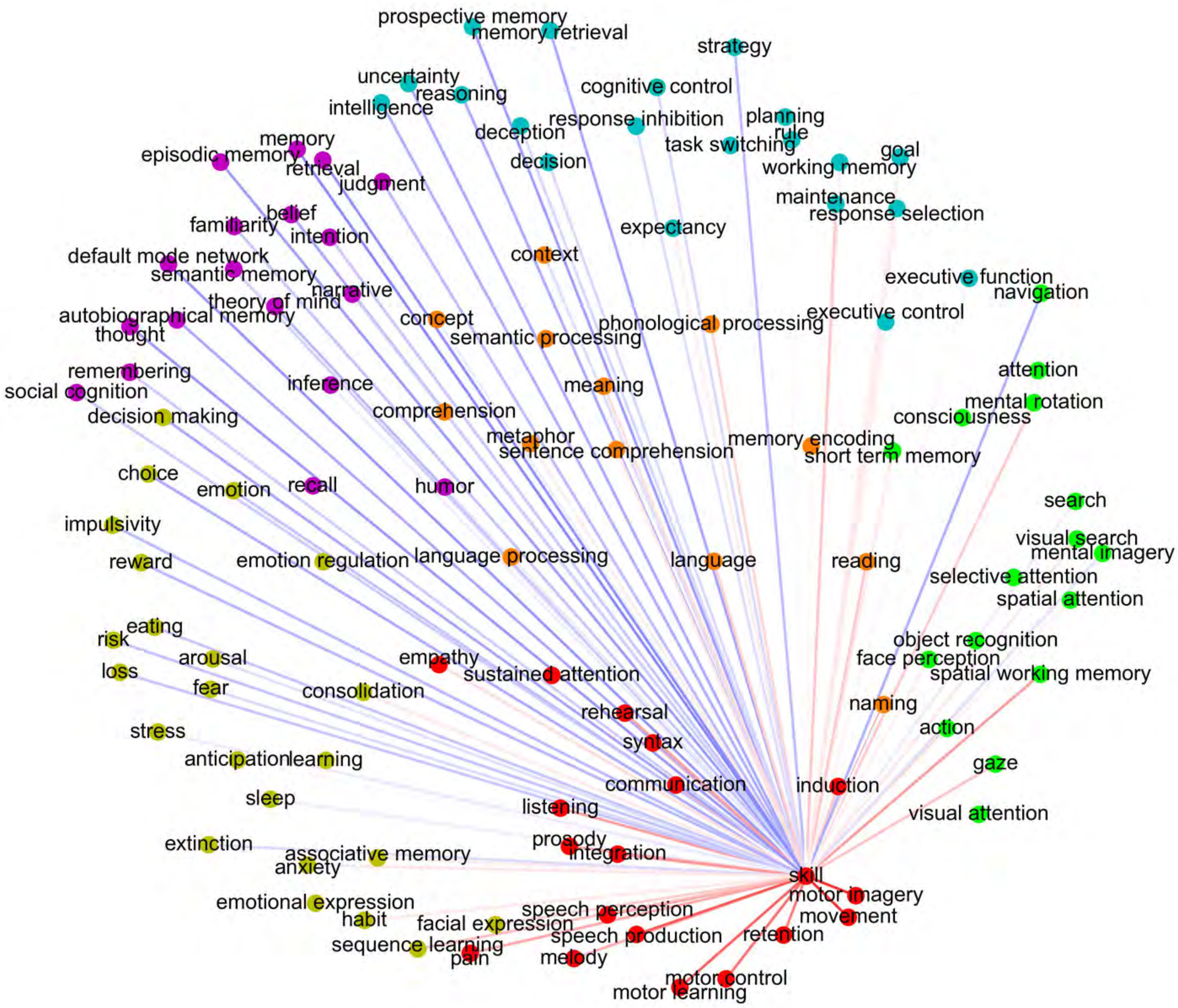
prospective memory
memory retrieval
strategy
uncertainty
reasoning
cognitive control
intelligence
response inhibition
planning
rule
deception
task switching
memory
goal
episodic memory
decision
working memory
retrieval
judgment
maintenance
response selection
belief
familiarity
intention
expectancy
context
default mode network
semantic memory
executive function
navigation
narrative
theory of mind
executive control
autobiographical memory
concept
phonological processing
thought
semantic processing
remembering
attention
meaning
inference
social cognition
mental rotation
comprehension
decision making
consciousness
metaphor
memory encoding
sentence comprehension
short term memory
choice
emotion
recall
humor
search
impulsivity
visual search
mental imagery
language processing
language
reading
reward
emotion regulation
selective attention
spatial attention
eating
risk
object recognition
arousal
empathy
face perception
loss
sustained attention
spatial working memory
fear
consolidation
rehearsal
naming
stress
action
syntax
anticipation
learning
gaze
communication
induction
sleep
listening
visual attention
extinction
prosody
integration
associative memory
anxiety
skill
motor imagery
emotional expression
speech perception
movement
habit
facial expression
speech production
retention
sequence learning
pain
melody
motor control
motor learning

sleep

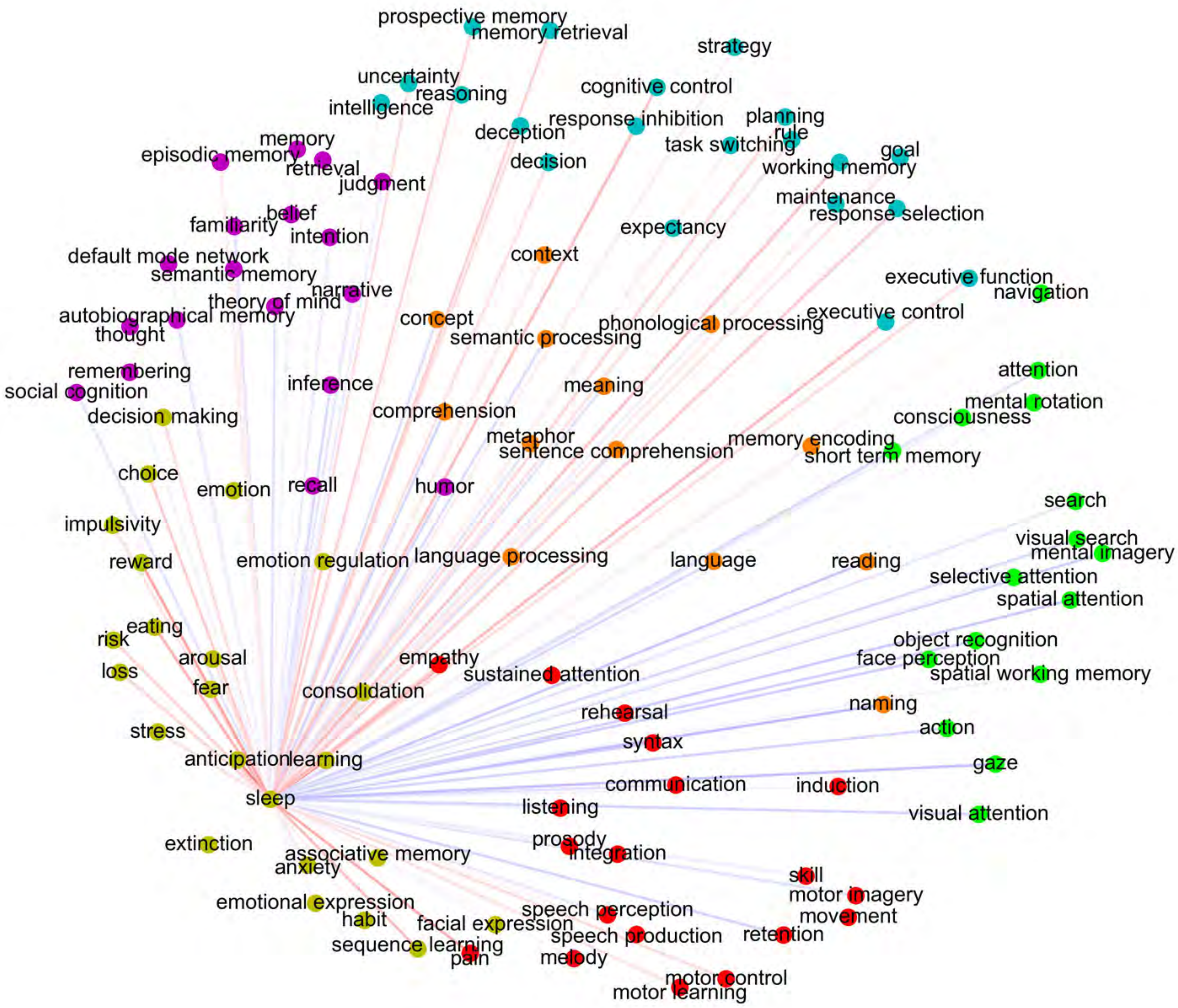
prospective memory
memory retrieval
strategy
uncertainty
reasoning
intelligence
cognitive control
response inhibition
planning
rule
deception
memory
episodic memory
retrieval
judgment
task switching
goal
working memory
decision
maintenance
response selection
belief
familiarity
intention
expectancy
context
default mode network
semantic memory
executive function
navigation
narrative
theory of mind
concept
autobiographical memory
thought
phonological processing
executive control
semantic processing
remembering
social cognition
inference
meaning
attention
mental rotation
decision making
comprehension
consciousness
metaphor
sentence comprehension
memory encoding
short term memory
choice
emotion
recall
humor
impulsivity
search
visual search
mental imagery
reward
emotion regulation
language processing
language
reading
selective attention
spatial attention
risk
eating
object recognition
arousal
empathy
face perception
loss
sustained attention
spatial working memory
fear
consolidation
naming
rehearsal
stress
action
syntax
anticipation
learning
gaze
communication
induction
sleep
listening
visual attention
extinction
prosody
integration
associative memory
anxiety
skill
motor imagery
emotional expression
speech perception
movement
habit
facial expression
speech production
retention
sequence learning
pain
melody
motor control
motor learning

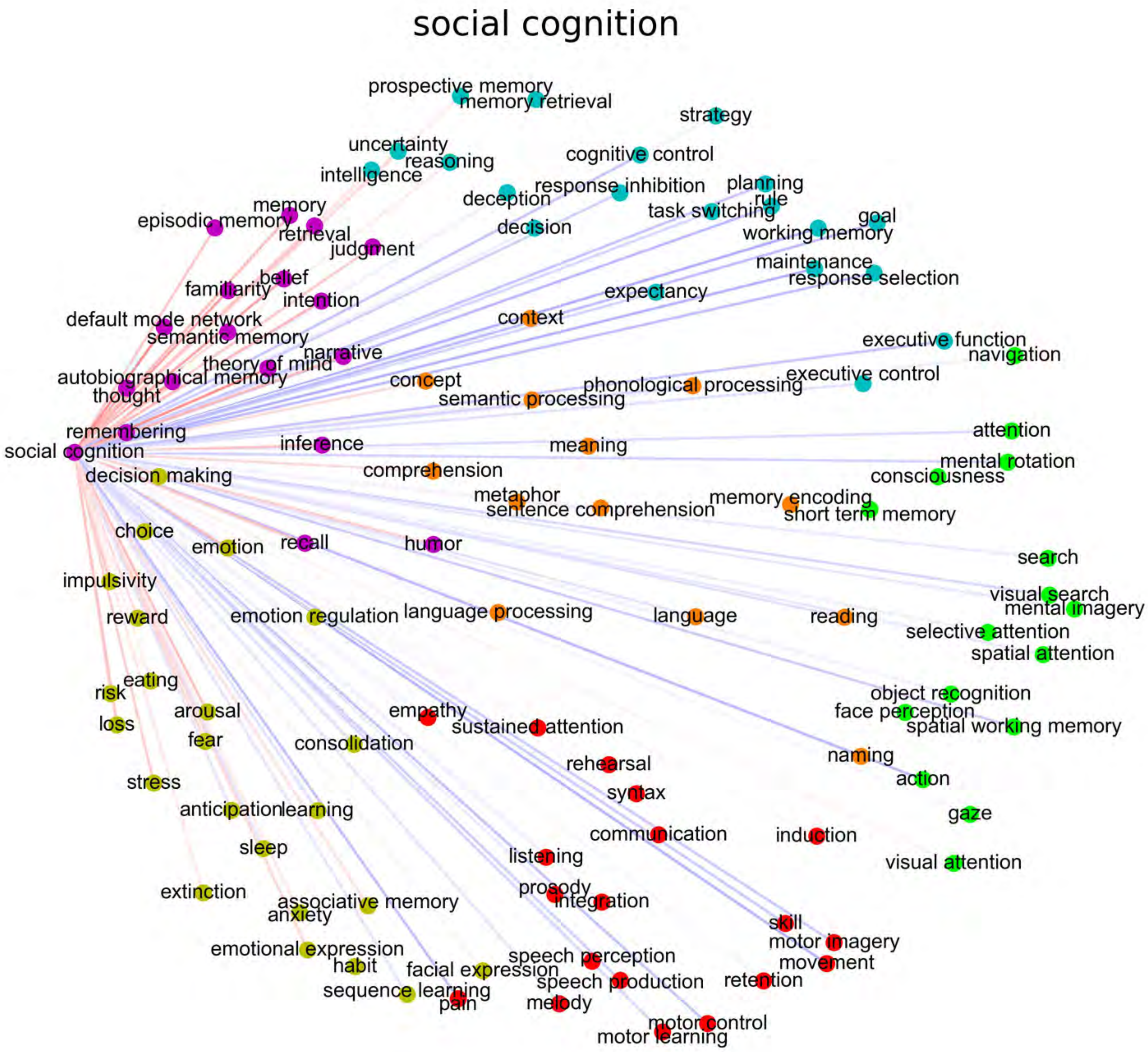
social cognition
prospective memory
memory retrieval
strategy
uncertainty
reasoning
cognitive control
intelligence
deception
response inhibition
planning
rule
memory
episodic memory
retrieval
judgment
task switching
goal
decision
working memory
maintenance
response selection
belief
familiarity
intention
expectancy
context
default mode network
semantic memory
executive function
navigation
narrative
theory of mind
concept
executive control
autobiographical memory
thought
phonological processing
semantic processing
attention
remembering
inference
meaning
mental rotation
decision making
comprehension
consciousness
metaphor
sentence comprehension
memory encoding
short term memory
choice
emotion
recall
humor
search
impulsivity
visual search
mental imagery
reward
emotion regulation
language processing
language
reading
selective attention
spatial attention
eating
risk
arousal
object recognition
face perception
empathy
loss
sustained attention
spatial working memory
fear
consolidation
rehearsal
naming
stress
action
syntax
anticipation
learning
gaze
communication
induction
sleep
listening
visual attention
extinction
prosody
integration
associative memory
anxiety
skill
motor imagery
emotional expression
habit
speech perception
movement
facial expression
speech production
retention
sequence learning
pain
melody
motor control
motor learning

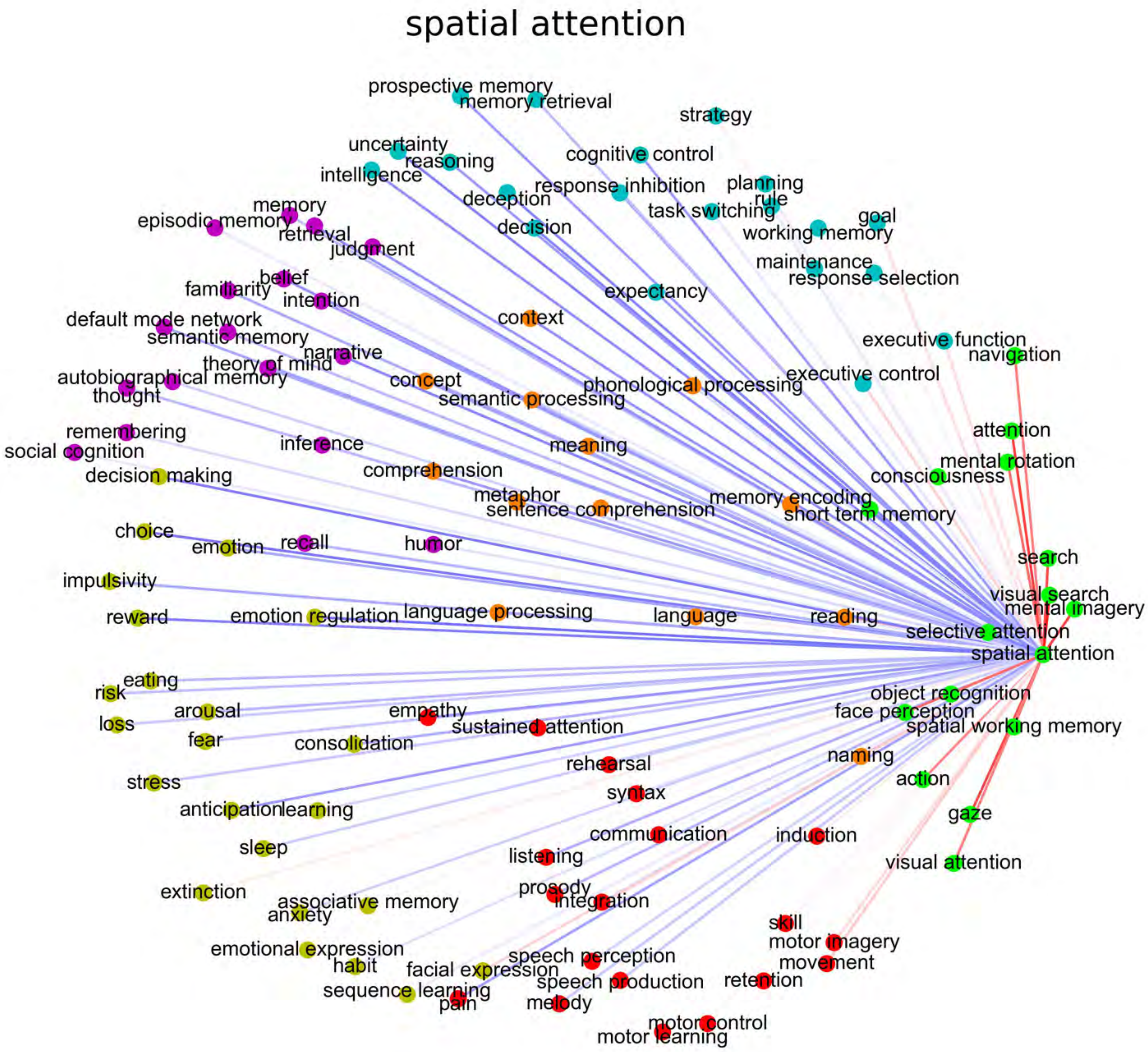
spatial attention
prospective memory
memory retrieval
strategy
uncertainty
reasoning
intelligence
cognitive control
response inhibition
planning
rule
deception
task switching
goal
decision
working memory
memory
episodic memory
retrieval
judgment
maintenance
response selection
belief
familiarity
intention
expectancy
default mode network
context
semantic memory
executive function
navigation
narrative
theory of mind
concept
phonological processing
executive control
autobiographical memory
thought
semantic processing
remembering
attention
inference
meaning
social cognition
mental rotation
decision making
comprehension
consciousness
metaphor
memory encoding
sentence comprehension
short term memory
choice
emotion
recall
humor
search
impulsivity
visual search
mental imagery
reward
emotion regulation
language processing
language
reading
selective attention
spatial attention
eating
risk
object recognition
arousal
empathy
face perception
loss
sustained attention
spatial working memory
fear
consolidation
rehearsal
naming
stress
action
syntax
anticipation
learning
gaze
communication
induction
sleep
listening
visual attention
extinction
prosody
integration
associative memory
anxiety
skill
motor imagery
emotional expression
speech perception
habit
movement
facial expression
speech production
retention
sequence learning
pain
melody
motor control
motor learning

spatial working memory

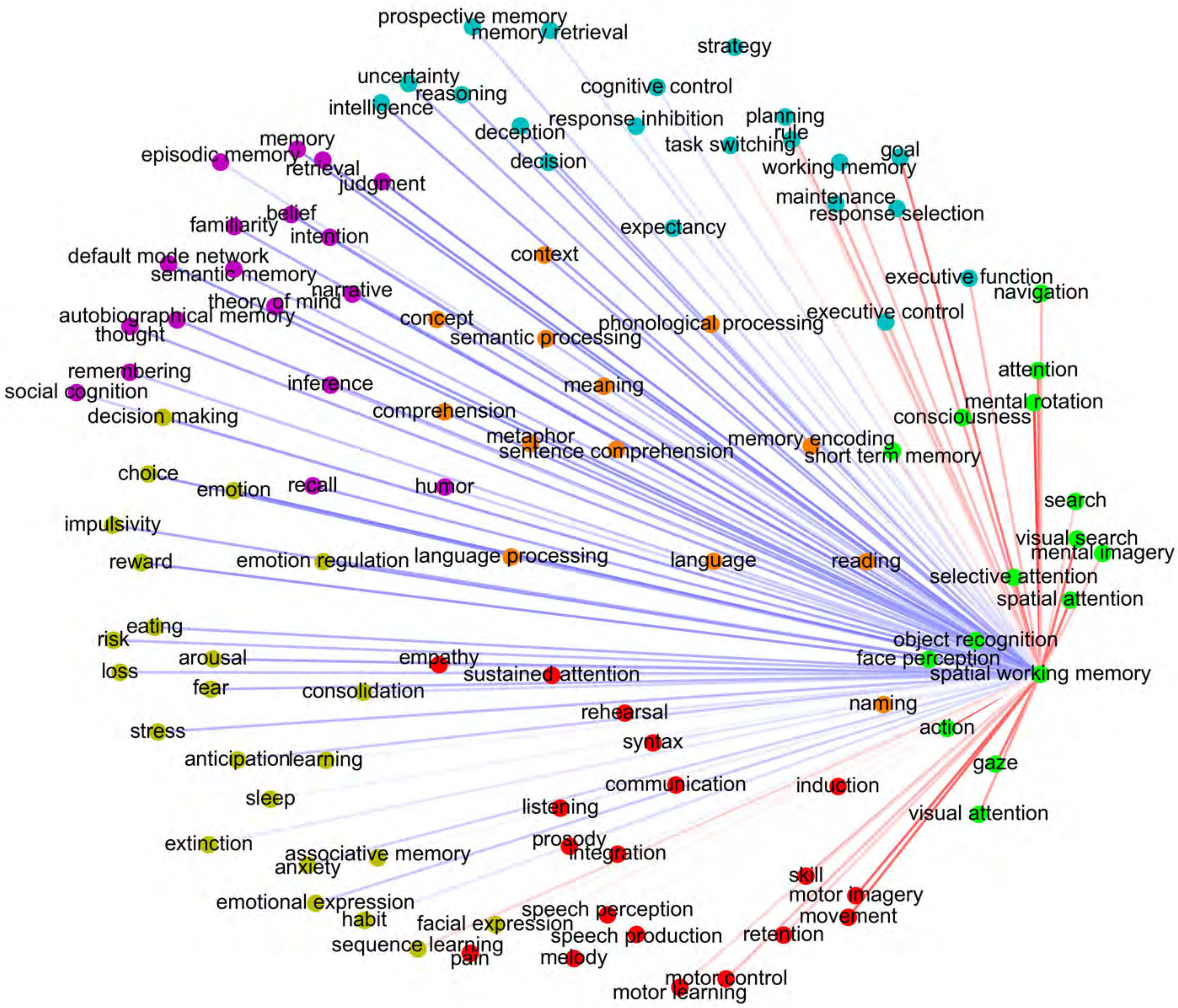

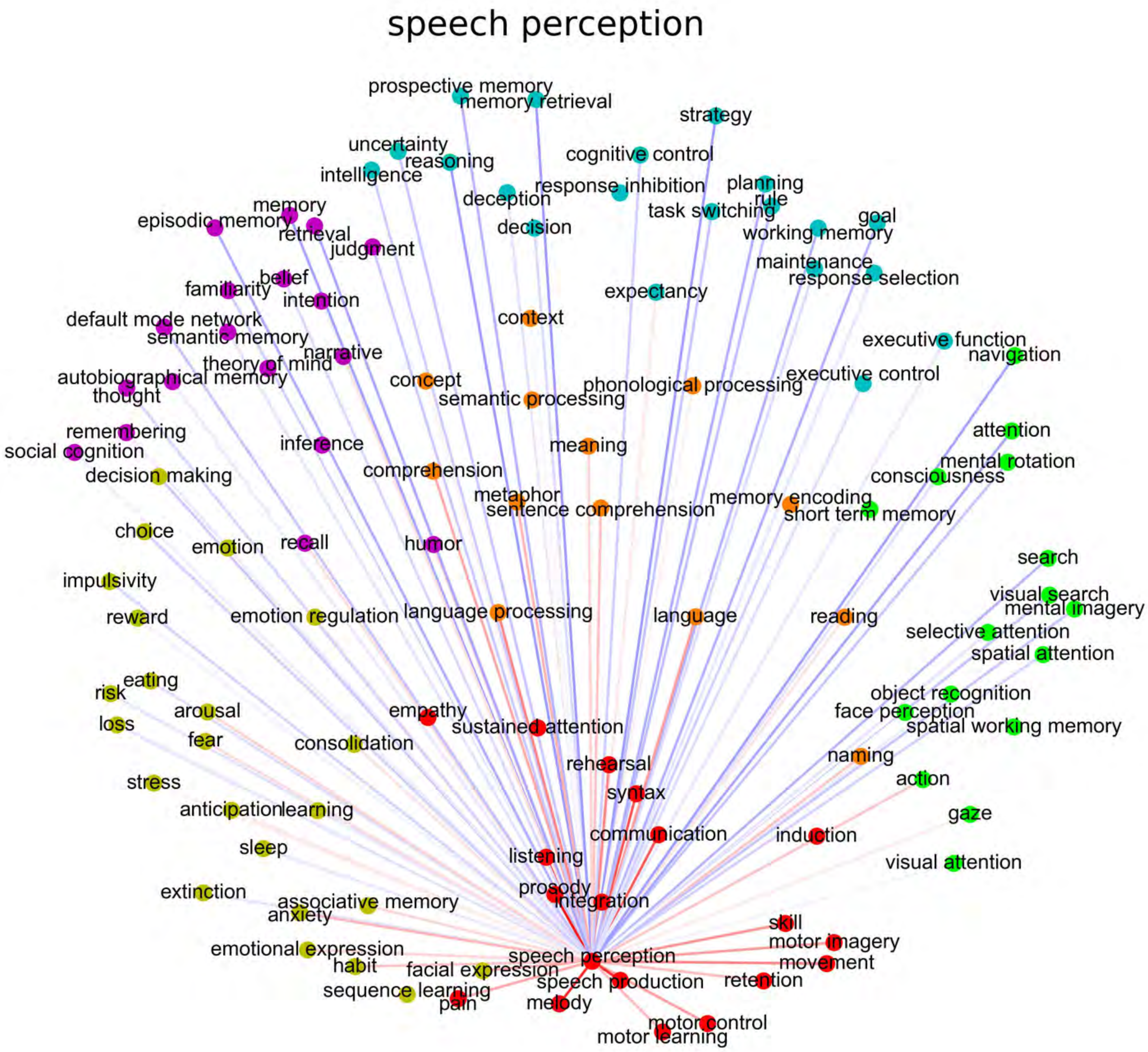

speech perception
prospective memory
memory retrieval
strategy
uncertainty
reasoning
cognitive control
intelligence
response inhibition
planning
deception
rule
task switching
memory
episodic memory
retrieval
decision
goal
working memory
judgment
maintenance
response selection
belief
familiarity
intention
expectancy
context
default mode network
semantic memory
executive function
navigation
narrative
theory of mind
concept
phonological processing
executive control
autobiographical memory
thought
semantic processing
attention
remembering
inference
meaning
social cognition
mental rotation
decision making
comprehension
consciousness
metaphor
memory encoding
sentence comprehension
short term memory
choice
emotion
recall
humor
search
impulsivity
visual search
mental imagery
reward
emotion regulation
language processing
language
reading
selective attention
spatial attention
eating
risk
object recognition
arousal
empathy
face perception
loss
sustained attention
spatial working memory
fear
consolidation
rehearsal
naming
stress
syntax
action
anticipation
learning
gaze
communication
induction
sleep
listening
visual attention
extinction
prosody
integration
associative memory
anxiety
skill
motor imagery
emotional expression
speech perception
movement
habit
facial expression
speech production
retention
sequence learning
pain
melody
motor control
motor learning

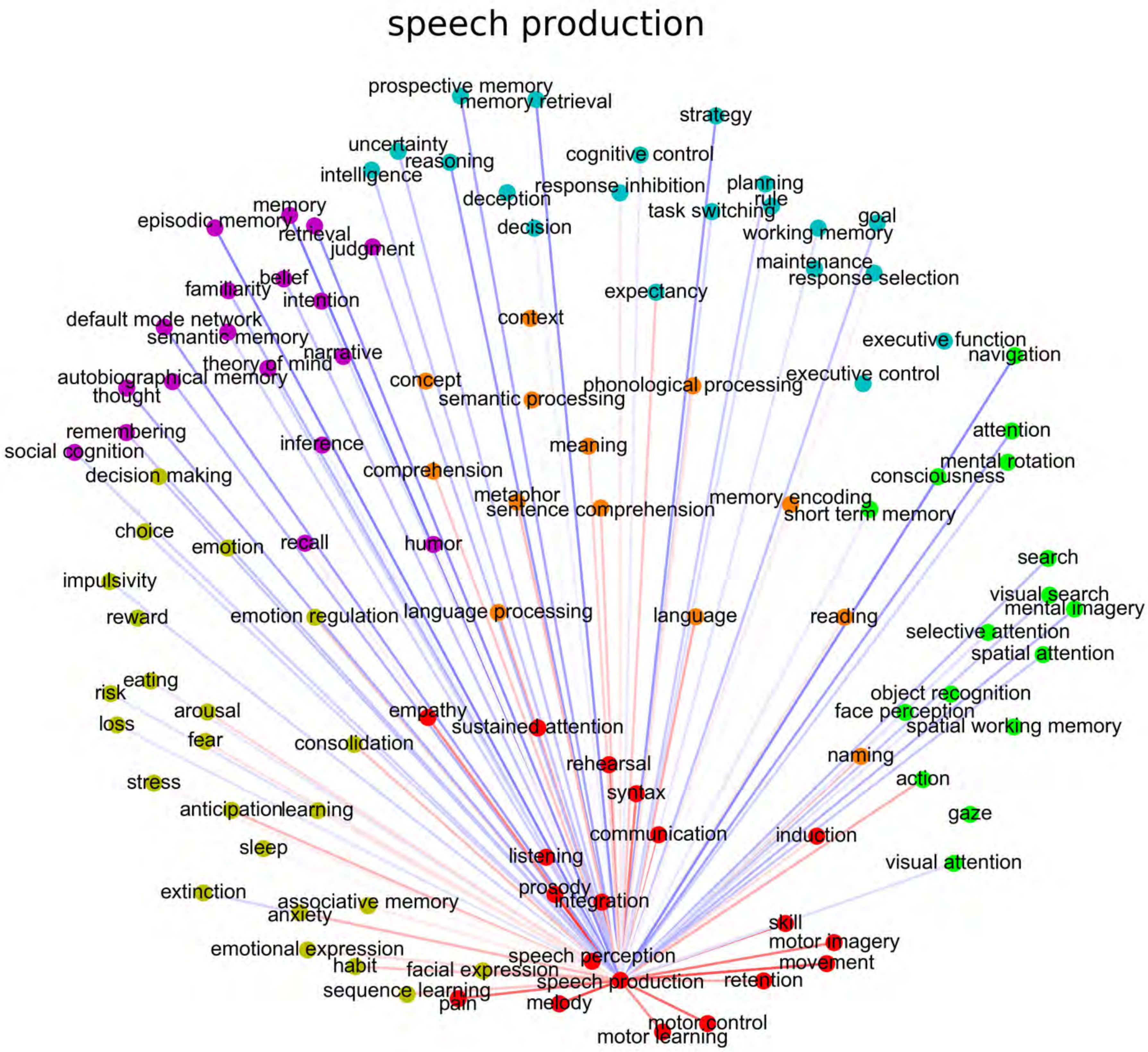
speech production
prospective memory
memory retrieval
strategy
uncertainty
reasoning
cognitive control
intelligence
response inhibition
planning
deception
rule
task switching
goal
memory
episodic memory
retrieval
decision
working memory
judgment
maintenance
response selection
belief
familiarity
intention
expectancy
context
default mode network
semantic memory
executive function
navigation
narrative
theory of mind
concept
phonological processing
executive control
autobiographical memory
thought
semantic processing
remembering
attention
inference
meaning
social cognition
mental rotation
decision making
comprehension
consciousness
metaphor
memory encoding
sentence comprehension
short term memory
choice
emotion
recall
humor
search
impulsivity
visual search
mental imagery
reward
emotion regulation
language processing
language
reading
selective attention
spatial attention
eating
risk
arousal
object recognition
empathy
face perception
loss
sustained attention
spatial working memory
fear
consolidation
rehearsal
naming
stress
action
syntax
anticipation
learning
gaze
communication
induction
sleep
listening
visual attention
extinction
prosody
integration
associative memory
anxiety
skill
motor imagery
emotional expression
speech perception
movement
habit
facial expression
speech production
retention
sequence learning
pain
melody
motor control
motor learning

strategy

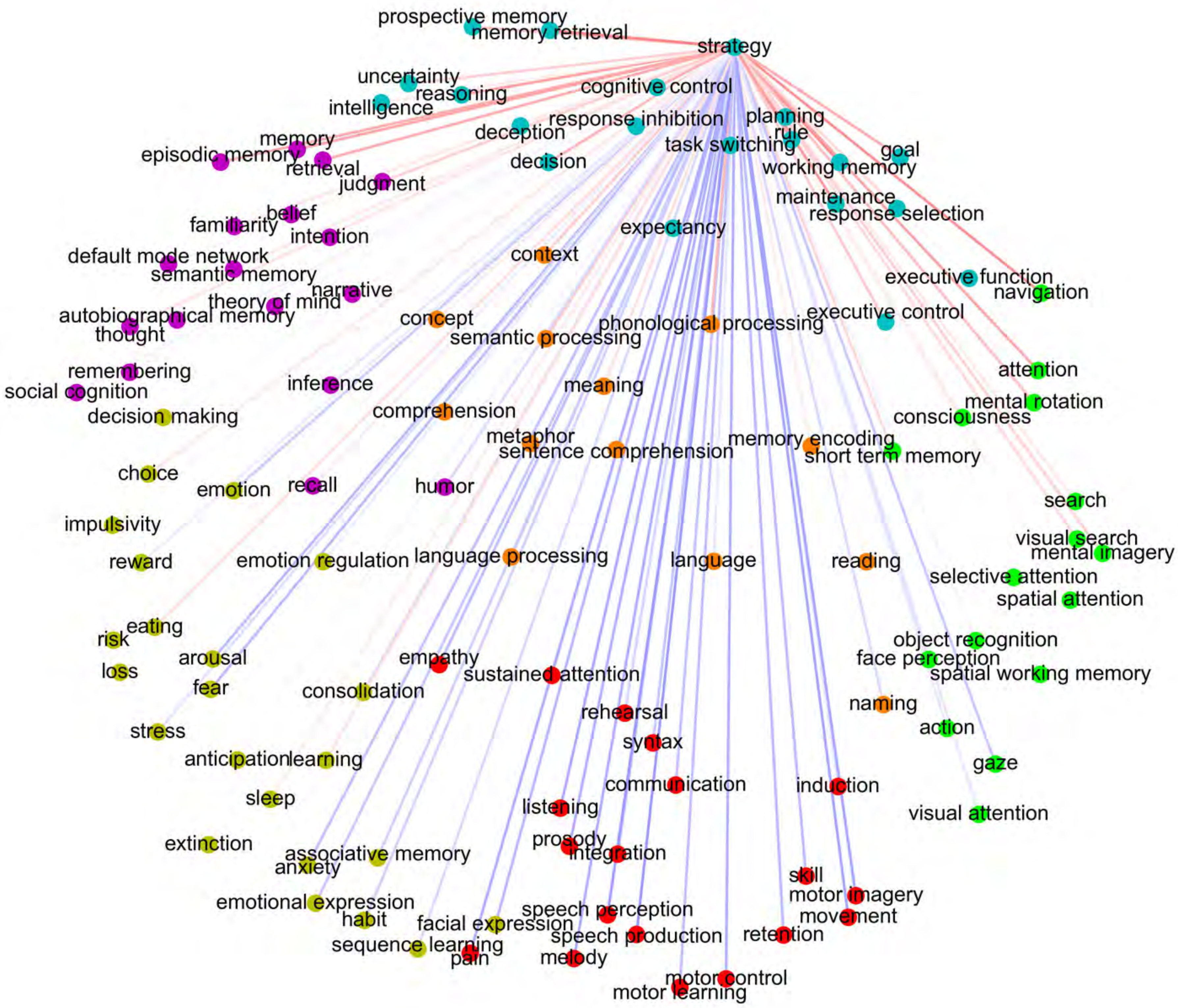

prospective memory
memory retrieval
strategy
uncertainty
reasoning
intelligence
cognitive control
response inhibition
planning
rule
deception
task switching
goal
memory
episodic memory
retrieval
decision
working memory
judgment
maintenance
response selection
belief
familiarity
intention
expectancy
default mode network
context
semantic memory
executive function
navigation
narrative
theory of mind
executive control
concept
phonological processing
autobiographical memory
thought
semantic processing
remembering
attention
social cognition
inference
meaning
mental rotation
decision making
comprehension
consciousness
metaphor
memory encoding
sentence comprehension
short term memory
choice
emotion
recall
humor
search
impulsivity
visual search
mental imagery
reward
emotion regulation
language processing
language
reading
selective attention
spatial attention
eating
risk
object recognition
arousal
empathy
face perception
loss
sustained attention
spatial working memory
fear
consolidation
naming
rehearsal
action
stress
syntax
anticipation
learning
gaze
communication
induction
sleep
listening
visual attention
prosody
extinction
integration
associative memory
anxiety
skill
motor imagery
emotional expression
speech perception
habit
movement
facial expression
speech production
retention
sequence learning
pain
melody
motor control
motor learning

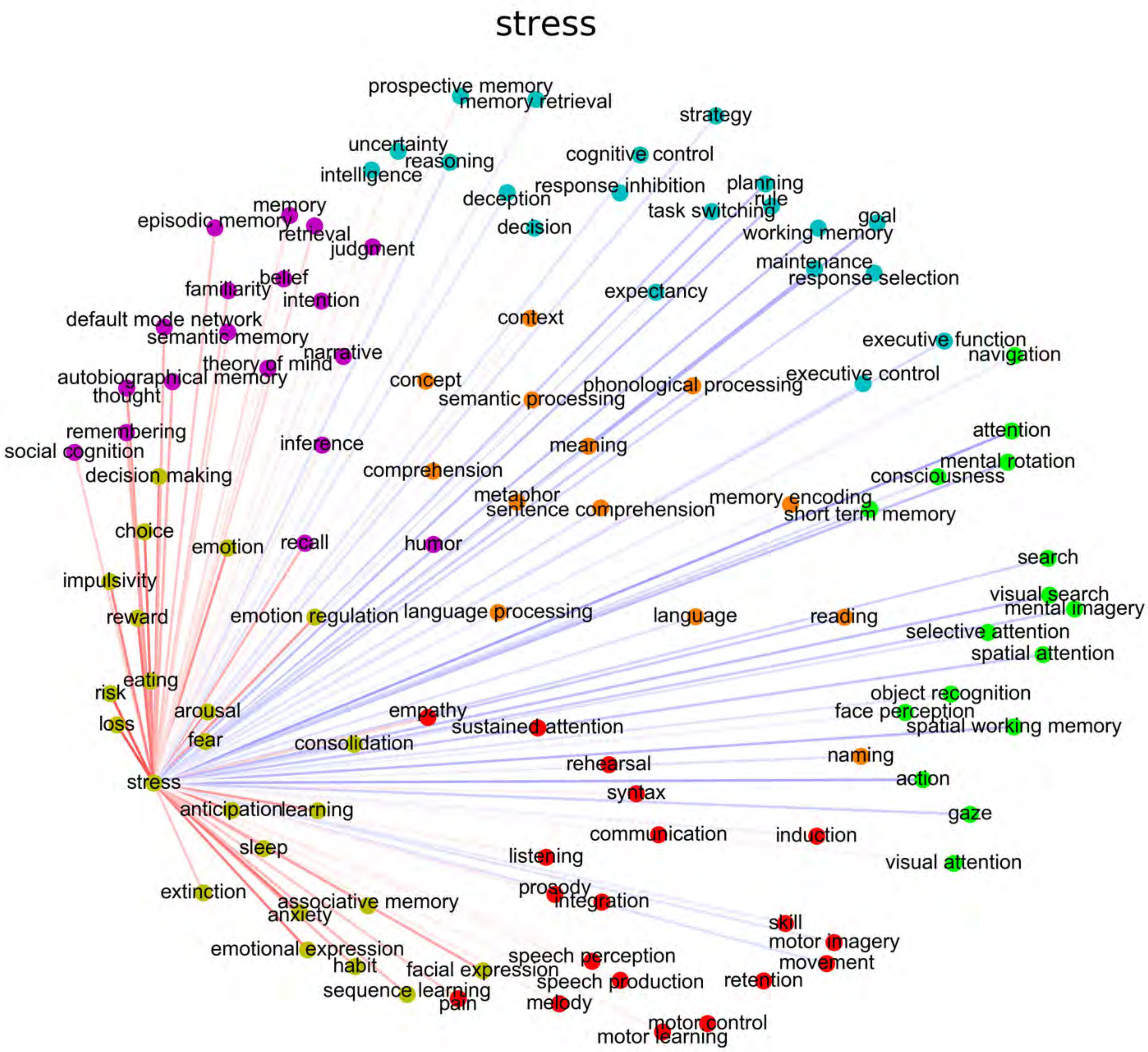
stress
prospective memory
memory retrieval
strategy
uncertainty
reasoning
cognitive control
intelligence
planning
response inhibition
deception
rule
memory
task switching
episodic memory
goal
retrieval
decision
working memory
judgment
maintenance
belief
response selection
familiarity
intention
expectancy
default mode network
context
semantic memory
executive function
navigation
narrative
theory of mind
executive control
concept
autobiographical memory
phonological processing
thought
semantic processing
remembering
attention
inference
meaning
social cognition
mental rotation
decision making
comprehension
consciousness
metaphor
memory encoding
sentence comprehension
short term memory
choice
emotion
recall
humor
search
impulsivity
visual search
mental imagery
emotion regulation
language processing
language
reading
reward
selective attention
spatial attention
eating
risk
object recognition
arousal
empathy
face perception
loss
sustained attention
spatial working memory
fear
consolidation
naming
rehearsal
stress
action
syntax
anticipation
learning
gaze
communication
induction
sleep
listening
visual attention
extinction
prosody
integration
associative memory
anxiety
skill
motor imagery
emotional expression
speech perception
movement
habit
facial expression
speech production
retention
sequence learning
pain
melody
motor control
motor learning

sustained attention

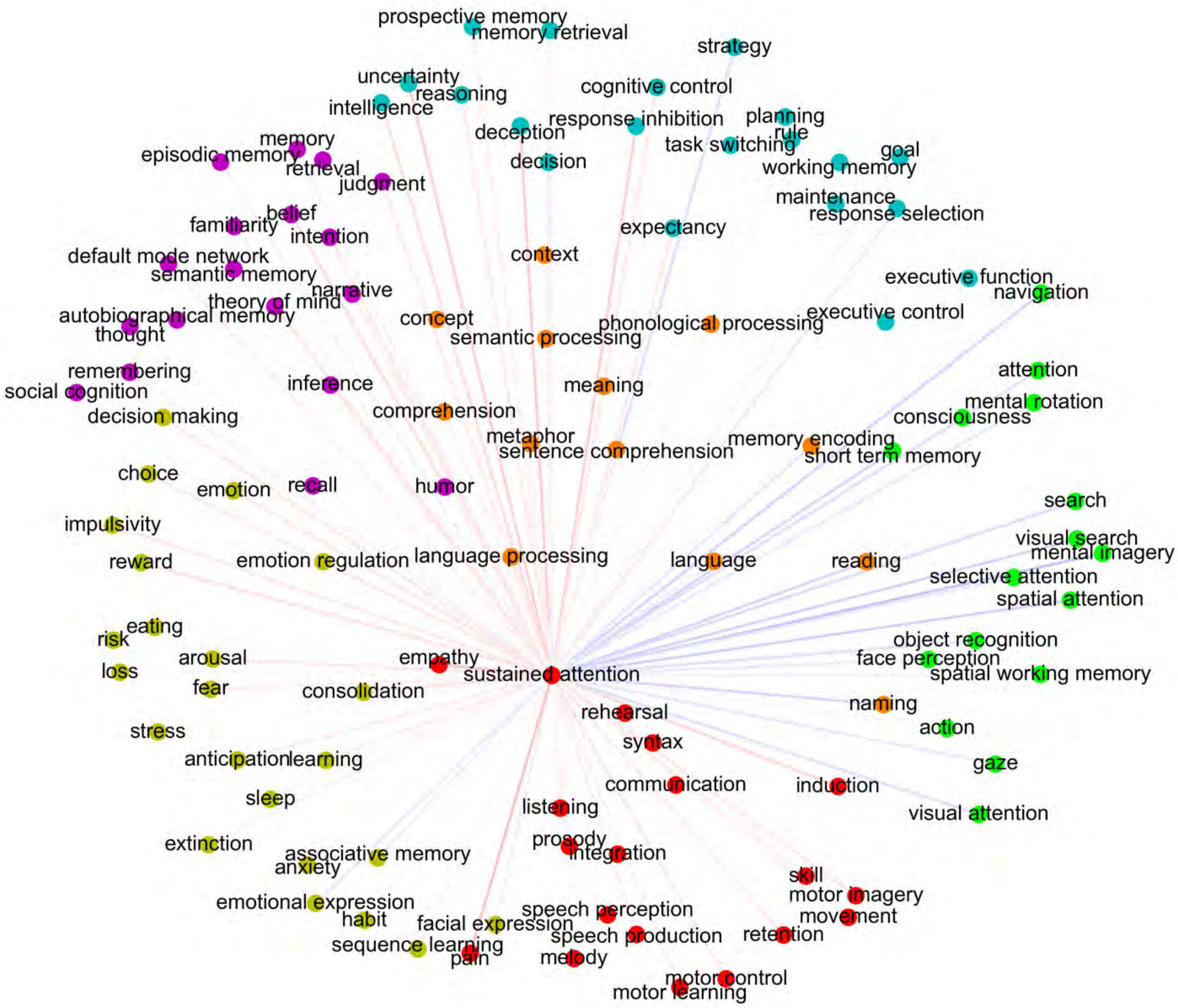

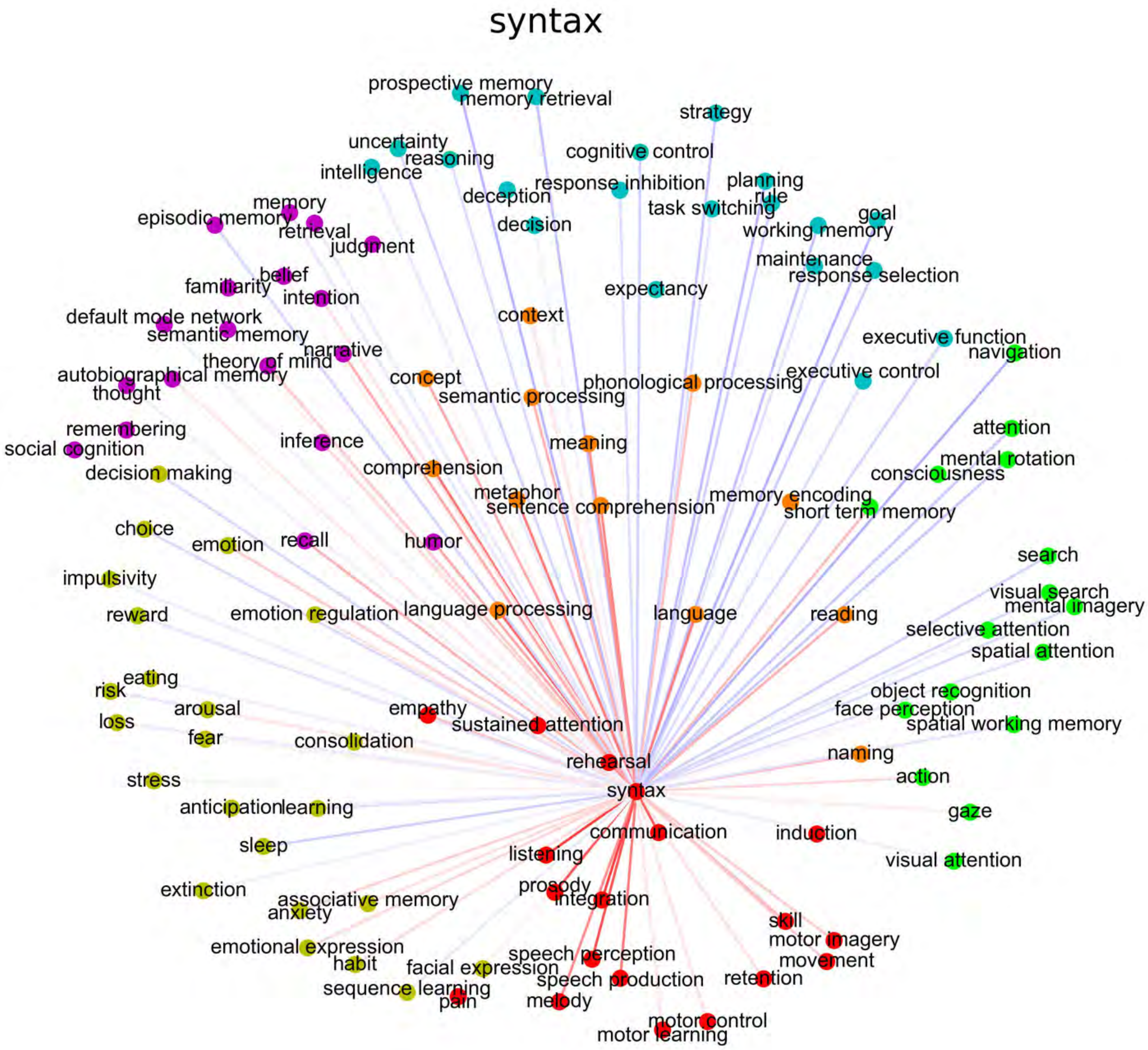
syntax
prospective memory
memory retrieval
strategy
uncertainty
reasoning
intelligence
cognitive control
response inhibition
planning
rule
deception
task switching
goal
working memory
decision
memory
episodic memory
retrieval
judgment
maintenance
response selection
belief
familiarity
intention
expectancy
context
default mode network
semantic memory
executive function
navigation
narrative
theory of mind
autobiographical memory
thought
concept
semantic processing
phonological processing
executive control
remembering
social cognition
inference
meaning
attention
mental rotation
comprehension
consciousness
decision making
metaphor
sentence comprehension
memory encoding
short term memory
choice
emotion
recall
humor
search
impulsivity
visual search
mental imagery
reward
emotion regulation
language processing
language
reading
selective attention
spatial attention
eating
risk
arousal
loss
fear
empathy
sustained attention
consolidation
object recognition
face perception
spatial working memory
rehearsal
naming
stress
action
syntax
anticipation
learning
gaze
sleep
communication
induction
listening
visual attention
extinction
prosody
integration
associative memory
anxiety
skill
motor imagery
emotional expression
habit
speech perception
facial expression
movement
speech production
retention
sequence learning
pain
melody
motor control
motor learning

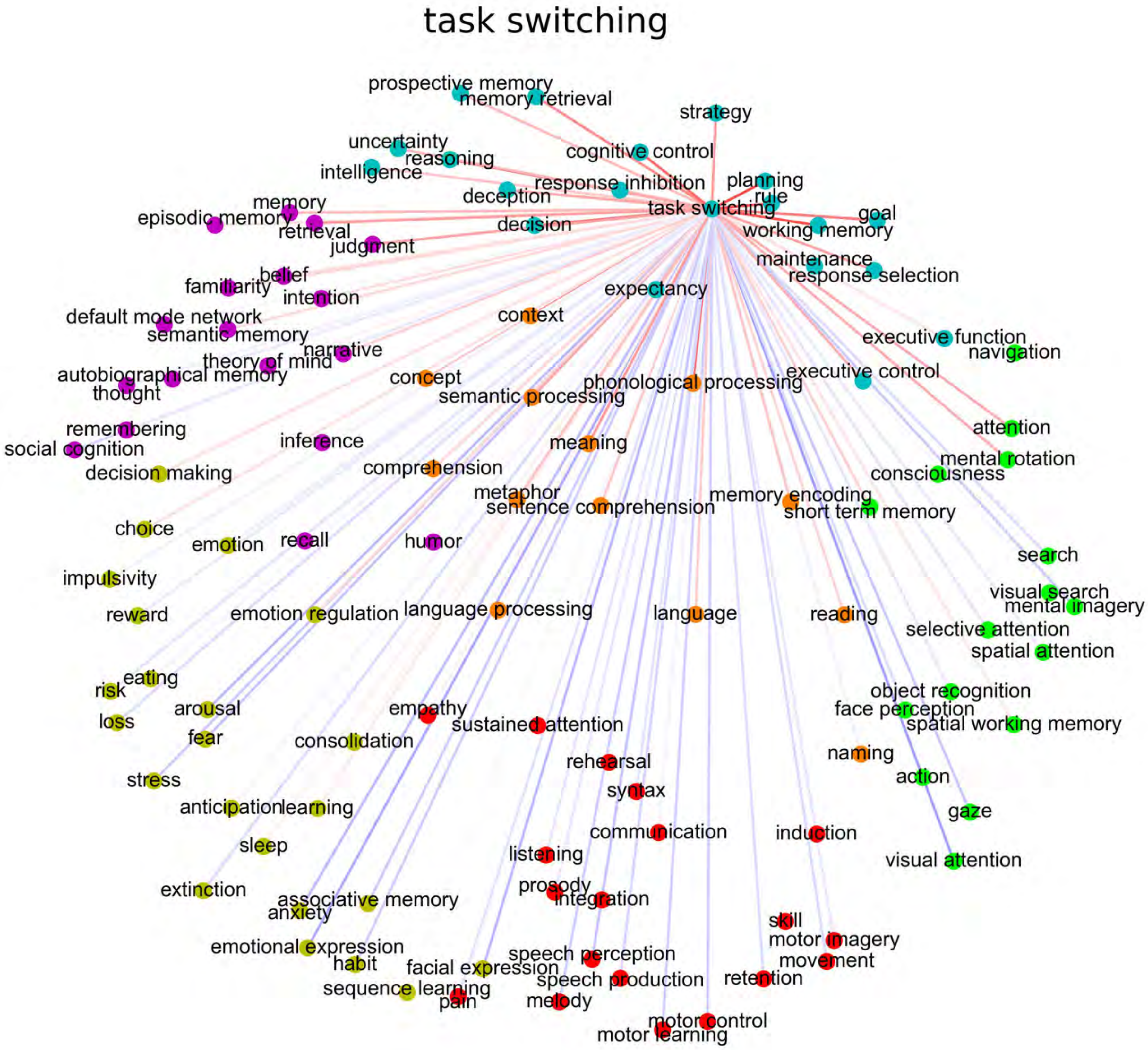
task switching
prospective memory
memory retrieval
strategy
uncertainty
reasoning
cognitive control
intelligence
response inhibition
planning
deception
rule
memory
task switching
goal
episodic memory
retrieval
decision
working memory
judgment
maintenance
belief
response selection
familiarity
intention
expectancy
default mode network
context
semantic memory
executive function
narrative
navigation
theory of mind
autobiographical memory
concept
executive control
phonological processing
thought
semantic processing
remembering
attention
inference
meaning
social cognition
mental rotation
decision making
comprehension
consciousness
metaphor
memory encoding
sentence comprehension
short term memory
choice
emotion
recall
humor
search
impulsivity
visual search
mental imagery
reward
emotion regulation
language processing
language
reading
selective attention
spatial attention
eating
risk
object recognition
arousal
empathy
face perception
loss
sustained attention
spatial working memory
fear
consolidation
naming
rehearsal
action
stress
syntax
anticipation
learning
gaze
communication
induction
sleep
listening
visual attention
extinction
prosody
integration
associative memory
anxiety
skill
motor imagery
emotional expression
speech perception
movement
habit
facial expression
speech production
retention
sequence learning
pain
melody
motor control
motor learning

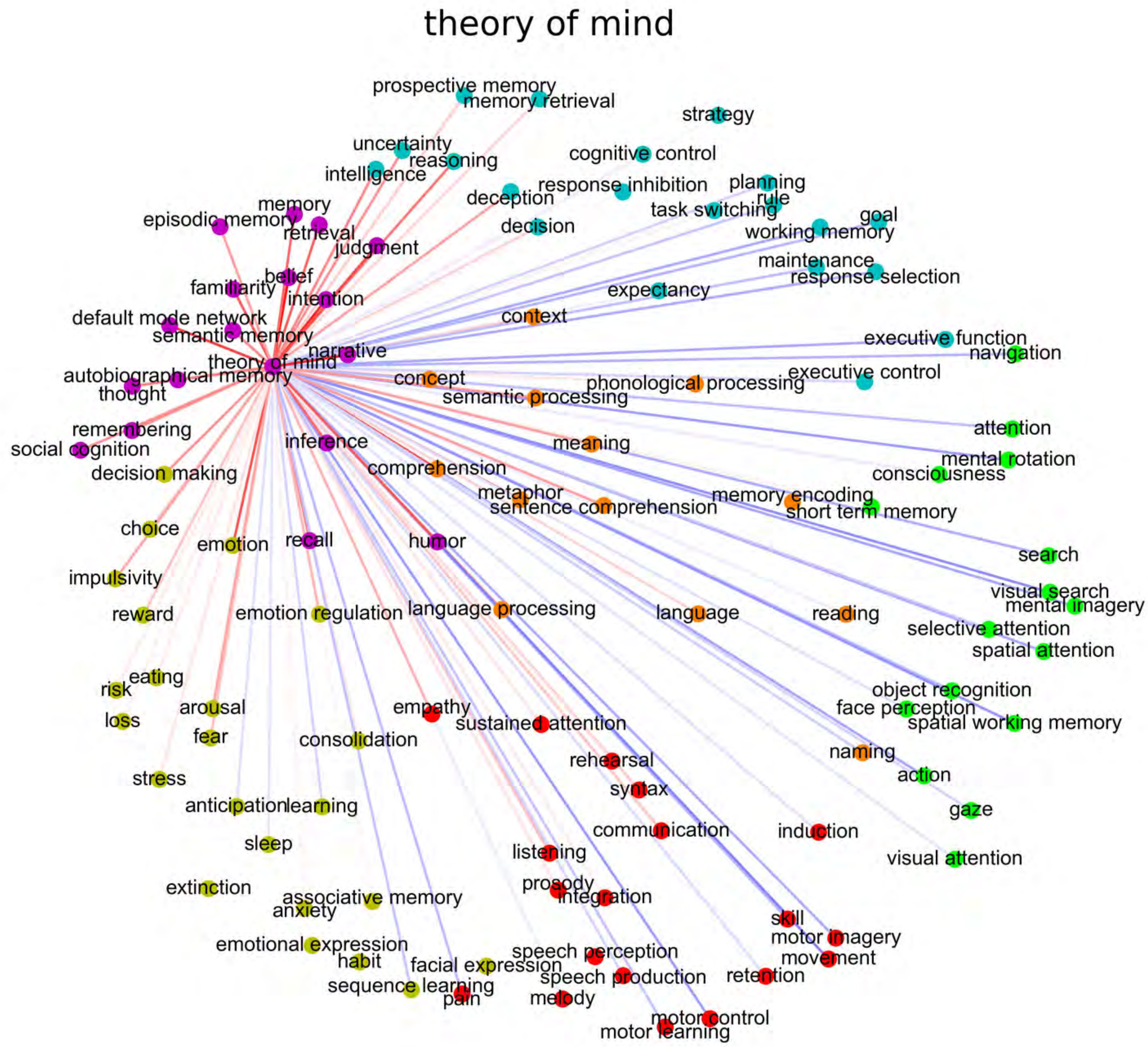
theory of mind
prospective memory
memory retrieval
strategy
uncertainty
reasoning
cognitive control
intelligence
response inhibition
planning
deception
rule
task switching
memory
episodic memory
retrieval
decision
goal
working memory
judgment
maintenance
response selection
belief
familiarity
intention
expectancy
default mode network
context
semantic memory
executive function
narrative
navigation
theory of mind
executive control
autobiographical memory
concept
phonological processing
thought
semantic processing
remembering
attention
social cognition
inference
meaning
mental rotation
decision making
comprehension
consciousness
metaphor
memory encoding
sentence comprehension
short term memory
choice
emotion
recall
humor
search
impulsivity
visual search
mental imagery
reward
emotion regulation
language processing
language
reading
selective attention
spatial attention
object recognition
risk
eating
face perception
arousal
empathy
spatial working memory
loss
sustained attention
fear
consolidation
naming
rehearsal
action
stress
syntax
gaze
anticipation
learning
communication
induction
sleep
listening
visual attention
extinction
prosody
integration
associative memory
anxiety
skill
motor imagery
emotional expression
movement
habit
speech perception
facial expression
speech production
retention
sequence learning
pain
melody
motor control
motor learning

thought

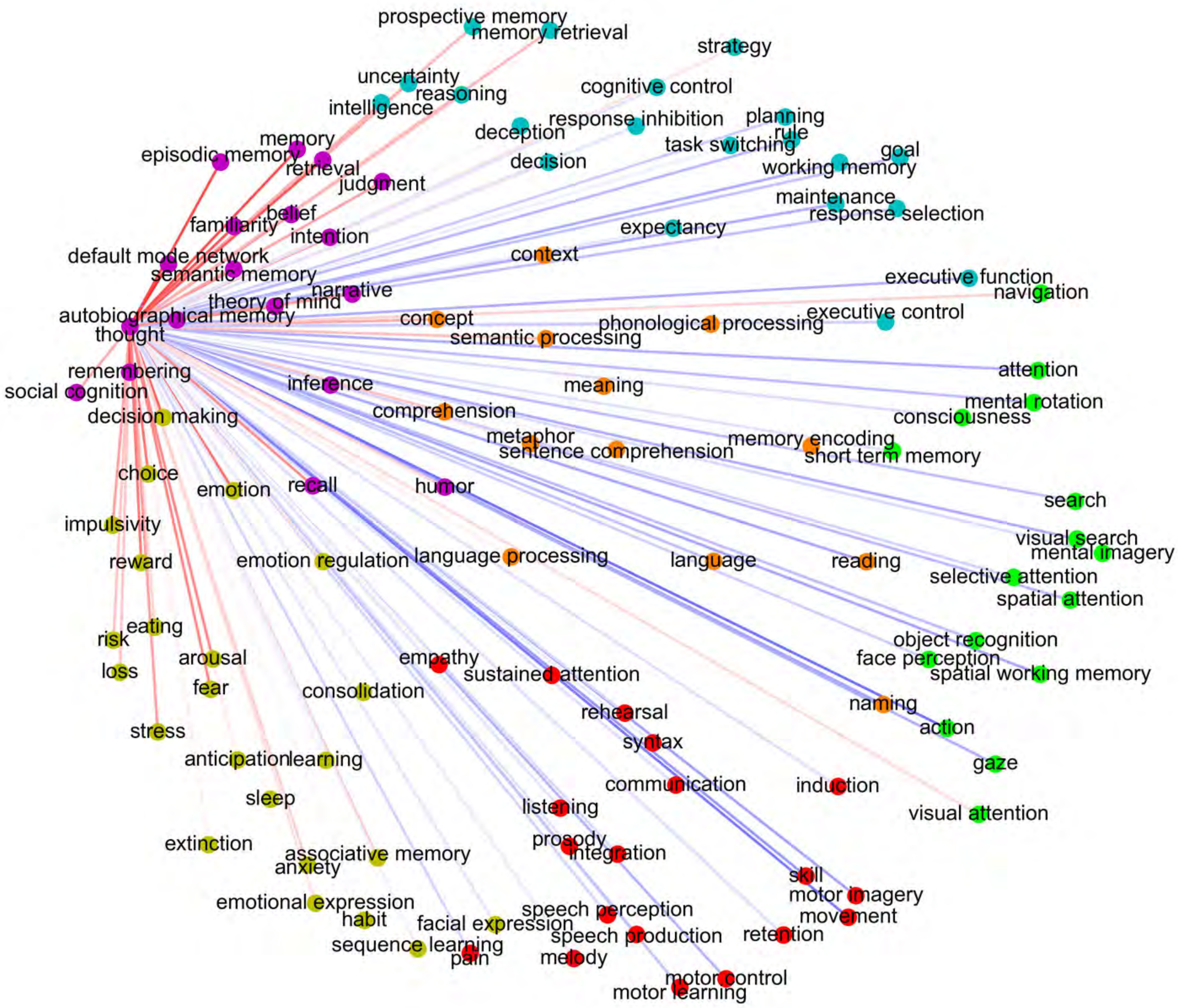
prospective memory
memory retrieval
strategy
uncertainty
reasoning
cognitive control
intelligence
response inhibition
planning
deception
rule
memory
task switching
goal
episodic memory
retrieval
decision
working memory
judgment
maintenance
belief
response selection
familiarity
intention
expectancy
default mode network
context
semantic memory
executive function
narrative
navigation
theory of mind
executive control
autobiographical memory
concept
phonological processing
thought
semantic processing
remembering
attention
social cognition
inference
meaning
decision making
comprehension
mental rotation
consciousness
metaphor
memory encoding
sentence comprehension
short term memory
choice
emotion
recall
humor
search
impulsivity
visual search
mental imagery
reward
emotion regulation
language processing
language
reading
selective attention
spatial attention
eating
risk
object recognition
arousal
empathy
face perception
loss
sustained attention
spatial working memory
fear
consolidation
rehearsal
naming
stress
action
syntax
anticipation
learning
gaze
communication
induction
sleep
listening
visual attention
extinction
prosody
integration
associative memory
anxiety
skill
motor imagery
emotional expression
movement
habit
speech perception
facial expression
speech production
retention
sequence learning
pain
melody
motor control
motor learning

uncertainty

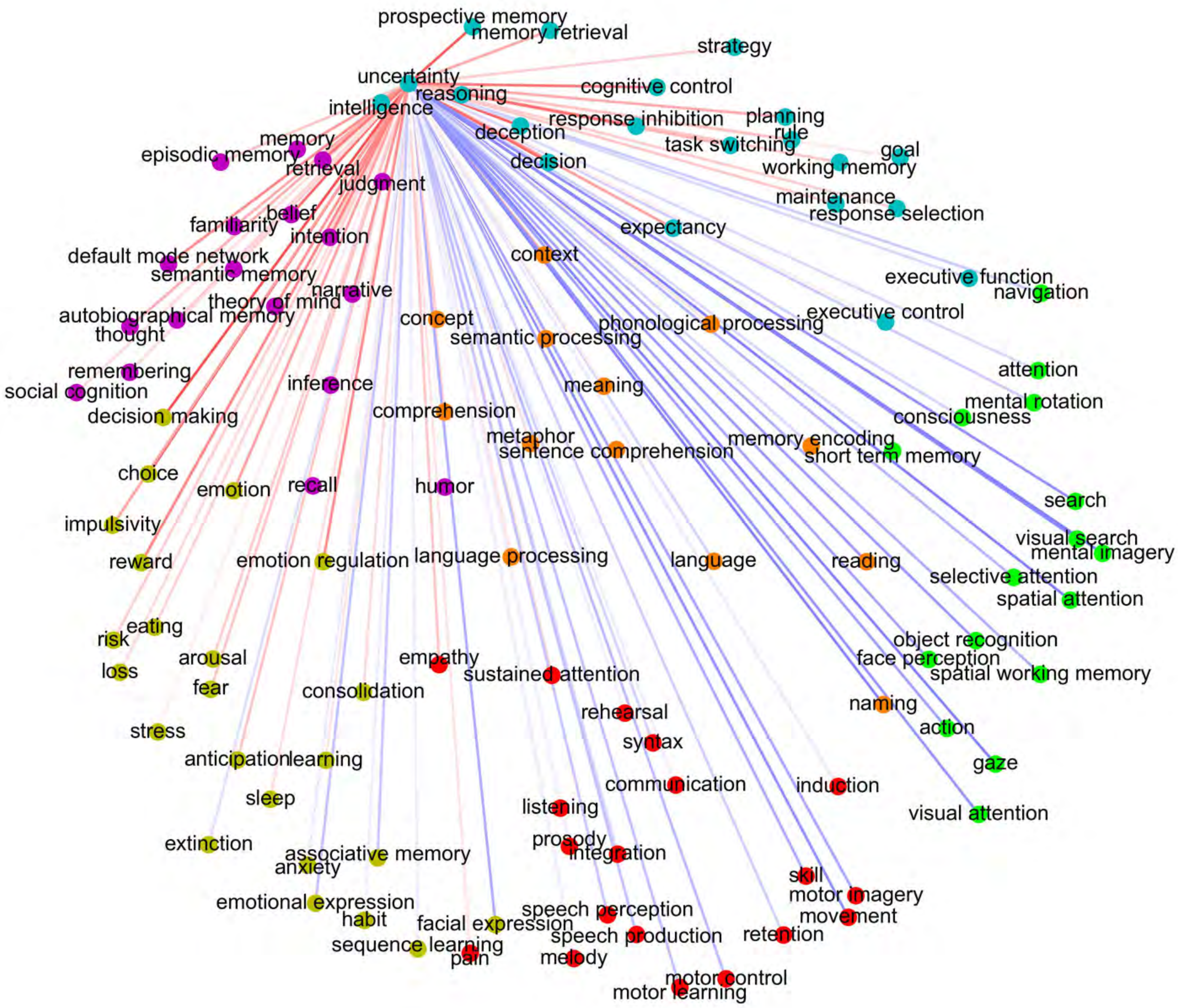

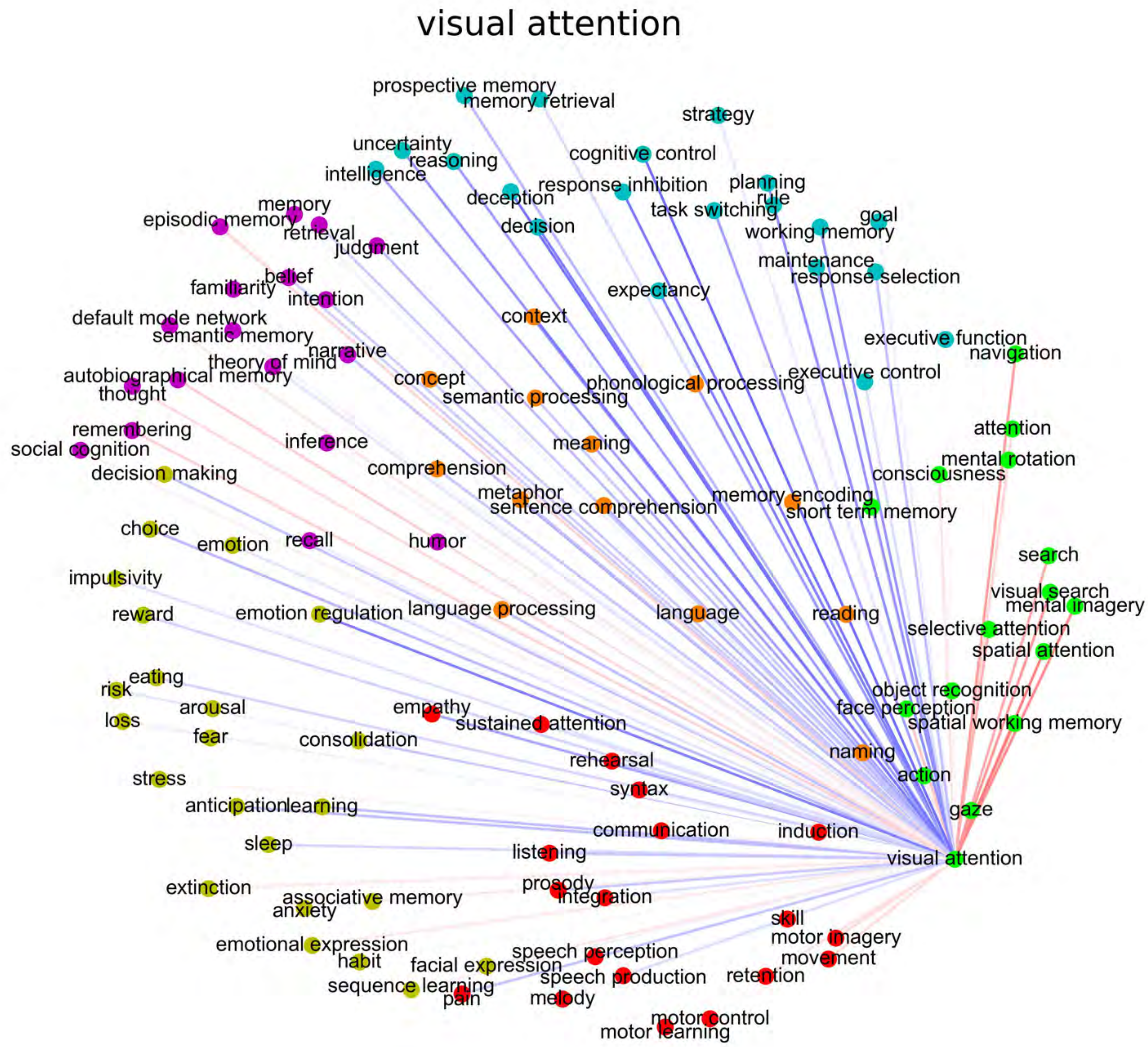
visual attention
prospective memory
memory retrieval
strategy
uncertainty
reasoning
cognitive control
intelligence
planning
response inhibition
rule
deception
task switching
goal
memory
episodic memory
retrieval
decision
working memory
judgment
maintenance
response selection
belief
familiarity
intention
expectancy
default mode network
context
semantic memory
executive function
navigation
narrative
theory of mind
executive control
concept
phonological processing
autobiographical memory
thought
semantic processing
remembering
attention
inference
meaning
social cognition
mental rotation
decision making
comprehension
consciousness
metaphor
memory encoding
sentence comprehension
short term memory
choice
recall
humor
emotion
search
impulsivity
visual search
mental imagery
reward
emotion regulation
language processing
language
reading
selective attention
spatial attention
eating
risk
object recognition
arousal
empathy
face perception
loss
sustained attention
spatial working memory
fear
consolidation
rehearsal
naming
stress
action
syntax
anticipation
learning
gaze
communication
induction
sleep
listening
visual attention
extinction
prosody
associative memory
integration
anxiety
skill
motor imagery
emotional expression
speech perception
habit
movement
facial expression
speech production
retention
sequence learning
pain
melody
motor control
motor learning

visual search

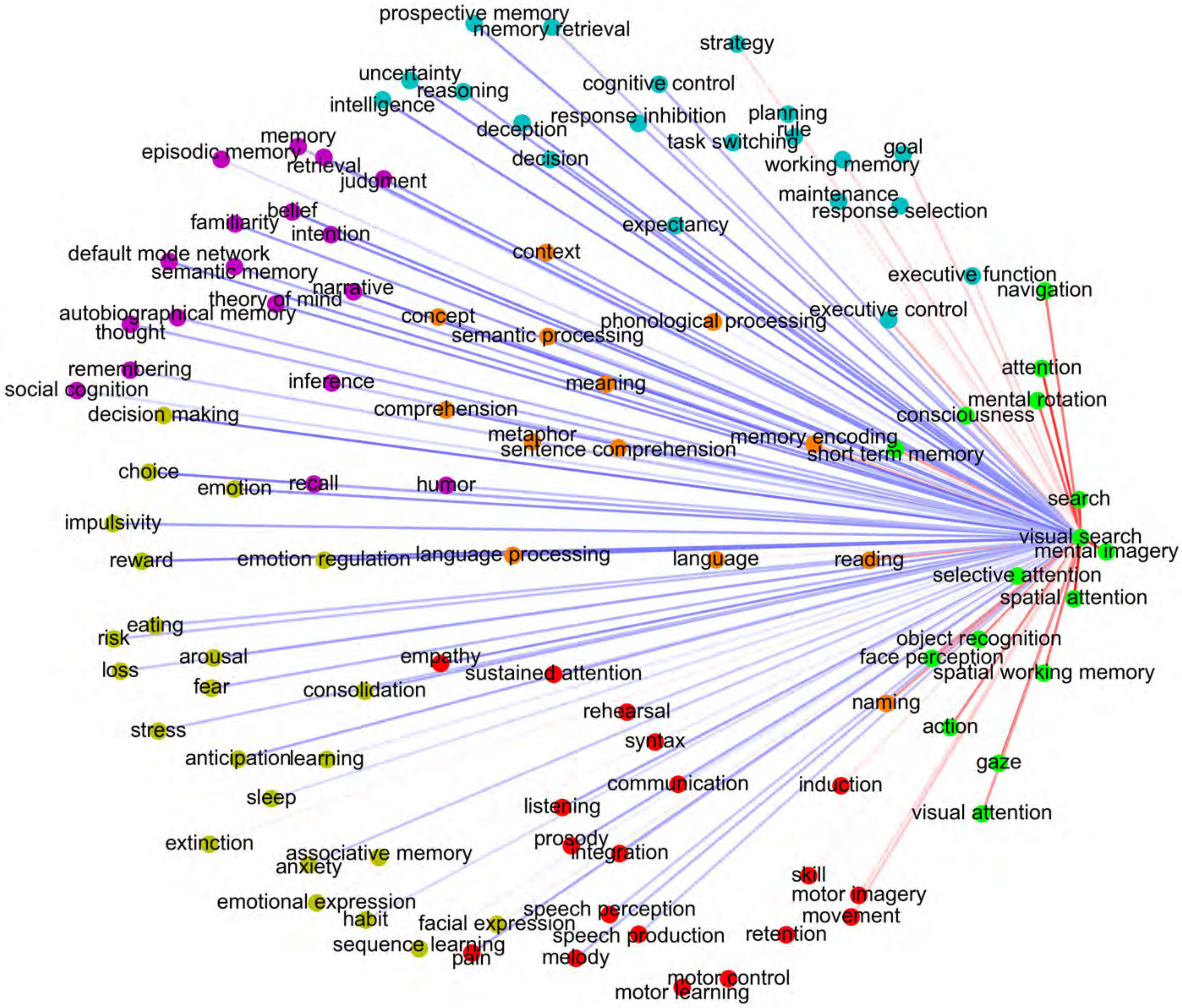

working memory

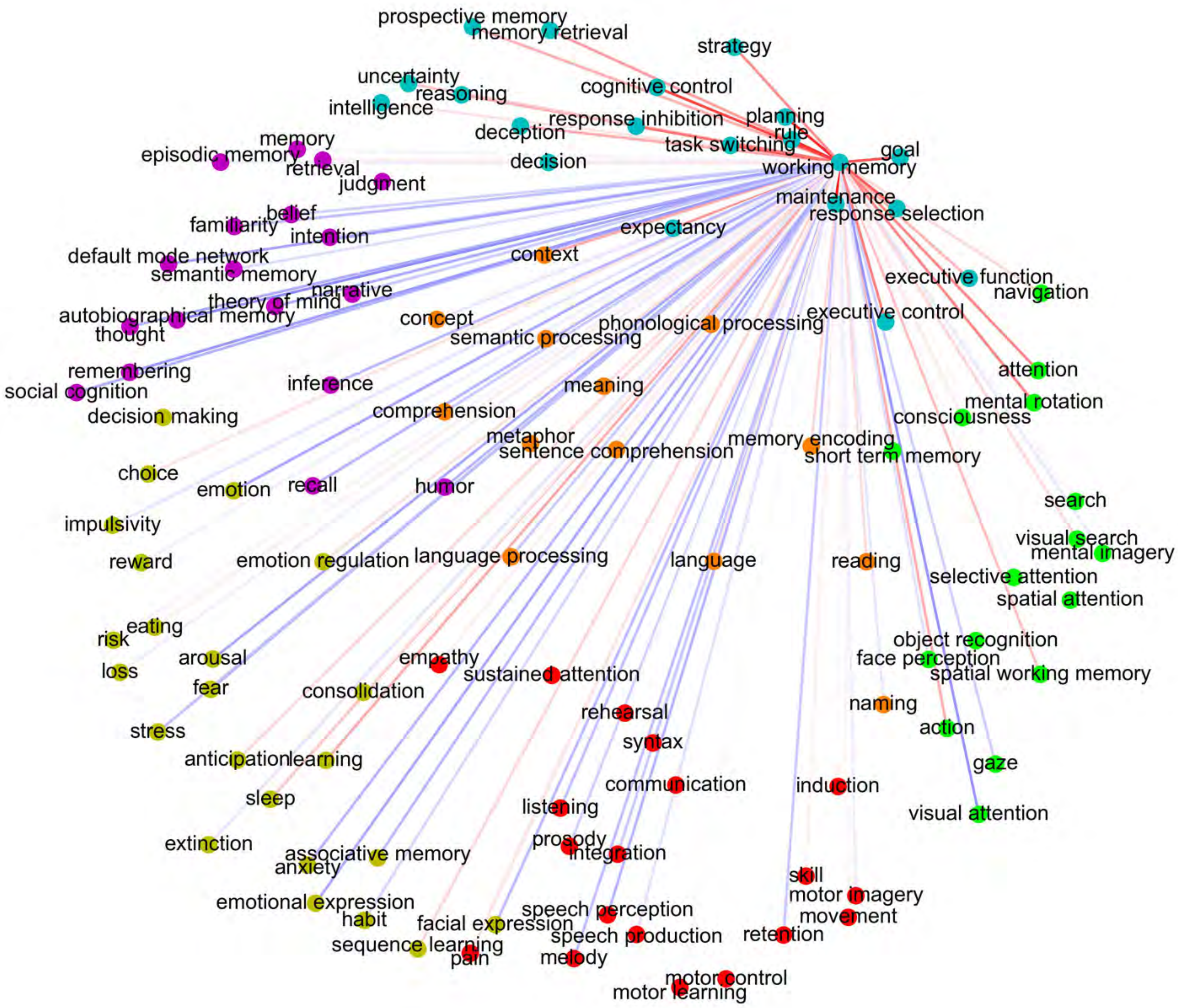

memory encoding

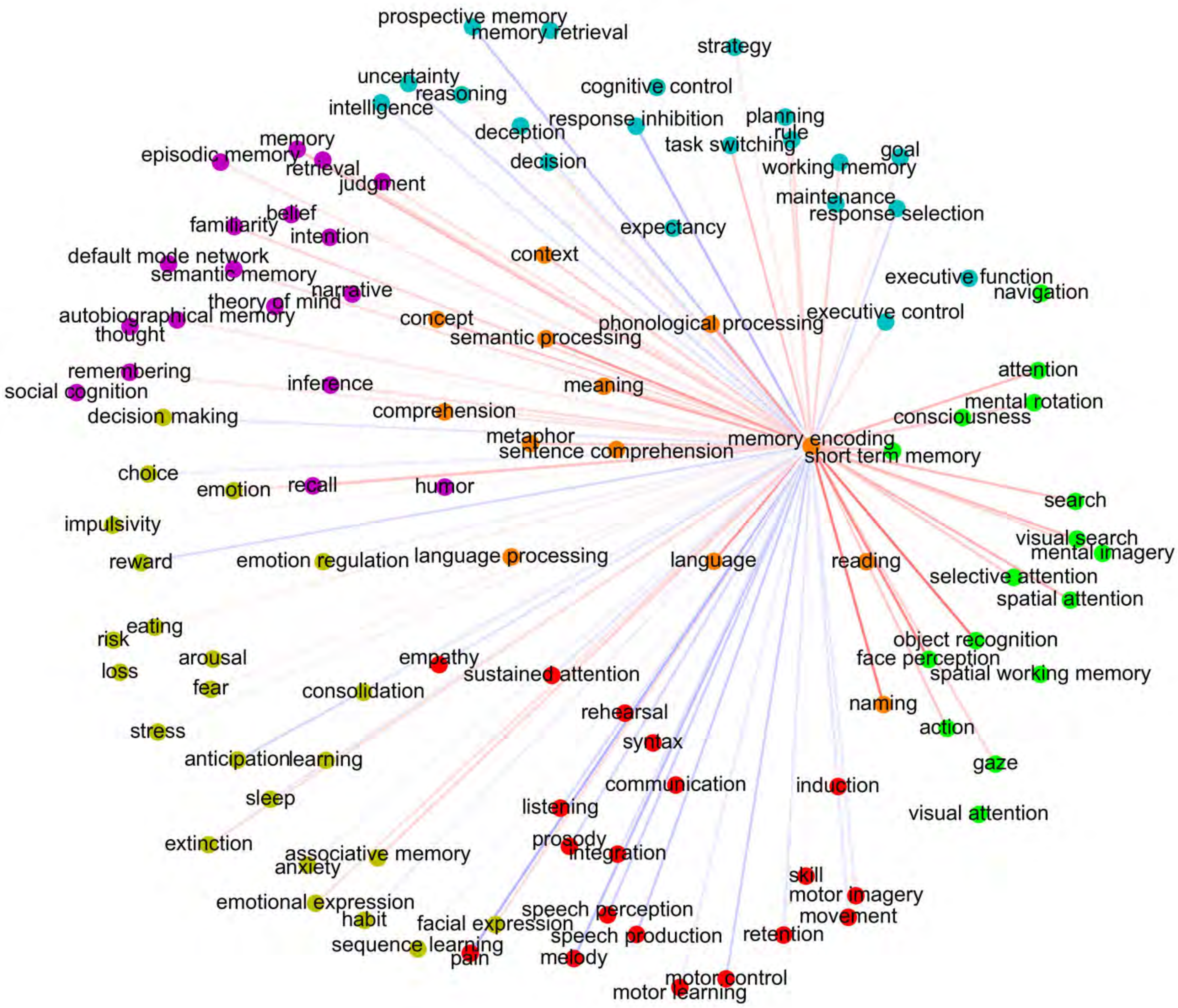

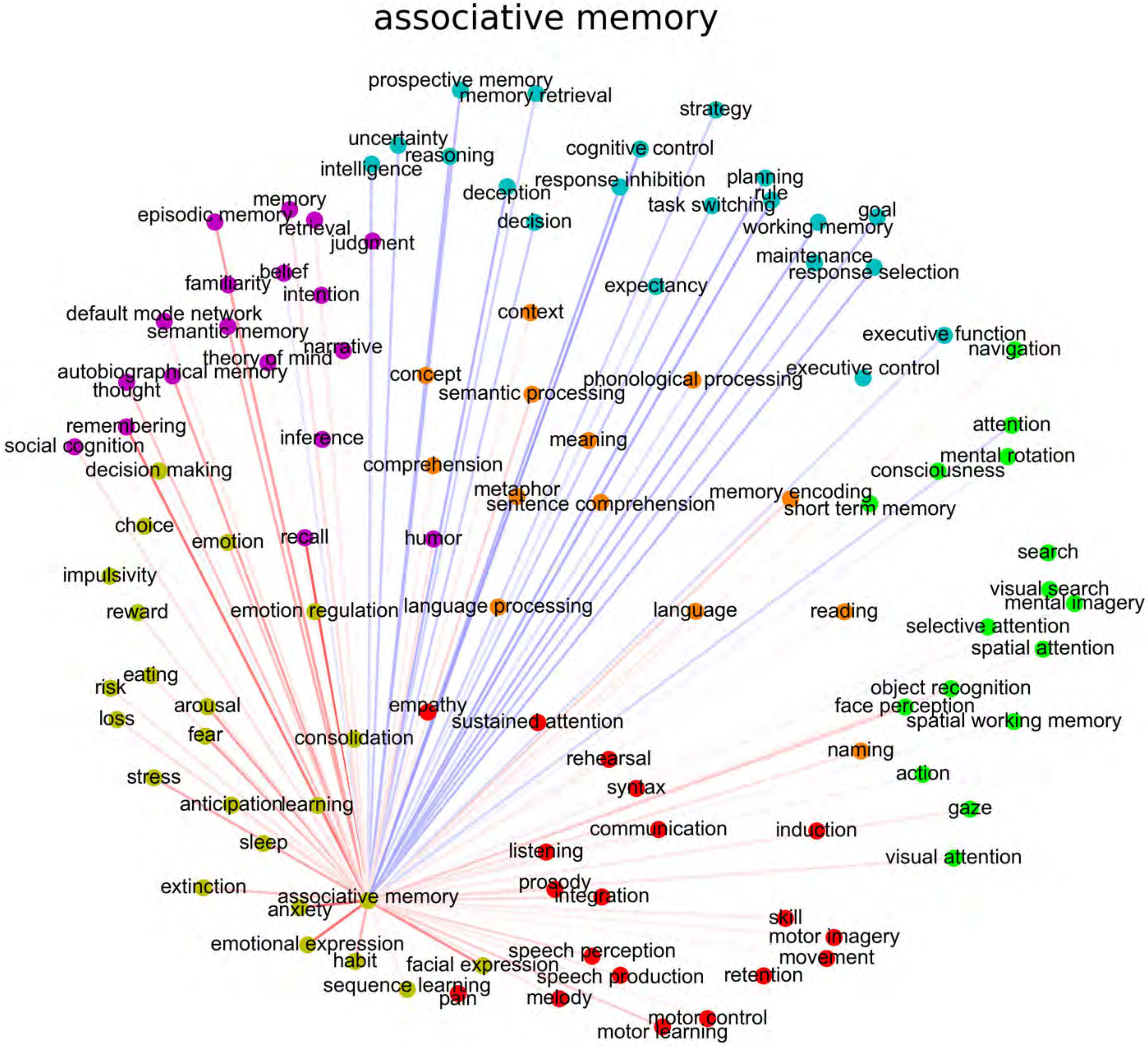
associative memory
prospective memory
memory retrieval
strategy
uncertainty
reasoning
cognitive control
intelligence
response inhibition
planning
rule
deception
memory
episodic memory
retrieval
task switching
goal
decision
working memory
judgment
maintenance
belief
response selection
familiarity
intention
expectancy
default mode network
context
semantic memory
executive function
navigation
narrative
theory of mind
concept
executive control
autobiographical memory
phonological processing
thought
semantic processing
remembering
attention
social cognition
inference
meaning
mental rotation
decision making
comprehension
consciousness
metaphor
memory encoding
sentence comprehension
short term memory
choice
emotion
recall
humor
search
impulsivity
visual search
mental imagery
reward
emotion regulation
language processing
language
reading
selective attention
spatial attention
eating
risk
arousal
object recognition
empathy
face perception
loss
sustained attention
spatial working memory
fear
consolidation
rehearsal
naming
stress
syntax
action
anticipation
learning
gaze
communication
induction
sleep
listening
visual attention
extinction
prosody
integration
anxiety
skill
motor imagery
emotional expression
speech perception
habit
movement
facial expression
speech production
retention
sequence learning
pain
melody
motor control
motor learning

motor imagery

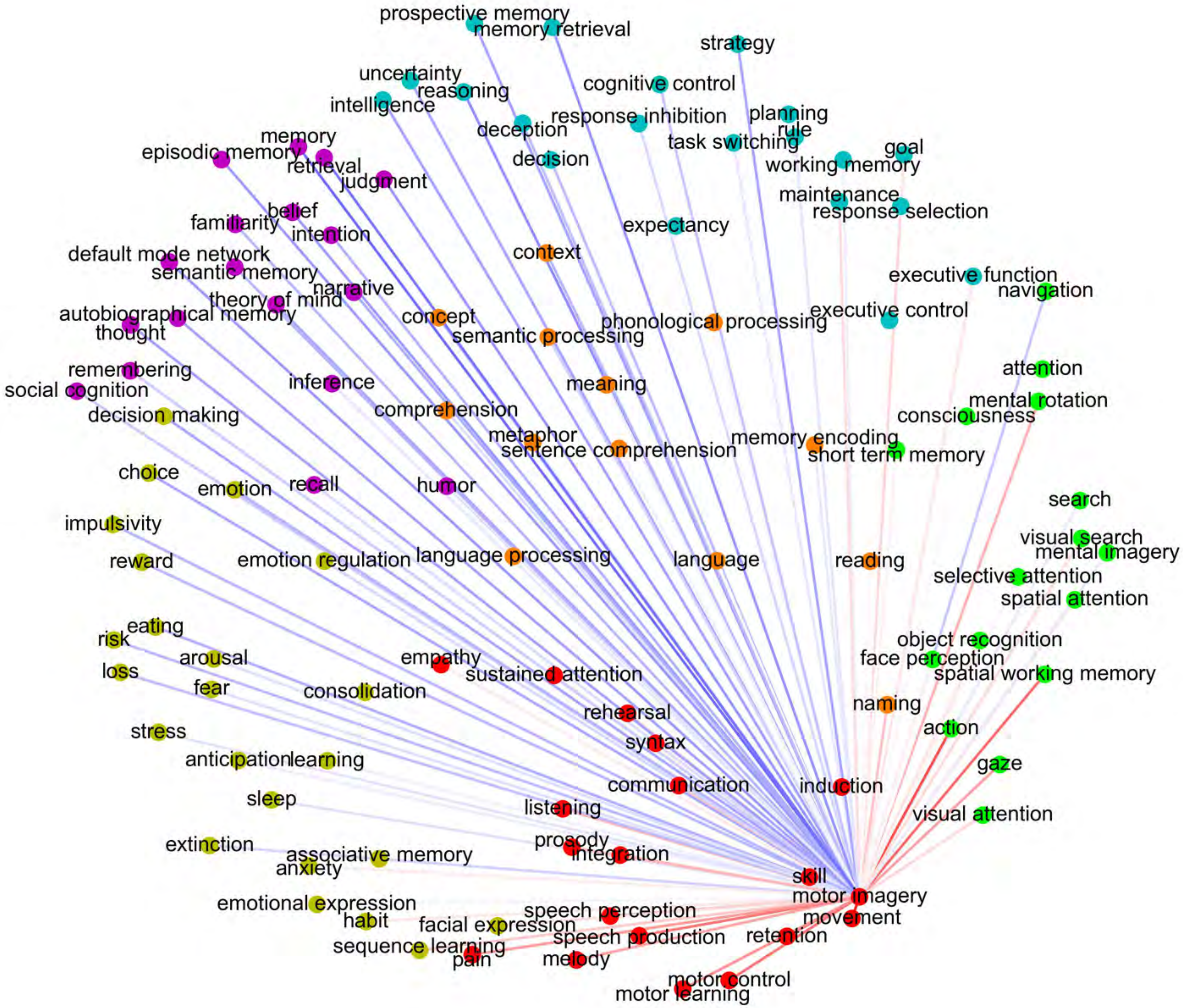

Supplement: Supplementary file 21 [file Image_7.PDF]
