# Supplementary material for: Revealing Relationships Among Cognitive Functions Using Functional Connectivity and a Large-Scale Meta-Analysis Database
Source: Front Hum Neurosci. 2020 Jan 10;13:457. doi: 10.3389/fnhum.2019.00457 (PMC6965330; doi:10.3389/fnhum.2019.00457)
Supplement: Supplementary file 22 [file Image_8.PDF]

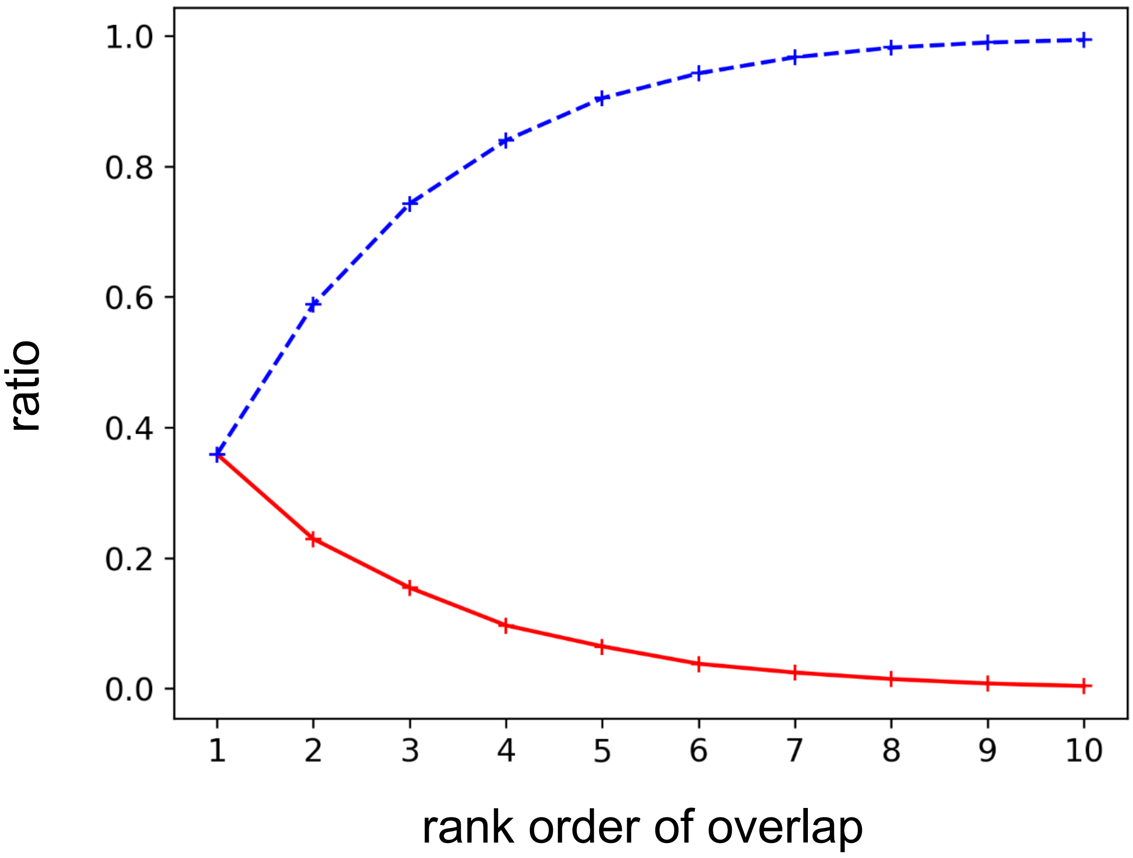

Supplementary Figure 8: Ratios of the number of voxels assigned to the Glasser's parcels ordered by the amounts of overlap. First, the number of voxels in each intersection between the parcels of the present and Glasser's parcellation was counted as shown in Table S6. According to the numbers of voxels in the intersections, for each parcel in the present parcellation, the Glasser's parcels in the table were sorted. Thus, in the sorted table, the first, second, third, … rows corresponded to the most overlapping parcel, the second most overlapping parcel, the third most overlapping parcel, …, respectively. Then, we calculated row sum of the table followed by dividing the values by the total sum, resulting in the ratios of the number of voxels assigned to the Glasser's parcels ordered by the amounts of overlap (solid line). The cumulative ratios are shown using a dashed line.
